# Aromatic Rings as Molecular Determinants for the Molecular Recognition of Protein Kinase Inhibitors

**DOI:** 10.3390/molecules26061776

**Published:** 2021-03-22

**Authors:** Yan Zhu, Saad Alqahtani, Xiche Hu

**Affiliations:** 1 Department of Chemistry and Biochemistry, University of Toledo, Toledo, OH 43606, USA; yan.zhu2@rockets.utoledo.edu (Y.Z.); salqahtani2@ksu.edu.sa (S.A.); 2 Department of Chemistry, King Saud University, Riyadh 12372, Saudi Arabia

**Keywords:** protein kinase inhibitor, aromatic rings, molecular recognition, π–π stacking interactions, CH–π interactions, rational drug design

## Abstract

Protein kinases are key enzymes in many signal transduction pathways, and play a crucial role in cellular proliferation, differentiation, and various cell regulatory processes. However, aberrant function of kinases has been associated with cancers and many other diseases. Consequently, competitive inhibition of the ATP binding site of protein kinases has emerged as an effective means of curing these diseases. Over the past three decades, thousands of protein kinase inhibitors (PKIs) with varying molecular frames have been developed. Large-scale data mining of the Protein Data Bank resulted in a database of 2139 non-redundant high-resolution X-ray crystal structures of PKIs bound to protein kinases. This provided us with a unique opportunity to study molecular determinants for the molecular recognition of PKIs. A chemoinformatic analysis of 2139 PKIs resulted in findings that PKIs are “flat” molecules with high aromatic ring counts and low fractions of sp^3^ carbon. All but one PKI possessed one or more aromatic rings. More importantly, it was found that the average weighted hydrogen bond count is inversely proportional to the number of aromatic rings. Based on this linear relationship, we put forward the exchange rule of hydrogen bonding interactions and *non-bonded π-interactions*. Specifically, a loss of binding affinity caused by a decrease in hydrogen bonding interactions is compensated by a gain in binding affinity acquired by an increase in aromatic ring-originated non-bonded interactions (i.e., π–π stacking interactions, CH–π interactions, cation–π interactions, etc.), and vice versa. The very existence of this inverse relationship strongly suggests that both hydrogen bonding and aromatic ring-originated non-bonded interactions are responsible for the molecular recognition of PKIs. As an illustration, two representative PKI–kinase complexes were employed to examine the relative importance of different modes of non-bonded interactions for the molecular recognition of PKIs. For this purpose, two FDA-approved PKI drugs, ibrutinib and lenvatinib, were chosen. The binding pockets of both PKIs were thoroughly examined to identify all non-bonded intermolecular interactions. Subsequently, the strengths of interaction energies between ibrutinib and its interacting residues in tyrosine kinase BTK were quantified by means of the double hybrid DFT method B2PLYP. The resulting energetics for the binding of ibrutinib in tyrosine kinase BTK showed that CH–π interactions and π–π stacking interactions between aromatic rings of the drug and hydrophobic residues in its binding pocket dominate the binding interactions. Thus, this work establishes that, in addition to hydrogen bonding, aromatic rings function as important molecular determinants for the molecular recognition of PKIs. In conclusion, our findings support the following pharmacophore model for ATP-competitive kinase inhibitors: a small molecule features a scaffold of one or more aromatic rings which is linked with one or more hydrophilic functional groups. The former has the structural role of acting as a scaffold and the functional role of participating in aromatic ring-originated non-bonded interactions with multiple hydrophobic regions in the ATP binding pocket of kinases. The latter ensure water solubility and form hydrogen bonds with the hinge region and other hydrophilic residues of the ATP binding pocket.

## 1. Introduction

Protein kinases are key enzymes in many signal transduction pathways, and play a crucial role in cellular proliferation, differentiation, and various cell regulatory processes [1,2,3,4,5]. It has been estimated that the human genome encodes 538 known protein kinase genes [6,7], which are nearly 2% of all genes. The common catalytic function of protein kinases is the covalent phosphorylation of substrate via transfer of the γ-phosphate of ATP [8,9]. Based on the phosphorylated amino acids of the substrates, the protein kinases are divided into three groups: serine/threonine kinases if they act on serine or threonine; tyrosine kinases if they act on tyrosine; and a small number of dual-specificity kinases if they act on all three [10,11].

Despite their diverse primary structure organizations, the catalytic domains of various kinases are generally conserved [5]. The kinase catalytic domain consists of two lobes: the small N-lobe is dominated by an anti-parallel β-sheet, and the large C-lobe is primarily formed of α-helices. The nucleotide, ATP, binds at the base of the cleft between the two lobes, positioning the γ-phosphate for transfer to the peptide substrate that binds to the surface of the large C-lobe.

Due to their pivotal role in signal transduction/cell cycle pathways [5], aberrant functions of protein kinases were known to cause many common diseases such as cancer, immunodeficiency, diabetes, atherosclerosis, and psoriasis [8,12,13,14,15,16]. It has been estimated that over 400 diseases are associated with protein kinases, either directly or indirectly [17]. The inhibition of aberrant protein kinases has the therapeutic potential to cure these diseases [8,18,19]. Thus, protein kinase inhibitors (PKIs) have emerged as a subject of great theoretical importance and therapeutic value [20,21]. Based on their binding modes with targeted protein kinases, small molecule PKIs can be classified into Type 1, 2, and 3 inhibitors [22,23,24,25]. A Type 1 inhibitor is defined as a small molecule that binds to the active conformation of a kinase in the ATP pocket; the Type 2 inhibitor binds to an inactive (usually DFG-OUT) conformation of a kinase; and the Type 3 inhibitor binds next to the ATP-binding pocket allosterically and is a non-ATP competitive inhibitor. In this study, the scope was limited to ATP competitive inhibitors only.

In 2001, the first protein kinase inhibitor drug, imatinib, was approved by the FDA for the treatment of chronic myeloid leukemia in the United States [26]. In the subsequent “sprouting decades of kinase inhibitors”, thousands of kinase inhibitors had been developed. As of 23 December 2020, a total of 62 FDA-approved small molecule kinase inhibitors are on the market [27]. Furthermore, over 200 kinase-targeting drugs are in different phases of clinical trials worldwide [28], and many kinase-specific inhibitors are in the preclinical stage of drug development [29]. Nevertheless, many challenges remain in kinase inhibitor drug discovery, including overcoming drug resistance, and obtaining target selectivity to reduce off-target-mediated toxicity [18,29,30,31,32]. In order to address the issue of drug selectivity, one needs to understand the molecular determinants responsible for the molecular recognition of PKIs in their respective targeted protein kinases [33]. The latter is what we aim to do in this work. Fortunately, the large number of available X-ray crystallographic structures of protein kinases with bound PKIs in the Protein Data Bank (PDB) make it possible to conduct a systematic study of protein kinase inhibitors [34].

As a first step, a large-scale data mining of the PDB [34] was performed using Pfam [35] accession numbers associated with all known kinases (see Section 4), which resulted in a database of 2139 unique PKIs bound with their target protein kinases at a high resolution (2.5 Å or better). Then, a chemoinformatic analysis of all 2139 PKIs was performed, utilizing a selective set of molecular descriptors [36] as listed in Table 1. The analysis was intended to address the question of drug-likeness of PKIs.

Several molecular descriptors, including molecular weight, number of donor atoms for hydrogen bonds, number of acceptor atoms for hydrogen bonds, cLogP, number of rotatable bonds and topological polar surface area, were chosen for our study because of their association with established roles for “drug likeness” from the perspective of bioavailability, i.e., Lipinski’s rule-of-five [37] and Veber’s rule [38]. Other molecular descriptors such as the number of aromatic rings, aromatic ratio, and fraction of sp^3^ carbon atoms, along with hydrogen bond counts, are considered to be important from the perspective of drug–protein binding affinity. As in all ligand–protein complexes, the molecular recognition between PKIs and their target protein kinases are achieved by non-bonded interactions [39,40]. Traditionally, consideration of non-bonded interactions included mainly hydrogen bonding and salt bridge interactions. However, increasingly, evidence suggests that π-moiety involved interactions, such as π–π stacking interactions [41] CH–π interactions [42], cation–π interactions [43], XH–π interactions (XH = NH, OH, SH), are equally important non-bonded interaction forces [42,44,45,46,47,48,49]. For easy reference, hereinafter, all these π-moiety involved interactions collectively will be named *non-bonded π-interactions*.

As detailed in the Results section, the chemoinformatic analysis resulted in findings that point to a potential role of aromatic rings in the molecular recognition of PKIs in their target protein kinases. As an illustration, in the second half of this paper, two representative PKI–protein complexes were employed to examine the relative importance of different modes of non-bonded interactions for the molecular recognition of PKIs in protein kinases. For this purpose, ibrutinib (an FDA-approved PKI drug) bound tyrosine–protein kinase BTK [50], and lenvatinib (also an FDA-approved PKI drug) bound vascular endothelial growth factor receptor 2 [51] were selected. The binding pockets of both PKIs were thoroughly examined to identify non-bonded interactions, such as hydrogen bonding, salt bridge interactions, π–π stacking interactions, CH–π interactions, cation–π interactions, and XH–π interactions (XH = NH, OH, SH). Subsequently, the strengths of intermolecular interaction energies between ibrutinib and its surrounding protein residues were quantified by means of the double hybrid DFT method B2PLYP [52]. The latter was identified in a benchmark study by one of the authors as the best performing DFT method for the calculation of non-bonded interactions in terms of both accuracy and computational efficiency in comparison with the highly accurate CCSD(T) method [53].

## 2. Results

### 2.1. Data Mining of PKIs

Large-scale data mining of the PDB [34] was performed (see Section 4) using Pfam [35] accession numbers PF00069, PF07714, PF00454, PF00794 and PF12330. The first two protein families were associated with conventional tyrosine protein kinases, serine/threonine protein kinases, and dual-specificity protein kinases [7], and account for a large majority of PDB entries collected here. The last three protein families are atypical protein kinases. When both redundant entries and low resolution (>2.5 Å) structures were filtered out, a total of 2139 unique PKIs bound to their associated protein kinases were obtained, which are listed in Table S1 in the Supplementary Material. Table S1 contains the following entries: Ligand name (i.e., name of PKI), Ligand ID, Name of protein kinase, PDB accession number, and Resolution for the X-ray crystal structure (in Å).

### 2.2. Chemoinformatic Analysis of PKIs

#### 2.2.1. Molecular Descriptors

**Figure 1:**
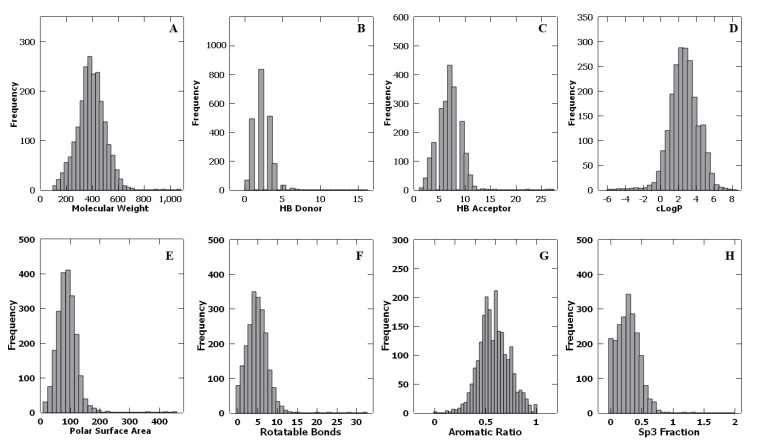
Distribution of molecular descriptors. (**A**) molecular weight (in Daltons); (**B**) number of HB donor atoms; (**C**) number of HB acceptor atoms; (**D**) calculated partition coefficient between octanol and water; (**E**) topological polar surface area (in Å^2^); (**F**) number of rotatable bonds; (**G**) aromatic ratio; and (**H**) fraction of sp^3^ carbon atoms.

For all 2139 PKIs listed in Table S1, molecular descriptors were calculated by DataWarrior 4.7 [54] and Dragon 6 [55], as described in the Section 4. Figure 1 shows the distribution of the calculated molecular descriptors in the form of histograms. Results of the statistical analysis of all calculated descriptors, including minimum, medium, maximum, and average values, are tabulated in Table 2.

The molecular descriptors studied here can be classified into two groups:

Group 1—bioavailability group—molecular weight (MW), number of donor atoms for hydrogen bonds (nHDon), number of acceptor atoms for hydrogen bonds (nHAcc), calculated partition coefficient between octanol and water (clogP), number of rotatable bonds (RBN), and topological polar surface area (TPSA).

Group 2—binding affinity group—number of aromatic rings, aromatic ratio, and fraction of sp^3^ carbon atoms. In addition, hydrogen bond counts (nHDon and nHAcc) also belong to this group.

Group 1 molecular descriptors are associated with bioavailability; therefore, established rules governing “drug likeness” should be considered. According to Lipinski’s rule-of-five (Ro5) [37], for better bioavailability, drug candidates with the following biophysical properties should be prioritized: molecular weight of less than 500 Da, calculated logarithm of the octanol−water partition coefficient (clogP) of lower than five, five or fewer hydrogen bond donors, and 10 or fewer hydrogen bond acceptors. The Veber’s rule [38] for bioavailability states that a drug candidate should have 10 or fewer rotatable bonds, fewer than 12 hydrogen bond donors or acceptors in total, and a topological polar surface area of less than 140 Å^2^.

The PKIs had an average molecular weight of 390.8 Da. Shown in Figure 1A is the distribution of molecular weights that follows a Gaussian curve. A total of 1826 PKIs had a molecular weight less than 500 Da, i.e., 85.4% of PKIs were consistent with Ro5. nHDon ranges from a minimum of zero to a maximum of 16, with an average value of 2.21. As shown in Figure 1B, PKIs with two, three, or four hydrogen bond (HB) donors account for a large majority (over 71%). There were 2122 PKIs which possessed five or fewer hydrogen bond donors, i.e., 99.2% of PKIs followed the Ro5. nHAcc has a range of 1 to 27, with an average of 6.77. Additionally, 2066 PKIs had 10 or fewer hydrogen bond acceptors, i.e., 96.6% of PKIs followed the Ro5. As far as the calculated logarithm of the octanol−water partition coefficient was concerned, 94.1% (2012) of PKIs were consistent with Ro5, i.e., with cLogP less than 5. Overall, the PKIs studied were mostly consistent with the Ro5.

The Veber’s rule was largely followed as well. As the hydrogen bond donor and acceptor counts were combined, 83.5% (1787) of PKIs satisfied Veber’s rule of possessing fewer than 12 hydrogen bond donors or acceptors in total. The topological polar surface area represents the sum of surfaces of all polar atoms in a molecule, which can be used to predict the absorption and transport properties of drugs. Molecules with a topological polar surface area that is greater than 140 Å^2^ give poor performance at permeating cell membranes. Figure 1E shows the histogram of the polar surface area. The number of PKIs with a topological polar surface area less than 140 Å^2^ was 1996, which was about 92.9% of all PKIs studied. The number of rotatable bonds ranged from a minimum of zero to a maximum of 32, with an average value of 5. As shown in Figure 1F, RBN followed a Gaussian distribution. Overall, 2069 (i.e., 96.7%) PKIs possessed 10 or fewer rotatable bonds.

The situation for aromatic rings is exactly the opposite of the above. At a first glance, one of the most striking structural features about PKIs is their “flatness”, which was well captured by three molecular descriptors in Group 2, i.e., nAR, ARR, and Fsp^3^. It is remarkable that all but one PKI possessed one or more aromatic rings. The count for the number of aromatic rings was astonishingly high, with an average of 3.04 and a maximum as high as eight. PKIs studied here, on average, had an aromatic ratio of 0.59 and a low sp^3^ carbon fraction of 0.31, respectively. In comparison, Lovering et al. reported that Fsp^3^ ranges from 0.36 in the discovery phase to 0.47 for approved drugs for compounds in GVK BIO database [56]. It is this high aromatic ring count and low fraction of sp^3^ carbon, i.e., flatness, that prompted us to undertake a study of the relationship between aromatic rings and hydrogen bonds from the perspective of molecular recognition in the next subsection.

#### 2.2.2. Relationship between Aromatic Rings and Hydrogen Bonds

We have classified PKIs based on the number of aromatic rings, which is shown in Table 3. All but one PKI contained aromatic rings. More than 88.6% of the PKIs fell into two, three, or four aromatic ring groups. There were 695 PKIs (32.5% overall) which possessed four or more aromatic rings, which were considered unfavorable for drug development according to Ritchie and Macdonald, who reported that “more than three aromatic rings in a molecule correlates with poorer compound developability and, thus, an increased risk of attrition in development” [57]. From a physicochemical property point of view, high aromatic content is undesirable because it leads to poor solubility, which was considered a negative for a compound’s druggability [56]. However, among PKIs studied here, many of them are approved by FDA as drugs and exhibit extraordinarily high counts of aromatic rings. Is there something special about protein kinases that make those PKIs work? This is what we intend to find out here.

**Figure 2:**
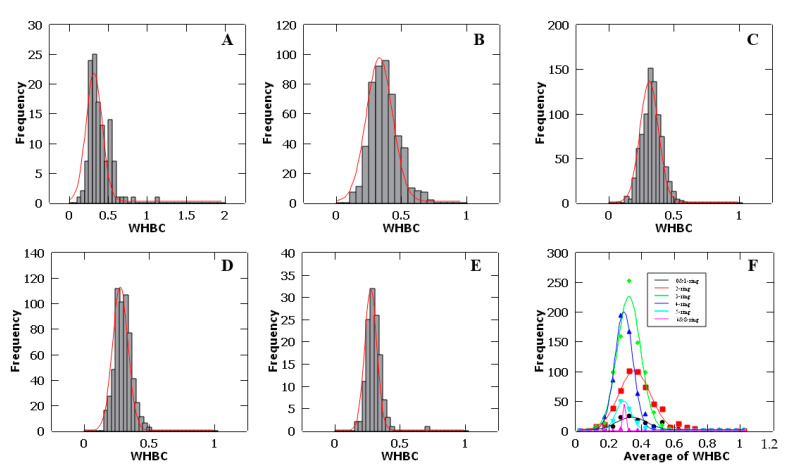
Histograms of the weighted hydrogen bond count (WHBC) for different classes of PKIs. (**A**) PKIs containing zero and one aromatic ring; (**B**) PKIs containing two aromatic rings; (**C**) PKIs containing three aromatic rings; (**D**) PKIs containing four aromatic rings; and (**E**) PKIs containing five and more aromatic rings. (**F**) Normal distribution of WHBC for different classes of PKIs.

Hydrogen bonding is widely accepted as one of the important contributors to ligand binding in proteins. To weigh the relative contribution of hydrogen bonding to binding affinity in a manner that is independent of the size of the molecule, we derived a molecular descriptor named weighted hydrogen bond count (WHBC). WHBC was obtained by dividing the combined hydrogen donor and acceptor counts with the total number of non-hydrogen atoms: (nHDon + nHAcc)/nSK. For each aromatic ring size, the detailed distributions of WHBC are displayed as histograms in Figure 2A–E. The histograms were then fit to Gaussian-shaped distribution functions and plotted in Figure 2F. It shows a clear shift to lower numbers of weighted hydrogen bond counts as the number of aromatic rings increase.

**Figure 3:**
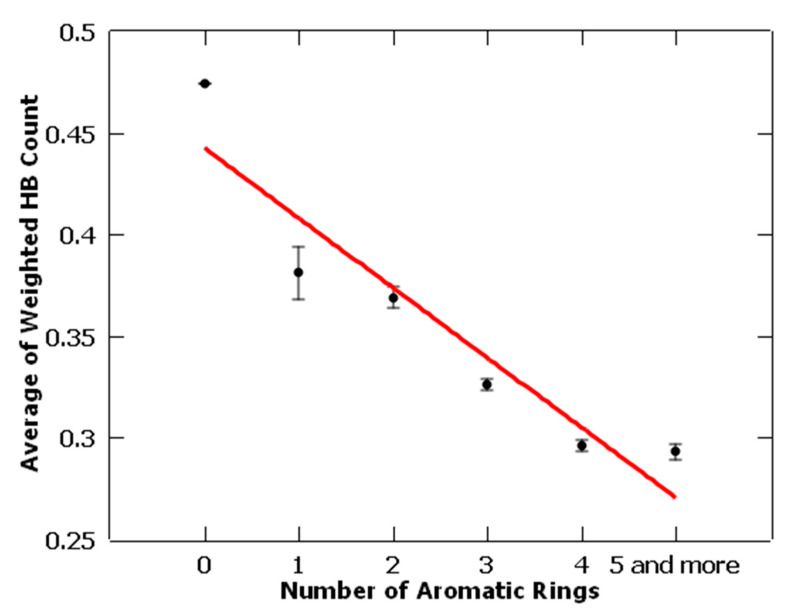
A plot of the average of WHBC versus number of aromatic rings.

For each aromatic ring size, the average value of WHBC was calculated and is listed in the last column of Table 3. In Figure 3, the average of WHBC is plotted versus the number of aromatic rings. It shows a linear relationship between the two: the average weighted hydrogen bond count is inversely proportional to the number of aromatic rings. As the number of aromatic rings increases, WHBC decreases linearly. Based on this linear relationship, we propose the exchange rule of hydrogen bonding interactions and non-bonded π-interactions. As the count of hydrogen bonds decreases, the PKI’s capacity of forming hydrogen bonds decreases. The latter resulted in a direct loss of binding energy. This loss of binding energy is compensated by the increased contribution of non-bonded π-interactions (i.e., π–π stacking interactions, CH–π interactions, cation–π interactions, and XH–π interactions (XH = NH, SH, OH)), which comes from the increase in number of aromatic rings. The very existence of this inverse relationship strongly suggests that both hydrogen bonding and non-bonded π-interactions are responsible for the molecular recognition of PKIs in their target kinases. Moreover, in addition to hydrogen bonding, the aforementioned non-bonded π-interactions may play an energetically important role in binding of PKIs to their respective target protein kinases.

### 2.3. Aromatic Rings as Determinants for Molecular Recognition of PKIs

In this subsection, modes of non-bonded intermolecular interactions and binding energetics for representative PKIs are studied.

#### 2.3.1. Modes of Non-Bonded Intermolecular Interactions

Modes of non-bonded intermolecular interactions for PKIs bound with their target proteins (as listed in Table S1) were examined systematically based on their X-ray crystal structures. The objective was to decipher the relative importance of different modes of non-bonded intermolecular interactions for the molecular recognition of PKIs in protein kinases. Due to space limitation, we have chosen two PKIs, ibrutinib and lenvatinib, as an illustration. Ibrutinib and lenvatinib are FDA-approved PKI drugs targeting the tyrosine–protein kinase BTK and vascular endothelial growth factor receptor 2, respectively.

On the basis of the 1.11 Å resolution X-ray crystal structure (PDB ID: 5P9I [50]), the binding pocket of ibrutinib in tyrosine–protein kinase BTK was thoroughly examined to identify non-bonded interactions, including hydrogen bonding, salt bridge interactions, π–π stacking interactions, cation–π interactions, CH–π interactions and XH–π interactions (XH = NH, OH, SH). The same was done for the binding pocket of lenvatinib in the vascular endothelial growth factor receptor 2 based on the 1.57 Å resolution X-ray crystal structure (PDB ID: 3WZD [51]).

**Figure 4:**
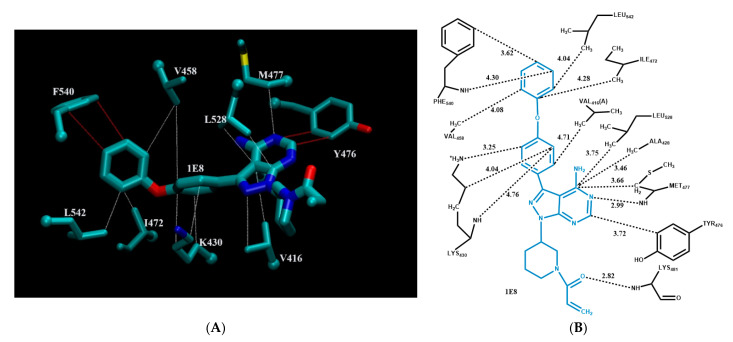
Modes of intermolecular interactions between ibrutinib and its interacting residues in tyrosine kinase BTK. (**A**) Structure of residues (in licorice representation, with the a-carbon displayed as a sphere) that are involved in π–π stacking interactions and CH–π interactions with ibrutinib (Ligand ID: 1E8) based on the 1.11 Å resolution X-ray crystal structure (PDB ID: 5P9I [50]). Dashed lines indicate the closest interatomic distance (color code: π–π stacking interactions in red, CH–π interactions in white). The structure was generated with the program VMD [59]. (**B**) A schematic intermolecular interaction map between ibrutinib and its interacting residues, with dashed lines indicating interatomic distances in Å.

Figure 4 shows the modes of intermolecular interactions between ibrutinib and its interacting residues in tyrosine kinase BTK. For clarity, Figure 4A displays only the three-dimensional structure of residues involved in π–π stacking interactions and CH–π interactions with ibrutinib. Figure 4B is a schematic intermolecular interaction map between ibrutinib and its interacting residues, with all modes of intermolecular interactions included. As can be seen from the figure, ibrutinib is interacting with its target protein BTK via hydrogen bonding, π–π stacking interactions, and CH–π, NH–π, and cation–π interactions. There are two hydrogen bonds between ibrutinib and Met477 and Lys481, with distances of 2.99 Å and 2.82 Å, respectively. Both are formed between the backbone of the protein and the PKI drug. Tyr476 is well positioned for π–π stacking interactions with ibrutinib. Phe540 can form π–π stacking interactions via the phenyl ring and NH–π interaction through the main chain amide hydrogen with the drug. There are numerous CH–π interactions between ibrutinib and its surrounding residues, including Met477, Val416, Val458, Ile472, Leu528, Ala428 and Leu542. As shown in Figure 4B, Lys430 interacts with ibrutinib via three types of non-bonded intermolecular interactions: the ε-amino group is well positioned for cation–π interactions; the main chain amide hydrogen can form a NH–π interaction; and three methylene groups can have multiple CH–π interactions with the drug [58].

**Figure 5:**
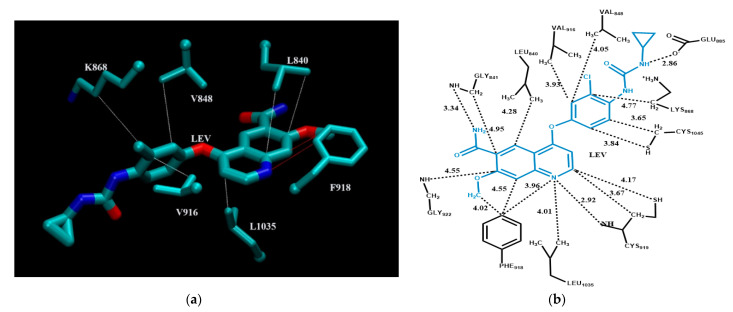
Modes of intermolecular interactions between lenvatinib and its interacting residues in vascular endothelial growth factor receptor 2. (**a**) Structure of residues (in licorice representation, with the a-carbon displayed as a sphere) that are involved in π–π stacking interactions and CH–π interactions with lenvatinib (Ligand ID: LEV) based on the 1.57 Å resolution X-ray crystal structure (PDB ID: 3WZD [51]). Dashed lines indicate the closest interatomic distance (color code: π–π stacking interactions in red, CH–π interactions in white). The structure was generated with the program VMD [59]. (**b**) A schematic intermolecular interaction map between lenvatinib and its interacting residues, with dashed lines indicating interatomic distances in Å.

Figure 5 shows modes of non-bonded intermolecular interactions between lenvatinib and its interacting residues in vascular endothelial growth factor receptor 2 [51]. For clarity, Figure 5a displays only the three-dimensional structure of residues involved in π–π stacking interactions and CH–π interactions with lenvatinib. Figure 5b is a schematic intermolecular interaction map between lenvatinib and its interacting residues, with all modes of intermolecular interactions included. As can be seen from the figure, lenvatinib is interacting with its target protein via hydrogen bonding, π–π stacking interactions, and CH–π, NH–π, and SH–π interactions. Two hydrogen bonds exist: the side chain carboxyl oxygen of Glu885 accepts a hydrogen bond from lenvatinib with a distance of 2.86 Å; the main chain amide group of Cys919 forms another hydrogen bond to lenvatinib with a distance of 2.92 Å. Phe918 is well positioned for π–π stacking interactions with lenvatinib. There are numerous CH–π interactions between lenvatinib and its surrounding residues, including Gly841, Val848, Leu840, Val916 and Leu1035. As shown in Figure 5b, both Cys919 and Cys1045 form SH–π interactions via the thiol side chain and CH–π interactions through the methylene group with lenvatinib.

In both cases, multiple modes of non-bonded interactions are involved in binding of PKIs with their binding pockets in the target proteins. In addition to hydrogen bonds, there are numerous non-bonded π-interactions, including π–π stacking interactions, CH–π interactions, cation–π interactions, and XH–π interactions (XH = NH, OH, SH).

#### 2.3.2. Strengths of Non-Bonded Intermolecular Interactions

The strengths of non-bonded intermolecular interactions between PKIs and their target protein kinases were quantified by means of quantum mechanics. Hereby, the resulting energetics for the binding of ibrutinib in tyrosine kinase BTK is presented. The strengths of intermolecular interaction energies between ibrutinib and its surrounding protein residues were quantified in a pair-wise manner by means of the double hybrid DFT method B2PLYP/def2-QZVP (see Section 4 for details). The resulting pair-wise intermolecular interaction energies between ibrutinib and surrounding residues are detailed in Table 4 and summarized on the basis of interaction modes in Table 5.

Table 4 lists the pair-wise intermolecular interaction energies between the entire ligand ibrutinib and each individual residue (first column) in its binding pocket. The second column shows the modes of intermolecular interactions as depicted in Figure 4B. The last column lists the aqueous phase interaction energy ($\Delta E_{int}^{\;aq}$), which is obtained by adding the gas phase energy ($\Delta E_{int}^{g}\;$, third column) and the energy of dehydration ($\Delta E_{Deh}^{}$, fourth column). Two general observations can be made here. First of all, as expected, the gas phase interaction energies for hydrogen bonding are strong; however, the high energetic cost of dehydration significantly offset the gas phase interaction strength. Secondly, dispersion force dominated CH–π interactions and π–π stacking interactions are relatively weak in the gas phase, although there is not much energetic cost of dehydration for the hydrophobic moieties. As a result, substantial interaction strengths remain in the aqueous phase for both CH–π interactions and π–π stacking interactions.

In Table 5, aqueous phase intermolecular interaction energies are combined based on modes of non-bonded interactions. It can be seen that CH–π interactions between aromatic rings of the drug and the aliphatic residues in its binding pocket and π–π stacking interactions between aromatic rings of the drug and the aromatic residues dominate the binding interactions between ibrutinib and tyrosine–protein kinase BTK. Cation–π interactions and NH–π interactions also make a contribution to binding. Altogether, *non-bonded π-interactions* (π–π stacking interactions, CH–π interactions, cation–π interactions, and NH–π interactions) contributed a total of −22.5 kcal/mol to the binding affinity. In contrast, two hydrogen bonds have a combined interaction energy of −0.6 kcal/mol. Although data shown here are only for one PKI–protein complex, the observation is consistent with a similar analysis of other FDA-approved PKI drugs with known drug–protein structures in Table S1 (data not shown). This quantitively affirms the dominant role played by the aromatic rings in the binding of PKIs to protein kinases.

## 3. Discussions

In this work, a high resolution non-redundant database of 2139 PKIs bound to their respective protein kinases was established through large-scale data mining of the PDB. Our choice of the PDB as a source of PKIs was largely driven by the need to analyze modes of non-bonded intermolecular interactions using three dimensional structures of PKI bound protein kinases. Furthermore, the database will be of general interest to a wide spectrum of kinase researchers working on structure-based kinase drug design and profiling analysis. The 2.5 Å resolution X-ray diffraction cut-off acts as a natural barrier to filter out PKIs with weak binding affinity, because the formation of a well-ordered ligand–protein co-crystal requires sufficiently high binding affinity [60]. The PKIs collected from PDB represent inhibitors in various phases of drug design, ranging from the discovery phase to clinical approval.

The chemoinformatic analysis of 2139 PKIs resulted in findings that PKIs are “flat” molecules with a high aromatic ring count and low fraction of sp^3^ carbon. This is consistent with a similar analysis of PKIs in early phase preclinical studies found in ChEMBL and FDA-approved PKI drugs by Bournez and co-workers [28]. More importantly, it was found here that the average weighted hydrogen bond count is inversely proportional to the number of aromatic rings. Based on this linear relationship, we put forward the exchange rule of hydrogen bonding interactions and non-bonded π-interactions. Specifically, a loss of binding affinity caused by a decrease in hydrogen bonding interactions is compensated by a gain in binding affinity acquired by an increase in non-bonded π-interactions, and vice versa. The very existence of this inverse relationship strongly suggests that both hydrogen bonding and non-bonded π-interactions are responsible for the molecular recognition of PKIs in their target kinases. This was supported by: (i) further analysis of modes of non-bonded intermolecular interactions for PKIs bound with their target proteins; and (ii) quantification of strengths of non-bonded intermolecular interactions. Thus, this work establishes that, in addition to hydrogen bonding, aromatic rings function as important molecular determinants for the molecular recognition of PKIs.

Finally, it is important to place this work in the broad context of the existing knowledge on molecular recognition of PKIs. For this purpose, a brief review on currently accepted pharmacophores for ATP competitive PKIs is warranted here. Many excellent published reviews on the subject [22,23,24] exist; therefore, only salient features will be highlighted below.

As mentioned in the Introduction section, the conserved catalytic domain of protein kinases contains the small anti-parallel β-sheet dominated N-lobe and the large α-helical C-lobe. ATP binds in a deep cleft located between the two lobes. Another important structural feature is the DFG (Asp–Phe–Gly) motif, which is a highly conserved sequence located at the beginning of the activation loop. The motif’s Asp residue is responsible for coordinating a magnesium ion which positions the phosphates of ATP for phosphotransfer. The Phe residue of the motif packs under the helix C is therefore important for the correct positioning of this helix. It is important to note that the helix C itself acts as a dynamic regulatory element for the catalytic function of protein kinases. In the active state of kinases, the DFG motif adopts a “in” conformation, with the Asp residue properly oriented toward bound ATP for transferring the γ-phosphate group to the substrate. In the inactive state of kinases, the DFG motif flips outward to adopt a “out” conformation in which the Phe residue of the DFG motif moves more than 10 Å from its position in the active kinase conformation. As a result, the Asp residue no longer coordinates the magnesium at the catalytic site, rendering the kinase inactive.

**Figure 6:**
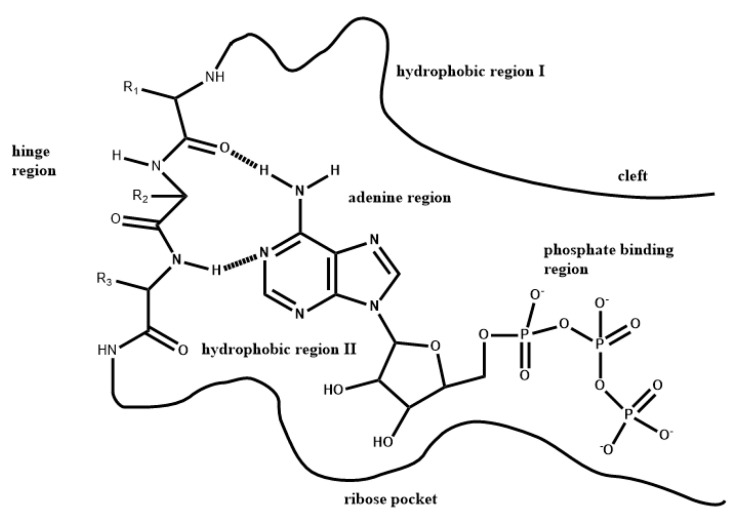
Pharmacophore model for Type 1 PKIs in the ATP binding pocket of protein kinases.

The Type 1 PKIs typically bind to the DFG-in active (open) conformation of protein kinases, mimicking the interactions of the adenine moiety of ATP [61], and forming multiple hydrogen bonds with the kinase hinge. Based on structural biology studies, the binding pocket for Type 1 PKIs can be divided into subregions: the hinge region; the adenine region; the hydrophobic region I; the hydrophobic region II; the ribose pocket; and the phosphate-binding region [22,23]. Shown in Figure 6 is a general pharmacophore model for Type 1 PKIs in the ATP binding pocket of protein kinases [24]. Both the ribose pocket and the phosphate-binding region are hydrophilic in nature. The remaining regions are generally of hydrophobic character [24]. The adenine region is mostly surrounded by hydrophobic residues. The hydrophobic region I extends deeply in the direction of the lone pair of the N7 nitrogen of adenine. The hydrophobic region II corresponds more to a hydrophobic slot opened to solvent that is not used by ATP. It is worth noting that, although both hydrophobic regions exist naturally, they are not used by ATP in most protein kinases.

In contrast, the Type 2 PKIs bind to the DFG-out inactive (closed) conformation of protein kinases. They occupy the adenosine pocket and still have contact with the hinge. The transition from the “DFG-in” to the “DFG-out” conformation exposes a third hydrophobic pocket adjacent to the ATP pocket, named hydrophobic region III. The latter is also commonly referred to as an “allosteric site” and is utilized by Type 2 PKIs to lock the kinase in the inactive conformation [7,22].

With the above background knowledge, we are now positioned to discuss the broad implications of our findings that, in addition to hydrogen bonding, aromatic rings of PKIs function as important molecular determinants for inhibitor binding. From the perspective of aromatic rings, hydrophobic regions I, II and III, plus the adenine region, are the most relevant. Those four regions are loaded with hydrophobic residues that can participate in non-bonded π-interactions (i.e., π–π stacking interactions, CH–π interactions, and XH–π interactions (XH = NH, SH, OH)). The aliphatic residues Gly, Ala, Val, Leu, and Ile in the four hydrophobic regions can form CH–π interactions with aromatic rings of PKIs. Additionally, aromatic residues Phe, Tyr and Trp can form π–π stacking interactions with aromatic rings of PKIs. As shown in the two illustrative cases, even the middle methylene groups of polar residues and charged residues can participate in CH–π interactions with aromatic rings. In addition, the side chains of Ser, Thr, Asn, Gln, Cys and Met residues can form OH–π, NH–π, and SH–π interactions, respectively, with aromatic rings of drugs.

As a general principle, from the perspective of binding energetics, the importance of CH–π interactions and π–π stacking interactions in molecular recognition must not be underestimated. As demonstrated in Table 4, although the dispersion forces dominating CH–π interactions and π–π stacking interactions are relatively weak in the gas phase in comparison with hydrogen bonding interactions, there is a much lower (or zero) energetic cost of dehydration involved. As a result, significant interaction strengths remain in the aqueous phase for both CH–π interactions and π–π stacking interactions.

In conclusion, our findings support the following pharmacophore model for ATP-competitive kinase inhibitors: a small molecule features a scaffold of one or more aromatic rings that is linked with one or more hydrophilic functional groups. The former has the structural role of acting as a scaffold and the functional role of participating in non-bonded π-interactions (i.e., π–π stacking interactions, CH–π interactions, cation–π interactions, and XH–π interactions (XH = NH, SH, OH)) with hydrophobic regions I, II, and III, and the adenine region. The latter ensure water solubility and form hydrogen bonds with the hinge region and other hydrophilic residues of the ATP binding pocket. It is our expectation that this pharmacophore has the promise to profoundly impact lead optimization in protein kinase targeted drug discovery.

## 4. Theory and Methods

### 4.1. Data Mining of PKIs

A large-scale data mining of the PDB was carried out to establish a high resolution non-redundant database of PKIs bound to their respective protein kinases according to the following protocol:

- The 28 March 2018 release of the PDB was searched for Pfam [35] accession numbers PF00069, PF07714, PF00454, PF00794 and PF12330, resulting in a total of 4884 entries that contained protein kinases;
- Only high-resolution (2.5 Å or better) X-ray crystal structures of protein kinases complexed with bound PKIs were retained for further analysis. The reason for the cut-off is two-fold: (i) to ensure quality of the structures, and (ii) to ensure that selected PKIs have a sufficiently high binding affinity to kinase because the formation of a well-ordered ligand-protein co-crystal that is good enough for 2.5 Å resolution requires a reasonably high binding affinity [60];
- Multiple protein kinases bound with the same PKI were filtered out to retain one protein kinase only for each PKI.

### 4.2. Chemoinformatic Analysis: Molecular Descriptors

Two widely used cheminformatics programs, DataWarrior 4.7 [54] and Dragon 6 [55], were employed to calculate the following molecular descriptors for all 2139 PKIs: Molecular weight (MW), Number of donor atoms for HB (nHDon), Number of acceptor atoms for HB (nHAcc), Number of aromatic rings (nAR), Aromatic ratio (ARR), Number of non-H atoms (nSK), Number of sp^3^ hybridized C atoms (Nsp3), Number of rotatable bonds (RBN), Calculated partition coefficient between octanol and water (cLogP), Total surface area (SA) and Topological polar surface area (TPSA). The last two molecular descriptors required input of three-dimensional geometries of PKIs that were gathered from the PDB.

We have also derived several secondary molecular descriptors based on the primary molecular descriptors. The fraction of sp^3^ carbon (Fsp^3^) was obtained by dividing Nsp3 by the total number of carbon atoms [56]. The weighted hydrogen bond count (WHBC) was calculated by the formula (nHDon + nHAcc)/nSK.

### 4.3. Quantum Chemical Calculation of Intermolecular Interaction Energies

The conceptual framework for the protein–protein complex formation in solution is depicted in the following scheme.

(1) $$A(\mathrm{aq})\;+\;B(\mathrm{aq})\;\overset{\Delta E_{int\;}^{aq}}{\to }\;\;\;\;\mathrm{AB}\left(\mathrm{aq}\right)\;\;\Delta G_{A}^{sol}↑\;\;\;\;\;\;\;\;\;\;\;\;\;\Delta G_{B}^{sol}↑\;\;\;\;\;\;\;\;\;\;\;\;\;\;\;↑\Delta G_{AB}^{sol}\;A(g)\;+\;B(g)\;\underset{\Delta E_{int\;}^{g}}{\to }\;\mathrm{AB}\left(g\right)$$

This served as a basis for our analysis of the binding affinity of PKIs in protein kinases. It is worth noting that a similar scheme was used to calculate solution phase binding affinities for ligand–protein complexes previously [58,62]. Both ligand and proteins were solvated before complex formation. They both lose part of their solvation shell upon binding, which incurs an energy cost commonly referred to as dehydration energy. According to the scheme, the binding energy for complex formation in solution $\Delta E_{int\;}^{aq}$ can be evaluated indirectly by calculating intermolecular interaction energies in the gas phase, $\Delta E_{int\;}^{g}$, followed by a correction for the dehydration energy $\Delta E_{Deh}^{}$:

(2) $$\Delta E_{int\;}^{aq}=\;\Delta E_{int\;}^{g}+\;\Delta E_{Deh}^{}\;$$

The gas phase intermolecular interaction energies between the PKI and its surrounding residues in protein kinases, $\Delta E_{int\;}^{g}$, were calculated by means of the supermolecular approach. In the supermolecular approach, the energy of interaction between molecules *A* and *B* is defined as the difference between the energy of the interacting dimer and the energies of the monomers:

(3) $$\Delta E_{int\;}^{g}=\;E_{AB}^{g}-E_{A}^{g}-\;E_{B}^{g}$$

The calculations were carried out using the ORCA 4.0 program [63] by means of the double-hybrid density functional method B2PLYP [52] using the def2-QZVP basis set [64]. Grimme’s D3BJ dispersion correction [65] was applied for the proper account of dispersion interactions. For efficiency, B2PLYP was implemented with the resolution of identity (RI) approximation for the perturbation step and RIJK [66] for the SCF step. In RIJK, appropriate auxiliary basis sets are used to substitute both Coulomb (J) and exchange integrals as used in the Kohn–Sham/Fock matrix by auxiliary three-center and two-center electron repulsion integrals. The choice of the double hybrid density functional method B2PLYP coupled with the def2-QZVP basis set is based on a systematic benchmark study conducted by one of the authors [53]. It was found that the double-hybrid RIJK RI-B2PLYP implementation is the best DFT method for the treatment of non-bonded interactions in terms of both accuracy and computational efficiency in comparison with the highly accurate CCSD(T) method [53]. The basis set superposition error (BSSE) was corrected by the Boys and Bernardi Counterpoise Method [67].

The dehydration energy for the complex formation is defined (see Equation (1)) by

(4) $$\Delta E_{Deh\;}^{}=\;\Delta G_{AB}^{sol}-\Delta G_{A}^{sol}-\;\Delta G_{B}^{sol}$$

where $\Delta G_{i}^{sol}$, *i* = *AB*, *A*, *B* represents the free energies of solvation for the complex *AB*, and the monomers *A*, *B*, respectively. Due to the prohibitively high cost of explicitly including solvent molecules in simulating biological systems, a common way to calculate the free energy of solvation is through continuum models. Here, we adopted the SM5.42R Solvation Model of Cramer and Truhlar [43], as implemented in the 2008 R1 version of GAMESS [68] for evaluation of the free energy of solvation.

## Figures and Tables

**Table 1:** List of studied molecular descriptors.

Name	Description
MW	Molecular weight
nHDon	Number of donor atoms for hydrogen bond (HB)
nHAcc	Number of acceptor atoms for HB
SA	Total surface area
TPSA	Topological polar surface area
nSK	Number of non-H atoms
nsp3	Number of sp^3^ hybridized carbon atoms
RBN	Number of rotatable bonds
ARR	Aromatic ratio
cLogP	Calculated partition coefficient between octanol and water
nAR	Number of aromatic rings
Fsp^3^	Fraction of sp^3^ carbon atoms

**Table 2:** Values of molecular descriptors.

Molecular Descriptor	Min	Median	Max	Average	Ro5 ^a^	Veber ^b^
MW (Da)	94.12	390.29	1057.11	390.76 ± 2.28	85.4%	-
nHDon	0	2	16	2.21 ± 0.03	99.2%	83.5% ^c^
nHAcc	1	7	27	6.77 ± 0.05	96.6%	
cLogP	−5.95	2.58	7.94	2.60 ± 0.03	94.1%	-
SA (Å^2^)	77.39	289.53	761.02	289.47 ± 1.69	-	-
TPSA (Å^2^)	16.61	94.06	457.72	95.64 ± 0.72	-	92.9%
RBN	0	5	32	4.77 ± 0.06	-	96.7%
ARR	0	0.58	1	0.59 ± 0.00	-	-
nAR	0	3	8	3.04 ± 0.02	-	-
Nsp3	0	6	31	6.38 ± 0.09	-	-
nSK	7	28	74	27.81 ± 0.17	-	-
Fsp^3^	0	0.30	1.36	0.31 ± 0.00	-	-

^a^ Percentage of molecular descriptors that obey the Lipinski’s rule of five (Ro5); ^b^ Percentage of molecular descriptors that obey Veber’s rule. ^c^ Based on combined count of hydrogen bond donors and acceptors.

**Table 3:** Distribution and the average value of the weighted hydrogen bond count (WHBC) for each class of protein kinase inhibitor (PKI).

Number of Aromatic Rings	Number of PKIs	Percentage (%)	Average of WHBC
0	1	0.05	0.474 ± 0
1	117	5.47	0.381 ± 0.013
2	507	23.70	0.369 ± 0.005
3	819	38.29	0.326 ± 0.003
4	570	26.65	0.296 ± 0.003
5 and more	125	5.84	0.293 ± 0.004

**Table 4:** Intermolecular interaction energies between ibrutinib and its interacting residues from tyrosine kinase BTK.

Residue	Interaction Mode	$\Delta E_{int}^{g}\;(\mathrm{kcal}/\mathrm{mol})$	$\Delta E_{Deh}^{}\;(\mathrm{kcal}/\mathrm{mol})$	$\Delta E_{int}^{\;aq}\;(\mathrm{kcal}/\mathrm{mol})$
Met477	H-bond, CH–π	−4.1	3.8	−0.3
Lys481	H-bond	−3.6	3.3	−0.3
Tyr476	π–π, CH–π	−4.0	2.5	−1.5
Phe540	π–π, NH–π	−2.8	0.4	−2.4
Lys430	cation–π, NH–π, CH–π	−12.8	5.4	−7.3
Val416	CH–π	−3.0	0.1	−2.9
Ala428	CH–π	−1.5	-0.1	−1.6
Val458	CH–π	−1.2	-0.1	−1.3
Ile472	CH–π	−1.4	0.0	−1.4
Leu528	CH–π	−3.9	1.2	−2.7
Leu542	CH–π	−1.4	0.0	−1.4

**Table 5:** Contributions of the different modes of intermolecular interactions to the binding affinity between ibrutinib and its interacting residues in tyrosine kinase BTK.

Table	Residue	Interaction Mode	$\Delta E_{int}^{\;aq}\;(\mathrm{kcal}/\mathrm{mol})$	Combined Energy (kcal/mol)
Hydrogen Bonding	Met477	H-bond, CH–π	−0.3	−0.6
	Lys481	H-bond	−0.3	
non-bonded π-interactions	Tyr476	π–π, CH–π	−1.5	−22.5
	Phe540	π–π, NH–π	−2.4	
	Lys430	cation–π, NH–π, CH–π	−7.3	
	Val416	CH–π	−2.9	
	Ala428	CH–π	−1.6	
	Val458	CH–π	−1.3	
	Ile472	CH–π	−1.4	
	Leu528	CH–π	−2.7	
	Leu542	CH–π	−1.4	

## Data Availability

The database of 2139 PKIs resulting from data mining of the PDB is provided in Supplementary Materials.

## Supplementary Materials

The following are available online. Table S1: List of 2139 PKIs in association with their respective binding protein kinases.
