# Supplementary material for: Aromatic Rings as Molecular Determinants for the Molecular Recognition of Protein Kinase Inhibitors
Source: Molecules. 2021 Mar 22;26(6):1776. doi: 10.3390/molecules26061776 (PMC8005117; doi:10.3390/molecules26061776)
Supplement: Supplementary file 1 [file molecules-26-01776-s001.pdf]

Table S1: List of 2139 PKIs in association with their respective binding protein kinases

PDB ID	Resolution	Kinase Name	Ligand ID	Ligand Name
1AGW	2.4	FGF receptor 1	SU2	3-[4-(1-FORMYLPIPERAZIN-4-YL)-BENZYLIDENYL]-2-INDOLINONE
1BL6	2.5	MAP Kinase P38	SB6	4-(4-FLUOROPHENYL)-1-CYCLOROPROPYLMETHYL-5-(4-PYRIDYL)-IMIDAZOLE
1BMK	2.4	MAP Kinase P38	SB5	4-(FLUOROPHENYL)-1-CYCLOPROPYLMETHYL-5-(2-AMINO-4-PYRIMIDINYL)IMIDAZOLE
1BX6	2.1	cAMP-dependent protein kinase	BA1	BALANOL
1CKP	2.05	Cyclin-dependent kinase 2	PVB	PURVALANOL B
1DI8	2.2	Cyclin-dependent kinase 2	DTQ	4-[3-HYDROXYANILINO]-6,7-DIMETHOXYQUINAZOLINE
1DM2	2.1	Cyclin-dependent kinase 2	HMD	4-(5-AMINO-4-OXO-4H-PYRAZOL-3-YL)-2-BROMO-4,5,6,7-TETRAHYDRO-3AH-PYRROLO[2,3-C]AZEPIN-8-ONE
1E1V	1.95	Cyclin-dependent kinase 2	CMG	6-O-CYCLOHEXYLMETHYL GUANINE
1E1X	1.85	Cyclin-dependent kinase 2	NW1	6-CYCLOHEXYLMETHYLOXY-5-NITROSO-PYRIMIDINE-2,4-DIAMINE
1E9H	2.5	Cyclin-dependent kinase 2	INR	2',3-DIOXO-1,1',2',3-TETRAHYDRO-2,3'-BIINDOLE-5'-SULFONIC ACID
1FGI	2.5	FGF receptor 1	SU1	3-[(3-(2-CARBOXYETHYL)-4-METHYLPYRROL-2-YL)METHYLENE]-2-INDOLINONE
1FPU	2.4	Proto-oncogene tyrosine-protein kinase ABL	PRC	N-[4-METHYL-3-[[4-(3-PYRIDINYL)-2-PYRIMIDINYL]AMINO]PHENYL]-3-PYRIDINECARBOXAMIDE
1FVT	2.2	Cyclin-dependent kinase 2	106	4-[(2Z)-2-(5-bromo-2-oxo-1,2-dihydro-3H-indol-3-ylidene)hydrazinyl]benzene-1-sulfonamide
1GII	2	Cyclin-dependent kinase 2	1PU	1-(5-OXO-2,3,5,9B-TETRAHYDRO-1H-PYRROLO[2,1-A]ISOINDOL-9-YL)-3-PYRIDIN-2-YL-UREA
1GIJ	2.2	Cyclin-dependent kinase 2	2PU	1-(5-OXO-2,3,5,9B-TETRAHYDRO-1H-PYRROLO[2,1-A]ISOINDOL-9-YL)-3-(5-PYRROLIDIN-2-YL-1H-PYRAZOL-3-YL)-UREA
1GZ8	1.3	Cyclin-dependent kinase 2	MBP	1-[(2-AMINO-6,9-DIHYDRO-1H-PURIN-6-YL)OXY]-3-METHYL-2-BUTANOL
1H00	1.6	Cyclin-dependent kinase 2	FAP	(2S)-1-[4-({6-[(2,6-DIFLUOROPHENYL)AMINO]PYRIMIDIN-4-YL}AMINO)PHENOXY]-3-(DIMETHYLAMINO)PROPAN-2-OL
1H01	1.79	Cyclin-dependent kinase 2	FAL	(2R)-1-[4-({4-[(2,5-DICHLOROPHENYL)AMINO]PYRIMIDIN-2-YL}AMINO)PHENOXY]-3-(DIMETHYLAMINO)PROPAN-2-OL
1H07	1.85	Cyclin-dependent kinase 2	MFP	((2-BROMO-4-METHYLPHENYL){6-[(4-{[(2R)-3-(DIMETHYLAMINO)-2-HYDROXYPROPYL]OXY}PHENYL)AMINO]PYRIMIDIN-4-YL}AMINO)ACETONITRILE
1H08	1.8	Cyclin-dependent kinase 2	BYP	(2R)-1-{4-[(4-ANILINO-5-BROMOPYRIMIDIN-2-YL)AMINO]PHENOXY}-3-(DIMETHYLAMINO)PROPAN-2-OL
1H0V	1.9	Cyclin-dependent kinase 2	UN4	5-{[(2-AMINO-9H-PURIN-6-YL)OXY]METHYL}-2-PYRROLIDINONE
1H0W	2.1	Cyclin-dependent kinase 2	207	1-AMINO-6-CYCLOHEX-3-ENYLMETHYLOXYPURINE
1H1Q	2.5	Cyclin-dependent kinase 2	2A6	2-ANILINO-6-CYCLOHEXYLMETHOXYPURINE
1H1R	2	Cyclin-dependent kinase 2	6CP	6-CYCLOHEXYLMETHOXY-2-(3'-CHLOROANILINO) PURINE
1IAN	2	MAP kinase P38	D13	4-[5-(3-IODO-PHENYL)-2-(4-METHANESULFINYL-PHENYL)-1H-IMIDAZOL-4-YL]-PYRIDINE
1JSV	1.96	Cyclin-dependent kinase 2	U55	4-[(6-AMINO-4-PYRIMIDINYL)AMINO]BENZENESULFONAMIDE
1JVP	1.53	Cyclin-dependent kinase 2	LIG	3-PYRIDIN-4-YL-2,4-DIHYDRO-INDENO[1,2-.C.]PYRAZOLE
1KE5	2.2	Cyclin-dependent kinase 2	LS1	N-METHYL-4-{[(2-OXO-1,2-DIHYDRO-3H-INDOL-3-YLIDENE)METHYL]AMINO}BENZENESULFONAMIDE
1KE6	2	Cyclin-dependent kinase 2	LS2	N-METHYL-{4-[2-(7-OXO-6,7-DIHYDRO-8H-[1,3]THIAZOLO[5,4-E]INDOL-8-YLIDENE)HYDRAZINO]PHENYL}METHANESULFONAMIDE
1KE7	2	Cyclin-dependent kinase 2	LS3	3-{[(2,2-DIOXIDO-1,3-DIHYDRO-2-BENZOTHIEN-5-YL)AMINO]METHYLENE}-5-(1,3-OXAZOL-5-YL)-1,3-DIHYDRO-2H-INDOL-2-ONE
1KE8	2	Cyclin-dependent kinase 2	LS4	4-{[(2-OXO-1,2-DIHYDRO-3H-INDOL-3-YLIDENE)METHYL]AMINO}-N-(1,3-THIAZOL-2-YL)BENZENESULFONAMIDE
1M2P	2	Casein kinase II	HNA	1,8-DI-HYDROXY-4-NITRO-ANTHRAQUINONE
1M2Q	1.79	Casein kinase II	MNX	1,8-DI-HYDROXY-4-NITRO-XANTHEN-9-ONE
1M2R	1.7	Casein kinase II	MNY	5,8-DI-AMINO-1,4-DIHYDROXY-ANTHRAQUINONE
1M7Q	2.4	MAP kinase 14	DQO	1-(2,6-DICHLOROPHENYL)-5-(2,4-DIFLUOROPHENYL)-7-PIPERAZIN-1-YL-3,4-DIHYDROQUINAZOLIN-2(1H)-ONE
1NVQ	2	Serine/threonine-protein kinase Chk1	UCN	7-HYDROXYSTAUROSPORINE
1NVS	1.8	Serine/threonine-protein kinase Chk1	UCM	REL-(9R,12S)-9,10,11,12-TETRAHYDRO-9,12-EPOXY-1H-DIINDOLO[1,2,3-FG:3',2',1'-KL]PYRROLO[3,4-I][1,6]BENZODIAZOCINE-1,3(2H)-DIONE
1O9U	2.4	Glycogen synthase kinase-3 beta	ADZ	9-METHYL-9H-PURIN-6-AMINE
1OEC	2.4	Fibroblast growth factor receptor 2	AA2	4-ARYL-2-PHENYLAMINO PYRIMIDINE
1OI9	2.1	Cyclin-dependent kinase 2	N20	6-CYCLOHEXYLMETHYLOXY-2-(4'-HYDROXYANILINO)PURINE
1OIQ	2.31	Cyclin-dependent kinase 2	HDU	N-[4-(2-METHYLIMIDAZO[1,2-A]PYRIDIN-3-YL)-2-PYRIMIDINYL]ACETAMIDE
1OIR	1.91	Cyclin-dependent kinase 2	HDY	1-(DIMETHYLAMINO)-3-(4-{{4-(2-METHYLIMIDAZO[1,2-A]PYRIDIN-3-YL)PYRIMIDIN-2-YL]AMINO}PHENOXY)PROPAN-2-OL
1OIT	1.6	Cyclin-dependent kinase 2	HDT	4-[(4-IMIDAZO[1,2-A]PYRIDIN-3-YLPYRIMIDIN-2-YL)AMINO]BENZENESULFONAMIDE
1OIU	2	Cyclin-dependent kinase 2	N76	3-(6-CYCLOHEXYLMETHOXY-9H-PURIN-2-YLAMINO)-BENZENESULFONAMIDE
1OIY	2.4	Cyclin-dependent kinase 2	N41	4-(6-CYCLOHEXYLMETHOXY-9H-PURIN-2-YLAMINO)--BENZAMIDE
1OM1	1.68	Casein kinase II	IQA	(5-OXO-5,6-DIHYDRO-INDOLO[1,2-A]QUINAZOLIN-7-YL)-ACETIC ACID
1OPK	1.8	Proto-oncogene tyrosine-protein kinase ABL1	P16	6-(2,6-DICHLOROPHENYL)-2-{[3-(HYDROXYMETHYL)PHENYL]AMINO}-8-METHYLPYRIDO[2,3-D]PYRIMIDIN-7(8H)-ONE
1OUK	2.5	MAP kinase 14	084	4-[5-[2-(1-PHENYL-ETHYLAMINO)-PYRIMIDIN-4-YL]-1-METHYL-4-(3-TRIFLUOROMETHYLPHENYL)-1H-IMIDAZOL-2-YL]-PIPERIDINE
1OUY	2.5	MAP kinase 14	094	1-(2,6-DICHLOROPHENYL)-6-[(2,4-DIFLUOROPHENYL)SULFANYL]-7-(1,2,3,6-TETRAHYDRO-4-PYRIDINYL)-3,4-DIHYDROPYRIDO[3,2-D]PYRIMIDIN-2(1H)-ONE
1OVE	2.1	MAP kinase 14	358	1-(2,6-DICHLOROPHENYL)-5-(2,4-DIFLUOROPHENYL)-7-PIPERIDIN-4-YL-3,4-DIHYDROQUINOLIN-2(1H)-ONE

1OZ1	2.1	MAP kinase 14	FPH	3-(4-FLUOROPHENYL)-2-PYRIDIN-4-YL-1H-PYRROLO[3,2-B]PYRIDIN-1-OL
1P2A	2.5	Cyclin-dependent kinase 2	5BN	5-[(2-AMINOETHYL)AMINO]-6-FLUORO-3-(1H-PYRROL-2-YL)BENZO[CD]INDOL-2(1H)-ONE
1P4F	1.9	Death-associated protein kinase 1	DRG	5,6-DIHYDRO-BENZO[H]CINNOLIN-3-YLAMINE
1P5E	2.22	Cyclin-dependent kinase 2	TBS	4,5,6,7-TETRABROMOBENZOTRIAZOLE
1PMN	2.2	MAP kinase 10	984	CYCLOPROPYL-{4-[5-(3,4-DICHLOROPHENYL)-2-[(1-METHYL)-PIPERIDIN]-4-YL-3-PROPYL-3H-IMIDAZOL-4-YL]-PYRIMIDIN-2-YL}AMINE
1PXI	1.95	Cyclin-dependent kinase 2	CK1	4-(2,5-DICHLOROTHIEN-3-YL)PYRIMIDIN-2-AMINE
1PXL	2.5	Cyclin-dependent kinase 2	CK4	4-(2,4-DIMETHYL-1,3-THIAZOL-5-YL)-N-[4-(TRIFLUOROMETHYL)PHENYL]PYRIMIDIN-2-AMINE
1PXN	2.5	Cyclin-dependent kinase 2	CK6	4-[4-(4-METHYL-2-METHYLAMINO-THIAZOL-5-YL)-PYRIMIDIN-2-YLAMINO]-PHENOL
1PXO	1.96	Cyclin-dependent kinase 2	CK7	[4-(2-AMINO-4-METHYL-THIAZOL-5-YL)-PYRIMIDIN-2-YL]-(3-NITRO-PHENYL)-AMINE
1PY5	2.3	TGF-beta receptor type I	PY1	4-(3-PYRIDIN-2-YL-1H-PYRAZOL-4-YL)QUINOLINE
1PYE	2	Cyclin-dependent kinase 2	PM1	[2-AMINO-6-(2,6-DIFLUORO-BENZOYL)-IMIDAZO[1,2-A]PYRIDIN-3-YL]-PHENYL-METHANONE
1Q3W	2.3	Glycogen synthase kinase-3 beta	ATU	9-NITRO-5,12-DIHYDRO-7H-BENZO[2,3]AZEPINO[4,5-B]INDOL-6-ONE
1Q41	2.1	Glycogen synthase kinase-3 beta	IXM	(Z)-1H,1'H-[2,3']BIINDOLYLIDENE-3,2'-DIONE-3-OXIME
1Q5K	1.94	Glycogen synthase kinase-3 beta	TMU	N-(4-METHOXYBENZYL)-N'-(5-NITRO-1,3-THIAZOL-2-YL)UREA
1Q8T	2	cAMP-dependent protein kinase	Y27	(R)-TRANS-4-(1-AMINOETHYL)-N-(4-PYRIDYL) CYCLOHEXANECARBOXAMIDE
1Q8U	1.9	cAMP-dependent protein kinase	H52	(S)-2-METHYL-1-[(4-METHYL-5-ISOQUINOLINE)SULFONYL]-HOMOPIPERAZINE
1QCF	2	Haematopoetic cell kinase (HCK)	PP1	1-TER-BUTYL-3-P-TOLYL-1H-PYRAZOLO[3,4-D]PYRIMIDIN-4-YLAMINE
1R0E	2.25	Glycogen synthase kinase-3 beta	DFN	3-[3-(2,3-DIHYDROXY-PROPYLAMINO)-PHENYL]-4-(5-FLUORO-1-METHYL-1H-INDOL-3-YL)-PYRROLE-2,5-DIONE
1R0P	1.8	Hepatocyte growth factor receptor	KSA	K-252A
1R78	2	Cyclin-dependent kinase 2	FMD	4-((3R,4S,5R)-4-AMINO-3,5-DIHYDROXY-HEX-1-YNYL)-5-FLUORO-3-[1-(3-METHOXY-1H-PYRROL-2-YL)-METH-(Z)-YLIDENE]-1,3-DIHYDRO-INDOL-2-ONE
1RE8	2.1	cAMP-dependent protein kinase	BD2	3-[(4-HYDROXYBENZOYL)AMINO]AZEPAN-4-YL 4-(2-HYDROXYBENZOYL)BENZOATE
1REJ	2.2	cAMP-dependent protein kinase	B1L	3-[(4-HYDROXYBENZOYL)AMINO]AZEPAN-4-YL 4-HYDROXYBENZOATE
1REK	2.3	cAMP-dependent protein kinase	B8L	3-[(3-SEC-BUTYL-4-HYDROXYBENZOYL)AMINO]AZEPAN-4-YL 4-(2-HYDROXY-5-METHOXYBENZOYL)BENZOATE
1RW8	2.4	TGF-beta receptor type I	580	3-(4-FLUOROPHENYL)-2-(6-METHYLPYRIDIN-2-YL)-5,6-DIHYDRO-4H-PYRROLO[1,2-B]PYRAZOLE
1SVE	2.49	cAMP-dependent protein kinase	I01	(4R)-4-(2-FLUORO-6-HYDROXY-3-METHOXY-BENZOYL)-BENZOIC ACID (3R)-3-[(PYRIDINE-4-CARBONYL)AMINO]-AZEPAN-4-YL ESTER
1SVG	2.02	cAMP-dependent protein kinase	I04	N-{(3R,4R)-4-[4-(2-FLUORO-6-HYDROXY-3-METHOXY-BENZOYL)-BENZOYLAMINO]-AZEPAN-3-YL}ISONICOTINAMIDE
1SVH	2.3	cAMP-dependent protein kinase	I08	(3R,4S)-N-(4-{TRANS-2-[4-(2-FLUORO-6-HYDROXY-3-METHOXY-BENZOYL)-PHENYL]-VINYL}-AZEPAN-3-YL)-ISONICOTINAMIDE
1SZM	2.5	cAMP-dependent protein kinase	BI4	3-(1H-INDOL-3-YL)-4-{1-[2-(1-METHYLPYRROLIDIN-2-YL)ETHYL]-1H-INDOL-3-YL}-1H-PYRROLE-2,5-DIONE
1UNG	2.3	Cyclin-dependent kinase 5	ALH	6-PHENYL[5H]PYRROLO[2,3-B]PYRAZINE
1URW	1.6	Cyclin-dependent kinase 2	I1P	2-[4-(N-(3-DIMETHYLAMINOPROPYL)SULPHAMOYL)ANILINO]-
1UU7	1.9	3-phosphoinositide dependent protein kinase-1	BI2	3-(1H-INDOL-3-YL)-4-(1-{2-[(2S)-1-METHYLPYRROLIDINYL]ETHYL}-1H-INDOL-3-YL)-1H-PYRROLE-2,5-DIONE
1UU9	1.95	3-phosphoinositide dependent protein kinase-1	BI3	3-[1-(3-AMINOPROPYL)-1H-INDOL-3-YL]-4-(1H-INDOL-3-YL)-1H-PYRROLE-2,5-DIONE
1V1K	2.31	Cyclin-dependent kinase 2	3FP	(2R)-1-(DIMETHYLAMINO)-3-{4-[(6-{[2-FLUORO-5-(TRIFLUOROMETHYL)PHENYL]AMINO}PYRIMIDIN-4-YL)AMINO]PHENOXY}PROPAN-2-OL
1VJY	2	TGF-beta receptor type I	460	2-[5-(6-METHYLPYRIDIN-2-YL)-2,3-DIHYDRO-1H-PYRAZOL-4-YL]-1,5-NAPHTHYRIDINE
1VYW	2.3	Cyclin-dependent kinase 2	292	N-(3-CYCLOPROPYL-1H-PYRAZOL-5-YL)-2-(2-NAPHTHYL)ACETAMIDE
1VYZ	2.21	Cyclin-dependent kinase 2	N5B	N-(5-CYCLOPROPYL-1H-PYRAZOL-3-YL)BENZAMIDE
1W7H	2.214	MAP kinase 14	3IP	3-(BENZYLOXY)PYRIDIN-2-AMINE
1W82	2.2	MAP kinase 14	L10	N-[(3Z)-5-TERT-BUTYL-2-PHENYL-1,2-DIHYDRO-3H-PYRAZOL-3-YLIDENE]-N'-(4-CHLOROPHENYL)UREA
1W83	2.5	MAP kinase 14	L11	N-[4-CHLORO-3-(PYRIDIN-3-YLOXYMETHYL)-PHENYL]-3-FLUORO-
1W84	2.2	MAP kinase 14	L12	3-(2-PYRIDIN-4-YLETHYL)-1H-INDOLE
1W8C	2.05	Cyclin-dependent kinase 2	N69	6-(CYCLOHEXYLMETHOXY)-8-ISOPROPYL-9H-PURIN-2-AMINE
1WBN	2.4	MAP kinase 14	L09	N-(3-TERT-BUTYL-1H-PYRAZOL-5-YL)-N'-{4-CHLORO-3-[(PYRIDIN-3-YLOXY)METHYL]PHENYL}UREA
1WBO	2.16	MAP kinase 14	2CH	2-CHLOROPHENOL
1WBS	1.8	MAP kinase 14	LI2	3-FLUORO-5-MORPHOLIN-4-YL-N-[3-(2-PYRIDIN-4-YLETHYL)-1H-INDOL-5-YL]BENZAMIDE
1WBT	2	MAP kinase 14	WBT	3-FLUORO-5-MORPHOLIN-4-YL-N-[1-(2-PYRIDIN-4-YLETHYL)-1H-INDOL-6-YL]BENZAMIDE
1WBV	2	MAP kinase 14	LI3	3-FLUORO-N-1H-INDOL-5-YL-5-MORPHOLIN-4-YLBENZAMIDE
1WBW	2.41	MAP kinase 14	LI4	3-(1-NAPHTHYLMETHOXY)PYRIDIN-2-AMINE
1WCC	2.2	Cyclin-dependent kinase 2	CIG	2-AMINO-6-CHLOROPYRAZINE
1WZY	2.5	MAP kinase 1	F29	1-ALLYL-5-(2-PHENYLPYRAZOLO[1,5-A]PYRIDIN-3-YL)-1H-PYRAZOLO[3,4-C]PYRIDAZIN-3-AMINE
1X8B	1.81	Wee1-like protein kinase	824	9-HYDROXY-4-PHENYLPYRROLO[3,4-C]CARBAZOLE-1,3(2H,6H)-DIONE
1XBB	1.57	Tyrosine-protein kinase SYK	STI	4-(4-METHYL-PIPERAZIN-1-YLMETHYL)-N-[4-METHYL-3-(4-PYRIDIN-3-YL-PYRIMIDIN-2-YLAMINO)-PHENYL]-BENZAMIDE
1XH5	2.05	cAMP-dependent protein kinase	R68	N-{4-[(4-{3-[(2R)-3,3-DIMETHYLPIPERIDIN-2-YL]-2-FLUORO-6-HYDROXYBENZOYL}BENZOYL)AMINO]AZEPAN-3-YL}ISONICOTINAMIDE
1XH6	1.9	cAMP-dependent protein kinase	R94	N-(4-{[4-(2-HYDROXY-5-PIPERIDIN-1-YLBENZOYL)BENZOYL]AMINO}AZEPAN-3-YL)ISONICOTINAMIDE

1XH7	2.47	cAMP-dependent protein kinase	R96	N-[4-({4-[5-(3,3-DIMETHYLPIPERIDIN-1-YL)-2-HYDROXYBENZOYL]BENZOYL}AMINO)AZEPAN-3-YL]ISONICOTINAMIDE
1XH8	1.6	cAMP-dependent protein kinase	R55	N-[4-({4-[5-(4,4-DIMETHYLPIPERIDIN-1-YL)-2-HYDROXYBENZOYL]BENZOYL}AMINO)AZEPAN-3-YL]ISONICOTINAMIDE
1XH9	1.64	cAMP-dependent protein kinase	R69	N-[4-({4-[5-(DIMETHYLAMINO)-2-HYDROXYBENZOYL]BENZOYL}AMINO)AZEPAN-3-YL]ISONICOTINAMIDE
1XKK	2.4	Epidermal growth factor receptor	FMM	N-{3-CHLORO-4-[(3-FLUOROBENZYL)OXY]PHENYL}-6-[5-({[2-(METHYLSULFONYL)ETHYL]AMINO}METHYL)-2-FURYL]-4-QUINAZOLINAMINE
1Y57	1.91	Proto-oncogene tyrosine-protein kinase Src	MPZ	4-[(4-METHYLPIPERAZIN-1-YL)METHYL]-N-{3-[(4-PYRIDIN-3-YLPYRIMIDIN-2-YL)AMINO]PHENYL}BENZAMIDE
1Y6A	2.1	Vascular endothelial growth factor receptor 2	AAZ	N-[5-(ETHYLSULFONYL)-2-METHOXYPHENYL]-5-[3-(2-PYRIDINYL)PHENYL]-1,3-OXAZOL-2-AMINE
1Y6B	2.1	Vascular endothelial growth factor receptor 2	AAX	N-(CYCLOPROPYLMETHYL)-4-(METHYLOXY)-3-({5-[3-(3-PYRIDINYL)PHENYL]-1,3-OXAZOL-2-YL}AMINO)BENZENESULFONAMIDE
1Y8Y	1.996	Cyclin-dependent kinase 2	CT7	(5-CHLOROPYRAZOLO[1,5-A]PYRIMIDIN-7-YL)-(4-METHANESULFONYLPHENYL)AMINE
1Y91	2.15	Cyclin-dependent kinase 2	CT9	4-[5-(TRANS-4-AMINOCYCLOHEXYLAMINO)-3-ISOPROPYLPYRAZOLO[1,5-A]PYRIMIDIN-7-YLAMINO]-N,N-DIMETHYLBENZENESULFONAMIDE
1YDS	2.2	cAMP-dependent protein kinase	IQS	N-[2-(METHYLAMINO)ETHYL]-5-ISOQUINOLINESULFONAMIDE
1YI3	2.5	Proto-oncogene serine/threonine-protein kinase	LY2	2-MORPHOLIN-4-YL-7-PHENYL-4H-CHROMEN-4-ONE
1YKR	1.8	Cyclin-dependent kinase 2	628	4-{[6-(2,6-DICHLOROBENZOYL)IMIDAZO[1,2-A]PYRIDIN-2-YL]AMINO}BENZENESULFONAMIDE
1YOL	2.3	Proto-oncogene tyrosine-protein kinase Src	S03	1-{4-[4-AMINO-5-(3-METHOXYPHENYL)-7H-PYRROLO[2,3-D]PYRIMIDIN-7-YL]BENZYL}PIPERIDIN-4-OL
1YQJ	2	MAP kinase 14	6NP	6((S)-3-BENZYLPIPERAZIN-1-YL)-3-(NAPHTHALEN-2-YL)-4-(PYRIDIN-4-YL)PYRAZINE
1YW2	2.01	MAP kinase 14	PGJ	2-(ETHOXYMETHYL)-4-(4-FLUOROPHENYL)-3-[2-(2-HYDROXYPHENOXY)PYRIMIDIN-4-YL]ISOXAZOL-5(2H)-ONE
1YWN	1.71	Vascular endothelial growth factor receptor 2	LIF	N-{4-[4-AMINO-6-(4-METHOXYPHENYL)FURO[2,3-D]PYRIMIDIN-5-YL]PHENYL}-N'-[2-FLUORO-5-(TRIFLUOROMETHYL)PHENYL]UREA
1YWR	1.95	MAP kinase 14	LI9	4-(4-FLUOROPHENYL)-1-METHYL-5-(2-{[(1S)-1-PHENYLETHYL]AMINO}PYRIMIDIN-4-YL)-2-PIPERIDIN-4-YL-1,2-DIHYDRO-3H-PYRAZOL-3-ONE
1YXV	2	Proto-oncogene serine/threonine-protein kinase	LI6	3,4-DIHYDROXY-1-METHYLQUINOLIN-2(1H)-ONE
1YXX	2	Proto-oncogene serine/threonine-protein kinase	LI7	(3E)-3-[(4-HYDROXYPHENYL)IMINO]-1H-INDOL-2(3H)-ONE
1Z57	1.7	Dual specificity protein kinase CLK1	DBQ	DEBROMOHYMENIALDISINE
1Z5M	2.17	3-phosphoinositide dependent protein kinase-1	LI8	N-(3-{[5-BROMO-2-({3-[(PYRROLIDIN-1-YLCARBONYL)AMINO]PHENYL}AMINO)PYRIMIDIN-4-YL]AMINO}PROPYL)-2,2-DIMETHYLMALONAMIDE
1ZLT	1.74	Serine/threonine-protein kinase Chk1	HYM	(4Z)-4-(2-AMINO-5-OXO-3,5-DIHYDRO-4H-IMIDAZOL-4-YLIDENE)-2,3-DICHLORO-4,5,6,7-TETRAHYDROPYRROLO[2,3-C]AZEPIN-8(1H)-ONE
1ZOE	1.77	Protein kinase CK2	K25	4,5,6,7-TETRABROMO-N,N-DIMETHYL-1H-BENZIMIDAZOL-2-AMINE
1ZOG	2.3	Protein kinase CK2	K37	4,5,6,7-TETRABROMO-2-(METHYLSULFANYL)-1H-BENZIMIDAZOLE
1ZOH	1.81	Protein kinase CK2	K44	5,6,7,8-TETRABROMO-1-METHYL-2,3-DIHYDRO-1H-IMIDAZO[1,2-A]BENZIMIDAZOLE
1ZYJ	2	MAP kinase 14	BI5	4-PHENOXY-N-(PYRIDIN-2-YLMETHYL)BENZAMIDE
1ZYS	1.7	Serine/threonine-protein kinase Chk1	199	N-{5-[4-(4-METHYLPIPERAZIN-1-YL)PHENYL]-1H-PYRROLO[2,3-B]PYRIDIN-3-YL}NICOTINAMIDE
1ZZ2	2	MAP kinase 14	B11	N-[3-(4-FLUOROPHENOXY)PHENYL]-4-[(2-HYDROXYBENZYL)AMINO]PIPERIDINE-1-SULFONAMIDE
1ZZL	2	MAP kinase 14	TZY	6-[4-(4-FLUOROPHENYL)-1,3-OXAZOL-5-YL]-3-ISOPROPYL[1,2,4]TRIAZOLO[4,3-A]PYRIDINE
2A0C	1.95	Cyclin-dependent kinase 2	CK9	2-{[(2-{[(1R)-1-(HYDROXYMETHYL)PROPYL]AMINO}-9-ISOPROPYL-9H-PURIN-6-YL)AMINO]METHYL}PHENOL
2B1P	1.9	MAP kinase 10	AIZ	3-{6-[(2-CHLOROPHENYL)AMINO]-1H-INDAZOL-3-YL}-5-{[4-(DIMETHYLAMINO)BUTANOYL]AMINO}BENZOIC ACID
2B52	1.88	Cyclin-dependent kinase 2	D42	1-(3-(2,4-DIMETHYLTHIAZOL-5-YL)-4-OXO-2,4-DIHYDROINDENO[1,2-C]PYRAZOL-5-YL)-3-(4-METHYLPIPERAZIN-1-YL)UREA
2B53	2	Cyclin-dependent kinase 2	D23	6-(3-AMINOPHENYL)-N-(TERT-BUTYL)-2-(TRIFLUOROMETHYL)QUINAZOLIN-4-AMINE
2B54	1.85	Cyclin-dependent kinase 2	D05	6-(3,4-DIHYDROXYBENZYL)-3-ETHYL-1-(2,4,6-TRICHLOROPHENYL)-1H-PYRAZOLO[3,4-D]PYRIMIDIN-4(5H)-ONE
2B55	1.85	Cyclin-dependent kinase 2	D31	2-(4-(AMINOMETHYL)PIPERIDIN-1-YL)-N-(3_CYCLOHEXYL-4-OXO-2,4-DIHYDROINDENO[1,2-C]PYRAZOL-5-YL)ACETAMIDE
2BAJ	2.25	MAP kinase 14	1PP	1-(3-tert-butyl-1-phenyl-1H-pyrazol-5-yl)-3-(2,3-dichlorophenyl)urea
2BAL	2.1	MAP kinase 14	PQA	[5-AMINO-1-(4-FLUOROPHENYL)-1H-PYRAZOL-4-YL][3-(PIPERIDIN-4-YLOXY)PHENYL]METHANONE
2BDF	2.1	Proto-oncogene tyrosine-protein kinase Src	24A	{[(4-{[2-(4-AMINOCYCLOHEXYL)-9-ETHYL-9H-PURIN-6-YL]AMINO}PHENYL)(HYDROXY)PHOSPHORYL]METHYL}PHOSPHONIC ACID
2BDJ	2.5	Proto-oncogene tyrosine-protein kinase Src	HET	3-[2-(2-CYCLOPENTYL-6-{[4-(DIMETHYLPHOSPHORYL)PHENYL]AMINO}-9H-PURIN-9-YL)ETHYL]PHENOL
2BHE	1.9	Cyclin-dependent kinase 2	BRY	(2Z)-5'-BROMO-2,3'-BIINDOLE-2',3(1H,1'H)-DIONE AMMONIATE
2BIK	1.8	Proto-oncogene serine/threonine-protein kinase	BI1	3-{1-[3-(DIMETHYLAMINO)PROPYL]-1H-INDOL-3-YL}-4-(1H-INDOL-3-YL)-1H-PYRROLE-2,5-DIONE
2BPM	2.4	Cyclin-dependent kinase 2	529	(2S)-N-[(3Z)-5-CYCLOPROPYL-3H-PYRAZOL-3-YLIDENE]-2-[4-(2-OXOIMIDAZOLIDIN-1-YL)PHENYL]PROPANAMIDE
2BR1	2	Serine/threonine-protein kinase Chk1	PFP	2-[5,6-BIS-(4-METHOXY-PHENYL)-FURO[2,3-D]PYRIMIDIN-4-YLAMINO]-ETHANOL
2BRG	2.1	Serine/threonine-protein kinase Chk1	DFY	(5,6-DIPHENYL-FURO[2,3-D]PYRIMIDIN-4-YLAMINO)-ACETIC
2BRH	2.1	Serine/threonine-protein kinase Chk1	DFW	N-(5,6-DIPHENYLFURO[2,3-D]PYRIMIDIN-4-YL)GLYCINE
2BRM	2.2	Serine/threonine-protein kinase Chk1	DFZ	3-AMINO-3-BENZYL-[4.3.0]BICYCLO-1,6-DIAZANONAN-2-ONE
2BRO	2.2	Serine/threonine-protein kinase Chk1	DF2	(2R)-3-{[(4Z)-5,6-DIPHENYL-6,7-DIHYDRO-4H-PYRROLO[2,3-D]PYRIMIDIN-4-YLIDENE]AMINO}PROPANE-1,2-DIOL
2BTR	1.85	Cyclin-dependent kinase 2	U73	N-(5-ISOPROPYL-THIAZOL-2-YL)-2-PYRIDIN-3-YL-ACETAMIDE
2BTS	1.99	Cyclin-dependent kinase 2	U32	4-[(5-ISOPROPYL-1,3-THIAZOL-2-YL)AMINO]BENZENESULFONAMIDE
2BZH	1.9	Proto-oncogene serine/threonine-protein kinase	HB1	RUTHENIUM-PYRIDOCARBAZOLE-1
2BZJ	2.05	Proto-oncogene serine/threonine-protein kinase	ME3	RUTHENIUM-PYRIDOCARBAZOLE-3
2C0I	2.3	Tyrosine-protein kinase HCK	L1G	N-(4-{4-AMINO-1-[4-(4-METHYLPIPERAZIN-1-YL)-TRANS-CYCLOHEXYL]-1H-PYRAZOLO[3,4-D]PYRIMIDIN-3-YL}-2-METHOXYPHENYL)-1-METHYL-1H-INDOLE-2-CARBOXAMIDE
2C0T	2.15	Tyrosine-protein kinase HCK	L3G	N-(4-{4-AMINO-1-[1-(TETRAHYDRO-2H-PYRAN-4-YL)PIPERIDIN-4-YL]-1H-PYRAZOLO[3,4-D]PYRIMIDIN-3-YL}-2-METHOXYPHENYL)-1-METHYL-1H-INDOLE-2-CARBOXAMIDE

2C1A	1.95	cAMP-dependent protein kinase	I5S	ISOQUINOLINE-5-SULFONIC ACID (2-(2-(4-CHLOROBENZYLOXY)ETHYLAMINO)ETHYL)AMIDE
2C1B	2	cAMP-dependent protein kinase	CQP	(4R,2S)-5'-(4-(4-CHLOROBENZYLOXY)PYRROLIDIN-2-YLMETHANESULFONYL)ISOQUINOLINE
2C3L	2.35	Serine/threonine-protein kinase Chk1	IDZ	3-(1H-BENZIMIDAZOL-2-YL)-1H-INDAZOLE
2C5N	2.1	Cyclin-dependent kinase 2	CK8	N-[4-(2,4-DIMETHYL-THIAZOL-5-YL)-PYRIMIDIN-2-YL]-N',N'-DIMETHYL-BENZENE-1,4-DIAMINE
2C5Y	2.25	Cyclin-dependent kinase 2	MTW	HYDROXY(OXO)(3-{[(2Z)-4-[3-(1H-1,2,4-TRIAZOL-1-YLMETHYL)PHENYL]PYRIMIDIN-2(5H)-YLIDENE]AMINO}PHENYL)AMMONIUM
2C68	1.95	Cyclin-dependent kinase 2	CT6	(5Z)-5-(3-BROMOCYCLOHEXA-2,5-DIEN-1-YLIDENE)-N-(PYRIDIN-4-YLMETHYL)-1,5-DIHYDROPYRAZOLO[1,5-A]PYRIMIDIN-7-AMINE
2C69	2.1	Cyclin-dependent kinase 2	CT8	(5Z)-5-(3-BROMOCYCLOHEXA-2,5-DIEN-1-YLIDENE)-N-(PYRIDIN-4-YLMETHYL)-1,5-DIHYDRO[1,2,4]TRIAZOLO[1,5-A]PYRIMIDIN-7-AMINE
2C6E	2.1	Serine/threonine-protein kinase 6	HPM	N-{5-[(7-{[(2S)-2-HYDROXY-3-PIPERIDIN-1-YLPROPYL]OXY}-6-METHOXYQUINAZOLIN-4-YL)AMINO]PYRIMIDIN-2-YL}BENZAMIDE
2C6I	1.8	Cyclin-dependent kinase 2	DT1	4-{[5-(CYCLOHEXYLMETHOXY)[1,2,4]TRIAZOLO[1,5-A]PYRIMIDIN-7-YL]AMINO}BENZENESULFONAMIDE
2C6K	1.9	Cyclin-dependent kinase 2	DT2	4-{[5-(CYCLOHEXYLAMINO)[1,2,4]TRIAZOLO[1,5-A]PYRIMIDIN-7-YL]AMINO}BENZENESULFONAMIDE
2C6L	2.3	Cyclin-dependent kinase 2	DT4	4-({5-[(4-AMINOCYCLOHEXYL)AMINO][1,2,4]TRIAZOLO[1,5-A]PYRIMIDIN-7-YL}AMINO)BENZENESULFONAMIDE
2C6M	1.9	Cyclin-dependent kinase 2	DT5	4-{[5-(CYCLOHEXYLOXY)[1,2,4]TRIAZOLO[1,5-A]PYRIMIDIN-7-YL]AMINO}BENZENESULFONAMIDE
2CGU	2.5	Serine/threonine-protein kinase Chk1	3A3	2,2'-{[9-(HYDROXYIMINO)-9H-FLUORENE-2,7-DIYL]BIS(OXY)}DIACETIC ACID
2CGW	2.2	Serine/threonine-protein kinase Chk1	3C3	4,4'-(1-PROPYL-1H-1,2,4-TRIAZOLE-3,5-DIYL)BIS(2,5-DIHYDRO-1,2,5-OXADIAZOL-3-AMINE)
2CGX	2.2	Serine/threonine-protein kinase Chk1	3D3	2-[(6-AMINO-7H-PURIN-8-YL)THIO]ACETAMIDE
2CLX	1.8	Cyclin-dependent kinase 2	F18	4-[(E)-(3,5-DIAMINO-1H-PYRAZOL-4-YL)DIAZENYL]PHENOL
2CMW	1.75	Casein kinase I	OLP	2-(2-HYDROXYETHYLAMINO)-6-(3-CHLOROANILINO)-9-ISOPROPYLPURINE
2CSN	2.5	Casein kinase I	CKI	N-(2-AMINOETHYL)-5-CHLOROISOQUINOLINE-8-SULFONAMIDE
2DS1	2	Cyclin-dependent kinase 2	1CD	(13R,15S)-13-METHYL-16-OXA-8,9,12,22,24-PENTAAZAHEXACYCLO[15.6.2.16,9.1,12,15.0,2,7.0,21,25]HEPTACOSA-1(24),2,4,6,17(25),18,20-HEPTAENE-23,26-DIONE
2DUV	2.2	Cyclin-dependent kinase 2	371	2-(3,4-DIHYDROXYPHENYL)-8-(1,1-DIOXIDOISOTHIAZOLIDIN-2-YL)-3-HYDROXY-6-METHYL-4H-CHROMEN-4-ONE
2E2B	2.2	Proto-oncogene tyrosine-protein kinase ABL1	406	N-[3-(4,5'-BIPYRIMIDIN-2-YLAMINO)-4-METHYLPHENYL]-4-{[(3S)-3-(DIMETHYLAMINO)PYRROLIDIN-1-YL]METHYL}-3-(TRIFLUOROMETHYL)BENZAMIDE
2E9N	2.5	Serine/threonine-protein kinase Chk1	76A	3-(4'-HYDROXYBIPHENYL-4-YL)-N-(4-HYDROXYCYCLOHEXYL)-1,4-DIHYDROINDENO[1,2-C]PYRAZOLE-6-CARBOXAMIDE
2E9U	2	Serine/threonine-protein kinase Chk1	A25	18-CHLORO-11,12,13,14-TETRAHYDRO-1H,10H-8,4-(AZENO)-9,15,1,3,6-BENZODIOXATRIAZACYCLOHEPTADECIN-2-ONE
2E9V	2	Serine/threonine-protein kinase Chk1	85A	18-CHLORO-2-OXO-17-[(PYRIDIN-4-YLMETHYL)AMINO]-2,3,11,12,13,14-HEXAHYDRO-1H,10H-4,8-(AZENO)-9,15,1,3,6-BENZODIOXATRIAZACYCLOHEPTADECINE-7-CARBONITRILE
2ERZ	2.2	cAMP-dependent protein kinase	HFS	1-(1-HYDROXY-5-ISOQUINOLINESULFONYL)HOMOPIPERAZINE
2ETM	2.3	Focal adhesion kinase 1	7PY	7-PYRIDIN-2-YL-N-(3,4,5-TRIMETHOXYPHENYL)-7H-PYRROLO[2,3-D]PYRIMIDIN-2-AMINE
2EXM	1.8	Cyclin-dependent kinase 2	ZIP	N-(3-METHYLBUT-2-EN-1-YL)-9H-PURIN-6-AMINE
2F57	1.8	Serine/threonine-protein kinase PAK 7	23D	N2-[(1R,2S)-2-AMINOCYCLOHEXYL]-N6-(3-CHLOROPHENYL)-9-ETHYL-9H-PURINE-2,6-DIAMINE
2F7E	2	cAMP-dependent protein kinase	2EA	(1S)-2-(1H-INDOL-3-YL)-1-{[(5-ISOQUINOLIN-6-YLPYRIDIN-3-YL)OXY]METHYL}ETHYLAMINE
2F7X	1.9	Protein kinase A	4EA	(1S)-2-(1H-INDOL-3-YL)-1-[({5-[(E)-2-PYRIDIN-4-YLVINYL]PYRIDIN-3-YL}OXY)METHYL]ETHYLAMINE
2FGI	2.5	Fibroblast growth factor receptor 1	PD1	1-TERT-BUTYL-3-[6-(3,5-DIMETHOXY-PHENYL)-2-(4-DIETHYLAMINO-BUTYLAMINO)-PYRIDO[2,3-D]PYRIMIDIN-7-YL]-UREA
2FVD	1.85	Cyclin-dependent kinase 2	LIA	(4-AMINO-2-{[1-(METHYLSULFONYL)PIPERIDIN-4-YL]AMINO}PYRIMIDIN-5-YL)(2,3-DIFLUORO-6-METHOXYPHENYL)METHANONE
2G9X	2.5	Cyclin-dependent kinase 2	NU5	3-({2-[(4-{[6-(CYCLOHEXYLMETHOXY)-9H-PURIN-2-YL]AMINO}PHENYL)SULFONYL]ETHYL}AMINO)PROPAN-1-OL
2GFS	1.752	MAP Kinase 14	PQB	[5-AMINO-1-(4-FLUOROPHENYL)-1H-PYRAZOL-4-YL](3-{[(2R)-2,3-DIHYDROXYPROPYL]OXY}PHENYL)METHANONE
2GHL	2.099	MAP kinase 14	LIB	3-(2-CHLOROPHENYL)-1-(2-{[(1S)-2-HYDROXY-1,2-DIMETHYLPROPYL]AMINO}PYRIMIDIN-4-YL)-1-(4-METHOXYPHENYL)UREA
2GHM	2.35	MAP kinase 14	LIC	3-(2-CHLOROBENZYL)-1-(2-{[(1S)-2-HYDROXY-1,2-DIMETHYLPROPYL]AMINO}PYRIMIDIN-4-YL)-1-(4-METHOXYPHENYL)UREA
2GTM	1.9	MAP kinase 14	LID	8-(2-CHLOROPHENYLAMINO)-2-(2,6-DIFLUOROPHENYLAMINO)-9-ETHYL-9H-PURINE-1,7-DIIUM
2GTN	1.8	MAP kinase 14	LIE	2-(2,6-DIFLUOROPHENOXY)-N-(2-FLUOROPHENYL)-9-ISOPROPYL-9H-PURIN-8-AMINE
2GU8	2.2	cAMP-dependent protein kinase	796	N-[(1S)-2-AMINO-1-(2,4-DICHLOROBENZYL)ETHYL]-5-[2-(METHYLAMINO)PYRIMIDIN-4-YL]THIOPHENE-2-CARBOXAMIDE
2HIW	2.2	Proto-oncogene tyrosine-protein kinase ABL1	7MP	7-AMINO-1-METHYL-3-(2-METHYL-5-{[3-(TRIFLUOROMETHYL)BENZOYL]AMINO}PHENYL)-2-OXO-2,3-DIHYDROPYRIMIDO[4,5-D]PYRIMIDIN-1-IUM
2HK5	2	Tyrosine-protein kinase HCK	1BM	3-{[2-(1H-BENZIMIDAZOL-1-YL)-6-{[2-(DIETHYLAMINO)ETHYL]AMINO}PYRIMIDIN-4-YL]AMINO}-4-METHYLPHENOL
2HOG	1.9	Serine/threonine-protein kinase Chk1	710	(5-{3-[5-(PIPERIDIN-1-YLMETHYL)-1H-INDOL-2-YL]-1H-INDAZOL-6-YL}-2H-1,2,3-TRIAZOL-4-YL)METHANOL
2HWO	2.5	Proto-oncogene tyrosine-protein kinase Src	RBS	N-(4-PHENYLAMINO-QUINAZOLIN-6-YL)-ACRYLAMIDE
2HXL	1.8	Serine/threonine-protein kinase Chk1	422	3-(5-{[4-(AMINOMETHYL)PIPERIDIN-1-YL]METHYL}-1H-INDOL-2-YL)-1H-INDAZOLE-6-CARBONITRILE
2HXQ	2	Serine/threonine-protein kinase Chk1	373	3-(5-{[4-(AMINOMETHYL)PIPERIDIN-1-YL]METHYL}-1H-INDOL-2-YL)QUINOLIN-2(1H)-ONE
2HY0	1.7	Serine/threonine-protein kinase Chk1	306	3-[5-(PIPERIDIN-1-YLMETHYL)-1H-INDOL-2-YL]-6-(1H-PYRAZOL-4-YL)QUINOLIN-2(1H)-ONE
2HY8	2	Serine/threonine-protein kinase PAK 1	1ST	(5S,6R,7R,9R)-12-HYDROXY-6-METHOXY-5-METHYL-7-(METHYLAMINO)-6,7,8,9-TETRAHYDRO-5H,14H-5,9-EPOXY-4B,9A,15-TRIAZADIBENZO[B,H]CYCLONONA[1,2,3,4-JKL]CYCLOPENTA[E]-AS-INDACEN-14-ONE
2HZ0	2.1	Proto-oncogene tyrosine-protein kinase ABL1	GIN	2-{[(6-OXO-1,6-DIHYDROPYRIDIN-3-YL)METHYL]AMINO}-N-[4-PROPYL-3-(TRIFLUOROMETHYL)PHENYL]BENZAMIDE
2HZI	1.7	Proto-oncogene tyrosine-protein kinase ABL1	JIN	6-(2,6-DICHLOROPHENYL)-2-[(4-FLUORO-3-METHYLPHENYL)AMINO]-8-METHYLPYRIDO[2,3-D]PYRIMIDIN-7(8H)-ONE
2I0H	2	MAP kinase 14	222	2-(3-{(2-CHLORO-4-FLUOROPHENYL)[1-(2-CHLOROPHENYL)-6-OXO-1,6-DIHYDROPYRIDAZIN-3-YL]AMINO}PROPYL)-1H-ISOINDOLE-1,3(2H)-DIONE
2I1M	1.8	Macrophage colony-stimulating factor 1 receptor	5CN	5-CYANO-FURAN-2-CARBOXYLIC ACID [5-HYDROXYMETHYL-2-(4-METHYL-PIPERIDIN-1-YL)-PHENYL]-AMIDE
2IN6	1.9	Wee1-like protein kinase	839	3-(9-HYDROXY-1,3-DIOXO-4-PHENYL-2,3-DIHYDROPYRROLO[3,4-C]CARBAZOL-6(1H)-YL)PROPANOIC ACID
2IO6	2.2	Wee1-like protein kinase	330	9-HYDROXY-6-(3-HYDROXYPROPYL)-4-(2-METHOXYPHENYL)PYRROLO[3,4-C]CARBAZOLE-1,3(2H,6H)-DIONE

2IVU	2.5	Proto-oncogene tyrosine-protein kinase Receptor	ZD6	Vandetanib
2IW6	2.3	Cyclin-dependent kinase 2	QQ2	[(2-CHLORO-5-METHYLPHENYL){6-[(4-{[(2R)-3-(DIMETHYLAMINO)-2-HYDROXYPROPYL]OXY}PHENYL)AMINO]PYRIMIDIN-4-YL}AMINO]ACETONITRILE
2IZR	1.3	Casein kinase I	BRK	{(2Z)-4-AMINO-2-[(4-METHOXYPHENYL)IMINO]-2,3-DIHYDRO-1,3-THIAZOL-5-YL}(4-METHOXYPHENYL)METHANONE
2IZS	1.95	Casein kinase I	BRQ	{[4-AMINO-2-(3-CHLOROANILINO)-1,3-THIAZOL-5-YL](4-FLUOROPHENYL)METHANONE
2J2I	1.9	Proto-oncogene serine/threonine-protein kinase	LY4	(9R)-9-[(DIMETHYLAMINO)METHYL]-6,7,10,11-TETRAHYDRO-9H,18H-5,21:12,17-DIMETHENODIBENZO[E,K]PYRROLO[3,4-H][1,4,13]OXADIAZACYCLOHEXADECINE-18,20-DIONE
2J7T	2	Serine/threonine-protein kinase 10	274	(3Z)-N-(3-CHLOROPHENYL)-3-({3,5-DIMETHYL-4-[(4-METHYLPIPERAZIN-1-YL)CARBONYL]-1H-PYRROL-2-YL}METHYLENE)-N-METHYL-2-OXOINDOLINE-5-SULFONAMIDE
2J9M	2.5	Cyclin-dependent kinase 2	PY8	6-BROMO-13-THIA-2,4,8,12,19-PENTAAZATRICYCLO[12.3.1.1~3,7~]NONADECA-1(18),3(19),4,6,14,16-HEXAENE 13,13-DIOXIDE
2JAV	2.2	Serine/threonine-protein kinase NEK2	5Z5	5-[(Z)-(5-CHLORO-2-OXO-1,2-DIHYDRO-3H-INDOL-3-YLIDENE)METHYL]-N-(DIETHYLAMINO)ETHYL]-2,4-DIMETHYL-1H-PYRROLE-3-CARBOXAMIDE
2JC6	2.3	Calcium/calmodulin-dependent protein kinase	QPP	N-(5-METHYL-1H-PYRAZOL-3-YL)-2-PHENYLQUINAZOLIN-4-AMINE
2JED	2.32	Protein kinase C	LG8	3-(8-DIMETHYLAMINOMETHYL-6,7,8,9-TETRAHYDRO-PYRIDO[1,2-A]INDOL-10-YL)-4-(1-METHYL-1H-INDOL-3-YL)-PYRROLE-2,5-DIONE
2JKM	2.31	Focal adhesion kinase 1	BII	2-{[5-CHLORO-2-({(1E,4R)-2-METHOXY-4-[(3R)-3-(METHYLAMINO)PYRROLIDIN-1-YL]CYCLOHEXA-2,5-DIEN-1-YLIDENE}AMINO)PYRIMIDIN-4-YL]AMINO}-N-(1-METHYLETHYL)BENZENESULFONAMIDE
2JKO	1.65	Focal adhesion kinase 1	BIJ	7-{[(2Z,5S)-5-CHLORO-2-{[2-METHOXY-4-(4-METHYLPIPERAZIN-1-YL)PHENYL]IMINO}-2,5-DIHYDROPYRIMIDIN-4-YL]AMINO}-2-METHYL-2,3-DIHYDRO-1H-ISOINDOL-1-ONE
2JLD	2.35	Glycogen synthase kinase-3 beta	AG1	RUTHENIUM PYRIDOCARBAZOLE
2NP8	2.25	Serine/threonine-protein kinase 6	CC3	N-{3-[(4-{[3-(TRIFLUOROMETHYL)PHENYL]AMINO}PYRIMIDIN-2-YL)AMINO]PHENYL}CYCLOPROPANECARBOXAMIDE
2NRU	2	Interleukin-1 receptor-associated kinase 4	T12	1-(3-HYDROXYPROPYL)-2-[(3-NITROBENZOYL)AMINO]-1H-BENZIMIDAZOL-5-YL PIVALATE
2O0U	2.1	MAP kinase 10	C0M	N-{3-CYANO-6-[3-(1-PIPERIDINYL)PROPANOYL]-4,5,6,7-TETRAHYDROTHIENO[2,3-C]PYRIDIN-2-YL}1-NAPHTHALENECARBOXAMIDE
2O2U	2.45	MAP kinase 10	738	N-(3-cyano-4,5,6,7-tetrahydro-1-benzothien-2-yl)-2-fluorobenzamide
2O63	2	Proto-oncogene serine/threonine-protein kinase	MYC	3,5,7-TRIHYDROXY-2-(3,4,5-TRIHYDROXYPHENYL)-4H-CHROMEN-4-ONE
2O64	2.44	Proto-oncogene serine/threonine-protein kinase	MYU	3,5,6,7-TETRAHYDROXY-2-(3,4-DIHYDROXYPHENYL)-4H-CHROMEN-4-ONE
2OBJ	2.5	Proto-oncogene serine/threonine-protein kinase	VRV	6-(5-BROMO-2-HYDROXYPHENYL)-2-OXO-4-PHENYL-1,2-DIHYDROPYRIDINE-3-CARBONITRILE
2OF2	2	Proto-oncogene tyrosine-protein kinase LCK	547	2,3-DIPHENYL-N-(2-PIPERAZIN-1-YLETHYL)FURO[2,3-B]PYRIDIN-4-AMINE
2OFU	2	Proto-oncogene tyrosine-protein kinase LCK	1N9	2,6-DIMETHYLPHENYL 2-(3,5-DIMETHOXY-4-(3-(4-METHYLPIPERAZIN-1-YL)PROPOXY)PHENYLAMINO)PYRIMIDIN- 4-YL(2,4-DIMETHOXYPHENYL)CARBAMATE
2OFV	2	Proto-oncogene tyrosine-protein kinase LCK	242	3-(2-AMINOQUINAZOLIN-6-YL)-4-METHYL-N-[3-(TRIFLUOROMETHYL)PHENYL]BENZAMIDE
2OG8	2.3	Proto-oncogene tyrosine-protein kinase LCK	1N8	N-{2-[(N,N-DIETHYLGLYCYL)AMINO]-5-(TRIFLUOROMETHYL)PHENYL}-4-METHYL-3-[2-(METHYLAMINO)QUINAZOLIN-6-YL]BENZAMIDE
2OH0	2.2	cAMP-dependent protein kinase	2PY	(2S)-1-{[5-(1H-INDAZOL-5-YL)PYRIDIN-3-YL]OXY}-3-[(7AS)-7AH-INDOL-3-YL]PROPAN-2-AMINE
2OH4	2.05	Vascular endothelial growth factor receptor 2	GIG	METHYL (5-{4-[({[2-FLUORO-5-(TRIFLUOROMETHYL)PHENYL]AMINO}CARBONYL)AMINO]PHENOXY}-1H-BENZIMIDAZOL-2-YL)CARBAMATE
2OJ9	2	Insulin-like growth factor 1 receptor	BMI	3-[5-(1H-IMIDAZOL-1-YL)-7-METHYL-1H-BENZIMIDAZOL-2-YL]-4-[(PYRIDIN-2-YLMETHYL)AMINO]PYRIDIN-2(1H)-ONE
2OJF	2.1	cAMP-dependent protein kinase	4PY	(2S)-1-(6H-INDOL-3-YL)-3-{[5-(7H-PYRAZOLO[3,4-C]PYRIDIN-5-YL)PYRIDIN-3-YL]OXY}PROPAN-2-AMINE
2OJG	2	MAP kinase 1	19A	N,N-DIMETHYL-4-(4-PHENYL-1H-PYRAZOL-3-YL)-1H-PYRROLE-2-CARBOXAMIDE
2OJJ	2.4	MAP kinase 1	82A	(S)-N-(1-(3-CHLORO-4-FLUOROPHENYL)-2-HYDROXYETHYL)-4-(4-(3-CHLOROPHENYL)-1H-PYRAZOL-3-YL)-1H-PYRROLE-2-CARBOXAMIDE
2OK1	2.4	MAP kinase 10	33A	N-BENZYL-4-[4-(3-CHLOROPHENYL)-1H-PYRAZOL-3-YL]-1H-PYRROLE-2-CARBOXAMIDE
2OO8	2.2	Angiopoietin-1 receptor	RAJ	N-{3-[3-(DIMETHYLAMINO)PROPYL]-5-(TRIFLUOROMETHYL)PHENYL}-4-METHYL-3-[(3-PYRIMIDIN-4-YLPYRIDIN-2-YL)AMINO]BENZAMIDE
2OWB	2.1	Serine/threonine-protein kinase PLK1	626	4-(4-METHYLPIPERAZIN-1-YL)-N-[5-(2-THIENYLACETYL)-1,5-DIHYDROPYRROLO[3,4-C]PYRAZOL-3-YL]BENZAMIDE
2OXD	2.3	Casein kinase II	K32	4,5,6,7-TETRABROMO-1H,3H-BENZIMIDAZOL-2-ONE
2OXX	2.3	Casein kinase II	K22	4,5,6,7-TETRABROMO-1H,3H-BENZIMIDAZOL-2-THIONE
2OXY	1.812	Casein kinase II	K17	4,5,6,7-TETRABROMO-BENZIMIDAZOLE
2P2H	1.95	Vascular endothelial growth factor receptor 2	994	4-(2-anilinopyridin-3-yl)-N-(3,4,5-trimethoxyphenyl)-1,3,5-triazin-2-amine
2P2I	2.4	Vascular endothelial growth factor receptor 2	608	N-(4-phenoxyphenyl)-2-[(pyridin-4-ylmethyl)amino]nicotinamide
2P33	2.4	c-Jun N-terminal kinase 3	J07	4-{[5-chloro-4-(1H-indol-3-yl)pyrimidin-2-yl]amino}-N-ethylpiperidine-1-carboxamide
2P4I	2.5	Angiopoietin-1 receptor	MR9	4-METHYL-3-({3-[2-(METHYLAMINO)PYRIMIDIN-4-YL]PYRIDIN-2-YL}OXY)-N-[2-MORPHOLIN-4-YL-5-(TRIFLUOROMETHYL)PHENYL]BENZAMIDE
2PE0	2.35	3-phosphoinositide-dependent protein kinase 1	39Z	5-HYDROXY-3-[(1R)-1-(1H-PYRROL-2-YL)ETHYL]-2H-INDOL-2-ONE
2PE1	2.14	3-phosphoinositide-dependent protein kinase 1	517	1-{2-OXO-3-[(1R)-1-(1H-PYRROL-2-YL)ETHYL]-2H-INDOL-5-YL}UREA
2PE2	2.13	3-phosphoinositide-dependent protein kinase 1	464	3-[5-({5-[(AMINOCARBONYL)AMINO]-2-OXO-2H-INDOL-3-YL}METHYL)-1H-PYRROL-3-YL]-N-(2-PIPERIDIN-1-YLETHYL)BENZAMIDE
2PUU	2.5	MAP kinase 14	G2G	5-TERT-BUTYL-2-(4-METHYLPHENYL)-3-{[({4-[6-(MORPHOLIN-4-YLMETHYL)PYRIDIN-3-YL]-1-NAPHTHYL}AMINO)CARBONYL]AMINO}-1H-PYRAZOL-2-IUM
2PVH	2.2	Casein kinase II	P19	N,N'-DIPHENYLPYRAZOLO[1,5-A][1,3,5]TRIAZINE-2,4-DIAMINE
2PVJ	1.7	Casein kinase II	P44	2-(CYCLOHEXYLMETHYLAMINO)-4-(PHENYLAMINO)PYRAZOLO[1,5-A][1,3,5]TRIAZINE-8-CARBONITRILE
2PVK	1.9	Casein kinase II	P45	2-(4-CHLOROBENZYLAMINO)-4-(PHENYLAMINO)PYRAZOLO[1,5-A][1,3,5]TRIAZINE-8-CARBONITRILE
2PVL	1.9	Casein kinase II	P55	2-(4-ETHYLPIPERAZIN-1-YL)-4-(PHENYLAMINO)PYRAZOLO[1,5-A][1,3,5]TRIAZINE-8-CARBONITRILE
2PVM	2	Casein kinase II	P29	4-(2-(1H-IMIDAZOL-4-YL)ETHYLAMINO)-2-(PHENYLAMINO)PYRAZOLO[1,5-A][1,3,5]TRIAZINE-8-CARBONITRILE
2PVN	2	Casein kinase II	P63	N-(3-(8-CYANO-4-(PHENYLAMINO)PYRAZOLO[1,5-A][1,3,5]TRIAZIN-2-YLAMINO)PHENYL)ACETAMIDE
2PZI	2.4	Probable serine/threonine-protein kinase pknG	AXX	2-[(CYCLOPROPYLCARBONYL)AMINO]-4,5,6,7-TETRAHYDRO-1-BENZOTHIOPHENE-3-CARBOXAMIDE
2QC6	1.85	Casein kinase II	G12	3,8-DIBROMO-7-HYDROXY-4-METHYL-2H-CHROMEN-2-ONE
2QD9	1.7	MAP kinase 14	LGF	1-[5-[[3-[2,4-bis(fluoranyl)phenyl]-6,8-dihydro-5~{H}-imidazo[1,5-a]pyrazin-7-yl]carbonyl]-6-methoxy-1~{H}-pyrrolo[2,3-b]pyridin-3-yl]-2-[(3~{R})-3-oxidanylpyrrolidin-1-yl]ethane-1,2-dione

2QHM	2	Serine/threonine-protein kinase Chk1	7CS	(3-ENDO)-8-METHYL-8-AZABICYCLO[3.2.1]OCT-3-YL 1H-PYRROLO[2,3-B]PYRIDINE-3-CARBOXYLATE
2QHN	1.7	Serine/threonine-protein kinase Chk1	582	5-ETHYL-3-METHYL-1,5-DIHYDRO-4H-PYRAZOLO[4,3-C]QUINOLIN-4-ONE
2QU6	2.1	Vascular endothelial growth factor receptor 2	857	4-({2-[(4-chloro-3-{[(2S)-1-methylpyrrolidin-2-yl]methoxy}phenyl)amino]-1,3-benzoxazol-5-yl}oxy)-N-methylpyridine-2-carboxamide
2R0U	1.9	Serine/threonine-protein kinase Chk1	M54	6-(3-aminopropyl)-4-(3-hydroxyphenyl)-9-(1H-pyrazol-4-yl)benzo[h]isoquinolin-1(2H)-one
2R3F	1.5	Cyclin-dependent kinase 2	SC8	5-(2,3-dichlorophenyl)-N-(pyridin-4-ylmethyl)pyrazolo[1,5-a]pyrimidin-7-amine
2R3G	1.55	Cyclin-dependent kinase 2	SC9	6-(2-fluorophenyl)-N-(pyridin-3-ylmethyl)imidazo[1,2-a]pyrazin-8-amine
2R3H	1.5	Cyclin-dependent kinase 2	SCE	3-methyl-N-(pyridin-4-ylmethyl)imidazo[1,2-a]pyrazin-8-amine
2R3I	1.28	Cyclin-dependent kinase 2	SCF	5-(2-fluorophenyl)-N-(pyridin-4-ylmethyl)pyrazolo[1,5-a]pyrimidin-7-amine
2R3J	1.65	Cyclin-dependent kinase 2	SCJ	3-bromo-5-phenyl-N-(pyridin-3-ylmethyl)pyrazolo[1,5-a]pyrimidin-7-amine
2R3K	1.7	Cyclin-dependent kinase 2	SCQ	3-bromo-5-phenyl-N-(pyrimidin-5-ylmethyl)pyrazolo[1,5-a]pyridin-7-amine
2R3L	1.65	Cyclin-dependent kinase 2	SCW	3-bromo-6-phenyl-N-(pyrimidin-5-ylmethyl)imidazo[1,2-a]pyridin-8-amine
2R3M	1.7	Cyclin-dependent kinase 2	SCX	N-((2-aminopyrimidin-5-yl)methyl)-5-(2,6-difluorophenyl)-3-ethylpyrazolo[1,5-a]pyrimidin-7-amine
2R3N	1.63	Cyclin-dependent kinase 2	SCZ	3-cyclopropyl-5-phenyl-N-(pyridin-3-ylmethyl)pyrazolo[1,5-a]pyrimidin-7-amine
2R3O	1.8	Cyclin-dependent kinase 2	2SC	(5-phenyl-7-(pyridin-3-ylmethylamino)pyrazolo[1,5-a]pyrimidin-3-yl)methanol
2R3P	1.66	Cyclin-dependent kinase 2	3SC	5-(2,3-dichlorophenyl)-N-(pyridin-4-ylmethyl)-3-thiocyanatopyrazolo[1,5-a]pyrimidin-7-amine
2R3Q	1.35	Cyclin-dependent kinase 2	5SC	3-((3-bromo-5-o-tolylpyrazolo[1,5-a]pyrimidin-7-ylamino)methyl)pyridine 1-oxide
2R3R	1.47	Cyclin-dependent kinase 2	6SC	3-bromo-5-phenyl-N-(pyridin-4-ylmethyl)pyrazolo[1,5-a]pyrimidin-7-amine
2R4B	2.4	Receptor tyrosine-protein kinase erbB-4	GW7	N-{3-chloro-4-[(3-fluorobenzyl)oxy]phenyl}-6-ethylthieno[3,2-d]pyrimidin-4-amine
2R64	2.3	Cyclin-dependent kinase 2	740	N-[5-(1,1-DIOXIDOISOTHIAZOLIDIN-2-YL)-1H-INDAZOL-3-YL]-2-(4-PIPERIDIN-1-YLPHENYL)ACETAMIDE
2R9S	2.4	MAP kinase 10	255	N-(tert-butyl)-4-[5-(pyridin-2-ylamino)quinolin-3-yl]benzenesulfonamide
2RFN	2.5	Hepatocyte growth factor receptor	AM7	2-benzyl-5-(3-fluoro-4-{[6-methoxy-7-(3-morpholin-4-ylpropoxy)quinolin-4-yl]oxy}phenyl)-3-methylpyrimidin-4(3H)-one
2RFS	2.2	Hepatocyte growth factor receptor	AM8	N-(3-chlorophenyl)-N-methyl-2-oxo-3-[(3,4,5-trimethyl-1H-pyrrol-2-yl)methyl]-2H-indole-5-sulfonamide
2RG5	2.4	MAP kinase 14	279	N-ethyl-4-{[5-(methoxycarbamoyl)-2-methylphenyl]amino}-5-methylpyrrolo[2,1-f][1,2,4]triazine-6-carboxamide
2RG6	1.72	MAP kinase 14	287	4-{[5-(methoxycarbamoyl)-2-methylphenyl]amino}-5-methyl-N-[(1S)-1-phenylethyl]pyrrolo[2,1-f][1,2,4]triazine-6-carboxamide
2RGP	2	Epidermal growth factor receptor	HYZ	N-[1-(3-fluorobenzyl)-1H-indazol-5-yl]-5-[(piperidin-1-ylamino)methyl]pyrimidine-4,6-diamine
2UUE	2.06	Cyclin-dependent kinase 2	MTZ	4-METHYL-5-{(2E)-2-[(4-MORPHOLIN-4-YLPHENYL)IMINO]-2,5-DIHYDROPYRIMIDIN-4-YL}-1,3-THIAZOL-2-AMINE
2UVX	2	cAMP-dependent protein kinase	GVH	1H-PYRROLO[2,3-B]PYRIDINE
2UVY	1.95	cAMP-dependent protein kinase	GVI	N-METHYL-1-[4-(9H-PURIN-6-YL)PHENYL]METHANAMINE
2UVZ	1.94	cAMP-dependent protein kinase	GVJ	(S)-1-PHENYL-1-[4-(9H-PURIN-6-YL)PHENYL]METHANAMINE
2UW0	2	cAMP-dependent protein kinase	GVK	6-{4-[4-(4-CHLOROPHENYL)PIPERIDIN-4-YL]PHENYL}-9H-PURINE
2UW3	2.19	cAMP-dependent protein kinase	GVG	3-METHYL-4-PHENYL-1H-PYRAZOLE
2UW4	2	cAMP-dependent protein kinase	L15	2-[4-(3-METHYL-1H-PYRAZOL-4-YL)PHENYL]ETHANAMINE
2UW5	2.14	cAMP-dependent protein kinase	GVN	(2R)-2-(4-CHLOROPHENYL)-2-[4-(1H-PYRAZOL-4-YL)PHENYL]ETHANAMINE
2UW6	2.23	cAMP-dependent protein kinase	GVO	(2S)-2-(4-CHLOROPHENYL)-2-[4-(1H-PYRAZOL-4-YL)PHENYL]ETHANAMINE
2UW7	2.1	cAMP-dependent protein kinase	GVP	4-(4-CHLOROPHENYL)-4-[4-(1H-PYRAZOL-4-YL)PHENYL]PIPERIDINE
2UW8	2	cAMP-dependent protein kinase	GVQ	(2R)-2-(4-CHLOROPHENYL)-2-PHENYLETHANAMINE
2UZE	2.4	Cyclin-dependent kinase 2	C95	4-{5-[(Z)-(2-IMINO-4-OXO-1,3-THIAZOLIDIN-5-YLIDENE)METHYL]FURAN-2-YL}BENZOIC ACID
2UZL	2.4	Cyclin-dependent kinase 2	C94	4-{5-[(Z)-(2-IMINO-4-OXO-1,3-THIAZOLIDIN-5-YLIDENE)METHYL]FURAN-2-YL}-2-(TRIFLUOROMETHYL)BENZENESULFONAMIDE
2UZN	2.3	Cyclin-dependent kinase 2	C96	4-{5-[(1Z)-1-(2-IMINO-4-OXO-1,3-THIAZOLIDIN-5-YLIDENE)ETHYL]-2-FURYL}BENZENESULFONAMIDE
2UZO	2.3	Cyclin-dependent kinase 2	C62	4-{5-[(Z)-(2,4-DIOXO-1,3-THIAZOLIDIN-5-YLIDENE)METHYL]FURAN-2-YL}BENZENESULFONAMIDE
2UZT	2.1	cAMP-dependent protein kinase	SS3	(2S)-1-{[5-(3-METHYL-1H-INDAZOL-5-YL)PYRIDIN-3-YL]OXY}-3-PHENYLPROPAN-2-AMINE
2UZV	2.5	cAMP-dependent protein kinase	SS5	(2S)-1-[3-(CYCLOHEXYLMETHOXY)PHENYL]-3-{[5-(3-METHYL-1H-INDAZOL-5-YL)PYRIDIN-3-YL]OXY}PROPAN-2-AMINE
2UZW	2.2	cAMP-dependent protein kinase	SS4	3-PYRIDIN-4-YL-2,4-DIHYDRO-INDENO[1,2-.C.] PYRAZOLE
2V0D	2.2	Cyclin-dependent kinase 2	C53	2-IMINO-5-(1-PYRIDIN-2-YL-METH-(E)-YLIDENE)-1,3-THIAZOLIDIN-4-ONE
2V4L	2.5	Phosphatidylinositol 4,5-bisphosphate 3-kinase	ABJ	3-[4-AMINO-1-(1-METHYLETHYL)-1H-PYRAZOLO[3,4-D]PYRIMIDIN-3-YL]PHENOL
2VAG	1.8	Dual specificity protein kinase CLK1	V25	ethyl 3-[(E)-2-amino-1-cyanoethenyl]-6,7-dichloro-1-methyl-1H-indole-2-carboxylate
2VGO	1.7	Serine/threonine-protein kinase 12-A	AD5	N~6~-cyclohexyl-N~2~-(4-morpholin-4-ylphenyl)-9H-purine-2,6-diamine
2VGP	1.7	Serine/threonine-protein kinase 12-A	AD6	4-[(5-bromo-1,3-thiazol-2-yl)amino]-N-methylbenzamide
2VNW	2.09	cAMP-dependent protein kinase	M01	1-[1-(9H-purin-6-yl)piperidin-4-yl]methanamine
2VNY	1.96	cAMP-dependent protein kinase	M02	1-(9H-purin-6-yl)piperidin-4-amine
2VO0	1.94	cAMP-dependent protein kinase	M03	1-[4-(4-chlorophenyl)-1-(7H-pyrrolo[2,3-d]pyrimidin-4-yl)piperidin-4-yl]methanamine
2VO3	1.98	cAMP-dependent protein kinase	M04	1-[4-(4-chlorobenzyl)-1-(7H-pyrrolo[2,3-d]pyrimidin-4-yl)piperidin-4-yl]methanamine
2VO6	1.97	cAMP-dependent protein kinase	M05	4-(4-chlorobenzyl)-1-(7H-pyrrolo[2,3-d]pyrimidin-4-yl)piperidin-4-aminium

2VRX	1.86	Serine/threonine-protein kinase 12-A	447	N-(4-{[6-methoxy-7-(3-morpholin-4-ylpropoxy)quinazolin-4-yl]amino}phenyl)benzamide
2VTA	2	Cyclin-dependent kinase 2	LZ1	1H-indazole
2VTH	1.9	Cyclin-dependent kinase 2	LZ2	5-hydroxynaphthalene-1-sulfonamide
2VTI	2	Cyclin-dependent kinase 2	LZ3	N-(4-sulfamoylphenyl)-1H-indazole-3-carboxamide
2VTJ	2.2	Cyclin-dependent kinase 2	LZ4	4-[(6-chloropyrazin-2-yl)amino]benzenesulfonamide
2VTL	2	Cyclin-dependent kinase 2	LZ5	N-phenyl-1H-pyrazole-3-carboxamide
2VTM	2.25	Cyclin-dependent kinase 2	LZM	PYRAZOLO[1,5-A]PYRIMIDINE-3-CARBONITRILE
2VTN	2.2	Cyclin-dependent kinase 2	LZ7	4-(acetylamino)-N-(4-fluorophenyl)-1H-pyrazole-3-carboxamide
2VTO	2.19	Cyclin-dependent kinase 2	LZ8	N-(4-FLUOROPHENYL)-4-[(PHENYLCARBONYL)AMINO]-1H-PYRAZOLE-3-CARBOXAMIDE
2VTQ	1.9	Cyclin-dependent kinase 2	LZA	{[(2,6-difluorophenyl)carbonyl]amino}-N-piperidin-4-yl-1H-pyrazole-3-carboxamide
2VTR	1.89	Cyclin-dependent kinase 2	LZB	5-chloro-7-[(1-methylethyl)amino]pyrazolo[1,5-a]pyrimidine-3-carbonitrile
2VTS	1.9	Cyclin-dependent kinase 2	LZC	5-[(4-AMINOCYCLOHEXYL)AMINO]-7-(PROPAN-2-YLAMINO)PYRAZOLO[1,5-A]PYRIMIDINE-3-CARBONITRILE
2VTT	1.68	Cyclin-dependent kinase 2	LZD	4-{[(2,6-difluorophenyl)carbonyl]amino}-N-[(3S)-piperidin-3-yl]-1H-pyrazole-3-carboxamide
2VU3	1.85	Cyclin-dependent kinase 2	LZE	4-{[(2,6-dichlorophenyl)carbonyl]amino}-N-piperidin-4-yl-1H-pyrazole-3-carboxamide
2VV9	1.9	Cyclin-dependent kinase 2	IM9	2-{4-[4-({4-[2-methyl-1-(1-methylethyl)-1H-imidazol-5-yl]pyrimidin-2-yl}amino)phenyl]piperazin-1-yl}-2-oxoethanol
2VWU	2	Ephrin type-B receptor 4	7X1	N-(5-chloro-1,3-benzodioxol-4-yl)-6-methoxy-7-(3-piperidin-1-ylpropoxy)quinazolin-4-amine
2VWV	1.9	Ephrin type-B receptor 4	7X3	N'-(3-CHLORO-4-METHOXY-PHENYL)-N-(3,4,5-TRIMETHOXYPHENYL)-1,3,5-TRIAZINE-2,4-DIAMINE
2VWW	1.9	Ephrin type-B receptor 4	7X2	N'-(5-CHLORO-1,3-BENZODIOXOL-4-YL)-N-(3,4,5- TRIMETHOXYPHENYL)PYRIMIDINE-2,4-DIAMINE
2VWX	1.65	Ephrin type-B receptor 4	7X4	3-({4-[(5-chloro-1,3-benzodioxol-4-yl)amino]pyrimidin-2-yl}amino)benzenesulfonamide
2VWY	1.65	Ephrin type-B receptor 4	7X5	N'-(5-chloro-1,3-benzodioxol-4-yl)-N-(3-methylsulfonylphenyl)pyrimidine-2,4-diamine
2VWZ	1.65	Ephrin type-B receptor 4	7X6	N-[3-[[4-[(5-CHLORO-1,3-BENZODIOXOL-4-YL)AMINO]PYRIMIDIN-2-YL]AMINO]PHENYL]METHANESULFONAMIDE
2VX0	2.1	Ephrin type-B receptor 4	7X7	N'-(5-CHLORO-1,3-BENZODIOXOL-4-YL)-N-(3-MORPHOLIN-4-YLPHENYL)PYRIMIDINE-2,4-DIAMINE
2VX1	1.65	Ephrin type-B receptor 4	7X8	3-({4-[(5-CHLORO-1,3-BENZODIOXOL-4-YL)AMINO]PYRIMIDIN-2-YL}AMINO)BENZAMIDE
2VZ6	2.3	Calcium/calmodulin-dependent protein kinase	FEF	(2Z,3E)-2,3'-BIINDOLE-2',3(1H,1'H)-DIONE 3-{O-[(3R)-3,4-DIHYDROXYBUTYL]OXIME}
2W05	1.9	Cyclin-dependent kinase 2	FRT	N-(2-METHOXYETHYL)-4-({4-[2-METHYL-1-(1-METHYLETHYL)-1H-IMIDAZOL-5-YL]PYRIMIDIN-2-YL}AMINO)BENZENESULFONAMIDE
2W06	2.04	Cyclin-dependent kinase 2	FRV	4-{[4-(1-CYCLOPROPYL-2-METHYL-1H-IMIDAZOL-5-YL)PYRIMIDIN-2-YL]AMINO}-N-METHYLBENZENESULFONAMIDE
2W0J	2.05	Serine/threonine-protein kinase Chk2	ZAT	4,4'-DIACETYLDIPHENYLUREA-BIS(GUANYLHYDRAZONE)
2W17	2.15	Cyclin-dependent kinase 2	I19	N-(4-{[(3S)-3-(dimethylamino)pyrrolidin-1-yl]carbonyl}phenyl)-5-fluoro-4-[2-methyl-1-(1-methylethyl)-1H-imidazol-5-yl]pyrimidin-2-amine
2W1H	2.15	Cyclin-dependent kinase 2	L0F	N-[3-(1H-BENZIMIDAZOL-2-YL)-1H-PYRAZOL-4-YL]BENZAMIDE
2W7X	2.07	Serine/threonine-protein kinase Chk2	D1A	N-[4-[(E)-N-carbamimidamido-C-methyl-carbonimidoyl]phenyl]-7-nitro-1H-indole-2-carboxamide
2WAJ	2.4	MAP kinase 10	SNB	1-(3-BROMOPHENYL)-7-CHLORO-6-METHOXY-3,4-DIHYDROISOQUINOLINE
2WD1	2	Hepatocyte growth factor receptor	ZZY	1-[(2-NITROPHENYL)SULFONYL]-1H-PYRROLO[3,2-B]PYRIDINE-6-CARBOXAMIDE
2WEI	1.65	Calmodulin-domain protein kinase 1	VGG	1-tert-butyl-3-(3-methylbenzyl)-1H-pyrazolo[3,4-d]pyrimidin-4-amine
2WEL	1.9	Calcium/calmodulin-dependent protein kinase	K88	(3Z)-N,N-DIMETHYL-2-OXO-3-(4,5,6,7-TETRAHYDRO-1H-INDOL-2-YLMETHYLIDENE)-2,3-DIHYDRO-1H-INDOLE-5-SULFONAMIDE
2WGJ	2	Hepatocyte growth factor receptor	VGH	3-[(1R)-1-(2,6-dichloro-3-fluorophenyl)ethoxy]-5-(1-piperidin-4-yl-1H-pyrazol-4-yl)pyridin-2-amine
2WKM	2.2	Hepatocyte growth factor receptor	PFY	(3Z)-5-[(2,6-DICHLOROBENZYL)SULFONYL]-3-[(3,5-DIMETHYL-4-{[(2S)-2-(PYRROLIDIN-1-YLMETHYL)PYRROLIDIN-1-YL]CARBONYL}-1H-PYRROL-2-YL)METHYLIDENE]-1,3-DIHYDRO-2H-INDOL-2-ONE
2WMQ	2.48	Serine/threonine-protein kinase Chk1	ZYQ	N-(4-OXO-5,6,7,8-TETRAHYDRO-4H-[1,3]THIAZOLO[5,4-C]AZEPIN-2-YL)ACETAMIDE
2WMR	2.43	Serine/threonine-protein kinase Chk1	ZYR	5,6,7,8-TETRAHYDRO[1]BENZOTHIENO[2,3-D]PYRIMIDIN-4(3H)-ONE
2WMV	2.009	Serine/threonine-protein kinase Chk1	ZYV	1-[(2S)-4-(7H-PURIN-6-YL)MORPHOLIN-2-YL]METHANAMINE
2WMW	2.43	Serine/threonine-protein kinase Chk1	ZYW	1-[(2S)-4-(5-BROMO-1H-PYRAZOLO[3,4-B]PYRIDIN-4-YL)MORPHOLIN-2-YL]METHANAMINE
2WMX	2.45	Serine/threonine-protein kinase Chk1	ZY6	1-[(2S)-4-(5-phenyl-1H-pyrazolo[3,4-b]pyridin-4-yl)morpholin-2-yl]methanamine
2WOT	1.85	TGF-beta receptor type I	ZZG	4-[(5,6-DIMETHYL-2,2'-BIPYRIDIN-3-YL)OXY]-N-(3,4,5-TRIMETHOXYPHENYL)PYRIDIN-2-AMINE
2WOU	2.3	TGF-beta receptor type I	ZZF	4-({4-[(2,6-DIMETHYLPYRIDIN-3-YL)OXY]PYRIDIN-2-YL}AMINO)BENZENESULFONAMIDE
2WQO	2.167	Serine/threonine-protein kinase NEK2	VGK	4-[2-AMINO-5-(3,4,5-TRIMETHOXYPHENYL)PYRIDIN-3-YL]BENZOIC ACID
2WTI	2.5	Checkpoint kinase 2	WTI	4-[2-AMINO-5-(2,3-DIHYDROTHIENO[3,4-B][1,4]DIOXIN-5-YL)PYRIDIN-3-YL]BENZAMIDE
2WTJ	2.1	Checkpoint kinase 2	WTJ	2-AMINO-5-(2,3-DIHYDROTHIENO[3,4-B][1,4]DIOXIN-5-YL)-N-[2-(DIMETHYLAMINO)ETHYL]PYRIDINE-3-CARBOXAMIDE
2WXF	1.9	Phosphatidylinositol 4,5-bisphosphate 3-kinase	039	2-((9H-PURIN-6-YLTHIO)METHYL)-5-CHLORO-3-(2-METHOXYPHENYL)QUINAZOLIN-4(3H)-ONE
2WXG	2	Phosphatidylinositol 4,5-bisphosphate 3-kinase	ZZN	2-{[4-amino-3-(3-fluoro-5-hydroxyphenyl)-1H-pyrazolo[3,4-d]pyrimidin-1-yl]methyl}-5-methyl-3-(2-methylphenyl)quinazolin-4(3H)-one
2WXH	1.9	Phosphatidylinositol 4,5-bisphosphate 3-kinase	ZZO	2-{[4-amino-3-(3-fluoro-4-hydroxyphenyl)-1H-pyrazolo[3,4-d]pyrimidin-1-yl]methyl}-5-methyl-3-(2-methylphenyl)quinazolin-4(3H)-one
2WXL	1.99	Phosphatidylinositol 4,5-bisphosphate 3-kinase	ZS4	2-(difluoromethyl)-1-(4,6-dimorpholin-4-yl-1,3,5-triazin-2-yl)-1H-benzimidazole
2WXO	2.49	Phosphatidylinositol 4,5-bisphosphate 3-kinase	ZZP	N-(3-{[(1Z)-3,5-DIMETHOXYCYCLOHEXA-2,4-DIEN-1-YLIDENE]AMINO}QUINOXALIN-2-YL)-4-FLUOROBENZENESULFONAMIDE
2WXP	2.3	Phosphatidylinositol 4,5-bisphosphate 3-kinase	GD9	2-(1H-indazol-4-yl)-6-{[4-(methylsulfonyl)piperazin-1-yl]methyl}-4-morpholin-4-yl-thieno[3,2-d]pyrimidine
2X2L	2	Proto-oncogene tyrosine-protein kinase Receptor	X2L	(3Z)-5-AMINO-3-[(4-METHOXYPHENYL)METHYLIDENE]-1,3-DIHYDRO-2H-INDOL-2-ONE

2X2M	2.5	Proto-oncogene tyrosine-protein kinase Receptor	X2M	(3Z)-3-[(3,5-DIMETHYL-1H-PYRROL-2-YL)METHYLIDENE]-1,3-DIHYDRO-2H-INDOL-2-ONE
2X38	2.2	Phosphatidylinositol 4,5-bisphosphate 3-kinase	IC8	2-[(6-AMINO-9H-PURIN-9-YL)METHYL]-5-METHYL-3-(2-METHYLPHENYL)QUINAZOLIN-4(3H)-ONE
2X39	1.93	RAC-alpha serine/threonine-protein kinase	X39	4-AMINO-N-(4-CHLOROBENZYL)-1-(7H-PYRROLO[2,3-D]PYRIMIDIN-4-YL)PIPERIDINE-4-CARBOXAMIDE
2X8D	1.9	Serine/threonine-protein kinase Chk1	X8D	5-METHYL[1,2,4]TRIAZOLO[4,3-A]QUINOLIN-1(2H)-ONE
2X8E	2.5	Serine/threonine-protein kinase Chk1	X8E	5-METHYL-8-PYRIDIN-4-YL[1,2,4]TRIAZOLO[4,3-A]QUINOLIN-1(2H)-ONE
2X8I	1.92	Serine/threonine-protein kinase Chk1	X8I	7-(3-hydroxyphenyl)-5-methyl[1,2,4]triazolo[4,3-a]quinolin-1(2H)-one
2X9F	1.75	Ephrin type-B receptor 4	X9F	N^4^-1H-INDAZOL-4-YL-N^2^-[3-(METHYLSULFONYL)PHENYL]PYRIMIDINE-2,4-DIAMINE
2XA4	2.04	Tyrosine-protein kinase JAK2	AZ5	5-CHLORO-N2-[(1S)-1-(5-FLUOROPYRIMIDIN-2-YL)ETHYL]-N4-(5-METHYL-1H-PYRAZOL-3-YL)PYRIMIDINE-2,4-DIAMINE
2XBA	1.95	ALK tyrosine kinase receptor	571	5-[(2R)-2-hydroxy-2-phenylacetyl]-3-({[4-(4-methylpiperazin-1-yl)phenyl]carbonyl}amino)-1,6-dihydropyrrolo[3,4-c]pyrazol-5-ium
2XBJ	2.3	Serine/threonine-protein kinase Chk2	XBJ	4-FLUORO-2-(4-{[(3S,4R)-4-(1-HYDROXY-1-METHYLETHYL)PYRROLIDIN-3-YL]AMINO}-6,7-DIMETHOXYQUINAZOLIN-2-YL)PHENOL
2XCH	2	3-phosphoinositide dependent protein kinase-1	CKG	8-(CYCLOHEXA-2,5-DIEN-1-YLIDENEAMINO)-1-(PIPERIDIN-4-YLMETHYL)-4,5-DIHYDRO-1H-PYRAZOLO[4,3-H]QUINAZOLINE-3-CARBOXAMIDE
2XCK	2.3	3-phosphoinositide dependent protein kinase-1	MH4	1-METHYL-8-{[4-(4-METHYLPIPERAZIN-1-YL)PHENYL]AMINO}-N-[(2-METHYLPYRIDIN-4-YL)METHYL]-4,5-DIHYDRO-1H-PYRAZOLO[4,3-H]QUINAZOLINE-3-CARBOXAMIDE
2XEZ	2.25	Serine/threonine-protein kinase Chk1	XEZ	6-(1H-PYRAZOL-3-YL)-3-(1H-PYRAZOL-4-YL)IMIDAZO[1,2-A]PYRAZINE
2XF0	2.4	Serine/threonine-protein kinase Chk1	4UB	3-PHENYL-6-(1H-PYRAZOL-4-YL)IMIDAZO[1,2-A]PYRAZINE
2XIR	1.5	Vascular endothelial growth factor receptor 2	00J	N,2-DIMETHYL-6-{[7-(2-MORPHOLIN-4-YLETHOXY)QUINOLIN-4-YL]OXY}-1-BENZOFURAN-3-CARBOXAMIDE
2XIX	2.4	Proto-oncogene serine/threonine-protein kinase	XIX	3,5-DIAMINO-1H-[1,2,4]TRIAZOLE
2XIY	2.2	Proto-oncogene serine/threonine-protein kinase	XIY	2-HYDROXYMETHYL-BENZOIMIDAZOLE
2XIZ	2.21	Proto-oncogene serine/threonine-protein kinase	XIZ	(E)-PYRIDIN-4-YL-ACRYLIC ACID
2XJ1	2.13	Proto-oncogene serine/threonine-protein kinase	XJ1	(2E)-3-(3-{6-[(TRANS-4-AMINOCYCLOHEXYL)AMINO]PYRAZIN-2-YL}PHENYL)PROP-2-ENOIC ACID
2XK3	2.2	Serine/threonine-protein kinase NEK2	XK3	4-[3-AMINO-6-(3-ETHYLTHIOPHEN-2-YL)PYRAZIN-2-YL]CYCLOHEXANE-1-CARBOXYLIC ACID
2XK4	2.1	Serine/threonine-protein kinase NEK2	OL2	4-[3-amino-6-(3,4,5-trimethoxyphenyl)pyrazin-2-yl]-2-ethoxybenzoic acid
2XK6	2.2	Serine/threonine-protein kinase NEK2	EQH	cis-4-[3-amino-6-(3-cyclopropylthiophen-2-yl)pyrazin-2-yl]cyclohexanecarboxylic acid
2XK7	1.992	Serine/threonine-protein kinase NEK2	30E	(3R,4R)-1-[3-amino-6-(3,4,5-trimethoxyphenyl)pyrazin-2-yl]-3-ethylpiperidine-4-carboxylic acid
2XK8	2.001	Serine/threonine-protein kinase NEK2	5R1	4-[3-amino-6-(3,4,5-trimethoxyphenyl)pyrazin-2-yl]-2-methoxybenzoic acid
2XK9	2.35	Checkpoint kinase 2	XK9	N-{4-[(1E)-N-(N-hydroxycarbamimidoyl)ethanehydrazonoyl]phenyl}-7-nitro-1H-indole-2-carboxamide
2XKC	2.5	Serine/threonine-protein kinase NEK2	4VQ	4-[3-amino-6-(3,4,5-trimethoxyphenyl)pyrazin-2-yl]-2-methylbenzoic acid
2XKD	1.96	Serine/threonine-protein kinase NEK2	T3M	4-[3-amino-6-(3,4,5-trimethoxyphenyl)pyrazin-2-yl]benzoic acid
2XKE	2.203	Serine/threonine-protein kinase NEK2	WI2	1-[3-amino-6-(3-methoxyphenyl)pyrazin-2-yl]piperidine-4-carboxylic acid
2XKF	2.35	Serine/threonine-protein kinase NEK2	BX1	1-[3-amino-6-(3,4,5-trimethoxyphenyl)pyrazin-2-yl]piperidine-4-carboxylic acid
2XM9	2.5	Serine/threonine-protein kinase Chk2	LWH	4-(1H-pyrazol-5-yl)-2-{4-[(3S)-pyrrolidin-3-ylamino]quinazolin-2-yl}phenol
2XMY	1.9	Cyclin-dependent kinase 2	CDK	4-[4-(3,4-DIMETHYL-2-OXO-2,3-DIHYDRO-THIAZOL-5-YL)-PYRIMIDIN-2-YLAMINO]-N-(2-METHOXY-ETHYL)-BENZENESULFONAMIDE
2XNB	1.85	Cyclin-dependent kinase 2	Y8L	3,4-DIMETHYL-5-(2-{[(1Z)-4-PIPERAZIN-1-YLCYCLOHEXA-2,4-DIEN-1-YLIDENE]AMINO}PYRIMIDIN-4-YL)-1,3-THIAZOL-2(3H)-ONE
2XNM	1.85	Serine/threonine-protein kinase NEK2	WGZ	5-{6-[(1-METHYLPIPERIDIN-4-YL)OXY]-1H-BENZIMIDAZOL-1-YL}-3-{(1R)-1-[2-(TRIFLUOROMETHYL)PHENYL]ETHOXY}THIOPHENE-2-CARBOXAMIDE
2XNN	2.5	Serine/threonine-protein kinase NEK2	430	5-(1H-benzimidazol-1-yl)-3-{(1R)-1-[2-(trifluoromethyl)phenyl]ethoxy}thiophene-2-carboxamide
2XNO	1.98	Serine/threonine-protein kinase NEK2	ED8	5-{6-[(1-methylpiperidin-4-yl)oxy]-1H-benzimidazol-1-yl}-3-{[2-(trifluoromethyl)benzyl]oxy}thiophene-2-carboxamide
2XNP	1.98	Serine/threonine-protein kinase NEK2	WCX	4-{5-[(1-METHYLPIPERIDIN-4-YL)OXY]-1H-BENZIMIDAZOL-1-YL}-2-{(1R)-1-[2-(TRIFLUOROMETHYL)PHENYL]ETHOXY}BENZAMIDE
2XVD	1.7	Ephrin type-B receptor 4	AS6	{4-METHYL-3-[(1-METHYLETHYL)(2-{[3-(METHYLSULFONYL)-5-MORPHOLIN-4-YLPHENYL]AMINO}PYRIMIDIN-4-YL)AMINO]PHENYL}METHANOL
2XYU	2.117	Ephrin type-A receptor 4	Q9G	5-(5-FLUORO-2-METHYLPHENYL)-6,7,8,9-TETRAHYDRO-3H-PYRAZOLO[3,4-C]ISOQUINOLIN-1-AMINE
2YAC	2.2	Serine/threonine-protein kinase PLK1	937	1-(2-HYDROXYETHYL)-8-[[5-(4-METHYLPIPERAZIN-1-YL)-2-(TRIFLUOROMETHOXY)PHENYL]AMINO]-4,5-DIHYDROPYRIMIDO[5,4-G]INDAZOLE-3-CARBOXAMIDE
2YAK	2.2	Death-associated protein kinase 1	OSV	RUTHENIUM OCTASPORINE 4
2YCF	1.77	Serine/threonine-protein kinase Chk2	YCF	(2E)-N-hydroxy-2-[1-(4-{[(4-{(1E)-1-[2-(N'-hydroxycarbamimidoyl)hydrazinylidene]ethyl}phenyl)carbamoyl]amino}phenyl)ethylidene]hydrazinecarboximidamide
2YCQ	2.05	Serine/threonine-protein kinase Chk2	UPX	N-{4-[(1E)-N-1H-IMIDAZOL-2-YLETHANEHYDRAZONOYL]PHENYL}-7-NITRO-1H-INDOLE-2-CARBOXAMIDE
2YCR	2.2	Serine/threonine-protein kinase Chk2	HCW	1,3-BIS{4-[(1E)-N-(4,5-DIHYDRO-1H-IMIDAZOL-2-YL)ETHANEHYDRAZONOYL]PHENYL}UREA
2YCS	2.35	Serine/threonine-protein kinase Chk2	NXP	N-{4-[(1E)-N-CARBAMIMIDOYLETHANEHYDRAZONOYL]PHENYL}-1H-INDOLE-3-CARBOXAMIDE
2YDI	1.6	Serine/threonine-protein kinase Chk1	YDI	5-[4-(2-DIMETHYLAMINOETHYLOXY)PHENYL]-2-UREIDO-THIOPHENE-3-CARBOXAMIDE
2YDK	1.9	Serine/threonine-protein kinase Chk1	YDK	2-(CARBAMOYLAMINO)-5-PHENYL-N-[(3S)-PIPERIDIN-3-YL]THIOPHENE-3-CARBOXAMIDE
2YER	1.83	Serine/threonine-protein kinase Chk1	TQ1	5-(HYDROXYMETHYL)-8-(1H-PYRROL-2-YL)-2H-[1,2,4]TRIAZOLO[4,3-A]QUINOLIN-1-ONE
2YEX	1.3	Serine/threonine-protein kinase Chk1	YEX	5-METHYL-8-(1H-PYRROL-2-YL)[1,2,4]TRIAZOLO[4,3-A]QUINOLIN-1(2H)-ONE
2YIQ	1.89	Serine/threonine-protein kinase Chk2	YIQ	(E)-5-(1-(2-CARBAMIMIDOYLHYDRAZONO)ETHYL)-N-(1H-INDOL-6-YL)-1H-INDOLE-2-CARBOXAMIDE
2YIR	2.1	Serine/threonine-protein kinase Chk2	YIR	(E)-N-(5-(2-CARBAMIMIDOYLHYDRAZONO)-5,6,7,8-TETRAHYDRONAPHTHALEN-2-YL)-7-NITRO-1H-INDOLE-2-CARBOXAMIDE
2YIS	2	MAP kinase 14	YIS	1-[3-tert-butyl-1-(3-chloro-4-hydroxyphenyl)-1H-pyrazol-5-yl]-3-{2-[(3-{2-[(2-hydroxyethyl)sulfanyl]phenyl}[1,2,4]triazolo[4,3-a]pyridin-6-yl)sulfanyl]benzyl}urea
2YIT	2.2	Serine/threonine-protein kinase Chk2	YIT	N-{4-[(1E)-N-carbamimidoylbutanehydrazonoyl]phenyl}-5-methoxy-1H-indole-2-carboxamide
2YIW	2	MAP kinase 14	YIW	1-(3-tert-butyl-1-phenyl-1H-pyrazol-5-yl)-3-(2-{[3-(1-methylethyl)[1,2,4]triazolo[4,3-a]pyridin-6-yl]sulfanyl}benzyl)urea

2YIX	2.3	MAP kinase 14	YIX	1-ethyl-3-(2-{[3-(1-methylethyl)[1,2,4]triazolo[4,3-a]pyridin-6-yl]sulfanyl}benzyl)urea
2YIY	2.49	MAP kinase kinase kinase 7	YIY	(1E)-1-[5-TERT-BUTYL-2-(3-FLUOROPHENYL)-1H-PYRAZOL-3-YLIDENE]-3-(4-PYRIDIN-3-YLOXYPHENYL)UREA
2YM3	2.007	Serine/threonine-protein kinase Chk1	YM3	ETHYL 4-(2-(AMINOMETHYL)MORPHOLINO)-1H-PYRAZOLO[3,4-B]PYRIDINE-5-CARBOXYLATE
2YM4	2.35	Serine/threonine-protein kinase Chk1	4YM	ethyl 4-[(2R)-2-(aminomethyl)morpholin-4-yl]-3-(3-cyanophenyl)-1H-pyrazolo[3,4-b]pyridine-5-carboxylate
2YM5	2.03	Serine/threonine-protein kinase Chk1	YM5	(3-{4-[(2S)-2-(AMINOMETHYL)MORPHOLIN-4-YL]-7H-PYRROLO[2,3-D]PYRIMIDIN-5-YL}PHENYL)METHANOL
2YM6	2.01	Serine/threonine-protein kinase Chk1	YM6	1-[(2R)-4-(9H-pyrido[4',3':4,5]pyrrolo[2,3-d]pyrimidin-4-yl)morpholin-2-yl]methanamine
2YM7	1.81	Serine/threonine-protein kinase Chk1	YM7	5-({6-[(piperidin-4-ylmethyl)amino]pyrimidin-4-yl}amino)pyrazine-2-carbonitrile
2YM8	2.07	Serine/threonine-protein kinase Chk1	YM8	(R)-5-(8-CHLOROISOQUINOLIN-3-YLAMINO)-3-(1-(DIMETHYLAMINO)PROPAN-2-YLOXY)PYRAZINE-2-CARBONITRILE
2Z2W	2.22	Wee1-like protein kinase	770	N-[4-(2-CHLOROPHENYL)-1,3-DIOXO-1,2,3,6-TETRAHYDROPYRROLO[3,4-C]CARBAZOL-9-YL]FORMAMIDE
2Z60	1.95	Proto-oncogene tyrosine-protein kinase ABL1	P3Y	5-[3-(2-METHOXYPHENYL)-1H-PYRROLO[2,3-B]PYRIDIN-5-YL]-N,N-DIMETHYLPYRIDINE-3-CARBOXAMIDE
2Z7L	2.41	MAP kinase 1	S91	[4-({5-(AMINOCARBONYL)-4-[(3-METHYLPHENYL)AMINO]PYRIMIDIN-2-YL}AMINO)PHENYL]ACETIC ACID
2Z7S	2.1	Ribosomal protein S6 kinase alpha-1	P01	2-({6-[(3-CHLOROPHENYL)AMINO]-9-ISOPROPYL-9H-PURIN-2-YL}AMINO)-3-METHYLBUTAN-1-OL
2ZAZ	1.8	MAP kinase 14	GK1	4-{4-[(5-hydroxy-2-methylphenyl)amino]quinolin-7-yl}-1,3-thiazole-2-carbaldehyde
2ZB0	2.1	MAP kinase 14	GK3	N-(3-cyanophenyl)-2'-methyl-5'-(5-methyl-1,3,4-oxadiazol-2-yl)-4-biphenylcarboxamide
2ZB1	2.5	MAP kinase 14	GK4	N-(cyclopropylmethyl)-2'-methyl-5'-(5-methyl-1,3,4-oxadiazol-2-yl)biphenyl-4-carboxamide
2ZDT	2	MAP kinase 10	46C	4-[(6-chloro-1-oxo-4-phenyl-3-propanoylisoquinolin-2(1H)-yl)methyl]benzoic acid
2ZDU	2.5	MAP kinase 10	446	4-[(4-{[6-bromo-3-(methoxycarbonyl)-1-oxo-4-phenylisoquinolin-2(1H)-yl]methyl}phenyl)amino]-4-oxobutanoic acid
2ZJW	2.4	Casein kinase II	REF	2,3,7,8-tetrahydroxychromeno[5,4,3-cde]chromene-5,10-dione
2ZM1	2.1	Proto-oncogene tyrosine-protein kinase LCK	KSF	N-(2-chlorophenyl)-5-phenylimidazo[1,5-a]pyrazin-8-amine
2ZM3	2.5	Insulin-like growth factor 1 receptor	575	(4Z)-6-bromo-4-({[4-(pyrrolidin-1-ylmethyl)phenyl]amino}methylidene)isoquinoline-1,3(2H,4H)-dione
2ZOQ	2.39	MAP kinase 3	5ID	(2R,3R,4S,5R)-2-(4-AMINO-5-IODO-7H-PYRROLO[2,3-D]PYRIMIDIN-7-YL)-5-(HYDROXYMETHYL)TETRAHYDROFURAN-3,4-DIOL
2ZV2	2.4	Calcium/calmodulin-dependent protein kinase	609	7-oxo-7H-benzimidazo[2,1-a]benz[de]isoquinoline-3-carboxylic acid
3AC1	1.99	Proto-oncogene tyrosine-protein kinase LCK	KZI	5-{[(1R,2S)-2-aminocyclohexyl]amino}-3-[(3,5-dimethoxyphenyl)amino]pyrazine-2-carboxamide
3AC2	2.1	Proto-oncogene tyrosine-protein kinase LCK	KSE	7-[(2-amino-2-methylpropyl)amino]-2-[(3,5-dimethoxyphenyl)amino]-5-methylpyrazolo[1,5-a]pyrimidine-3-carboxamide
3AC5	2.5	Proto-oncogene tyrosine-protein kinase LCK	KZM	5-{[(1R,2S)-2-aminocyclohexyl]amino}-7-[(3,5-dimethoxyphenyl)amino]-2-(3-hydroxyphenyl)[1,2,4]triazolo[1,5-c]pyrimidine-8-carboxamide
3AC8	2.3	Proto-oncogene tyrosine-protein kinase LCK	KSK	7-[(2-amino-2-methylpropyl)amino]-5-cyclopropyl-2-[(3,5-dimethoxyphenyl)amino]pyrazolo[1,5-a]pyrimidine-3-carboxamide
3ACJ	2.2	Proto-oncogene tyrosine-protein kinase LCK	KSS	7-(3,4-dimethoxyphenyl)-5-(ethylsulfanyl)imidazo[1,2-c]pyrimidine
3AD4	2.2	Proto-oncogene tyrosine-protein kinase LCK	KBM	(4-chlorophenyl)(5-methoxy-1-benzofuran-2-yl)methanone
3AD5	2	Proto-oncogene tyrosine-protein kinase LCK	5PB	4-[4-(benzyloxy)phenyl]-5-{[2-(4-chlorophenyl)-2-oxoethyl]sulfanyl}-2,4-dihydro-3H-1,2,4-triazol-3-one
3AD6	2.15	Proto-oncogene tyrosine-protein kinase LCK	KSC	7-[(cyclopropylmethyl)amino]-2-[(4-methoxyphenyl)amino]-5-methylpyrazolo[1,5-a]pyrimidine-3-carboxamide
3AG9	2	cAMP-dependent protein kinase	A02	(10R,20R,23R)-10-(4-aminobutyl)-1-[(2S,3S,4R,5R)-5-(6-amino-9H-purin-9-yl)-3,4-dihydroxytetrahydrofuran-2-yl]-20,23-bis(3-carbamimidamidopropyl)-1,8,11,18,21-pentaoxo-2,9,12,19,22-pentaazatetracosan-24-amide
3AGL	2.1	cAMP-dependent protein kinase	A03	(10R,20R,23R)-1-[(2S,3S,4R,5R)-5-(6-amino-9H-purin-9-yl)-3,4-dihydroxytetrahydrofuran-2-yl]-20,23-bis(3-carbamimidamidopropyl)-10-methyl-1,8,11,18,21-pentaoxo-2,9,12,19,22-pentaazatetracosan-24-amide
3AMA	1.75	cAMP-dependent protein kinase	SKE	4-({5-amino-1-[(2,6-difluorophenyl)carbonyl]-1H-1,2,4-triazol-3-yl}amino)benzenesulfonamide
3AMB	2.25	cAMP-dependent protein kinase	VX6	CYCLOPROPANECARBOXYLIC ACID {4-[4-(4-METHYL-PIPERAZIN-1-YL)-6-(5-METHYL-2H-PYRAZOL-3-YLAMINO)-PYRIMIDIN-2-YLSULFANYL]-PHENYL}-AMIDE
3AMY	2.3	Casein kinase II	AGI	5,7-dihydroxy-2-(4-hydroxyphenyl)-4H-chromen-4-one
3AQV	2.08	5'-AMP-activated protein kinase	TAK	6-[4-(2-piperidin-1-ylethoxy)phenyl]-3-pyridin-4-ylpyrazolo[1,5-a]pyrimidine
3AT4	2.2	Casein kinase II	CCK	[1-(6-{6-[(1-methylethyl)amino]-1H-indazol-1-yl}pyrazin-2-yl)-1H-pyrrol-3-yl]acetic acid
3AXW	2.5	Casein kinase II	TID	4-(5-amino-1,3,4-thiadiazol-2-yl)benzoic acid
3B2T	1.8	Fibroblast growth factor receptor 2	M33	5'-O-[(S)-hydroxy{[(S)-hydroxy(methyl)phosphoryl]oxy}phosphoryl]adenosine
3B2W	2.3	Proto-oncogene tyrosine-protein kinase LCK	9NH	N-[5-({[2-fluoro-3-(trifluoromethyl)phenyl]amino}carbonyl)-2-methylphenyl]-4-methoxy-2-[(4-piperazin-1-ylphenyl)amino]pyrimidine-5-carboxamide
3BE9	2	Casein kinase II	P04	19-(cyclopropylamino)-4,6,7,15-tetrahydro-5H-16,1-(azenometheno)-10,14-(metheno)pyrazolo[4,3-o][1,3,9]triazacyclohexadecin-8(9H)-one
3BEA	2.02	Macrophage colony-stimulating factor 1 receptor	IXH	8-(2,3-dihydro-1H-inden-5-yl)-2-({4-[(3R,5S)-3,5-dimethylpiperazin-1-yl]phenyl}amino)-5-oxo-5,8-dihydropyrido[2,3-d]pyrimidine-6-carboxamide
3BEL	2.3	Epidermal growth factor receptor	POX	4-amino-6-{[1-(3-fluorobenzyl)-1H-indazol-5-yl]amino}pyrimidine-5-carbaldehyde O-(2-methoxyethyl)oxime
3BGQ	2	Proto-oncogene serine/threonine-protein kinase	VX2	N-cyclohexyl-3-[3-(trifluoromethyl)phenyl][1,2,4]triazolo[4,3-b]pyridazin-6-amine
3BGZ	2.4	Proto-oncogene serine/threonine-protein kinase	VX3	2,3-diphenyl-1H-indole-7-carboxylic acid
3BHH	2.4	Calcium/calmodulin-dependent protein kinase	5CP	[4-({4-[(5-cyclopropyl-1H-pyrazol-3-yl)amino]-6-(methylamino)pyrimidin-2-yl}amino)phenyl]acetonitrile
3BHT	2	Cyclin-dependent kinase 2	MFR	4-(4-methoxy-1H-pyrrolo[2,3-b]pyridin-3-yl)pyrimidin-2-amine
3BHU	2.3	Cyclin-dependent kinase 2	MHR	4-(4-propoxy-1H-pyrrolo[2,3-b]pyridin-3-yl)pyrimidin-2-amine
3BHV	2.1	Cyclin-dependent kinase 2	VAR	9-amino-5-(2-aminopyrimidin-4-yl)pyrido[3',2':4,5]pyrrolo[1,2-c]pyrimidin-4-ol
3BI6	2.2	Wee1-like protein kinase	396	4-(2-chlorophenyl)-9-hydroxy-6-methyl-1,3-dioxo-N-(2-pyrrolidin-1-ylethyl)pyrrolo[3,4-g]carbazole-8-carboxamide
3BIZ	2.2	Wee1-like protein kinase	61E	4-(2-chlorophenyl)-8-[3-(dimethylamino)propoxy]-9-hydroxy-6-methylpyrrolo[3,4-c]carbazole-1,3(2H,6H)-dione
3BQR	1.75	Death-associated protein kinase 3	4RB	4-(6-{[(1R)-1-(hydroxymethyl)propyl]amino}imidazo[1,2-b]pyridazin-3-yl)benzoic acid
3BV2	2.4	MAP kinase 14	P38	5-methyl-4-[(2-methyl-5-{[(2-morpholin-4-ylpyridin-4-yl)carbonyl]amino}phenyl)amino]-N-(1-phenylethenyl)pyrrolo[2,1-f][1,2,4]triazine-6-carboxamide

3BWJ	2.3	cAMP-dependent protein kinase	ARX	(2S,3S,4R,5R)-5-(6-amino-9H-purin-9-yl)-N-(6-{[(1R)-4-carbamimidamido-1-{[(1R)-4-carbamimidamido-1-carbamoylbutyl]carbamoyl}butyl]amino}-6-oxohexyl)-3,4-dihydroxytetrahydrofuran-2-carboxamide
3BX5	2.4	MAP kinase 14	304	N-[2-methyl-5-(methylcarbamoyl)phenyl]-2-{[(1R)-1-methylpropyl]amino}-1,3-thiazole-5-carboxamide
3BYM	2	Proto-oncogene tyrosine-protein kinase LCK	AM0	N-phenyl-1-{4-[(3,4,5-trimethoxyphenyl)amino]-1,3,5-triazin-2-yl}-1H-benzimidazol-2-amine
3BYO	2	Proto-oncogene tyrosine-protein kinase LCK	AM9	6-(2,6-dimethylphenyl)-2-{[4-(4-methylpiperazin-1-yl)phenyl]amino}pyrimido[5',4':5,6]pyrimido[1,2-a]benzimidazol-5(6H)-one
3BYS	2.2	Proto-oncogene tyrosine-protein kinase LCK	AM5	4-methyl-N~3~-(2-{[4-(4-methylpiperazin-1-yl)phenyl]amino}pyrimidin-5-yl)-N~1~-[3-(trifluoromethyl)phenyl]benzene-1,3-dicarboxamide
3BYU	2.3	Proto-oncogene tyrosine-protein kinase LCK	AM6	2-methyl-N-{4-methyl-3-[(2-{[4-(4-methylpiperazin-1-yl)phenyl]amino}pyrimidin-5-yl)carbamoyl]phenyl}-3-(trifluoromethyl)benzamide
3BZ3	2.2	Focal adhesion kinase 1	YAM	N-methyl-N-{3-[({2-[(2-oxo-2,3-dihydro-1H-indol-5-yl)amino]-5-(trifluoromethyl)pyrimidin-4-yl}amino)methyl]pyridin-2-yl}methanesulfonamide
3C0I	1.85	Peripheral plasma membrane protein CASK	3AM	[(2R,3S,4R,5R)-5-(6-aminopurin-9-yl)-4-hydroxy-2-(hydroxymethyl)oxolan-3-yl] dihydrogen phosphate
3C1X	2.17	Hepatocyte growth factor receptor	CKK	N-{[4-({5-[(4-aminopiperidin-1-yl)methyl]pyrrolo[2,1-f][1,2,4]triazin-4-yl}oxy)-3-fluorophenyl]carbamoyl}-2-(4-fluorophenyl)acetamide
3C4E	1.98	Proto-oncogene serine/threonine-protein kinase	C4E	N-phenyl-1H-pyrrolo[2,3-b]pyridin-3-amine
3C4F	2.07	Basic fibroblast growth factor receptor 1	C4F	3-(3-methoxybenzyl)-1H-pyrrolo[2,3-b]pyridine
3CCN	1.9	Hepatocyte growth factor receptor	LKG	4-[(6-phenyl[1,2,4]triazolo[4,3-b]pyridazin-3-yl)methyl]phenol
3CD8	2	Hepatocyte growth factor receptor	L5G	7-methoxy-4-[(6-phenyl[1,2,4]triazolo[4,3-b]pyridazin-3-yl)methoxy]quinoline
3CE3	2.4	Hepatocyte growth factor receptor	1FN	1-(4-fluorophenyl)-N-[3-fluoro-4-(1H-pyrrolo[2,3-b]pyridin-4-yloxy)phenyl]-2-oxo-1,2-dihydropyridine-3-carboxamide
3CJF	2.15	Vascular endothelial growth factor receptor 2	SAV	N~4~-(3-methyl-1H-indazol-6-yl)-N~2~-(3,4,5-trimethoxyphenyl)pyrimidine-2,4-diamine
3CJG	2.25	Vascular endothelial growth factor receptor 2	KIM	N~4~-methyl-N~4~-(3-methyl-1H-indazol-6-yl)-N~2~-(3,4,5-trimethoxyphenyl)pyrimidine-2,4-diamine
3CP9	2.5	Vascular endothelial growth factor receptor 2	C19	3-(2-aminoquinazolin-6-yl)-1-(3,3-dimethylindolin-6-yl)-4-methylpyridin-2(1H)-one
3CPC	2.4	Vascular endothelial growth factor receptor 2	C52	3-(2-aminoquinazolin-6-yl)-4-methyl-1-[3-(trifluoromethyl)phenyl]pyridin-2(1H)-one
3CQE	2.5	Wee1-like protein kinase	P91	8-bromo-4-(2-chlorophenyl)-N-(2-hydroxyethyl)-6-methyl-1,3-dioxo-1,2,3,6-tetrahydropyrrolo[3,4-e]indole-7-carboxamide
3CQU	2.2	RAC-alpha serine/threonine-protein kinase	CQU	N-[2-(5-methyl-4H-1,2,4-triazol-3-yl)phenyl]-7H-pyrrolo[2,3-d]pyrimidin-4-amine
3CQW	2	Glycogen synthase kinase-3 beta	CQW	5-(5-chloro-7H-pyrrolo[2,3-d]pyrimidin-4-yl)-4,5,6,7-tetrahydro-1H-imidazo[4,5-c]pyridine
3CR0	2.3	Wee1-like protein kinase	809	4-(2-chlorophenyl)-8-(2-hydroxyethyl)-6-methylpyrrolo[3,4-e]indole-1,3(2H,6H)-dione
3CTH	2.3	Hepatocyte growth factor receptor	319	N-({4-[(2-aminopyridin-4-yl)oxy]-3-fluorophenyl}carbamoyl)-2-(4-fluorophenyl)acetamide
3CTJ	2.5	Hepatocyte growth factor receptor	320	2-(4-fluorophenyl)-N-{[3-fluoro-4-(1H-pyrrolo[2,3-b]pyridin-4-yloxy)phenyl]carbamoyl}acetamide
3CTQ	1.95	MAP kinase 14	337	N-benzyl-1-[5-({5-tert-butyl-2-methoxy-3-[(methylsulfonyl)amino]phenyl}carbamoyl)-2-methylphenyl]-1H-1,2,3-triazole-4-carboxamide
3CXW	2.1	Proto-oncogene serine/threonine-protein kinase	7CP	(4R)-7,8-dichloro-1',9-dimethyl-1-oxo-1,2,4,9-tetrahydrospiro[beta-carboline-3,4'-piperidine]-4-carbonitrile
3CY2	2.01	Proto-oncogene serine/threonine-protein kinase	MB9	(4R)-7-chloro-9-methyl-1-oxo-1,2,4,9-tetrahydrospiro[beta-carboline-3,4'-piperidine]-4-carbonitrile
3CY3	2.15	Proto-oncogene serine/threonine-protein kinase	JN5	(2S)-1,3-benzothiazol-2-yl{2-[(2-pyridin-3-ylethyl)amino]pyrimidin-4-yl}ethanenitrile
3D0E	2	RAC-beta serine/threonine-protein kinase	G93	4-{2-(4-amino-1,2,5-oxadiazol-3-yl)-1-ethyl-7-[(3S)-piperidin-3-ylmethoxy]-1H-imidazo[4,5-c]pyridin-4-yl}-2-methylbut-3-yn-2-ol
3D14	1.9	serine/threonine kinase 6	AK1	1-{5-[2-(thieno[3,2-d]pyrimidin-4-ylamino)ethyl]-1,3-thiazol-2-yl}-3-[3-(trifluoromethyl)phenyl]urea
3D15	2.3	serine/threonine kinase 6	AK2	1-(3-chlorophenyl)-3-{5-[2-(thieno[3,2-d]pyrimidin-4-ylamino)ethyl]-1,3-thiazol-2-yl}urea
3D2K	2.5	serine/threonine kinase 6	AK4	(7-{[2-(2-{[(3-chlorophenyl)carbamoyl]amino}-1,3-thiazol-5-yl)ethyl]amino}-1H-pyrazolo[4,3-d]pyrimidin-1-yl)acetic acid
3D7Z	2.1	MAP kinase 14	GK5	N~3~-cyclopropyl-N~4~'-(cyclopropylmethyl)-6-methylbiphenyl-3,4'-dicarboxamide
3D83	1.9	MAP kinase 14	GK6	N-{4'-[(cyclopropylmethyl)carbamoyl]-6-methylbiphenyl-3-yl}-2-morpholin-4-ylpyridine-4-carboxamide
3D94	2.3	Insulin-like growth factor 1 receptor	D94	3-[cis-3-(4-methylpiperazin-1-yl)cyclobutyl]-1-(2-phenylquinolin-7-yl)imidazo[1,5-a]pyrazin-8-amine
3DA6	2	MAP kinase 10	BZ9	N-[3-methyl-4-({3-[2-(methylamino)pyrimidin-4-yl]pyridin-2-yl}oxy)naphthalen-1-yl]-1H-benzimidazol-2-amine
3DAJ	2	serine/threonine kinase 6	FXG	N-butyl-3-{[6-(9H-purin-6-ylamino)hexanoyl]amino}benzamide
3DDQ	1.8	Cyclin-dependent kinase 2	RRC	R-ROSCOVITINE
3DJ5	1.8	serine/threonine kinase 6	AK5	3-({3-[(6-amino-5-bromopyrimidin-4-yl)sulfanyl]propanoyl}amino)-4-methoxy-N-phenylbenzamide
3DJ6	1.7	serine/threonine kinase 6	AK6	4-methoxy-N-phenyl-3-({3-[(1H-pyrrolo[2,3-b]pyridin-5-ylmethyl)sulfanyl]propanoyl}amino)benzamide
3DK3	2.02	Proto-oncogene tyrosine-protein kinase ABL1	SX7	2-amino-5-[3-(1-ethyl-1H-pyrazol-5-yl)-1H-pyrrolo[2,3-b]pyridin-5-yl]-N,N-dimethylbenzamide
3DKF	1.8	Hepatocyte growth factor receptor	SX8	6-{[6-(1-methyl-1H-pyrazol-4-yl)[1,2,4]triazolo[4,3-b]pyridazin-3-yl]sulfanyl}quinoline
3DKO	2	Ephrin type-A receptor 7	IHZ	5-[(2-methyl-5-{[3-(trifluoromethyl)phenyl]carbamoyl}phenyl)amino]pyridine-3-carboxamide
3DND	2.26	cAMP-dependent protein kinase	LL2	5-benzyl-1,3-thiazol-2-amine
3DNE	2	cAMP-dependent protein kinase	LL1	3-pyridin-4-yl-1H-indazole
3DPK	1.95	Macrophage colony-stimulating factor 1 receptor	8C5	8-cyclohexyl-N-methoxy-5-oxo-2-{[4-(2-pyrrolidin-1-ylethyl)phenyl]amino}-5,8-dihydropyrido[2,3-d]pyrimidine-6-carboxamide
3DU8	2.2	Glycogen synthase kinase-3 beta	553	(7S)-2-(2-aminopyrimidin-4-yl)-7-(2-fluoroethyl)-1,5,6,7-tetrahydro-4H-pyrrolo[3,2-c]pyridin-4-one
3DZQ	1.75	EPH receptor A3	IFC	N-[2-methyl-5-({[3-(4-methyl-1H-imidazol-1-yl)-5-(trifluoromethyl)phenyl]carbonyl}amino)phenyl]isoxazole-5-carboxamide
3E62	1.922	Tyrosine-protein kinase JAK2	5B1	5-bromo-1H-indazol-3-amine
3E63	1.9	Tyrosine-protein kinase JAK2	5B2	5-phenyl-1H-indazol-3-amine
3E64	1.8	Tyrosine-protein kinase JAK2	5B3	4-(3-amino-1H-indazol-5-yl)-N-tert-butylbenzenesulfonamide
3E7O	2.14	MAP kinase 9	35F	N-{3-[5-(1H-1,2,4-triazol-3-yl)-1H-indazol-3-yl]phenyl}furan-2-carboxamide
3E7V	2	Serine/threonine-protein kinase haspin	DZO	3-(3-aminophenyl)-N-(3-chlorophenyl)pyrazolo[1,5-a]pyrimidin-5-amine

3E87	2.3	Glycogen synthase kinase-3 beta	G95	N-[(1S)-2-amino-1-phenylethyl]-5-(1H-pyrrolo[2,3-b]pyridin-4-yl)thiophene-2-carboxamide
3E88	2.5	RAC-beta serine/threonine-protein kinase	G96	4-[2-(4-amino-1,2,5-oxadiazol-3-yl)-6-{[(2R)-2-amino-3-phenylpropyl]oxy}-1-ethyl-1H-imidazo[4,5-c]pyridin-4-yl]-2-methylbut-3-yn-2-ol
3E8E	2	cAMP-dependent protein kinase	G98	4-[2-(4-amino-2,5-dihydro-1,2,5-oxadiazol-3-yl)-6-{[(1S)-3-amino-1-phenylpropyl]oxy}-1-ethyl-1H-imidazo[4,5-c]pyridin-4-yl]-2-methylbut-3-yn-2-ol
3E92	2	MAP kinase 14	G6A	N-cyclopropyl-2',6-dimethyl-4'-(5-methyl-1,3,4-oxadiazol-2-yl)biphenyl-3-carboxamide
3E93	2	MAP kinase 14	19B	4-methyl-N-(3-morpholin-4-ylphenyl)-3-(3-piperidin-4-yl-1,2-benzisoxazol-6-yl)benzamide
3EFK	2.2	Hepatocyte growth factor receptor	MT4	5-{4-[(6,7-dimethoxyquinolin-4-yl)oxy]-3-fluorophenyl}-2-[(4-fluorophenyl)amino]-3-methylpyrimidin-4(3H)-one
3EFL	2.2	Vascular endothelial growth factor receptor 2	706	N-(3,3-dimethyl-2,3-dihydro-1H-indol-6-yl)-2-[(pyridin-4-ylmethyl)amino]pyridine-3-carboxamide
3EFW	2.29	Serine/threonine-protein kinase 6	AK8	1-[3-methyl-4-({3-[2-(methylamino)pyrimidin-4-yl]pyridin-2-yl}oxy)phenyl]-3-[3-(trifluoromethyl)phenyl]urea
3EKK	2.1	Insulin receptor	GS2	2-[(2-{[1-(N,N-dimethylglycyl)-5-methoxy-1H-indol-6-yl]amino}-7H-pyrrolo[2,3-d]pyrimidin-4-yl)amino]-6-fluoro-N-methylbenzamide
3EKN	2.2	Insulin receptor	GS3	2-fluoro-6-{[2-({2-methoxy-4-[4-(1-methylethyl)piperazin-1-yl]phenyl}amino)-7H-pyrrolo[2,3-d]pyrimidin-4-yl]amino}benzamide
3EL8	2.3	Proto-oncogene tyrosine-protein kinase Src	PD5	1-{4-[4-amino-1-(1-methylethyl)-1H-pyrazolo[3,4-d]pyrimidin-3-yl]phenyl}-3-[3-(trifluoromethyl)phenyl]urea
3ELJ	1.8	MAP kinase 8	GS7	2-fluoro-6-{[2-({2-methoxy-4-[(methylsulfonyl)methyl]phenyl}amino)-7H-pyrrolo[2,3-d]pyrimidin-4-yl]amino}benzamide
3EN6	2.39	Proto-oncogene tyrosine-protein kinase Src	KS5	1-(1-methylethyl)-3-quinolin-6-yl-1H-pyrazolo[3,4-d]pyrimidin-4-amine
3ENE	2.4	Phosphatidylinositol 4,5-bisphosphate 3-kinase	NPZ	1-methyl-3-naphthalen-2-yl-1H-pyrazolo[3,4-d]pyrimidin-4-amine
3EQC	1.8	Dual specificity MAP kinase kinase 1	3BM	2-[(2-chloro-4-iodophenyl)amino]-N-{[(2R)-2,3-dihydroxypropyl]oxy}-3,4-difluorobenzamide
3EQH	2	Dual specificity MAP kinase kinase 1	5BM	(2Z)-bis{amino[(2-aminophenyl)sulfanyl]methylidene}butanedinitrile
3EQP	2.3	Activated CDC42 kinase 1	T95	N-(2,6-dimethylphenyl)-4-(2-ethoxyphenoxy)-2-({4-[4-(2-hydroxyethyl)piperazin-1-yl]phenyl}amino)pyrimidine-5-carboxamide
3EQR	2	Activated CDC42 kinase 1	T74	N~3~-(2,6-dimethylphenyl)-1-(3-methoxy-3-methylbutyl)-N~6~-(4-piperazin-1-ylphenyl)-1H-pyrazolo[3,4-d]pyrimidine-3,6-diamine
3EWH	1.6	vascular endothelial growth factor receptor 2	K11	N-[4-({3-[2-(methylamino)pyrimidin-4-yl]pyridin-2-yl}oxy)naphthalen-1-yl]-6-(trifluoromethyl)-1H-benzimidazol-2-amine
3EYG	1.9	Tyrosine-protein kinase	MI1	3-{(3R,4R)-4-methyl-3-[methyl(7H-pyrrolo[2,3-d]pyrimidin-4-yl)amino]piperidin-1-yl}-3-oxopropanenitrile
3EZR	1.9	Cyclin-dependent kinase 2	EZR	3-methoxy-4-{3-[4-(4-methylpiperazin-1-yl)-1H-benzimidazol-2-yl]-1H-indazol-6-yl}aniline
3EZV	1.99	Cyclin-dependent kinase 2	EZV	4-{3-[7-(4-methylpiperazin-1-yl)-1H-benzimidazol-2-yl]-1H-indazol-6-yl}aniline
3F2A	1.9	Proto-oncogene serine/threonine-protein kinase	985	(2E)-3-{3-[6-(4-methyl-1,4-diazepan-1-yl)pyrazin-2-yl]phenyl}prop-2-enoic acid
3F2N	1.8	Serine/threonine-protein kinase haspin	IZZ	(2S)-2-{[3-(3-aminophenyl)imidazo[1,2-b]pyridazin-6-yl]amino}-3-methylbutan-1-ol
3F3T	2.5	Proto-oncogene tyrosine-protein kinase Src	1AU	1-[1-(3-aminophenyl)-3-tert-butyl-1H-pyrazol-5-yl]-3-naphthalen-1-ylurea
3F3U	2.5	Proto-oncogene tyrosine-protein kinase Src	1AW	1-[1-(3-aminophenyl)-3-tert-butyl-1H-pyrazol-5-yl]-3-phenylurea
3F3Z	1.84	Calcium/calmodulin-dependent protein kinase	DRK	3-({[(3S)-3,4-dihydroxybutyl]oxy}amino)-1H,2'H-2,3'-biindol-2'-one
3F66	1.4	Hepatocyte growth factor receptor	IHX	3-[3-(4-methylpiperazin-1-yl)-7-(trifluoromethyl)quinoxalin-5-yl]phenol
3F6X	2.35	Proto-oncogene tyrosine-protein kinase Src	IHH	[4-({4-[(5-cyclopropyl-1H-pyrazol-3-yl)amino]quinazolin-2-yl}amino)phenyl]acetonitrile
3F7Z	2.4	Glycogen synthase kinase-3 beta	34O	2-(1,3-benzodioxol-5-yl)-5-[(3-fluoro-4-methoxybenzyl)sulfanyl]-1,3,4-oxadiazole
3F82	2.5	hepatocyte growth factor receptor	353	N-{4-[(2-amino-3-chloropyridin-4-yl)oxy]-3-fluorophenyl}-4-ethoxy-1-(4-fluorophenyl)-2-oxo-1,2-dihydropyridine-3-carboxamide
3FC2	2.45	Serine/threonine-protein kinase PLK1	IBI	N-{trans-4-[4-(cyclopropylmethyl)piperazin-1-yl]cyclohexyl}-4-{[(7R)-7-ethyl-5-methyl-8-(1-methylethyl)-6-oxo-5,6,7,8-tetrahydropteridin-2-yl]amino}-3-methoxybenzamide
3FDN	1.9	Serine/threonine-protein kinase 6	MMH	N-[3-(acetylamino)phenyl]-5-{(2E)-2-[(4-methoxyphenyl)methylidene]hydrazino}-3-methyl-1H-pyrazole-4-carboxamide
3FHR	1.9	MAP kinase-activated protein kinase 3	P4O	2-(2-QUINOLIN-3-YLPYRIDIN-4-YL)-1,5,6,7-TETRAHYDRO-4H-PYRROLO[3,2-C]PYRIDIN-4-ONE
3FI2	2.28	MAP kinase 10	JK1	3-{4-[(phenylcarbamoyl)amino]-1H-pyrazol-1-yl}-N-(3,4,5-trimethoxyphenyl)benzamide
3FI3	2.2	MAP kinase 10	JK2	3-{5-[(2-fluorophenyl)amino]-1H-indazol-1-yl}-N-(3,4,5-trimethoxyphenyl)benzamide
3FI4	2.2	MAP kinase 14	FI4	(2S)-1-{[3-(2-chlorophenyl)-6-(2,4-difluorophenoxy)-1H-pyrazolo[3,4-d]pyrimidin-4-yl]amino}propan-2-ol
3FKL	2	MAP kinase 14	FKL	N-cyclopropyl-4-methyl-3-[8-methyl-7-oxo-2-(tetrahydro-2H-pyran-4-ylamino)-7,8-dihydropyrido[2,3-d]pyrimidin-6-yl]benzamide
3FKN	2	MAP kinase 14	FKN	3-[2-chloro-5-(methylsulfonyl)phenyl]-6-(2,4-difluorophenoxy)-1H-pyrazolo[3,4-d]pyrimidine
3FKO	2	MAP kinase 14	FKO	3-(2-chlorophenyl)-6-(2-fluorophenoxy)-2H-indazole
3FL4	1.8	MAP kinase 14	FL4	6-(2,4-difluorophenoxy)-3-(2-methylphenyl)-1H-pyrazolo[3,4-d]pyrimidine
3FLN	1.9	MAP kinase 14	3FN	6-(2,4-difluorophenoxy)-8-methyl-2-(tetrahydro-2H-pyran-4-ylamino)pyrido[2,3-d]pyrimidin-7(8H)-one
3FLQ	1.9	MAP kinase 14	891	6-(2,4-difluorophenoxy)-8-methyl-2-{[(1S)-1-methyl-2-(methylsulfonyl)ethyl]amino}pyrido[2,3-d]pyrimidin-7(8H)-one
3FLS	2.3	MAP kinase 14	FLS	6-(2,4-difluorophenoxy)-8-methyl-2-{[(1R)-1-methyl-2-(methylsulfonyl)ethyl]amino}pyrido[2,3-d]pyrimidin-7(8H)-one
3FLW	2.1	MAP kinase 14	FLW	6-(2,4-difluorophenoxy)-2-{[3-hydroxy-1-(2-hydroxyethyl)propyl]amino}-8-methylpyrido[2,3-d]pyrimidin-7(8H)-one
3FLY	1.8	MAP kinase 14	FLY	6-(2,4-difluorophenoxy)-8-methyl-2-[(1-methylethyl)amino]pyrido[2,3-d]pyrimidin-7(8H)-one
3FLZ	2.23	MAP kinase 14	FLZ	8-methyl-6-phenoxy-2-(tetrahydro-2H-pyran-4-ylamino)pyrido[2,3-d]pyrimidin-7(8H)-one
3FMD	2	Serine/threonine-protein kinase haspin	IQB	N-[2-(4-BROMOCINNAMYLAMINO)ETHYL]-5-ISOQUINOLINE SULFONAMIDE
3FMH	1.9	MAP kinase 14	533	6-(2,4-difluorophenoxy)-8-methyl-2-{[(1R)-1-methyl-2-(2H-tetrazol-2-yl)ethyl]amino}pyrido[2,3-d]pyrimidin-7(8H)-one
3FMJ	2	MAP kinase 14	FMJ	4-(5-methyl-3-phenylisoxazol-4-yl)pyrimidin-2-amine
3FMK	1.7	MAP kinase 14	FMK	6-(2,4-difluorophenoxy)-8-methyl-2-{[(1S)-1-methyl-2-(2H-tetrazol-2-yl)ethyl]amino}pyrido[2,3-d]pyrimidin-7(8H)-one
3FML	2.1	MAP kinase 14	FML	6-(2,4-difluorophenoxy)-N-[(1S)-1-methyl-2-(methylsulfonyl)ethyl]-1H-pyrazolo[3,4-d]pyrimidin-3-amine
3FMM	2	MAP kinase 14	XI2	6-(2,4-difluorophenoxy)-N-[(1R)-1-methyl-2-(methylsulfonyl)ethyl]-1H-pyrazolo[3,4-d]pyrimidin-3-amine

3FMN	1.9	MAP kinase 14	530	6-(2,4-difluorophenoxy)-N-(tetrahydro-2H-pyran-4-yl)-1H-pyrazolo[3,4-d]pyrimidin-3-amine
3FQE	2.5	Tyrosine-protein kinase SYK	P5C	2-{[(1R,2S)-2-aminocyclohexyl]amino}-4-[(3-methylphenyl)amino]pyrimidine-5-carboxamide
3FQH	2.26	Tyrosine-protein kinase SYK	057	N-(2-hydroxy-1,1-dimethylethyl)-1-methyl-3-(1H-pyrrolo[2,3-b]pyridin-2-yl)-1H-indole-5-carboxamide
3FQS	2.1	Tyrosine-protein kinase SYK	585	6-({5-fluoro-2-[(3,4,5-trimethoxyphenyl)amino]pyrimidin-4-yl}amino)-2,2-dimethyl-2H-pyrido[3,2-b][1,4]oxazin-3(4H)-one
3FSF	2.1	MAP kinase 14	FSS	3-(2,6-dichlorophenyl)-7-({4-[(diethylamino)methoxy]phenyl}amino)-1-methyl-3,4-dihydropyrimido[4,5-d]pyrimidin-2(1H)-one
3FSK	2	MAP kinase 14	RO6	3-(2-chlorophenyl)-7-[(trans-4-hydroxycyclohexyl)amino]-3,4-dihydropyrimido[4,5-d]pyrimidin-2(1H)-one
3FV8	2.28	MAP kinase 10	JK3	5-bromo-N-(3-chloro-2-(4-(prop-2-ynyl)piperazin-1-yl)phenyl)furan-2-carboxamide
3FXW	2	MAP kinase-activated protein kinase 3	F10	2-[2-(2-FLUOROPHENYL)PYRIDIN-4-YL]-1,5,6,7-TETRAHYDRO-4H-PYRROLO[3,2-C]PYRIDIN-4-ONE
3FXZ	1.64	Serine/threonine-protein kinase PAK 1	FLL	OCTAHEDRAL RU-PYRIDOCARBAZOLE
3FZ1	1.9	Cyclin-dependent kinase 2	B98	(3R)-3-(aminomethyl)-9-methoxy-1,2,3,4-tetrahydro-5H-[1]benzothieno[3,2-e][1,4]diazepin-5-one
3FZT	1.95	Protein tyrosine kinase 2 beta	4JZ	1-[5-tert-butyl-2-(4-methylphenyl)-1,2-dihydro-3H-pyrazol-3-ylidene]-3-{3-[(pyridin-3-yloxy)methyl]-1H-pyrazol-5-yl}urea
3G5D	2.2	Proto-oncogene tyrosine-protein kinase Src	1N1	N-(2-CHLORO-6-METHYLPHENYL)-2-({6-[4-(2-HYDROXYETHYL)PIPERAZIN-1-YL]-2-METHYLPYRIMIDIN-4-YL}AMINO)-1,3-THIAZOLE-5-CARBOXAMIDE
3G6G	2.31	Proto-oncogene tyrosine-protein kinase Src	G6G	N-{3-[(3-{4-[(4-methoxyphenyl)amino]-1,3,5-triazin-2-yl}pyridin-2-yl)amino]-4-methylphenyl}-4-[(4-methylpiperazin-1-yl)methyl]benzamide
3G6H	2.35	Proto-oncogene tyrosine-protein kinase Src	G6H	N-{4-methyl-3-[(3-{4-[(3,4,5-trimethoxyphenyl)amino]-1,3,5-triazin-2-yl}pyridin-2-yl)amino]phenyl}-3-(trifluoromethyl)benzamide
3G90	2.4	MAP kinase 10	J72	(3E)-5-fluoro-1-[(6-fluoro-4H-1,3-benzodioxin-8-yl)methyl]-1H-indole-2,3-dione 3-oxime
3G9L	2.2	MAP kinase 10	J67	(3Z)-1-[(6-fluoro-4H-1,3-benzodioxin-8-yl)methyl]-4-[(E)-2-phenylethenyl]-1H-indole-2,3-dione 3-oxime
3GB2	2.4	Glycogen synthase kinase-3 beta	G3B	2-methyl-5-(3-{4-[(S)-methylsulfinyl]phenyl}-1-benzofuran-5-yl)-1,3,4-oxadiazole
3GC7	1.8	MAP kinase 14	B45	5-(2-chloro-4-fluorophenyl)-1-(2,6-dichlorophenyl)-7-[1-(1-methylethyl)piperidin-4-yl]-3,4-dihydroquinazolin-2(1H)-one
3GCQ	2	MAP kinase 14	1BU	1-{4-[(6-aminoquinazolin-4-yl)amino]phenyl}-3-[3-tert-butyl-1-(3-methylphenyl)-1H-pyrazol-5-yl]urea
3GCS	2.1	MAP kinase 14	BAX	4-{4-[({[4-CHLORO-3-(TRIFLUOROMETHYL)PHENYL]AMINO}CARBONYL)AMINO]PHENOXY}-N-METHYLPYRIDINE-2-CARBOXAMIDE
3GCU	2.1	MAP kinase 14	R48	1-{3-[(6-aminoquinazolin-4-yl)amino]phenyl}-3-[3-tert-butyl-1-(4-methylphenyl)-1H-pyrazol-5-yl]urea
3GCV	2.3	MAP kinase 14	SS6	1-{3-[(6-aminoquinazolin-4-yl)amino]phenyl}-3-[3-tert-butyl-1-(3-methylphenyl)-1H-pyrazol-5-yl]urea
3GEN	1.6	Tyrosine-protein kinase BTK	B43	4-Amino-5-(4-phenoxyphenyl)-7H-pyrrolo[2,3-d]pyrimidin-7-yl-cyclopentane
3GEQ	2.2	Proto-oncogene tyrosine-protein kinase Src	PP2	1-TERT-BUTYL-3-(4-CHLORO-PHENYL)-1H-PYRAZOLO[3,4-D]PYRIMIDIN-4-YLAMINE
3GFE	2.1	MAP kinase 14	P37	N-cyclopropyl-3-{[1-(2,4-difluorophenyl)-7-methyl-6-oxo-6,7-dihydro-1H-pyrazolo[3,4-b]pyridin-4-yl]amino}-4-methylbenzamide
3GI3	2.4	MAP kinase 14	B10	N-{5-tert-butyl-2-methoxy-3-[({4-[6-(morpholin-4-ylmethyl)pyridin-3-yl]naphthalen-1-yl}carbamoyl)amino]phenyl}methanesulfonamide
3GP0	1.9	MAP kinase 11	NIL	Nilotinib
3GU8	1.6	Death-associated protein kinase 1	3GU	N6-cyclopentyladenosine
3GUB	1.71	Death-associated protein kinase 1	GUB	9-alpha-L-lyxofuranosyl-N-(2-phenylethyl)-9H-purin-6-amine
3GXL	1.8	TGF-beta receptor type-1	QIG	N-1H-indazol-5-yl-2-(6-methylpyridin-2-yl)quinazolin-4-amine
3H0Y	2.5	Serine/threonine-protein kinase 6	48B	2-chloro-N-[4-({5-fluoro-2-[(4-hydroxyphenyl)amino]pyrimidin-4-yl}amino)phenyl]benzamide
3H10	2.2	Serine/threonine-protein kinase 6	97B	9-chloro-7-(2,6-difluorophenyl)-N-{4-[(4-methylpiperazin-1-yl)carbonyl]phenyl}-5H-pyrimido[5,4-d][2]benzazepin-2-amine
3H3C	2	Protein tyrosine kinase 2 beta	P1E	4-{[4-{[(1R,2R)-2-(dimethylamino)cyclopentyl]amino}-5-(trifluoromethyl)pyrimidin-2-yl]amino}-N-methylbenzenesulfonamide
3H9O	2.3	3-phosphoinositide-dependent protein kinase 1	9BD	2-(1H-imidazol-1-yl)-9-methoxy-8-(2-methoxyethoxy)benzo[c][2,7]naphthyridin-4-amine
3HA6	2.36	Serine/threonine-protein kinase 6	2JZ	N~2~-(3,4-dimethoxyphenyl)-N~4~-[2-(2-fluorophenyl)ethyl]-N~6~-quinolin-6-yl-1,3,5-triazine-2,4,6-triamine
3HA8	2.48	MAP kinase 14	5JZ	N~2~-{4-[6-(3,4-dihydroquinolin-1(2H)-ylcarbonyl)-1H-benzimidazol-1-yl]-6-ethoxy-1,3,5-triazin-2-yl}-3-(2,2-dimethyl-4H-1,3-benzodioxin-6-yl)-N-methyl-L-alaninamide
3HL7	1.88	MAP kinase 14	I47	2-{4-[5-(4-chlorophenyl)-4-pyrimidin-4-yl-1H-pyrazol-3-yl]piperidin-1-yl}-2-oxoethanol
3HLL	1.95	MAP kinase 14	I45	3-{3-bromo-4-[(2,4-difluorobenzyl)oxy]-6-methyl-2-oxopyridin-1(2H)-yl}-N,4-dimethylbenzamide
3HMI	1.65	Tyrosine-protein kinase ABL2	DKI	5-AMINO-3-{[4-(AMINOSULFONYL)PHENYL]AMINO}-N-(2,6-DIFLUOROPHENYL)-1H-1,2,4-TRIAZOLE-1-CARBOTHIOAMIDE
3HMM	1.7	TGF-beta receptor type-1	855	2-(6-methylpyridin-2-yl)-N-pyridin-4-ylquinazolin-4-amine
3HMP	2.3	Dual specificity protein kinase TTK	CX4	7-chloro-N-(cyclopropylmethyl)quinazolin-4-amine
3HP2	2.15	MAP kinase 14	P36	1-benzyl-4-(benzyloxy)-3-bromopyridin-2(1H)-one
3HP5	2.3	MAP kinase 14	52P	5-(2,6-dichlorophenyl)-2-[(2,4-difluorophenyl)sulfanyl]-6H-pyrimido[1,6-b]pyridazin-6-one
3HRB	2.2	MAP kinase 14	I39	[3-amino-2-(2-methylphenyl)-1-oxidopyridin-4-yl](2,4-difluorophenyl)methanone
3HUC	1.8	MAP kinase 14	G97	N-[2-phenyl-4-(1H-pyrazol-3-ylamino)quinazolin-7-yl]prop-2-enamide
3HV3	2	MAP kinase 14	R49	1-{4-[(6-aminoquinolin-4-yl)amino]phenyl}-3-[3-tert-butyl-1-(3-methylphenyl)-1H-pyrazol-5-yl]urea
3HV5	2.25	MAP kinase 14	R24	1-[3-tert-butyl-1-(4-methylphenyl)-1H-pyrazol-5-yl]-3-{3-[(6-nitroquinolin-4-yl)amino]phenyl}urea
3HV6	1.95	MAP kinase 14	R39	1-[3-tert-butyl-1-(4-methylphenyl)-1H-pyrazol-5-yl]-3-[4-(2-morpholin-4-ylethoxy)phenyl]urea
3HVC	2.1	MAP kinase 14	GG5	4-[3-(4-FLUOROPHENYL)-1H-PYRAZOL-4-YL]PYRIDINE
3HZT	2	Calcium-dependent protein kinase 3	J60	5-[(E)-(5-CHLORO-2-OXO-1,2-DIHYDRO-3H-INDOL-3-YLIDENE)METHYL]-N-[2-(DIETHYLAMINO)ETHYL]-2,4-DIMETHYL-1H-PYRROLE-3-CARBOXAMIDE
3I5N	2	Hepatocyte growth factor receptor	B2D	7-methoxy-N-[(6-phenyl[1,2,4]triazolo[4,3-b]pyridazin-3-yl)methyl]-1,5-naphthyridin-4-amine
3I5Z	2.2	MAP kinase 1	Z48	N-[(1S)-2-hydroxy-1-phenylethyl]-4-[5-methyl-2-(phenylamino)pyrimidin-4-yl]-1H-pyrrole-2-carboxamide
3I60	2.5	MAP kinase 1	E86	4-{2-[(2-chlorophenyl)amino]-5-methylpyrimidin-4-yl}-N-[(1S)-2-hydroxy-1-phenylethyl]-1H-pyrrole-2-carboxamide

3I7C	1.98	Calmodulin-domain protein kinase 1	BK2	1-tert-butyl-3-naphthalen-2-yl-1H-pyrazolo[3,4-d]pyrimidin-4-amine
3I81	2.08	Insulin-like growth factor 1 receptor	EBI	1-{4-[(3-cyclopropyl-1H-pyrazol-5-yl)amino]pyrrolo[2,1-f][1,2,4]triazin-2-yl}-N-(6-fluoropyridin-3-yl)-2-methyl-L-prolinamide
3IG7	1.8	Cyclin-dependent kinase 2	EFP	N-{1-[cis-3-(acetylamino)cyclobutyl]-1H-imidazol-4-yl}-2-(4-methoxyphenyl)acetamide
3IGG	1.8	Cyclin-dependent kinase 2	EFQ	N-[1-(cis-3-hydroxycyclobutyl)-1H-imidazol-4-yl]-2-(4-methoxyphenyl)acetamide
3IK3	1.9	Proto-oncogene tyrosine-protein kinase ABL1	0LI	3-(imidazo[1,2-b]pyridazin-3-ylethynyl)-4-methyl-N-{4-[(4-methylpiperazin-1-yl)methyl]-3-(trifluoromethyl)phenyl}benzamide
3IOK	2.1	Tyrosine-protein kinase JAK2	1P6	3-(6-{[(1S)-1-(4-fluorophenyl)ethyl]amino}pyrimidin-4-yl)pyrazolo[1,5-a]pyrimidin-2-amine
3ION	2.4	3-phosphoinositide-dependent protein kinase 1	8H1	2-(5-{[(2S)-2-amino-3-phenylpropyl]oxy}pyridin-3-yl)-8,9-dimethoxybenzo[c][2,7]naphthyridin-4-amine
3IOP	2.2	3-phosphoinositide-dependent protein kinase 1	8I1	2-(5-{[(2R)-2-amino-3-phenylpropyl]oxy}pyridin-3-yl)-8,9-dimethoxybenzo[c][2,7]naphthyridin-4-amine
3IPH	2.1	MAP kinase 14	G11	6-[5-(cyclopropylcarbamoyl)-2-methylphenyl]-N-(cyclopropylmethyl)pyridine-3-carboxamide
3ITZ	2.25	MAP kinase 14	P66	4-chloro-N-cyclopropyl-3-{[1-(2,6-difluorophenyl)-1H-pyrazolo[3,4-d]pyridazin-4-yl]amino}benzamide
3IW5	2.5	MAP kinase 14	DF3	N-[2-(3-{[2-(2,3-dihydro-1,4-benzodioxin-6-ylamino)-2-oxoethyl]sulfanyl}-1H-indol-1-yl)ethyl]-3-(trifluoromethyl)benzamide
3IW6	2.1	MAP kinase 14	PP0	ethyl 4-[(4-benzylpiperazin-1-yl)carbonyl]-1-ethyl-3,5-dimethyl-1H-pyrrole-2-carboxylate
3IW7	2.4	MAP kinase 14	IPK	2-({4-[(4-benzylpiperidin-1-yl)carbonyl]benzyl}sulfanyl)-3H-imidazo[4,5-c]pyridine
3IW8	2	MAP kinase 14	HIZ	1-{4-[(1S)-1-amino-2-(benzyloxy)ethyl]-1,3-thiazol-2-yl}-3-(3-chloro-4-fluorophenyl)urea
3JPV	2.35	Proto-oncogene serine/threonine-protein kinase	1DR	1,10-dihydropyrrolo[2,3-a]carbazole-3-carbaldehyde
3JS2	2.2	Basic fibroblast growth factor receptor 1	VM1	5-(2-thienyl)nicotinic acid
3JY0	2.4	Proto-oncogene serine/threonine-protein kinase	LYG	8-chloro-2-{[(3S)-3-hydroxypyrrolidin-1-yl]methyl}[1]benzothieno[3,2-d]pyrimidin-4(3H)-one
3JY9	2.1	Tyrosine-protein kinase JAK2	JZH	(3S)-3-(4-hydroxyphenyl)-1,5-dihydro-1,5,12-triazabenzo[4,5]cycloocta[1,2,3-cd]inden-4(3H)-one
3JYA	2.1	Proto-oncogene serine/threonine-protein kinase	LWG	6,9-dichloro[1]benzothieno[3,2-d]pyrimidin-4(3H)-one
3K3I	1.7	MAP kinase 14	JZJ	(3S)-3-[4-(4-bromophenyl)-1H-imidazol-2-yl]-1,2,3,4-tetrahydroisoquinoline
3K3J	1.995	MAP kinase 14	F4C	2-(4-fluorophenyl)-3-oxo-6-pyridin-4-yl-N-[2-(trifluoromethyl)benzyl]-2,3-dihydropyridazine-4-carboxamide
3K5U	2.35	Serine/threonine-protein kinase 6	PFQ	2-[(5,6-DIPHENYLFURO[2,3-D]PYRIMIDIN-4-YL)AMINO]ETHANOL
3KB7	2.5	Serine/threonine-protein kinase PLK1	071	8-{[2-methoxy-5-(4-methylpiperazin-1-yl)phenyl]amino}-1-methyl-4,5-dihydro-1H-pyrazolo[4,3-h]quinazoline-3-carboxamide
3KCK	2.2	Tyrosine-protein kinase JAK2	3KC	3-chloro-4-(4H-3,4,7-triazadibenzo[cd,f]azulen-6-yl)phenol
3KF4	1.9	Tyrosine-protein kinase ABL1	B90	N-[4-(dimethylphosphoryl)phenyl]-9-[(E)-2-(5-methyl-1H-indazol-4-yl)ethenyl]-9H-purin-6-amine
3KF7	2	MAP kinase 14	L9G	3-{6-[2-(2,4-difluorophenyl)ethyl][1,2,4]triazolo[4,3-a]pyridin-3-yl}-4-methylbenzamide
3KFA	1.22	Tyrosine-protein kinase ABL1	B91	3-{(E)-2-[6-(cyclopropylamino)-9H-purin-9-yl]ethenyl}-4-methyl-N-[3-(4-methyl-1H-imidazol-1-yl)-5-(trifluoromethyl)phenyl]benzamide
3KKV	1.8	cAMP-dependent protein kinase	B99	(2S)-1-{[6-furan-3-yl-5-(3-methyl-2H-indazol-5-yl)pyridin-3-yl]oxy}-3-(1H-indol-3-yl)propan-2-amine
3KQ7	1.8	MAP kinase p38 alpha	KQ7	N-(2-methyl-5-{[(2-morpholin-4-ylpyridin-4-yl)carbonyl]amino}phenyl)-6-({2-[(2S)-1-methylpyrrolidin-2-yl]ethyl}amino)pyridine-3-carboxamide
3KRJ	2.1	Macrophage colony-stimulating factor 1 receptor	KRJ	4-cyano-N-(2-cyclohex-1-en-1-yl-4-piperidin-4-ylphenyl)-1H-imidazole-2-carboxamide
3KRL	2.4	Macrophage colony-stimulating factor 1 receptor	KRL	5-cyano-N-[4-(4-methylpiperazin-1-yl)-2-piperidin-1-ylphenyl]furan-2-carboxamide
3KRR	1.8	Tyrosine-protein kinase JAK2	DQX	8-[3,5-difluoro-4-(morpholin-4-ylmethyl)phenyl]-2-(1-piperidin-4-yl-1H-pyrazol-4-yl)quinoxaline
3KVW	2.28	Dual specificity tyrosine-phosphorylation-	IRB	(2Z,3E)-7'-bromo-3-(hydroxyimino)-2'-oxo-1,1',2',3-tetrahydro-2,3'-biindole-5-carboxylic acid
3KVX	2.4	MAP kinase 10	FMY	N-[(2Z)-4-(3-fluoro-5-morpholin-4-ylphenyl)pyrimidin-2(1H)-ylidene]-4-(3-morpholin-4-yl-1H-1,2,4-triazol-1-yl)aniline
3KXG	1.7	Casein kinase II	K6X	3,4,5,6,7-pentabromo-1H-indazole
3KXH	1.7	Casein kinase II	K66	[4,5,6,7-tetrabromo-2-(dimethylamino)-1H-benzimidazol-1-yl]acetic acid
3KXM	1.75	Casein kinase II	K74	N-methyl-2-[(4,5,6,7-tetrabromo-1-methyl-1H-benzimidazol-2-yl)sulfanyl]acetamide
3KXN	2	Casein kinase II	K8X	4,5,6,7-tetraiodo-1H-benzimidazole
3KXZ	2.37	Proto-oncogene tyrosine-protein kinase LCK	925	3-[7-[(3-hydroxyphenyl)amino]pyrazolo[1,5-a]pyrimidin-2-yl]-N-(1-hydroxy-2,2,6,6-tetramethyl-piperidin-4-yl)benzamide
3L54	2.3	Phosphatidylinositol 4,5-bisphosphate 3-kinase	LXX	6-(1H-pyrazolo[3,4-b]pyridin-5-yl)-4-pyridin-4-ylquinoline
3L8P	2.4	Angiopoietin-1 receptor	0CE	2-methyl-11-(1-methylethyl)-8-[(2S)-tetrahydro-2H-pyran-2-yl]-2,11,12,13-tetrahydro-4H-indazolo[5,4-a]pyrrolo[3,4-c]carbazol-4-one
3L8S	2.35	MAP kinase 14	BFF	3-[(4-bromo-2,6-difluorobenzyl)oxy]-5-{[(4-pyrrolidin-1-ylbutyl)carbamoyl]amino}isothiazole-4-carboxamide
3L8V	2.4	Hepatocyte growth factor receptor	L8V	2-({4-[(2-aminopyridin-4-yl)oxy]-3-fluorophenyl}amino)-N-(2,4-difluorophenyl)pyridine-3-carboxamide
3L8X	2.4	MAP kinase 14	N4D	N,4-dimethyl-3-[(1-phenyl-1H-pyrazolo[3,4-d]pyrimidin-4-yl)amino]benzamide
3L9L	2	cAMP-dependent protein kinase	L9L	5-[2-({(2S)-2-amino-3-[4-(trifluoromethyl)phenyl]propyl}amino)-1,3-thiazol-5-yl]-1,3-dihydro-2H-indol-2-one
3L9M	1.9	cAMP-dependent protein kinase	L9M	(2S)-N~1~-[5-(3-methyl-1H-indazol-5-yl)-1,3,4-thiadiazol-2-yl]-3-(4-methylphenyl)propane-1,2-diamine
3L9N	2	cAMP-dependent protein kinase	L9N	(2S)-N~1~-[5-(1H-indazol-5-yl)-1,3,4-thiadiazol-2-yl]-3-(4-methylphenyl)propane-1,2-diamine
3LAU	2.1	Serine/threonine-protein kinase 6	OFI	N-[6-(4-hydroxyphenyl)-1H-indazol-3-yl]butanamide
3LCD	2.5	Macrophage colony-stimulating factor 1 receptor	BDY	N~3~-(2,6-dichlorobenzyl)-5-(4-{[(2R)-2-(pyrrolidin-1-ylmethyl)pyrrolidin-1-yl]carbonyl}phenyl)pyrazine-2,3-diamine
3LE6	2	Cyclin-dependent kinase 2	2BZ	5-(2-chlorophenyl)-3-methyl-7-nitropyrazolo[3,4-b][1,4]benzodiazepine
3LFE	2.3	MAP kinase 14	Z84	1-[3-tert-butyl-1-(4-methylphenyl)-1H-pyrazol-5-yl]-3-{4-[2-(pyridin-4-ylmethoxy)ethyl]-1,3-thiazol-2-yl}urea
3LFF	1.5	MAP kinase 14	Z83	(4-{3-tert-butyl-5-[(1,3-thiazol-2-ylcarbamoyl)amino]-1H-pyrazol-1-yl}phenyl)acetic acid
3LFN	2.28	Cyclin-dependent kinase 2	A27	N-[6-(4-hydroxyphenyl)-5-phenyl-1H-indazol-3-yl]butanamide

3LFQ	2.03	Cyclin-dependent kinase 2	A28	N-(6,7-difluoro-5-phenyl-1H-indazol-3-yl)butanamide
3LFS	2.4	Cyclin-dependent kinase 2	A07	N-(6-chloro-5-phenyl-1H-indazol-3-yl)butanamide
3LJ3	2.43	Phosphatidylinositol 4,5-bisphosphate 3-kinase	WYE	(2Z)-4,6-dihydroxy-2-{[1-methyl-4-(4-methylpiperazin-1-yl)-1H-pyrrolo[2,3-b]pyridin-3-yl]methylidene}-1-benzofuran-3(2H)-one
3LM0	2.35	Serine/threonine-protein kinase 17B	EBD	[4-({4-[(5-cyclopropyl-1H-pyrazol-3-yl)amino]-5-methylpyrimidin-2-yl}amino)phenyl]acetonitrile
3LM5	2.29	Serine/threonine-protein kinase 17B	QUE	3,5,7,3',4'-PENTAHYDROXYFLAVONE
3LPB	2	Tyrosine-protein kinase JAK2	NVB	N-methyl-4-[3-(3,4,5-trimethoxyphenyl)quinoxalin-5-yl]benzenesulfonamide
3LQ8	2.02	Hepatocyte growth factor receptor	88Z	N-(3-fluoro-4-{[6-methoxy-7-(3-morpholin-4-ylpropoxy)quinolin-4-yl]oxy}phenyl)-N'-(4-fluorophenyl)cyclopropane-1,1-dicarboxamide
3LXL	1.74	Tyrosine-protein kinase JAK3	IZA	2-TERT-BUTYL-9-FLUORO-3,6-DIHYDRO-7H-BENZ[H]-IMIDAZ[4,5-F]ISOQUINOLINE-7-ONE
3M2W	2.41	MAP kinase-activated protein kinase 2	L8I	2'-(2-fluorophenyl)-1-methyl-6',8',9',11'-tetrahydrospiro[azetidine-3,10'-pyrido[3',4':4,5]pyrrolo[2,3-f]isoquinolin]-7'(5'H)-one
3MA3	2.3	Proto-oncogene serine/threonine-protein kinase	01I	naphtho[2,1-b:7,6-b']difuran-2,8-dicarboxylic acid
3MA6	2.5	Calmodulin-domain protein kinase 1	DXR	3-(3-bromobenzyl)-1-tert-butyl-1H-pyrazolo[3,4-d]pyrimidin-4-amine
3MB7	1.65	Casein kinase II	14I	naphtho[2,1-b:7,8-b']difuran-2,9-dicarboxylic acid
3MDY	2.05	Bone morphogenetic protein receptor type-1B	LDN	4-[6-(4-piperazin-1-ylphenyl)pyrazolo[1,5-a]pyrimidin-3-yl]quinoline
3MIY	1.67	Tyrosine-protein kinase ITK/TSK	B49	N-[2-(diethylamino)ethyl]-5-[(Z)-(5-fluoro-2-oxo-1,2-dihydro-3H-indol-3-ylidene)methyl]-2,4-dimethyl-1H-pyrrole-3-carboxamide
3MJ1	1.72	Tyrosine-protein kinase ITK/TSK	614	7-[(4-methylpiperazin-1-yl)methyl]-4-[(3-methyl-1H-pyrazol-5-yl)amino]-2-(tetrahydro-2H-pyran-4-yl)phthalazin-1(2H)-one
3MJ2	1.9	Tyrosine-protein kinase ITK/TSK	MJG	N-[5-({5-[(4-acetylpiperazin-1-yl)carbonyl]-4-methoxy-2-methylphenyl}sulfanyl)-1,3-thiazol-2-yl]-4-({[(1S)-1,2,2-trimethylpropyl]amino}methyl)benzamide
3MPM	1.95	Tyrosine-protein kinase Lck	5LK	4-{(6R,7R)-7-amino-3-[3-(4-methylpiperazin-1-yl)phenyl]-6,7-dihydropyrazolo[1,5-a]pyrimidin-6-yl}phenol
3MPT	1.89	MAP kinase 14	1GK	N-(furan-2-ylmethyl)-4-[(2-methylphenyl)carbonyl]-1H-pyrrole-2-carboxamide
3MTF	2.15	Activin receptor type-1	A3F	3-[6-amino-5-(3,4,5-trimethoxyphenyl)pyridin-3-yl]phenol
3MVH	2.01	RAC-alpha serine/threonine-protein kinase	WFE	N-{[(3S)-3-amino-1-(5-ethyl-7H-pyrrolo[2,3-d]pyrimidin-4-yl)pyrrolidin-3-yl]methyl}-2,4-difluorobenzamide
3MVJ	2.49	cAMP-dependent protein kinase	XFE	(3R)-1-(5-methyl-7H-pyrrolo[2,3-d]pyrimidin-4-yl)pyrrolidin-3-amine
3MVM	2	MAP kinase 14	39P	4-{[5-(isoxazol-3-ylcarbamoyl)-2-methylphenyl]amino}-5-methyl-N-propylpyrrolo[2,1-f][1,2,4]triazine-6-carboxamide
3MWU	1.98	Calmodulin-domain protein kinase 1	BK3	3-(naphthalen-1-ylmethyl)-1-(piperidin-4-ylmethyl)-1H-pyrazolo[3,4-d]pyrimidin-4-amine
3MY5	2.1	Cyclin-dependent kinase 2	RFZ	5,6-dichloro-1-beta-D-ribofuranosyl-1H-benzimidazole
3MYG	2.4	Serine/threonine-protein kinase 6	EML	2-{ethyl[(5-{[6-methyl-3-(1H-pyrazol-4-yl)imidazo[1,2-a]pyrazin-8-yl]amino}isothiazol-3-yl)methyl]amino}-2-methylpropan-1-ol
3NAX	1.75	3-phosphoinositide-dependent protein kinase 1	MP7	1-(3,4-difluorobenzyl)-2-oxo-N-{(1R)-2-[(2-oxo-2,3-dihydro-1H-benzimidazol-5-yl)oxy]-1-phenylethyl}-1,2-dihydropyridine-3-carboxamide
3NCG	2.49	Calmodulin-domain protein kinase 1	BK1	1-(1-methylethyl)-3-(naphthalen-1-ylmethyl)-1H-pyrazolo[3,4-d]pyrimidin-4-amine
3NLB	1.9	Serine/threonine-protein kinase Chk1	5BE	3-methyl-5-[5-(1-methylethyl)-1H-benzimidazol-2-yl]-N-(1-methylpiperidin-4-yl)-1H-pyrazole-4-carboxamide
3NNU	2.4	MAP kinase 14	EDB	2-{3-[(5E)-5-{[(2,3-dichlorophenyl)carbamoyl]imino}-3-thiophen-2-yl-2,5-dihydro-1H-pyrazol-1-yl]phenyl}acetamide
3NNV	2.1	MAP kinase 14	437	1-{3-tert-butyl-1-[4-(hydroxymethyl)phenyl]-1H-pyrazol-5-yl}-3-naphthalen-1-ylurea
3NNW	1.89	MAP kinase 14	EDD	2-[3-(3-tert-butyl-5-{[(2,3-dichlorophenyl)carbamoyl]imino}-2,5-dihydro-1H-pyrazol-1-yl)phenyl]acetamide
3NPC	2.35	MAP kinase 9	B96	1-(5-TERT-BUTYL-2-P-TOLYL-2H-PYRAZOL-3-YL)-3-[4-(2-MORPHOLIN-4-YL-ETHOXY)-NAPHTHALEN-1-YL]-UREA
3NS9	1.78	Cyclin-dependent kinase 2	NS9	(2S,3S)-3-{[7-(benzylamino)-3-(1-methylethyl)pyrazolo[1,5-a]pyrimidin-5-yl]amino}butane-1,2,4-triol
3NUN	2.2	PKB-like	JMZ	6-(2-aminopyrimidin-4-yl)-1H-indazol-3-amine
3NUU	1.9803	PKB-like	JOZ	3,4-dihydroisoquinolin-1(2H)-one
3NUY	2.1	PKB-like	JPZ	quinazolin-4(1H)-one
3NW5	2.14	Insulin-like growth factor 1 receptor	LGX	N-(5-cyclopropyl-1H-pyrazol-3-yl)-2-{(2R)-1-[(6-fluoropyridin-3-yl)carbonyl]pyrrolidin-2-yl}pyrrolo[2,1-f][1,2,4]triazin-4-amine
3NW6	2.2	Insulin-like growth factor 1 receptor	LGW	N-(5-cyclopropyl-1H-pyrazol-3-yl)-2-{(2S)-1-[(6-fluoropyridin-3-yl)carbonyl]pyrrolidin-2-yl}pyrrolo[2,1-f][1,2,4]triazin-4-amine
3NW7	2.11	Insulin-like growth factor 1 receptor	LGV	N-({4-[(3-cyclopropyl-1H-pyrazol-5-yl)amino]pyrrolo[2,1-f][1,2,4]triazin-2-yl}methyl)-6-fluoropyridine-3-carboxamide
3NWW	2.09	MAP kinase 14	3NW	1-[2-(2-{[2-(dimethylamino)ethyl]amino}-6-{2-[(1-methylethyl)amino]-1,3-thiazol-5-yl}pyrimidin-4-yl)benzyl]-3-ethylurea
3NYX	2.5	Non-receptor tyrosine-protein kinase TYK2	TZ1	N-{5-[(7-chloroquinolin-4-yl)sulfanyl]-1,3,4-thiadiazol-2-yl}thiophene-2-carboxamide
3O0G	1.95	Cyclin-dependent kinase 5	3O0	{4-amino-2-[(4-chlorophenyl)amino]-1,3-thiazol-5-yl}(3-nitrophenyl)methanone
3O23	2.1	Insulin-like growth factor 1 receptor	MQY	(5S)-5-methyl-1-(quinolin-4-ylmethyl)-3-{4-[(trifluoromethyl)sulfonyl]phenyl}imidazolidine-2,4-dione
3O50	2	Serine/threonine-protein kinase 6	LJE	N-{3-methyl-4-[(3-pyrimidin-4-ylpyridin-2-yl)oxy]phenyl}-3-(trifluoromethyl)benzamide
3O8T	2	MAP kinase 14	BMU	1-(5-TERT-BUTYL-2-METHYL-2H-PYRAZOL-3-YL)-3-(4-CHLORO-PHENYL)-UREA
3OCG	2.21	MAP kinase 14	OCG	5-amino-N-[5-(isoxazol-3-ylcarbamoyl)-2-methylphenyl]-1-phenyl-1H-pyrazole-4-carboxamide
3OCS	1.8	Tyrosine-protein kinase BTK	746	4-tert-butyl-N-[2-methyl-3-(4-methyl-6-{[4-(morpholin-4-ylcarbonyl)phenyl]amino}-5-oxo-4,5-dihydropyrazin-2-yl)phenyl]benzamide
3OG7	2.45	Serine/threonine-protein kinase B-raf	032	N-(3-{[5-(4-chlorophenyl)-1H-pyrrolo[2,3-b]pyridin-3-yl]carbonyl}-2,4-difluorophenyl)propane-1-sulfonamide
3OOG	2	cAMP-dependent protein kinase	YTP	1-(4-hydroxy-3-methylphenyl)ethanone
3OOM	2	Activin receptor type-1	507	1-{3-[6-(tetrahydro-2H-pyran-4-ylamino)imidazo[1,2-b]pyridazin-3-yl]phenyl}ethanone
3OP5	2.4	Serine/threonine-protein kinase VRK1	REB	[4-({4-[(5-cyclopropyl-1H-pyrazol-3-yl)amino]pyrimidin-2-yl}amino)phenyl]acetonitrile
3OT3	1.44	Serine/threonine-protein kinase Chk1	22K	5-[(1R,3S)-3-aminocyclohexyl]-6-bromo-3-(1-methyl-1H-pyrazol-4-yl)pyrazolo[1,5-a]pyrimidin-7-amine
3OT8	1.6455	Serine/threonine-protein kinase Chk1	MI5	N-(3-methylisothiazol-5-yl)-3-(1-methyl-1H-pyrazol-4-yl)-5-[(3R)-piperidin-3-yl]pyrazolo[1,5-a]pyrimidin-7-amine

3OVV	1.58	cAMP-dependent protein kinase	1SB	N'-[(1E)-(4-hydroxyphenyl)methylidene]-2-(3-methoxyphenyl)acetohydrazide
3OW3	1.9	cAMP-dependent protein kinase	SMY	(2R)-3-(1H-indol-3-yl)-1-{4-[(5S)-5-methyl-5,7-dihydrothieno[3,4-d]pyrimidin-4-yl]piperazin-1-yl}-1-oxopropan-2-amine
3OWJ	1.85	Casein kinase II	1EL	9-hydroxy-5,11-dimethyl-4,6-dihydro-1H-pyrido[4,3-b]carbazol-1-one
3OWK	1.8	Casein kinase II	18E	7-chloro-10-methyl-11H-benzo[g]pyrido[4,3-b]indol-3-ol
3OWL	2.1	Casein kinase II	19E	11-chloro-8-methyl-7H-benzo[e]pyrido[4,3-b]indol-3-ol
3OWP	1.88	cAMP-dependent protein kinase	2SB	(2S)-2-amino-N'-[(1E)-(3-bromo-4-hydroxyphenyl)methylidene]-2-phenylethanehydrazide
3OXI	2.2	MAP kinase 10	SYY	methyl 3-[(thiophen-2-ylacetyl)amino]thiophene-2-carboxylate
3OXT	2.2	cAMP-dependent protein kinase	3SB	(2S)-2-amino-N'-[(1E)-(2,4-dihydroxy-6-methylphenyl)methylidene]-2-phenylethanehydrazide
3OY1	1.7	MAP kinase 10	589	5-[2-(cyclohexylamino)pyridin-4-yl]-4-naphthalen-2-yl-2-(tetrahydro-2H-pyran-4-yl)-2,4-dihydro-3H-1,2,4-triazol-3-one
3OY3	1.95	Tyrosine-protein kinase ABL1	XY3	5-[(5-{[4-{[4-(2-hydroxyethyl)piperazin-1-yl]methyl}-3-(trifluoromethyl)phenyl]carbamoyl}-2-methylphenyl)ethynyl]-1-methyl-1H-imidazole-2-carboxamide
3P0M	2.03	cAMP-dependent protein kinase	4SB	(2R)-4-amino-N'-[(1E)-(3-bromo-4-hydroxyphenyl)methylidene]-2-phenylbutanehydrazide
3P5K	2.09	MAP kinase 14	P5K	1-{5-tert-butyl-3-[(1,1-dioxidothiomorpholin-4-yl)carbonyl]thiophen-2-yl}-3-naphthalen-1-ylurea
3P78	2.3	MAP kinase 14	P78	1-{5-tert-butyl-3-[(1,1-dioxidothiomorpholin-4-yl)carbonyl]thiophen-2-yl}-3-naphthalen-2-ylurea
3P79	2.1	MAP kinase 14	P79	1-{3-tert-butyl-1-[2-(1,1-dioxidothiomorpholin-4-yl)-2-oxoethyl]-1H-pyrazol-5-yl}-3-naphthalen-2-ylurea
3P7A	2.31	MAP kinase 14	P7A	1-[5-tert-butyl-2-(1,1-dioxidothiomorpholin-4-yl)thiophen-3-yl]-3-naphthalen-1-ylurea
3P7B	1.9	MAP kinase 14	P7B	1-{5-tert-butyl-3-[(5-oxo-1,4-diazepan-1-yl)carbonyl]thiophen-2-yl}-3-naphthalen-1-ylurea
3P7C	2.3	MAP kinase 14	P7C	1-[5-tert-butyl-3-({4-[2-(dimethylamino)ethyl]-5-oxo-1,4-diazepan-1-yl}carbonyl)thiophen-2-yl]-3-(2,3-dichlorophenyl)urea
3P86	2.496	Serine/threonine-protein kinase CTR1	STU	STAUROSPORINE
3PA3	1.4	Serine/threonine-protein kinase Chk1	C70	2-(4-chlorophenyl)-4-[(3S)-piperidin-3-ylamino]thieno[2,3-d]pyridazine-7-carboxamide
3PA4	1.59	Serine/threonine-protein kinase Chk1	C72	2-(4-chlorophenyl)-4-[(3S)-piperidin-3-ylamino]thieno[3,2-c]pyridine-7-carboxamide
3PA5	1.7	Serine/threonine-protein kinase Chk1	C73	2-(carbamoylamino)-5-(4-chlorophenyl)-N-[(3S)-piperidin-3-yl]thiophene-3-carboxamide
3PE1	1.6	Casein kinase II	3NG	5-[(3-chlorophenyl)amino]benzo[c][2,6]naphthyridine-8-carboxylic acid
3PE2	1.9	Casein kinase II	E1B	5-[(3-ethynylphenyl)amino]pyrimido[4,5-c]quinoline-8-carboxylic acid
3PG3	2	MAP kinase 14	DG7	1-[3-tert-butyl-1-(4-methylphenyl)-1H-pyrazol-5-yl]-3-{4-[2-(pyridin-3-ylmethoxy)ethyl]-1,3-thiazol-2-yl}urea
3PIX	1.85	Tyrosine-protein kinase BTK	027	7-(4-methylpiperazin-1-yl)-4-[(5-methyl-1H-pyrazol-3-yl)amino]-2-(propan-2-yl)phthalazin-1(2H)-one
3PIZ	2.21	Tyrosine-protein kinase BTK	03C	[5-amino-1-(2-methylphenyl)-1H-pyrazol-4-yl]{3-[1-(methylsulfonyl)piperidin-4-yl]phenyl}methanone
3PJ1	2	Tyrosine-protein kinase BTK	LHL	3-(2,6-dichlorophenyl)-7-({4-[2-(diethylamino)ethoxy]phenyl}amino)-1-methyl-3,4-dihydropyrimido[4,5-d]pyrimidin-2(1H)-one
3PJ2	1.75	Tyrosine-protein kinase BTK	04K	2-({4-[2-(diethylamino)ethoxy]phenyl}amino)-6-(4-fluorophenoxy)-8-methylpyrido[2,3-d]pyrimidin-7(8H)-one
3PJ3	1.85	Tyrosine-protein kinase BTK	04L	2-methyl-N-(2-phenyl-3H-imidazo[4,5-b]pyridin-6-yl)-5-{[(2E)-3-phenylprop-2-enoyl]amino}benzamide
3PJ8	1.96	Cyclin-dependent kinase 2	404	(2R)-2-{[7-(benzylamino)-3-(propan-2-yl)-1H-pyrazolo[4,3-d]pyrimidin-5-yl]amino}butan-1-ol
3PJC	2.2	Tyrosine-protein kinase JAK3	PJC	3-(1H-indol-3-yl)-4-[2-(4-oxopiperidin-1-yl)-5-(trifluoromethyl)pyrimidin-4-yl]-1H-pyrrole-2,5-dione
3POO	1.6	cAMP-dependent protein kinase	S69	N'-[(E)-(2,4-dihydroxy-6-methylphenyl)methylidene]-2-(3-methoxyphenyl)acetohydrazide
3POZ	1.5	Epidermal growth factor receptor	03P	N-{2-[4-({3-chloro-4-[3-(trifluoromethyl)phenoxy]phenyl}amino)-5H-pyrrolo[3,2-d]pyrimidin-5-yl]ethyl}-3-hydroxy-3-methylbutanamide
3PP0	2.25	Receptor tyrosine-protein kinase erbB-2	03Q	2-{2-[4-({5-chloro-6-[3-(trifluoromethyl)phenoxy]pyridin-3-yl}amino)-5H-pyrrolo[3,2-d]pyrimidin-5-yl]ethoxy}ethanol
3PTG	2.43	MAP kinase 10	932	N-[4-methyl-3-(1H-1,2,4-triazol-5-yl)thiophen-2-yl]-2-(2-oxo-3,4-dihydroquinolin-1(2H)-yl)acetamide
3PVG	1.5	Casein kinase II	K68	(4,5,6,7-tetrabromo-1H-benzimidazol-1-yl)acetic acid
3PVU	2.48	Beta-adrenergic receptor kinase 1	QRW	3-({[4-methyl-5-(pyridin-4-yl)-4H-1,2,4-triazol-3-yl]methyl}amino)-N-[2-(trifluoromethyl)benzyl]benzamide
3PVW	2.49	Beta-adrenergic receptor kinase 1	QRX	N-(2,6-difluorobenzyl)-3-({[4-propyl-5-(pyrimidin-4-yl)-4H-1,2,4-triazol-3-yl]methyl}amino)benzamide
3PWD	2.2	Casein kinase II	CZ0	8-hydroxy-4-methyl-9-nitro-2H-benzo[g]chromen-2-one
3PXY	1.8	Cyclin-dependent kinase 2	JWS	2-(4,6-diamino-1,3,5-triazin-2-yl)-4-methoxyphenol
3PXZ	1.7	Cyclin-dependent kinase 2	2AN	8-ANILINO-1-NAPHTHALENE SULFONATE
3PY0	1.75	Cyclin-dependent kinase 2	SU9	(3Z)-3-(1H-IMIDAZOL-5-YLMETHYLENE)-5-METHOXY-1H-INDOL-2(3H)-ONE
3PZE	2	MAP kinase 8	CFK	3-(carbamoylamino)-5-phenylthiophene-2-carboxamide
3PZH	1.919	Casein kinase II	EMO	3-METHYL-1,6,8-TRIHYDROXYANTHRAQUINONE
3Q32	2.5	Tyrosine-protein kinase JAK2	J2I	2-(2,6-difluoro-4-methoxyphenyl)-1-(4-{4-[(3-methyl-1H-pyrazol-5-yl)amino]pyrrolo[2,1-f][1,2,4]triazin-2-yl}piperazin-1-yl)ethanone
3Q6W	1.75	Hepatocyte growth factor receptor	Q6W	3-{5-oxo-3-[1-(piperidin-4-yl)-1H-pyrazol-4-yl]-5H-benzo[4,5]cyclohepta[1,2-b]pyridin-7-yl}-N-(pyridin-2-ylmethyl)propanamide
3Q9Y	1.8	Casein kinase II	TXQ	1,2,5,8-tetrahydroxyanthracene-9,10-dione
3QCQ	2.501	3-phosphoinositide-dependent protein kinase 1	3Q0	6-(3-amino-2H-indazol-6-yl)-N~4~-ethylpyrimidine-2,4-diamine
3QCS	2.487	3-phosphoinositide-dependent protein kinase 1	3Q1	6-[2-amino-6-(morpholin-4-yl)pyrimidin-4-yl]-2H-indazol-3-amine
3QCX	2.3	3-phosphoinositide-dependent protein kinase 1	3Q2	6-{2-amino-6-[(3R)-3-methylmorpholin-4-yl]pyrimidin-4-yl}-2H-indazol-3-amine
3QCY	2.2	3-phosphoinositide-dependent protein kinase 1	3Q3	(2S)-4-[2-amino-6-(3-amino-2H-indazol-6-yl)pyrimidin-4-yl]-N-phenylmorpholine-2-carboxamide
3QD0	1.99	3-phosphoinositide-dependent protein kinase 1	3Q4	(3S,6R)-1-[2-amino-6-(3-amino-2H-indazol-6-yl)pyrimidin-4-yl]-6-methyl-N-phenylpiperidine-3-carboxamide
3QD3	2	3-phosphoinositide-dependent protein kinase 1	3Q5	tert-butyl {(3R,6S)-1-[2-amino-6-(3-amino-2H-indazol-6-yl)pyrimidin-4-yl]-6-methylpiperidin-3-yl}carbamate

3QD4	2.3	3-phosphoinositide-dependent protein kinase 1	3Q6	tert-butyl {(3R,5R)-1-[2-amino-6-(3-amino-2H-indazol-6-yl)pyrimidin-4-yl]-5-methylpiperidin-3-yl}carbamate
3QF9	2.2	Proto-oncogene serine/threonine-protein kinase	NM8	6-{5-[(Z)-(2,4-dioxo-1,3-thiazolidin-5-ylidene)methyl]furan-2-yl}-N-{3-[(4-ethylpiperazin-1-yl)methyl]-5-(trifluoromethyl)phenyl}naphthalene-1-carboxamide
3QGW	2.1	Tyrosine-protein kinase ITK/TSK	L7A	N-(6-oxo-1,6-dihydro-3,4'-bipyridin-5-yl)benzamide
3QGY	2.1	Tyrosine-protein kinase ITK/TSK	L7O	N-{5-[2-(methylamino)pyrimidin-4-yl]-2-oxo-1,2-dihydropyridin-3-yl}-4-(piperidin-1-yl)benzamide
3QKK	2.3	RAC-alpha serine/threonine-protein kinase	SMH	N-(2-ethoxyethyl)-N-{(2S)-2-hydroxy-3-[(2R)-6-hydroxy-4-oxo-3,4-dihydro-1'H-spiro[chromene-2,3'-piperidin]-1'-yl]propyl}-2,6-dimethylbenzenesulfonamide
3QKL	1.9	RAC-alpha serine/threonine-protein kinase	SMR	N-{(2S)-3-[(3S)-8',9'-dihydro-1H,3'H-spiro[piperidine-3,7'-pyrano[3,2-e]indazol]-1-yl]-2-hydroxypropyl}-N-(2-ethoxyethyl)-2,6-dimethylbenzenesulfonamide
3QKM	2.2	RAC-alpha serine/threonine-protein kinase	SM9	N-(2-ethoxyethyl)-N-{(2S)-2-hydroxy-3-[(5R)-2-(quinazolin-4-yl)-2,7-diazaspiro[4.5]dec-7-yl]propyl}-2,6-dimethylbenzenesulfonamide
3QL8	1.9	Cyclin-dependent kinase 2	X01	3-(4-amino-1,3,5-triazin-2-yl)-4-hydroxybenzonitrile
3QQF	1.75	Cyclin-dependent kinase 2	X07	5-nitro-2-[(pyridin-3-ylmethyl)amino]benzamide
3QQG	1.9	Cyclin-dependent kinase 2	X06	4-chloro-2-(4,6-diamino-1,3,5-triazin-2-yl)phenol
3QQH	1.87	Cyclin-dependent kinase 2	X0A	2-[4-amino-6-(phenylamino)-1,3,5-triazin-2-yl]-4-chlorophenol
3QQJ	1.7	Cyclin-dependent kinase 2	X11	2-(4,6-diamino-1,3,5-triazin-2-yl)phenol
3QQK	1.86	Cyclin-dependent kinase 2	X02	[4-amino-2-(prop-2-en-1-ylamino)-1,3-thiazol-5-yl](phenyl)methanone
3QQL	1.85	Cyclin-dependent kinase 2	X03	(5R)-5-(2-methylbutan-2-yl)-4,5,6,7-tetrahydro-1H-indazole-3-carbohydrazide
3QRJ	1.82	Tyrosine-protein kinase ABL1	919	4-[4-({[3-tert-butyl-1-(quinolin-6-yl)-1H-pyrazol-5-yl]carbamoyl}amino)-3-fluorophenoxy]-N-methylpyridine-2-carboxamide
3QRK	2.3	Tyrosine-protein kinase ABL1	9DP	(3S)-6-(3-tert-butyl-5-{[(2,3-dichlorophenyl)carbamoyl]amino}-1H-pyrazol-1-yl)-1,2,3,4-tetrahydroisoquinoline-3-carboxylic acid
3QRT	1.75	Cyclin-dependent kinase 2	X14	(5R)-5-(2-methylbutan-2-yl)-N-(4-sulfamoylbenzyl)-4,5,6,7-tetrahydro-2H-indazole-3-carboxamide
3QRU	1.95	Cyclin-dependent kinase 2	X19	(5S)-N-methyl-5-(2-methylbutan-2-yl)-4,5,6,7-tetrahydro-2H-indazole-3-carboxamide
3QTI	2	Hepatocyte growth factor receptor	3QT	6-{[6-(1-methyl-1H-pyrazol-4-yl)imidazo[1,2-b]pyridazin-3-yl]methyl}quinoline
3QTQ	1.8	Cyclin-dependent kinase 2	X35	[4-amino-2-(prop-2-en-1-ylamino)-1,3-thiazol-5-yl](pyridin-3-yl)methanone
3QTR	1.85	Cyclin-dependent kinase 2	X36	[4-amino-2-(phenylamino)-1,3-thiazol-5-yl](phenyl)methanone
3QTS	1.9	Cyclin-dependent kinase 2	X46	[4-amino-2-(phenylamino)-1,3-thiazol-5-yl](3-methoxyphenyl)methanone
3QTU	1.82	Cyclin-dependent kinase 2	X44	4-{[4-amino-5-(4-sulfamoylbenzoyl)-1,3-thiazol-2-yl]amino}benzenesulfonamide
3QTW	1.85	Cyclin-dependent kinase 2	X3A	[4-amino-2-(phenylamino)-1,3-thiazol-5-yl](pyridin-3-yl)methanone
3QTX	1.95	Cyclin-dependent kinase 2	X43	4-{[4-amino-5-(3-nitrobenzoyl)-1,3-thiazol-2-yl]amino}benzenesulfonamide
3QTZ	2	Cyclin-dependent kinase 2	X42	4-{[4-amino-5-(3-fluorobenzoyl)-1,3-thiazol-2-yl]amino}benzenesulfonamide
3QU0	1.95	Cyclin-dependent kinase 2	X40	4-{[4-amino-5-(pyridin-3-ylcarbonyl)-1,3-thiazol-2-yl]amino}benzenesulfonamide
3QUD	2	MAP kinase 14	N3F	{4-[(2-aminophenyl)amino]phenyl}(phenyl)methanone
3QUP	1.9	Tyrosine-protein kinase receptor TYRO3	LUN	(5-fluoro-1H-indol-2-yl)[(3R)-1'-[(3R)-piperidin-3-yl]spiro[indole-3,3'-pyrrolidin]-1(2H)-yl]methanone
3QWJ	1.75	Cyclin-dependent kinase 2	X6A	2-{[(2-aminopyrimidin-5-yl)methyl]amino}-4-chloro-5-nitrobenzamide
3QWK	1.85	Cyclin-dependent kinase 2	X62	4-chloro-2-{[(2-chloropyrimidin-5-yl)methyl]amino}-5-nitrobenzamide
3QX2	1.75	Cyclin-dependent kinase 2	X63	2-{[(2-aminopyrimidin-5-yl)methyl]amino}-4-[(2-hydroxyethyl)amino]-5-nitrobenzamide
3QX4	1.92	Cyclin-dependent kinase 2	X4B	4-chloro-5-nitro-2-[(pyridin-3-ylmethyl)amino]benzamide
3QXO	1.75	Cyclin-dependent kinase 2	X65	5-nitro-2-[(4-sulfamoylbenzyl)amino]benzamide
3QYW	1.5	MAP kinase 1	6PB	6-(3-bromophenyl)-7H-purin-2-amine
3QYZ	1.46	MAP kinase 1	Z8B	5'-azido-8-bromo-5'-deoxyadenosine
3QZF	2	Cyclin-dependent kinase 2	X66	2-(4,6-diamino-1,3,5-triazin-2-yl)benzene-1,4-diol
3QZG	1.75	Cyclin-dependent kinase 2	X67	2-(4,6-diamino-1,3,5-triazin-2-yl)-4-fluorophenol
3QZH	1.95	Cyclin-dependent kinase 2	X69	4-methoxy-5-nitro-2-[(pyridin-3-ylmethyl)amino]benzamide
3QZI	1.75	Cyclin-dependent kinase 2	X72	4-chloro-5-nitro-2-[(pyrimidin-5-ylmethyl)amino]benzamide
3R00	2.1	Proto-oncogene serine/threonine-protein kinase	UNJ	5-bromo-1-benzofuran-2-carboxylic acid
3R02	1.95	Proto-oncogene serine/threonine-protein kinase	UNM	7-[(cis-4-aminocyclohexyl)amino]-5-bromo-1-benzofuran-2-carboxylic acid
3R04	1.7	Proto-oncogene serine/threonine-protein kinase	UNQ	5-{6-[(trans-4-aminocyclohexyl)amino]pyrazin-2-yl}-1-benzofuran-2-carboxylic acid
3R0T	1.75	Casein kinase II	FU9	3-(cyclopropylamino)-5-{[3-(trifluoromethyl)phenyl]amino}pyrimido[4,5-c]quinoline-8-carboxylic acid
3R1N	2.09	MAP kinase-activated protein kinase 3	05B	2'-[2-(1,3-benzodioxol-5-yl)pyrimidin-4-yl]-5',6'-dihydrospiro[piperidine-4,7'-pyrrolo[3,2-c]pyridin]-4'(1'H)-one
3R1Q	1.85	Cyclin-dependent kinase 2	X75	4-chloro-2-{[(6-chloropyridin-3-yl)methyl]amino}-5-nitrobenzamide
3R1S	1.8	Cyclin-dependent kinase 2	X73	2-{[(6-chloropyridin-3-yl)methyl]amino}-5-nitrobenzamide
3R1Y	1.8	Cyclin-dependent kinase 2	X76	2-[(pyridin-3-ylmethyl)amino]benzamide
3R28	1.75	Cyclin-dependent kinase 2	XA0	4-chloro-5-nitro-2-[(3,4,5-trifluorobenzyl)amino]benzamide
3R6X	1.75	Cyclin-dependent kinase 2	X84	4-[(3-hydroxypropyl)amino]-5-nitro-2-[(pyridin-3-ylmethyl)amino]benzamide
3R71	1.75	Cyclin-dependent kinase 2	X86	4-[(3-methoxypropyl)amino]-5-nitro-2-[(pyridin-3-ylmethyl)amino]benzamide
3R73	1.7	Cyclin-dependent kinase 2	X87	4-[(3-aminopropyl)amino]-5-nitro-2-[(pyridin-3-ylmethyl)amino]benzamide
3R7E	1.9	Cyclin-dependent kinase 2	X88	5-nitro-2-[(pyrazin-2-ylmethyl)amino]benzamide

3R7I	1.85	Cyclin-dependent kinase 2	X9I	4-{[(2-carbamoyl-4-nitrophenyl)amino]methyl}benzoic acid
3R7O	2.3	Hepatocyte growth factor receptor	M61	N-[(2R)-1,4-dioxan-2-ylmethyl]-N-methyl-N'-{5-oxo-3-[1-(piperidin-4-yl)-1H-pyrazol-4-yl]-5H-benzo[4,5]cyclohepta[1,2-b]pyridin-7-yl}sulfuric diamide
3R7Q	2.5	Phosphatidylinositol 4,5-bisphosphate 3-kinase	FAV	N-(2-chlorophenyl)-N-methyl-4H-thieno[3,2-c]chromene-2-carboxamide
3R7U	1.75	Cyclin-dependent kinase 2	X96	2-{[4-(aminomethyl)benzyl]amino}-5-nitrobenzamide
3R7V	1.95	Cyclin-dependent kinase 2	Z02	5-nitro-2-[(pyridin-2-ylmethyl)amino]benzamide
3R7Y	1.9	Cyclin-dependent kinase 2	Z04	2-{[(2-aminopyrimidin-5-yl)methyl]amino}-4-(morpholin-4-yl)-5-nitrobenzamide
3R83	1.75	Cyclin-dependent kinase 2	Z14	4-(4-aminopiperidin-1-yl)-2-{[(2-aminopyrimidin-5-yl)methyl]amino}-5-nitrobenzamide
3R8L	1.9	Cyclin-dependent kinase 2	Z30	(5R)-5-tert-butyl-4,5,6,7-tetrahydro-1H-indazole-3-carbohydrazide
3R8M	1.8	Cyclin-dependent kinase 2	Z19	1H-indazole-3-carbohydrazide
3R8P	1.8	Cyclin-dependent kinase 2	Z46	(5R)-5-propyl-4,5,6,7-tetrahydro-1H-indazole-3-carbohydrazide
3R8U	2	Cyclin-dependent kinase 2	Z31	[4-amino-2-(prop-2-en-1-ylamino)-1,3-thiazol-5-yl](naphthalen-2-yl)methanone
3R8V	1.9	Cyclin-dependent kinase 2	Z62	[4-amino-2-(prop-2-en-1-ylamino)-1,3-thiazol-5-yl](3-nitrophenyl)methanone
3R8Z	1.85	Cyclin-dependent kinase 2	Z63	[4-amino-2-(prop-2-en-1-ylamino)-1,3-thiazol-5-yl](pyridin-4-yl)methanone
3R9D	1.95	Cyclin-dependent kinase 2	X6B	4-amino-N-(3-fluorophenyl)-2-[(4-sulfamoylphenyl)amino]-1,3-thiazole-5-carboxamide
3R9H	2.1	Cyclin-dependent kinase 2	Z67	4-amino-N-(2,6-difluorophenyl)-2-[(4-sulfamoylphenyl)amino]-1,3-thiazole-5-carboxamide
3R9N	1.75	Cyclin-dependent kinase 2	Z68	[4-amino-2-(cyclohexylamino)-1,3-thiazol-5-yl](3-nitrophenyl)methanone
3R9O	1.9	Cyclin-dependent kinase 2	Z71	4-amino-N-(3,5-difluorophenyl)-2-[(4-sulfamoylphenyl)amino]-1,3-thiazole-5-carboxamide
3RAH	1.75	Cyclin-dependent kinase 2	O1Z	[4-amino-2-(cyclohexylamino)-1,3-thiazol-5-yl](naphthalen-2-yl)methanone
3RAI	1.7	Cyclin-dependent kinase 2	X85	4-{[3-(morpholin-4-yl)propyl]amino}-5-nitro-2-[(pyridin-3-ylmethyl)amino]benzamide
3RAK	1.75	Cyclin-dependent kinase 2	03Z	4-[(4-amino-5-benzoyl-1,3-thiazol-2-yl)amino]benzenesulfonamide
3RAL	1.75	Cyclin-dependent kinase 2	04Z	4-{[4-amino-5-(3-methoxybenzoyl)-1,3-thiazol-2-yl]amino}benzenesulfonamide
3RAW	2.09	Dual specificity protein kinase CLK3	3RA	5-(1,3-benzodioxol-5-ylmethyl)-2-(phenylamino)-4H-imidazol-4-one
3RCJ	1.7	3-phosphoinositide-dependent protein kinase 1	3RC	3-(1-benzyl-1H-1,2,3-triazol-4-yl)-1H-pyrrolo[2,3-b]pyridine
3RHK	1.94	Hepatocyte growth factor receptor	M97	1-[(3R,4R)-4-(1H-indol-3-yl)-2,5-dioxopyrrolidin-3-yl]pyrrolo[3,2,1-ij]quinolinium
3RHX	2.01	Basic fibroblast growth factor receptor 1	3RH	(6S)-6-phenyl-5,6-dihydrobenzo[h]quinazolin-2-amine
3RIN	2.2	MAP kinase 14	I2O	N-cyclopropyl-4-methyl-3-(2'-oxo-1',2'-dihydrospiro[cyclopentane-1,3'-indol]-6'-yl)benzamide
3RJC	1.85	Cyclin-dependent kinase 2	06Z	{4-amino-2-[(3-fluorophenyl)amino]-1,3-thiazol-5-yl}(phenyl)methanone
3RK5	2	Cyclin-dependent kinase 2	07Z	4-{[4-amino-5-(pyridin-3-ylcarbonyl)-1,3-thiazol-2-yl]amino}benzoic acid
3RK7	1.8	Cyclin-dependent kinase 2	08Z	4-{[4-amino-5-(pyridin-3-ylcarbonyl)-1,3-thiazol-2-yl]amino}benzamide
3RK9	1.85	Cyclin-dependent kinase 2	09Z	[4-amino-2-(propan-2-ylamino)-1,3-thiazol-5-yl](pyridin-3-yl)methanone
3RKB	2	Cyclin-dependent kinase 2	12Z	[4-amino-2-(cyclohexylamino)-1,3-thiazol-5-yl](pyridin-3-yl)methanone
3RM6	1.6	Cyclin-dependent kinase 2	18Z	2-{[(2-aminopyrimidin-5-yl)methyl]amino}-5-nitro-4-{[2-(piperazin-1-yl)ethyl]amino}benzamide
3RM7	1.85	Cyclin-dependent kinase 2	19Z	2-[(4-hydroxybenzyl)amino]-5-nitrobenzamide
3RMF	1.75	Cyclin-dependent kinase 2	20Z	4-{[4-amino-5-(naphthalen-2-ylcarbonyl)-1,3-thiazol-2-yl]amino}benzenesulfonamide
3RNI	1.95	Cyclin-dependent kinase 2	21Z	3-[(4-amino-5-benzoyl-1,3-thiazol-2-yl)amino]benzenesulfonamide
3ROC	1.7	MAP kinase 14	29A	3-{5-chloro-4-[(2,4-difluorobenzyl)oxy]-6-oxopyrimidin-1(6H)-yl}-N-(2-hydroxyethyl)-4-methylbenzamide
3ROY	1.75	Cyclin-dependent kinase 2	22Z	4-(hexylamino)-5-nitro-2-[(pyridin-3-ylmethyl)amino]benzamide
3RPO	1.75	Cyclin-dependent kinase 2	24Z	4-({4-carbamoyl-2-nitro-5-[(pyridin-3-ylmethyl)amino]phenyl}amino)butanoic acid
3RPR	1.75	Cyclin-dependent kinase 2	25Z	[4-amino-2-(phenylamino)-1,3-thiazol-5-yl][3-(trifluoromethyl)phenyl]methanone
3RPS	2.3	Casein kinase II	4B0	3-(4,5,6,7-tetrabromo-1H-benzotriazol-1-yl)propan-1-ol
3RPV	1.8	Cyclin-dependent kinase 2	26Z	4-{[4-amino-5-(3-aminobenzoyl)-1,3-thiazol-2-yl]amino}benzenesulfonamide
3RPY	1.9	Cyclin-dependent kinase 2	27Z	4-amino-2-[(4-sulfamoylphenyl)amino]-1,3-thiazole-5-carboxamide
3RTP	2.4	MAP kinase 10	34I	N-[4-cyano-3-(1H-1,2,4-triazol-5-yl)thiophen-2-yl]-2-(2-oxo-3,4-dihydroquinolin-1(2H)-yl)acetamide
3RVG	2.498	Tyrosine-protein kinase JAK2	17P	1-(cyclohexylamino)-7-(1-methyl-1H-pyrazol-4-yl)-5H-pyrido[4,3-b]indole-4-carboxamide
3RWP	1.92	3-phosphoinositide-dependent protein kinase 1	ABQ	[4-amino-7-(propan-2-yl)-7H-pyrrolo[2,3-d]pyrimidin-5-yl](6-{[(3S,4R)-4-(4-fluorophenyl)tetrahydrofuran-3-yl]amino}pyrazin-2-yl)methanone
3RZB	1.9	Cyclin-dependent kinase 2	02Z	4-amino-2-(phenylamino)-1,3-thiazole-5-carboxamide
3S00	1.8	Cyclin-dependent kinase 2	Z60	[4-amino-2-(prop-2-en-1-ylamino)-1,3-thiazol-5-yl](5-chlorothiophen-2-yl)methanone
3S0O	2	Cyclin-dependent kinase 2	50Z	[4-amino-2-(prop-2-en-1-ylamino)-1,3-thiazol-5-yl](pyridin-2-yl)methanone
3S1H	1.75	Cyclin-dependent kinase 2	56Z	4-{[4-amino-5-(4-methoxybenzoyl)-1,3-thiazol-2-yl]amino}benzenesulfonamide
3S2P	2.3	Cyclin-dependent kinase 2	PMU	(3S,4S)-1-{3-[2-amino-6-(propan-2-yl)pyrimidin-4-yl]-4-hydroxyphenyl}pyrrolidine-3,4-diol
3S3I	1.8	MAP kinase 14	CQ0	3-(3-tert-butyl[1,2,4]triazolo[4,3-a]pyridin-7-yl)-N-cyclopropyl-4-methylbenzamide
3S4Q	2.27	MAP kinase 14	NK0	3-[(6-benzoyl-5-methylpyrrolo[2,1-f][1,2,4]triazin-4-yl)amino]-N-cyclopropyl-4-methylbenzamide
3SA0	1.5947	MAP kinase 1	NRA	norathyriol

3SAY	2.231	Glycogen synthase kinase-3 beta	OFT	(3Z)-N,N-diethyl-3-[(3E)-3-(hydroxyimino)-1,3-dihydro-2H-indol-2-ylidene]-2-oxo-2,3-dihydro-1H-indole-5-sulfonamide
3SHE	2.25	MAP kinase-activated protein kinase 3	I85	N-{4-[(3S)-4'-oxo-1',4',5',6'-tetrahydrospiro[piperidine-3,7'-pyrrolo[3,2-c]pyridin]-2'-yl]pyridin-2-yl}-3-(trifluoromethyl)benzamide
3SOC	1.95	Activin receptor type-2A	GVD	[4-({4-[(5-CYCLOPROPYL-1H-PYRAZOL-3-YL)AMINO]QUINAZOLIN-2-YL}IMINO)CYCLOHEXA-2,5-DIEN-1-YL]ACETONITRILE
3SQQ	1.85	Cyclin-dependent kinase 2	99Z	4-{[4-amino-5-(2-methylbenzoyl)-1,3-thiazol-2-yl]amino}benzenesulfonamide
3SRV	1.95	Tyrosine-protein kinase SYK	S19	2-{[(3R,4R)-3-aminotetrahydro-2H-pyran-4-yl]amino}-4-[(4-methylphenyl)amino]pyrimidine-5-carboxamide
3SVV	2.204	Proto-oncogene tyrosine-protein kinase Src	VSP	N-(3-{[4-amino-1-(propan-2-yl)-1H-pyrazolo[3,4-d]pyrimidin-3-yl]methyl}phenyl)ethanesulfonamide
3SW4	1.7	Cyclin-dependent kinase 2	18K	N'-[4-(2-amino-4-methyl-1,3-thiazol-5-yl)pyrimidin-2-yl]-N,N-dimethylbenzene-1,4-diamine
3SW7	1.8	Cyclin-dependent kinase 2	19K	N~4~-[4-(2,4-dimethyl-1,3-thiazol-5-yl)pyrimidin-2-yl]-N~1~,N~1~-dimethyl-2-nitrobenzene-1,4-diamine
3T3U	2.1	Calmodulin-domain protein kinase 1	BK6	3-(6-methoxynaphthalen-2-yl)-1-(piperidin-4-ylmethyl)-1H-pyrazolo[3,4-d]pyrimidin-4-amine
3T3V	2.04	Calmodulin-domain protein kinase 1	BK4	3-(6-methoxynaphthalen-2-yl)-1-(propan-2-yl)-1H-pyrazolo[3,4-d]pyrimidin-4-amine
3T8M	2.5	Phosphatidylinositol 4,5-bisphosphate 3-kinase	3T8	N~2~-{2-chloro-4-[(4-methylpiperazin-1-yl)carbonyl]phenyl}-N~2~,N~8~-dimethyl-4,5-dihydrothieno[3,2-d][1]benzoxepine-2,8-dicarboxamide
3T9T	1.65	Tyrosine-protein kinase ITK/TSK	IAQ	(2Z)-4-(dimethylamino)-N-{7-fluoro-4-[(2-methylphenyl)amino]imidazo[1,5-a]quinoxalin-8-yl}-N-methylbut-2-enamide
3THB	2.5	Serine/threonine-protein kinase PLK1	3TA	9-chloro-2-({5-[3-(dimethylamino)propyl]-2-methylpyridin-3-yl}amino)-5,7-dihydro-6H-pyrimido[5,4-d][1]benzazepine-6-thione
3TIY	1.84	Cyclin-dependent kinase 2	TIY	2,3,4,6-tetrahydroxy-5H-benzo[7]annulen-5-one
3TIZ	2.02	Cyclin-dependent kinase 2	3TI	1-{(E)-[(4-hydroxyphenyl)imino]methyl}naphthalen-2-ol
3TJC	2.4	Tyrosine-protein kinase JAK2	0TP	4-amino-N-methyl-2-[4-(morpholin-4-yl)phenyl]thieno[3,2-c]pyridine-7-carboxamide
3TKH	1.79	Serine/threonine-protein kinase Chk1	07S	1-(morpholin-4-yl)-2-[4-(2-{[5-(pyridin-3-yl)-1,3-thiazol-2-yl]amino}pyridin-4-yl)piperazin-1-yl]ethanone
3TKI	1.6	Serine/threonine-protein kinase Chk1	S25	N-(2-aminoethyl)-5-(2-{[4-(morpholin-4-yl)pyridin-2-yl]amino}-1,3-thiazol-5-yl)pyridine-3-carboxamide
3TTI	2.2	MAP kinase 10	KBI	trans-4-({9-[(3S)-tetrahydrofuran-3-yl]-8-[(2,4,6-trifluorophenyl)amino]-9H-purin-2-yl}amino)cyclohexanol
3TTJ	2.1	MAP kinase 10	JBI	9-cyclopentyl-N~8~-(2-fluorophenyl)-N~2~-(4-methoxyphenyl)-9H-purine-2,8-diamine
3TUB	2.23	Tyrosine-protein kinase SYK	FPU	1-{5-[(6,7-dimethoxyquinolin-4-yl)oxy]pyridin-2-yl}-3-[(1R,2S)-2-phenylcyclopropyl]urea
3TUC	2.1	Tyrosine-protein kinase SYK	FPW	1-benzyl-N-{5-[(6,7-dimethoxyquinolin-4-yl)oxy]pyridin-2-yl}-2-oxo-1,2-dihydropyridine-3-carboxamide
3TUD	2.33	Tyrosine-protein kinase SYK	FPX	N-{4-methyl-3-[8-methyl-7-oxo-2-(phenylamino)-7,8-dihydropyrido[2,3-d]pyrimidin-6-yl]phenyl}-3-(trifluoromethyl)benzamide
3TXO	2.05	Protein kinase C	07U	2-methyl-N~1~-[3-(pyridin-4-yl)-2,6-naphthyridin-1-yl]propane-1,2-diamine
3TZM	1.7	TGF-beta receptor type-1	085	4-[5-(1,3-benzodioxol-5-yl)-4-(pyridin-2-yl)-1H-imidazol-2-yl]benzamide
3U4U	2.2	Casein kinase II	LNH	3-{5-(acetylamino)-3-[3-cyano-7-(cyclopropylamino)pyrazolo[1,5-a]pyrimidin-5-yl]-1H-indol-1-yl}propanoic acid
3U51	2.241	Proto-oncogene tyrosine-protein kinase Src	P4E	
3U6H	2	Hepatocyte growth factor receptor	03X	N-{4-[(6,7-dimethoxyquinolin-4-yl)oxy]-3-fluorophenyl}-1,5-dimethyl-3-oxo-2-phenyl-2,3-dihydro-1H-pyrazole-4-carboxamide
3U6I	2.1	Hepatocyte growth factor receptor	044	N-{3-fluoro-4-[(7-methoxyquinolin-4-yl)oxy]phenyl}-1-[(2R)-2-hydroxypropyl]-5-methyl-3-oxo-2-phenyl-2,3-dihydro-1H-pyrazole-4-carboxamide
3U8W	2.15	MAP kinase 14	09J	3-[3-(2-chloro-6-fluorophenyl)-5-ethyl-6-oxo-5,6-dihydro[1,2,4]triazolo[4,3-b]pyridazin-7-yl]-N-cyclopropyl-4-methylbenzamide
3U9N	1.85	Serine/threonine-protein kinase Chk1	09H	2-(2,3-dihydro-1-benzofuran-5-yl)-N-[2-(piperazin-1-yl)phenyl]-1,3-thiazole-4-carboxamide
3UBD	1.53	Ribosomal protein S6 kinase alpha-3	SL0	5,7-dihydroxy-2-(4-hydroxyphenyl)-4-oxo-4H-chromen-3-yl 3,4-di-O-acetyl-6-deoxy-alpha-L-mannopyranoside
3UGC	1.34	Tyrosine-protein kinase JAK2	046	5-{[6-(acetylamino)pyrimidin-4-yl]oxy}-N-{4-[(4-methylpiperazin-1-yl)methyl]-3-(trifluoromethyl)phenyl}-2,3-dihydro-1H-indole-1-carboxamide
3ULI	2	Cyclin-dependent kinase 2	1N3	1-(aminomethyl)-N-(3-{[6-bromo-2-(4-methoxyphenyl)-3H-imidazo[4,5-b]pyridin-7-yl]amino}propyl)cyclopropanecarboxamide
3UMW	2.08	Proto-oncogene serine/threonine-protein kinase	596	(2Z)-2-(1H-indazol-3-ylmethylidene)-6-methoxy-7-(piperazin-1-ylmethyl)-1-benzofuran-3(2H)-one
3UNJ	1.9001	Cyclin-dependent kinase 2	0BX	4-{[4-(phenylamino)pyrimidin-2-yl]amino}benzoic acid
3UNK	2.1	Cyclin-dependent kinase 2	0BY	4-({4-[(2-chlorophenyl)amino]pyrimidin-2-yl}amino)benzoic acid
3UO4	2.45	Aurora kinase A	0C0	4-{[4-(biphenyl-2-ylamino)pyrimidin-2-yl]amino}benzoic acid
3UOD	2.5002	Aurora kinase A	0C3	4-[(4-{[2-(trifluoromethyl)phenyl]amino}pyrimidin-2-yl)amino]benzoic acid
3UOL	2.4	Aurora kinase A	0C7	N~4~-(2-chlorophenyl)-N~2~-[4-(1H-tetrazol-5-yl)phenyl]pyrimidine-2,4-diamine
3UP2	2.3001	Aurora kinase A	0C8	4-[(4-{[2-(trifluoromethoxy)phenyl]amino}pyrimidin-2-yl)amino]benzoic acid
3UPX	2.27	Calmodulin-domain protein kinase 1	B6A	[2-amino-1-(piperidin-4-ylmethyl)-1H-benzimidazol-6-yl](phenyl)methanone
3UPZ	2.2	Calmodulin-domain protein kinase 1	B5A	1-(piperidin-4-ylmethyl)-1H-pyrazolo[3,4-d]pyrimidin-4-amine
3UQF	2.27	Proto-oncogene tyrosine-protein kinase Src	BK5	3-(6-ethoxynaphthalen-2-yl)-1-(propan-2-yl)-1H-pyrazolo[3,4-d]pyrimidin-4-amine
3UVP	2.4	MAP kinase 14	048	N-{2-fluoro-5-[(5-oxo-6,7,8,9-tetrahydro-5H-benzo[7]annulen-2-yl)amino]phenyl}benzamide
3UVQ	2.2	MAP kinase 14	FS8	N-{5-[(7-{[(2R)-2,3-dihydroxypropyl]oxy}-5-oxo-10,11-dihydro-5H-dibenzo[a,d][7]annulen-2-yl)amino]-2-fluorophenyl}benzamide
3UVR	2.1	MAP kinase 14	06F	1-[3-tert-butyl-1-(4-methylphenyl)-1H-pyrazol-5-yl]-3-{3-[(5-oxo-6,7,8,9-tetrahydro-5H-benzo[7]annulen-2-yl)amino]phenyl}urea
3UZP	1.94	Casein kinase I	0CK	4-[1-cyclohexyl-4-(4-fluorophenyl)-1H-imidazol-5-yl]pyrimidin-2-amine
3V51	1.95	Calmodulin-domain protein kinase 1	I76	3-{6-[(3-chlorobenzyl)oxy]naphthalen-2-yl}-1-(propan-2-yl)-1H-pyrazolo[3,4-d]pyrimidin-4-amine
3V5L	1.86	Tyrosine-protein kinase ITK/TSK	0G1	3-[3-(4-methoxyphenyl)-2-(1H-thieno[3,2-c]pyrazol-3-yl)-1H-indol-6-yl]pentan-3-ol
3V5P	2.1	Calmodulin-domain protein kinase 1	C88	3-(4-methoxy-3-methylphenyl)-1-(piperidin-4-ylmethyl)-1H-pyrazolo[3,4-d]pyrimidin-4-amine
3V5Q	2.2001	NT-3 growth factor receptor	0F4	1-(3-{[(3Z)-2-oxo-3-(1H-pyrrol-2-ylmethylidene)-2,3-dihydro-1H-indol-6-yl]amino}phenyl)-3-[3-(trifluoromethyl)phenyl]urea
3V5T	2.13	Calmodulin-domain protein kinase 1	UW9	1-[(2S)-2-(dimethylamino)-3-methylbutyl]-3-(6-ethoxynaphthalen-2-yl)-1H-pyrazolo[3,4-d]pyrimidin-4-amine

3V8S	2.286	Rho-associated protein kinase 1	0HD	1-(1H-indazol-5-yl)-3-(2-phenylethyl)urea
3V8T	2	Tyrosine-protein kinase ITK/TSK	477	3-{2-[5-(difluoromethyl)-2H-thieno[3,2-c]pyrazol-3-yl]-1H-indol-6-yl}pentan-3-ol
3V8W	2.27	Tyrosine-protein kinase ITK/TSK	0G2	3-[2-(5-phenyl-2H-thieno[3,2-c]pyrazol-3-yl)-1H-indol-6-yl]pentan-3-ol
3VBQ	1.85	Serine/threonine-protein kinase pim-1	0F5	(5~{Z})-5-[[3-[6-[(4-azanylcyclohexyl)amino]pyrazin-2-yl]phenyl]methylidene]-1,3-thiazolidine-2,4-dione
3VBT	2.23	Serine/threonine-protein kinase pim-1	0F9	4-chloro-2-(1H-pyrazol-3-yl)phenol
3VBV	2.08	Serine/threonine-protein kinase pim-1	0FK	8-hydroxyquinoline-2-carboxamide
3VBW	2.48	Serine/threonine-protein kinase pim-1	0FN	1,3-dioxo-2,3-dihydro-1H-indene-2-carbonitrile
3VBX	2.03	Serine/threonine-protein kinase pim-1	0FO	6-bromo-4-hydroxy-2H-chromen-2-one
3VBY	2.27	Serine/threonine-protein kinase pim-1	0FR	furan-2-yl(1H-indol-3-yl)methanone
3VC4	2.23	Serine/threonine-protein kinase pim-1	0FS	(5Z)-5-[3-(trifluoromethyl)benzylidene]-1,3-thiazolidine-2,4-dione
3VF8	2.08	Tyrosine-protein kinase SYK	0JE	3-[5-(5-ethoxy-6-fluoro-1H-benzimidazol-2-yl)-1H-pyrazol-4-yl]-1,1-diethylurea
3VHE	1.55	Vascular endothelial growth factor receptor 2	42Q	1-{2-fluoro-4-[(5-methyl-5H-pyrrolo[3,2-d]pyrimidin-4-yl)oxy]phenyl}-3-[3-(trifluoromethyl)phenyl]urea
3VHK	2.49	Vascular endothelial growth factor receptor 2	BPK	{3-[(5-methyl-2-phenyl-1,3-oxazol-4-yl)methoxy]phenyl}methanol
3VID	2.3	Vascular endothelial growth factor receptor 2	4TT	4,5,6,11-tetrahydro-1H-pyrazolo[4',3':6,7]cyclohepta[1,2-b]indole
3VNT	1.64	Vascular endothelial growth factor receptor 2	0JA	2-chloro-3-(1-cyanocyclopropyl)-N-[5-({2-[(cyclopropylcarbonyl)amino][1,3]thiazolo[5,4-b]pyridin-5-yl}oxy)-2-fluorophenyl]benzamide
3VO3	1.52	Vascular endothelial growth factor receptor 2	0KF	N-[3-({2-[(cyclopropylcarbonyl)amino]imidazo[1,2-b]pyridazin-6-yl}oxy)phenyl]-1,3-dimethyl-1H-pyrazole-5-carboxamide
3VQU	2.4	Dual specificity protein kinase TTK	O22	4-[(4-amino-5-cyano-6-ethoxypyridin-2-yl)amino]benzamide
3VRZ	2.218	Tyrosine-protein kinase HCK	VRZ	1-[4-(4-amino-7-cyclopentyl-7H-pyrrolo[2,3-d]pyrimidin-5-yl)phenyl]-3-benzylurea
3VS1	2.464	Tyrosine-protein kinase HCK	VSA	1-[4-(4-amino-7-cyclopentyl-7H-pyrrolo[2,3-d]pyrimidin-5-yl)phenyl]-3-phenylurea
3VS3	2.17	Tyrosine-protein kinase HCK	VSE	7-[trans-4-(4-methylpiperazin-1-yl)cyclohexyl]-5-(4-phenoxyphenyl)-7H-pyrrolo[2,3-d]pyrimidin-4-amine
3VS6	2.373	Tyrosine-protein kinase HCK	VSH	tert-butyl {4-[4-amino-1-(propan-2-yl)-1H-pyrazolo[3,4-d]pyrimidin-3-yl]-2-methoxyphenyl}carbamate
3VW6	2.4	MAP kinase kinase kinase 5	IM6	4-tert-butyl-N-[6-(1H-imidazol-1-yl)imidazo[1,2-a]pyridin-2-yl]benzamide
3VW8	2.1	Hepatocyte growth factor receptor	DF6	N-({4-[(6,7-dimethoxyquinolin-4-yl)oxy]phenyl}carbamothioyl)-2-phenylacetamide
3W18	2.5	Aurora kinase A	N13	2-{3-[3-(1H-benzimidazol-2-yl)-1H-indazol-6-yl]-1H-pyrazol-5-yl}-N-(3-fluorophenyl)acetamide
3W2C	2.45	Aurora kinase A	N15	2-{4-[3-(1H-benzimidazol-2-yl)-1H-indazol-6-yl]-1H-pyrazol-1-yl}-N-(3-methylbutyl)acetamide
3W2P	2.05	Epidermal growth factor receptor	W2P	N-{2-[4-({3-chloro-4-[3-(trifluoromethyl)phenoxy]phenyl}amino)-5H-pyrrolo[3,2-d]pyrimidin-5-yl]ethyl}-4-(dimethylamino)butanamide
3W2Q	2.2	Epidermal growth factor receptor	HKI	N-(4-{[3-chloro-4-(pyridin-2-ylmethoxy)phenyl]amino}-3-cyano-7-ethoxyquinolin-6-yl)-4-(dimethylamino)butanamide
3W2S	1.9	Epidermal growth factor receptor	W2R	1-{3-[2-chloro-4-({5-[2-(2-hydroxyethoxy)ethyl]-5H-pyrrolo[3,2-d]pyrimidin-4-yl}amino)phenoxy]phenyl}-3-cyclohexylurea
3W32	1.8	Epidermal growth factor receptor	W32	4-({3-chloro-4-[3-(trifluoromethyl)phenoxy]phenyl}amino)-N-[2-(methylsulfonyl)ethyl]-8,9-dihydro-7H-pyrimido[4,5-b]azepine-6-carboxamide
3W33	1.7	Epidermal growth factor receptor	W19	4-{[4-(1-benzothiophen-4-yloxy)-3-chlorophenyl]amino}-N-(2-hydroxyethyl)-8,9-dihydro-7H-pyrimido[4,5-b]azepine-6-carboxamide
3WAR	1.04	Casein kinase II	NIO	NICOTINIC ACID
3WBL	2	Cyclin-dependent kinase 2	PDY	N~7~-(4-ethoxyphenyl)-6-methyl-N~5~-[(3S)-piperidin-3-yl]pyrazolo[1,5-a]pyrimidine-5,7-diamine
3WE4	1.995	Ribosomal protein S6 kinase beta-1	5FI	2-{[4-(5-ethylpyrimidin-4-yl)piperazin-1-yl]methyl}-5-(trifluoromethyl)-1H-benzimidazole
3WF5	2.099	Ribosomal protein S6 kinase beta-1	FZ8	4-[4-(1H-benzimidazol-2-yl)piperidin-1-yl]-1H-pyrazolo[3,4-d]pyrimidine
3WF6	2.031	Ribosomal protein S6 kinase beta-1	FZ9	4-[4-(1H-indol-3-yl)-3,6-dihydropyridin-1(2H)-yl]-1H-pyrazolo[3,4-d]pyrimidine
3WF7	1.85	Ribosomal protein S6 kinase beta-1	FS9	1-(9H-purin-6-yl)-N-[3-(trifluoromethyl)phenyl]piperidine-4-carboxamide
3WF8	1.975	Ribosomal protein S6 kinase beta-1	F76	2-oxo-2-[(4-sulfamoylphenyl)amino]ethyl 7,8,9,10-tetrahydro-6H-cyclohepta[b]quinoline-11-carboxylate
3WF9	2.035	Ribosomal protein S6 kinase beta-1	FS7	(2S)-1-oxo-1-[(4-sulfamoylphenyl)amino]propan-2-yl (2S)-2-methyl-1,2,3,4-tetrahydroacridine-9-carboxylate
3WIK	1.995	Casein kinase II	LCT	N-[5-(4-nitrophenyl)-1,3,4-thiadiazol-2-yl]acetamide
3WZD	1.57	Vascular endothelial growth factor receptor 2	LEV	4-{3-chloro-4-[(cyclopropylcarbamoyl)amino]phenoxy}-7-methoxyquinoline-6-carboxamide
3WZK	2.3	Dual specificity protein kinase TTK	O23	N-cyclopropyl-4-{8-[(thiophen-2-ylmethyl)amino]imidazo[1,2-a]pyrazin-3-yl}benzamide
3ZBX	2.2	Hepatocyte growth factor receptor	6XE	6-[[6-(4-fluorophenyl)-[1,2,4]triazolo[4,3-b][1,2,4]triazin-3-yl]methyl]quinoline
3ZC5	2.2	Hepatocyte growth factor receptor	W9Z	6-{(1S)-1-[6-(1-methyl-1H-pyrazol-4-yl)[1,2,4]triazolo[4,3-b]pyridazin-3-yl]ethyl}quinoline
3ZC6	2.42	Tyrosine-protein kinase JAK3	VFC	N-[(2R)-1-(3-cyanoazetidin-1-yl)-1-oxidanylidene-propan-2-yl]-2-(6-fluoranyl-1-methyl-indazol-3-yl)-5H-pyrrolo[2,3-b]pyrazine-7-carboxamide
3ZCL	1.4	Hepatocyte growth factor receptor	5TF	(S)-3-(1-(1H-pyrrolo(2,3-b)pyridin-3-yl)ethyl)-N-isopropyl-(1,2,4)triazolo(4,3-b)pyridazin-6-amine
3ZDU	2.2	Cyclin-dependent kinase-Like 3	38R	[4-({4-[(3-cyclopentyl-1H-pyrazol-5-yl)amino]pyrimidin-2-yl}amino)phenyl]acetonitrile
3ZEP	2.35	Tyrosine-protein kinase JAK3	1NX	2-[[(3R)-3-acetamido-2,3-dihydro-1H-inden-5-yl]oxy]-N-[(1S)-1-cyclopropylethyl]-5H-pyrrolo[2,3-b]pyrazine-7-carboxamide
3ZLS	2.5	Dual specificity MAP kinase kinase 1	92P	1H-PYRROLO[2,3-B]PYRIDINE-3-CARBOXYLIC ACID
3ZLW	2.12	Dual specificity MAP kinase kinase 1	MT8	(1R)-1-hydroxy-1-methyl-2,3,6,7-tetrahydro-1H,5H-pyrido[3,2,1-ij]quinolin-5-one
3ZLX	2.2	Dual specificity MAP kinase kinase 1	5EZ	7-choro-6-[(3R)-pyrrolidin-3-ylmethoxy]isoquinolin-1(2H)-one
3ZLY	2.11	Dual specificity MAP kinase kinase 1	YSO	3-AMINO-1H-INDAZOLE-4-CARBONITRILE
3ZM4	2.37	Dual specificity MAP kinase kinase 1	22T	7-chloranyl-6-[(3S)-pyrrolidin-3-yl]oxy-2H-isoquinolin-1-one
3ZO1	2	cAMP-dependent protein kinase	SIJ	6-(1,9-DIAZASPIRO[5.5]UNDECAN-9-YL)-9H-PURINE

3ZO2	1.98	cAMP-dependent protein kinase	15I	6-(2,9-Diazaspiro[5.5]undecan-9-yl)-9H-purine
3ZO3	2.1	cAMP-dependent protein kinase	QNI	6-(2,9-DIAZASPIRO[5.5]UNDECAN-2-YL)-9H-PURINE
3ZO4	1.65	cAMP-dependent protein kinase	QWI	6-(4-PHENYL-1,9-DIAZASPIRO[5.5]UNDECAN-9-YL)-9H-PURINE
3ZRK	2.37	Glycogen synthase kinase-3 beta	ZRK	2-(4-PYRIDINYL)FURO[3,2-C]PYRIDIN-4(5H)-ONE
3ZRL	2.48	Proto-oncogene FRAT1	ZRL	7-BROMO-2-PYRIDIN-4-YL-5H-THIENO[3,2-C]PYRIDIN-4-ONE
3ZRM	2.49	Glycogen synthase kinase-3 beta	ZRM	7-(4-HYDROXYPHENYL)-2-PYRIDIN-4-YL-5H-THIENO[3,2-C]PYRIDIN-4-ONE
3ZS5	1.6	MAP kinase 14	SB2	4-[5-(4-FLUORO-PHENYL)-2-(4-METHANESULFINYL-PHENYL)-3H-IMIDAZOL-4-YL]-PYRIDINE
3ZSG	1.89	MAP kinase 14	T75	TAK-715
3ZSH	2.05	MAP kinase 14	469	2-(6-chloro-5-{[(2R,5S)-4-(4-fluorobenzyl)-2,5-dimethylpiperazin-1-yl]carbonyl}-1-methyl-1H-indol-3-yl)-N,N-dimethyl-2-oxoacetamide
3ZTX	1.95	Serine/threonine-protein kinase 12-A	ZTX	2-((4-(4-HYDROXYPIPERIDIN-1-YL)PHENYL)AMINO)-5,11-DIMETHYL-5H-BENZO[E]PYRIMIDO [5,4-B][1,4]DIAZEPIN-6(11H)-ONE
3ZVV	2.5	Phosphatidylinositol 4,5-bisphosphate 3-kinase	XAZ	5,7-dimethylpyrazolo[1,5-a]pyrimidin-2-amine
3ZXZ	1.8	Hepatocyte growth factor receptor	KRW	2-{4-[1-(QUINOLIN-6-YLMETHYL)-1H-[1,2,3]TRIAZOLO[4,5-B]PYRAZIN-6-YL]-1H-PYRAZOL-1-YL}ETHANOL
3ZYA	1.9	MAP kinase 14	2A8	2-AMINO-PHENYLAMINO-DIBENZOSUBERONE
3ZZE	1.87	Hepatocyte growth factor receptor	6XP	(2S)-N'-[(3R)-4-chloro-7-methyl-2-oxo-2,3-dihydro-1H-indol-3-yl]-2-(4-hydroxyphenyl)propanehydrazide
4A4L	2.35	Serine/threonine-protein kinase PLK1	939	1-METHYL-5-(2-{[5-(4-METHYLPIPERAZIN-1-YL)-2-(TRIFLUOROMETHOXY)PHENYL]AMINO}PYRIMIDIN-4-YL)-1H-PYRROLE-3-CARBOXAMIDE
4A4X	2.4	Serine/threonine-protein kinase NEK2	JUP	4-(2-AMINO-5-{4-[(DIMETHYLAMINO)METHYL]THIOPHEN-2-YL}PYRIDIN-3-YL)-2-{(1R)-1-[2-(TRIFLUOROMETHYL)PHENYL]ETHOXY}BENZAMIDE
4A7C	2.3	Proto-oncogene serine/threonine-protein kinase	E46	N-(piperidin-4-ylmethyl)-3-[3-(trifluoromethyloxy)phenyl]-[1,2,3]triazolo[4,5-b]pyridin-5-amine
4A9U	2.48	Serine/threonine-protein kinase Chk2	A9U	2-{4-[(1-BENZYLPIPERIDIN-4-YL)METHOXY]PHENYL}-1H-BENZIMIDAZOLE-6-CARBOXAMIDE
4A9Y	2.2	MAP kinase 14	AQZ	N-(3-{[7-METHOXY-6-(2-PYRROLIDIN-1-YLETHOXY)QUINAZOLIN-4-YL]AMINO}-4-METHYLPHENYL)-2-MORPHOLIN-4-YLISONICOTINAMIDE
4AA0	1.8	MAP kinase 14	AA0	N-[4-methyl-3-[6-(4-methylpiperazin-1-yl)-4-oxidanylidene-quinazolin-3-yl]phenyl]-2-morpholin-4-yl-pyridine-4-carboxamide
4AA4	2.3	MAP kinase 14	QC0	N-[4-METHYL-3-[6-(4-METHYLPIPERAZIN-1-YL)-4-OXIDANYLIDENE-QUINAZOLIN-3-YL]PHENYL]FURAN-3-CARBOXAMIDE
4AA5	2.38	MAP kinase 14	NQB	N-CYCLOPROPYL-4-METHYL-3-[6-(4-METHYLPIPERAZIN-1-YL)-4-OXIDANYLIDENE-QUINAZOLIN-3-YL]BENZAMIDE
4AAC	2.5	MAP kinase 14	AAV	N-isoxazol-3-yl-4-methyl-3-[6-(4-methylpiperazin-1-yl)-4-oxo-quinazolin-3-yl]benzamide
4ACM	1.63	Cyclin-dependent kinase 2	7YG	3-AMINO-6-(4-{[2-(DIMETHYLAMINO)ETHYL]SULFAMOYL}PHENYL)-N-PYRIDIN-3-YLPYRAZINE-2-CARBOXAMIDE
4AFJ	1.98	Proto-oncogene FRAT1	SJJ	5-(4-METHOXYPHENYL)-N-(PYRIDIN-4-YLMETHYL)-1,3-OXAZOLE-4-CARBOXAMIDE
4AG8	1.95	Vascular endothelial growth factor receptor 2	AXI	AXITINIB
4AGU	2.4	Cyclin-dependent kinase-Like 1	D15	N-(5-{[(2S)-4-amino-2-(3-chlorophenyl)butanoyl]amino}-1H-indazol-3-yl)benzamide
4ALW	1.92	Proto-oncogene serine/threonine-protein kinase	HY7	8-BROMANYL-2-[(4-METHYLPIPERAZIN-1-YL)METHYL]-3H-[1]BENZOFURO[3,2-D]PYRIMIDIN-4-ONE
4AN2	2.5	Dual specificity MAP kinase kinase 1	EUI	[3,4-BIS(FLUORANYL)-2-[(2-FLUORANYL-4-IODANYL-PHENYL)AMINO]PHENYL]-[3-OXIDANYL-3-[(2S)-PIPERIDIN-2-YL]AZETIDIN-1-YL]METHANONE
4ANB	2.2	Dual specificity MAP kinase kinase 1	YQY	[3-(AMINOMETHYL)-3-OXIDANYL-AZETIDIN-1-YL]-[3,4-BIS(FLUORANYL)-2-[(2-FLUORANYL-4-IODANYL-PHENYL)AMINO]PHENYL]METHANONE
4ANM	1.7	Casein kinase II	WUL	8-BROMANYL-2-[[(3S)-3-OXIDANYLPYRROLIDIN-1-YL]METHYL]-3H-[1]BENZOFURO[3,2-D]PYRIMIDIN-4-ONE
4ANV	2.13	Phosphatidylinositol 4,5-bisphosphate 3-kinase	751	2-{4-[(4'-METHOXYBIPHENYL-3-YL)SULFONYL]PIPERAZIN-1-YL}-3-(4-METHOXYPHENYL)PYRAZINE
4ANW	2.31	Phosphatidylinositol 4,5-bisphosphate 3-kinase	O92	3-AMINO-6-{4-CHLORO-3-[(2,3-DIFLUOROPHENYL)SULFAMOYL]PHENYL}-N-METHYLPYRAZINE-2-CARBOXAMIDE
4AOI	1.9	Hepatocyte growth factor receptor	4K0	4-[3-(1H-pyrrolo[2,3-b]pyridin-3-ylmethyl)-[1,2,4]triazolo[4,3-b][1,2,4]triazin-6-yl]benzenecarbonitrile
4AOT	2.33	Serine/threonine-protein kinase 10	GW8	1-(4-{methyl[2-({4-[(methylsulfonyl)methyl]phenyl}amino)pyrimidin-4-yl]amino}phenyl)-3-{3-[(4-methylpiperazin-1-yl)carbonyl]phenyl}urea
4AP7	1.8	Hepatocyte growth factor receptor	F47	4-[[6-(4-fluorophenyl)-[1,2,4]triazolo[4,3-b][1,2,4]triazin-3-yl]methyl]phenol
4APP	2.2	Serine/threonine-protein kinase PAK 4	N53	N-[6,6-dimethyl-5-[(2S)-4-methyl-2-(phenylmethyl)piperazin-1-yl]carbonyl-2,4-dihydropyrrolo[3,4-c]pyrazol-3-yl]-3-phenoxy-benzamide
4AQC	1.9	Tyrosine-protein kinase JAK2	88A	8-(4-methylsulfonylphenyl)-N-(4-morpholin-4-ylphenyl)-[1,2,4]triazolo[1,5-a]pyridin-2-amine
4AS0	2.3	Serine/threonine-protein kinase pim-1	RPS	PHTALIMIDE-RUTHENIUM COMPLEX
4ASE	1.83	Vascular endothelial growth factor receptor 2	AV9	TIVOZANIB
4ASX	2.05	Activin receptor type-2A	6OJ	7,8-bis(chloranyl)-9-methyl-3,4-dihydro-2H-pyrido[3,4-b]indol-1-one
4AT3	1.77	BDNF/NT-3 growth factor receptor	LTI	(5Z)-5-(carbamoylimino)-3-[(5R)-6,7,8,9-tetrahydro-5H-benzo[7]annulen-5-ylsulfanyl]-2,5-dihydroisothiazole-4-carboxamide
4AT4	2.36	BDNF/NT-3 growth factor receptor	T6E	1-[4-(4-aminothieno[2,3-d]pyrimidin-5-yl)phenyl]-3-[2-fluoro-5-(trifluoromethyl)phenyl]urea
4AT5	1.71	BDNF/NT-3 growth factor receptor	MUJ	5-{3-methoxy-4-[(4-methoxybenzyl)oxy]benzyl}pyrimidine-2,4-diamine
4AU8	1.9	Cyclin-dependent kinase 5	Z3R	4-(1,3-benzothiazol-2-yl)thiophene-2-sulfonamide
4AUA	2.31	Cyclin-dependent kinase 6	4AU	1H-benzimidazol-2-yl(1H-pyrrol-2-yl)methanone
4AW2	1.7	Serine/threonine-protein kinase MRCK alpha	22E	5,11-dimethyl-1-oxo-2,6-dihydro-1h-pyrido[4,3-b]carbazol-9-yl benzoate
4AW5	2.33	Ephrin type-B receptor 4	30K	(3Z)-5-[(1-ethylpiperidin-4-yl)amino]-3-[(5-methoxy-1H-benzimidazol-2-yl)(phenyl)methylidene]-1,3-dihydro-2H-indol-2-one
4AWI	1.91	MAP kinase 8	AQ2	N-(1,1-dioxidotetrahydro-2H-thiopyran-4-yl)-4-[2-(1-methylethyl)-1H-pyrrolo[2,3-b]pyridin-4-yl]benzenesulfonamide
4AXA	1.9	cAMP-dependent protein kinase	RKD	(2S)-2-(4-chlorophenyl)-2-hydroxy-2-[4-(1H-pyrazol-4-yl)phenyl]ethanaminium
4B0G	2.5	Aurora kinase A	VEK	6-bromo-2-(1-methyl-1H-imidazol-5-yl)-7-{4-[(5-methyl-1,2-oxazol-3-yl)methyl]piperazin-1-yl}-1H-imidazo[4,5-b]pyridine
4B6L	1.9	Serine/threonine-protein kinase PLK3	9ZP	4-[[(4R)-5-cyclopentyl-4-ethyl-3a,4-dihydro-3H-[1,2,4]triazolo[4,3-f]pteridin-7-yl]amino]-N-cyclopropyl-3-methoxy-benzamide
4BB4	1.65	Ephrin type-B receptor 4	32W	N-(2-methoxyethyl)-4-[(6-pyridin-4-ylquinazolin-2-yl)amino]benzamide

4BBE	1.9	Tyrosine-protein kinase JAK2	3O4	N-[4-[2-[(4-morpholin-4-ylphenyl)amino]pyrimidin-4-yl]phenyl]ethanamide
4BBF	2	Tyrosine-protein kinase JAK2	O19	(2R)-N-[4-[2-[(4-morpholin-4-ylphenyl)amino]pyrimidin-4-yl]phenyl]pyrrolidine-2-carboxamide
4BBM	2	Cyclin-dependent kinase-Like 2	TC0	4'-[5-[[3-[(CYCLOPROPYLAMINO)METHYL]PHENYL]AMINO]-1H-PYRAZOL-3-YL]-[1,1'-BIPHENYL]-2,4-DIOL
4BCK	2.052	Cyclin-dependent kinase 2	T3E	3-[[5-cyano-4-[4-methyl-2-(methylamino)-1,3-thiazol-5-yl]pyrimidin-2-yl]amino]benzenesulfonamide
4BCM	2.45	Cyclin-dependent kinase 2	T7Z	4-(4-methyl-2-methylimino-3H-1,3-thiazol-5-yl)-2-[(4-methyl-3-morpholin-4-ylsulfonyl-phenyl)amino]pyrimidine-5-carbonitrile
4BCN	2.1	Cyclin-dependent kinase 2	T9N	2-[(3-hydroxyphenyl)amino]-4-[4-methyl-2-(methylamino)-1,3-thiazol-5-yl]pyrimidine-5-carbonitrile
4BCO	2.05	Cyclin-dependent kinase 2	T6Q	2-[[3-(4-ethanoyl-1,4-diazepan-1-yl)phenyl]amino]-4-[4-methyl-2-(methylamino)-1,3-thiazol-5-yl]pyrimidine-5-carbonitrile
4BCP	2.26	Cyclin-dependent kinase 2	T3C	2-[[3-(1,4-diazepan-1-yl)phenyl]amino]-4-[4-methyl-2-(methylamino)-1,3-thiazol-5-yl]pyrimidine-5-carbonitrile
4BCQ	2.4	Cyclin-dependent kinase 2	TJF	4-[4-methyl-2-(methylamino)-1,3-thiazol-5-yl]-2-{[3-(morpholin-4-ylcarbonyl)phenyl]amino}pyrimidine-5-carbonitrile
4BDB	2.5	Serine/threonine-protein kinase Chk2	ODO	4-[(E)-C-methyl-N-oxidanyl-carbonimidoyl]benzene-1,3-diol
4BDI	2.32	Serine/threonine-protein kinase Chk2	HAU	1-acetyl-N-(5-methylpyridin-2-yl)piperidine-4-carboxamide
4BGH	1.95	Cyclin-dependent kinase 2	3I6	4-((5-BROMO-4-(PROP-2-YN-1-YLAMINO)PYRIMIDIN-2-YL)AMINO)BENZENESULFONAMIDE
4BHN	2.3	MAP kinase kinase kinase 5	BH9	4-tert-butyl-N-[6-(1H-pyrazol-4-yl)imidazo[1,2-a]pyridin-2-yl]benzamide
4BIB	2.43	MAP kinase kinase kinase 5	IEO	3-cyano-4-(piperidin-4-yloxy)-1H-indole-7-carboxamide
4BIE	2.36	MAP kinase kinase kinase 5	IE6	N-(2-aminoethyl)-5-{2-methyl-1H-pyrrolo[2,3-b]pyridin-4-yl}thiophene-2-sulfonamide
4BKY	1.83	Maternal embryonic leucine zipper kinase	82B	3'-{[(4-bromo-1-methyl-1H-pyrrol-2-yl)carbonyl]amino}-N-[(1S)-1-phenyl-2-(pyrrolidin-1-yl)ethyl]-1',4'-dihydro-5'H-spiro[cyclopropane-1,6'-pyrrolo[3,4-c]pyrazole]-5'-carboxamide
4BKZ	2.2	Maternal embryonic leucine zipper kinase	1WS	N-(3-aminopropyl)-8-[(3-fluorophenyl)amino]-2,4,5,7-tetrahydropyrazolo[3,4-e]indazole-3-carboxamide
4BRX	2.05	Focal adhesion kinase 1	KGW	2-(4-(2-methoxy-4-morpholinophenylamino)-1,3,5-triazin-2-ylamino)-N-methylbenzamide
4BZD	1.83	Cyclin-dependent kinase 2	D6I	6-(benzimidazol-1-yl)-N-[5-[3-(dimethylamino)propoxy]pyridin-2-yl]pyrimidin-4-amine
4BZN	1.9	Serine/threonine-protein kinase pim-1	UGX	N-(2,2-dimethylpropyl)-2-[1-oxo-7-(thiophen-3-yl)-1,2,3,4-tetrahydropyrrolo[1,2-a]pyrazin-4-yl]acetamide
4BZO	2.1	Serine/threonine-protein kinase pim-1	676	N-[(1S)-2-AMINO-1-PHENYLETHYL]-2-[(4S)-7-(2-FLUORO-4-PYRIDINYL)-1-OXO-1,2,3,4-TETRAHYDROPYRROLO[1,2-A]PYRAZIN-4-YL]ACETAMIDE
4C2V	1.49	Aurora kinase B-A	YJA	2-[5-[[7-[3-[ethyl(2-hydroxyethyl)amino]propoxy]quinazolin-4-yl]amino]-1H-pyrazol-3-yl]-N-(3-fluorophenyl)ethanamide
4C35	2.19	cAMP-dependent protein kinase	NU3	2-(4-hydroxyphenyl)-1H-benzimidazole-4-carboxamide
4C36	1.98	cAMP-dependent protein kinase	ZO9	4-[1-(cyclopropylmethyl)-1H-benzimidazol-2-yl]-1,2,5-oxadiazol-3-amine
4C37	1.7	cAMP-dependent protein kinase	Z21	4-(6-bromo-1-ethyl-1H-imidazo[4,5-c]pyridin-2-yl)-1,2,5-oxadiazol-3-amine
4C38	1.58	cAMP-dependent protein kinase	VUP	4-(1-ethyl-6-methyl-imidazo[4,5-c]pyridin-2-yl)-1,2,5-oxadiazol-3-amine
4C3F	1.72	Tyrosine-protein kinase Lck	7KW	N-phenyl-4-(5-phenyl-1H-pyrazol-4-yl)pyrimidin-2-amine
4C4F	2.36	Dual specificity protein kinase TTK	7CE	N-(2-methoxyphenyl)-2-(1,3-oxazol-5-yl)-1H-pyrrolo[3,2-c]pyridin-6-amine
4C4J	2.5	Dual specificity protein kinase TTK	X21	tert-butyl 6-{[2-chloro-4-(1-methyl-1H-imidazol-5-yl)phenyl]amino}-2-(1-methyl-1H-pyrazol-4-yl)-1H-pyrrolo[3,2-c]pyridine-1-carboxylate
4C61	2.45	Tyrosine-protein kinase JAK2	LMM	N2-[(1S)-1-(5-fluoropyrimidin-2-yl)ethyl]-7-methyl-N4-(1-methylimidazol-4-yl)thieno[3,2-d]pyrimidine-2,4-diamine
4C7T	2.05	Focal adhesion kinase 1	5RI	N-methyl-2-[[4-[(3,4,5-trimethoxyphenyl)amino]-1,3,5-triazin-2-yl]amino]benzamide
4CCB	2.03	ALK tyrosine kinase receptor	OFG	3-[(1R)-1-[5-fluoranyl-2-(1,2,3-triazol-2-yl)phenyl]ethoxy]-5-(3-methyl-1H-pyrazol-4-yl)pyridin-2-amine
4CCU	2	ALK tyrosine kinase receptor	AWF	2-(5-(6-amino-5-((R)-1-(5-fluoro-2-(2H-1,2,3-triazol-2-yl)phenyl)ethoxy)pyridin-3-yl)-4-methylthiazol-2-yl)propan-2-ol
4CD0	2.23	ALK tyrosine kinase receptor	AWJ	(2R)-2-[5-(6-amino-5-{(1R)-1-[2-(1,3-dihydro-2H-1,2,3-triazol-2-yl)-5-fluorophenyl]ethoxy}pyridin-3-yl)-4-methyl-1,3-thiazol-2-yl]propane-1,2-diol
4CFN	2.2	Cyclin-dependent kinase 2	JYM	6-(cyclohexylmethoxy)-8-(trifluoromethyl)-9H-purin-2-amine
4CFU	2.2	Cyclin-dependent kinase 2	2WC	3-[2-azanyl-6-(cyclohexylmethoxy)-7H-purin-8-yl]-2-methyl-benzoic acid
4CFV	2	Cyclin-dependent kinase 2	75X	3-[2-amino-6-(cyclohexylmethoxy)-7H-purin-8-yl]-2-methylphenol
4CFW	2.45	Cyclin-dependent kinase 2	SQ9	3-[2-amino-6-(cyclohexylmethoxy)-7H-purin-8-yl]-2-methylbenzenesulfonamide
4CKR	2.2	Epithelial discoidin domain-containing receptor 1	DI1	4-[(4-ethylpiperazin-1-yl)methyl]-n-{4-methyl-3-[(2-oxo-2,3-dihydro-1h-indol-5-yl)oxy]phenyl}-3-(trifluoromethyl)benzamide
4CMO	2.05	ALK tyrosine kinase receptor	YPW	2-[(1R)-1-{[3-amino-6-(2-methoxypyridin-3-yl)pyrazin-2-yl]oxy}ethyl]-4-fluoro-N-methylbenzamide
4CMT	1.73	ALK tyrosine kinase receptor	GWH	3-{(1R)-1-[2-(1,3-dihydro-2H-1,2,3-triazol-2-yl)-5-fluorophenyl]ethoxy}-5-[3-(methylsulfonyl)phenyl]pyridin-2-amine
4CMU	1.8	ALK tyrosine kinase receptor	IV7	(10R)-7-amino-12-fluoro-1,3,10,16-tetramethyl-16,17-dihydro-1H-8,4-(metheno)pyrazolo[4,3-h][2,5,11]benzoxadiazacyclotetradecin-15(10H)-one
4CNH	1.9	ALK tyrosine kinase receptor	3U9	3-[(1R)-1-(5-fluoro-2-methoxyphenyl)ethoxy]-5-(1-methyl-1H-1,2,3-triazol-5-yl)pyridin-2-amine
4CQE	2.3	serine/threonine-protein kinase B-Raf	CQE	N-{4-[2-(1-cyclopropylpiperidin-4-yl)-4-(3-{[(2,5-difluorophenyl)sulfonyl]amino}-2-fluorophenyl)-1,3-thiazol-5-yl]pyridin-2-yl}acetamide
4CRL	2.4	Cyclin-dependent kinase 8	C1I	CORTISTATIN A
4CTB	1.79	ALK tyrosine kinase receptor	KVC	(5R)-8-amino-3-fluoro-5,19-dimethyl-20-oxo-5,18,19,20-tetrahydro-11,7-(azeno)pyrido[2',1':2,3]imidazo[4,5-h][2,5,11]benzoxadiazacyclotetradecine-14-carbonitrile
4CTC	2.03	ALK tyrosine kinase receptor	J99	(10R)-7-amino-3-cyclopropyl-12-fluoro-1,10,16-trimethyl-16,17-dihydro-1H-8,4-(metheno)pyrazolo[4,3-h][2,5,11]benzoxadiazacyclotetradecin-15(10H)-one
4CV9	2.5	Dual specificity protein kinase TTK	W2K	4-(4-Methylpiperazin-1-yl)-N-(2-oxo-5-(pyridin-4-yl)-1,2-dihydropyridin-3-yl)benzamide
4CVA	2.5	Dual specificity protein kinase TTK	WBI	N-(6-oxo-1,6-dihydro-3,4'-bipyridin-5-yl)-4-(piperidin-1-yl)benzamide
4D0W	1.77	Tyrosine-protein kinase JAK2	VVQ	5-(2-aminopyrimidin-4-yl)-2-(5-chloro-2-methylphenyl)-1H-pyrrole-3-carboxamide
4D0X	1.82	Tyrosine-protein kinase JAK2	953	5-(2-aminopyrimidin-4-yl)-2-[2-chloro-5-(trifluoromethyl)phenyl]-1H-pyrrole-3-carboxamide
4D1S	1.66	Tyrosine-protein kinase JAK2	BJG	2-(5-chloro-2-methylphenyl)-1-methyl-5-(2-{[4-(4-methylpiperazin-1-yl)phenyl]amino}pyrimidin-4-yl)-1H-pyrrole-3-carboxamide
4D1X	2.1	Cyclin-dependent kinase 2	ESJ	(4S)-2-(6-hydroxy-1,3-benzothiazol-2-yl)-4,5-dihydro-1,3-thiazole-4-carboxylic acid

4D1Z	1.851	Cyclin-dependent kinase 2	WG8	(4S)-2-(8-hydroxyquinolin-2-yl)-4,5-dihydro-1,3-thiazole-4-carboxylic acid
4D2R	2.1	Insulin-like growth factor 1 receptor	DYK	N-{2-methoxy-4-[(1-methylpiperidin-4-yl)oxy]phenyl}-4-(1H-pyrrolo[2,3-c]pyridin-3-yl)pyrimidin-2-amine
4D2V	2.45	Maternal embryonic leucine zipper kinase	45R	7-{[2-methoxy-4-(1H-pyrazol-4-yl)benzoyl]amino}-2,3,4,5-tetrahydro-1H-3-benzazepinium
4D2W	1.92	Maternal embryonic leucine zipper kinase	NF5	4-bromo-N-(2,3,4,5-tetrahydro-1H-3-benzazepin-7-yl)benzamide
4D4V	2.1	Focal adhesion kinase	KB8	6-methyl-4-(piperazin-1-yl)-2-(trifluoromethyl)quinoline
4D5H	1.75	Focal adhesion kinase 1	9RM	6-methyl-5-{[3-(trifluoromethyl)phenyl]amino}-1,2,4-triazin-3(4H)-one
4D9T	2.4	Ribosomal protein S6 kinase alpha-3	0JG	methyl (2S)-3-{4-amino-7-[(1E)-3-hydroxyprop-1-en-1-yl]-5-(4-methylphenyl)-7H-pyrrolo[2,3-d]pyrimidin-6-yl}-2-cyanopropanoate
4D9U	2.4	Ribosomal protein S6 kinase alpha-3	0JH	tert-butyl (2S)-3-[4-amino-7-(3-hydroxypropyl)-5-(4-methylphenyl)-7H-pyrrolo[2,3-d]pyrimidin-6-yl]-2-cyanopropanoate
4DAW	2	Serine/threonine-protein kinase PAK 1	0H2	[1,3-dioxo-6-(pyridin-2-yl-kappaN)-2,3-dihydro-1H-isoindol-5-yl-kappaC~5~][(thioxomethylidene)azanido-kappaN](1,4,7-trithionane-kappa~3~S~1~,S~4~,S~7~)ruthenium
4DCE	2.03	ALK tyrosine kinase receptor	0JF	(3S)-N-(4-methylbenzyl)-1-{2-[(3,4,5-trimethoxyphenyl)amino]pyrimidin-4-yl}piperidine-3-carboxamide
4DEA	2.45	Aurora kinase A	NHI	4,4'-(pyrimidine-2,4-diyldiimino)dibenzoic acid
4DEG	2	Hepatocyte growth factor receptor	0JJ	7-methoxy-N-{[6-(3-methyl-1,2-thiazol-5-yl)[1,2,4]triazolo[4,3-b]pyridazin-3-yl]methyl}-1,5-naphthyridin-4-amine
4DEH	2	Hepatocyte growth factor receptor	0JK	5-phenyl-3-(quinolin-6-ylmethyl)-3,5,6,7-tetrahydro-4H-[1,2,3]triazolo[4,5-c]pyridin-4-one
4DEI	2.05	Hepatocyte growth factor receptor	0JL	3-{(1S)-1-[3-(2-methoxyethoxy)quinolin-6-yl]ethyl}-5-(3-methyl-1,2-thiazol-5-yl)-3,5-dihydro-4H-[1,2,3]triazolo[4,5-c]pyridin-4-one
4DFL	1.98	Tyrosine-protein kinase SYK	0K0	3-amino-6-{3-[(methylsulfonyl)amino]phenyl}-N-(piperidin-4-ylmethyl)pyrazine-2-carboxamide
4DFN	2.48	Tyrosine-protein kinase SYK	0K1	3-amino-6-[3-(1-methyl-1H-pyrazol-4-yl)phenyl]-N-[(1R,2r,3S,5s,7s)-5-hydroxyadamantan-2-yl]pyrazine-2-carboxamide
4DTK	1.86	Serine/threonine-protein kinase pim-1	7LI	(5Z)-5-{2-[(3R)-3-aminopiperidin-1-yl]-3-(propan-2-yloxy)benzylidene}-1,3-thiazolidine-2,4-dione
4DYM	2.42	Activin receptor type-1	IYZ	1-(3-{6-[(CYCLOPROPYLMETHYL)AMINO]IMIDAZO[1,2-B]PYRIDAZIN-3-YL}PHENYL)ETHANONE
4E1Z	2.5	Non-receptor tyrosine-protein kinase TYK2	0MX	N-[5-(4-{[(3-chlorophenyl)sulfonyl]amino}phenyl)-1H-indazol-3-yl]furan-2-carboxamide
4E4L	2	Tyrosine-protein kinase JAK1	0NH	1-[4-methyl-1-(methylsulfonyl)piperidin-4-yl]-1,6-dihydroimidazo[4,5-d]pyrrolo[2,3-b]pyridine
4E4N	1.9	Tyrosine-protein kinase JAK1	0NL	tert-butyl [(1R,3R)-3-(imidazo[4,5-d]pyrrolo[2,3-b]pyridin-1(6H)-yl)cyclopentyl]carbamate
4E5W	1.86	Tyrosine-protein kinase JAK1	0NT	[4-(imidazo[4,5-d]pyrrolo[2,3-b]pyridin-1(6H)-yl)piperidin-1-yl][(2S)-1-(propan-2-yl)pyrrolidin-2-yl]methanone
4E6D	2.22	Tyrosine-protein kinase JAK2	0NU	3-[(3R)-3-(imidazo[4,5-d]pyrrolo[2,3-b]pyridin-1(6H)-yl)piperidin-1-yl]-3-oxopropanenitrile
4E6Q	1.948	Tyrosine-protein kinase JAK2	0NV	1-(1-benzylpiperidin-4-yl)-1,6-dihydroimidazo[4,5-d]pyrrolo[2,3-b]pyridine
4E73	2.27	MAP kinase 8	0NR	methyl 3-(4-{[(1R,2S,3S,5S,7s)-5-aminotricyclo[3.3.1.1~3,7~]dec-2-yl]carbamoyl}benzyl)-4-oxo-1-phenyl-1,4-dihydro-1,8-naphthyridine-2-carboxylate
4E93	1.84	Tyrosine-protein kinase Fes/Fps	GUI	5-CHLORO-N-[2-METHOXY-4-[4-(4-METHYLPIPERAZIN-1-YL)PIPERIDIN-1-YL]PHENYL]-N'-(2-PROPAN-2-YLSULFONYLPHENYL)PYRIMIDINE-2,4-DIAMINE
4EEV	1.8	Hepatocyte growth factor receptor	L1X	N-(3-fluoro-4-{[1-methyl-6-(1H-pyrazol-4-yl)-1H-indazol-5-yl]oxy}phenyl)-1-(4-fluorophenyl)-6-methyl-2-oxo-1,2-dihydropyridine-3-carboxamide
4EH2	2	MAP kinase 14	0OK	3-phenylquinazolin-4(3H)-one
4EH3	2.4	MAP kinase 14	NAR	NARINGENIN
4EH4	2.5	MAP kinase 14	0OL	phenyl(piperidin-1-yl)methanone
4EH5	2	MAP kinase 14	0OM	benzyl pyridine-3-carboxylate
4EH6	2.1	MAP kinase 14	0ON	N-phenylpyridine-3-carboxamide
4EH7	2.1	MAP kinase 14	0OO	(3-phenoxyphenyl)methanol
4EH8	2.2	MAP kinase 14	0OP	[3-(benzyloxy)phenyl]methanol
4EHZ	2.174	Tyrosine-protein kinase JAK1	JAK	2-methyl-1-(piperidin-4-yl)-1,6-dihydroimidazo[4,5-d]pyrrolo[2,3-b]pyridine
4EI4	2.22	Tyrosine-protein kinase JAK1	0Q2	(1R,3R)-3-(2-methylimidazo[4,5-d]pyrrolo[2,3-b]pyridin-1(8H)-yl)cyclohexanol
4EK4	1.26	Cyclin-dependent kinase 2	1CK	4-bromo-N-(5-methyl-1H-pyrazol-3-yl)benzamide
4EK5	1.6	Cyclin-dependent kinase 2	03K	N-(5-cyclopropyl-1H-pyrazol-3-yl)benzene-1,4-dicarboxamide
4EK6	1.52	Cyclin-dependent kinase 2	10K	N-(3-cyclopropyl-1H-pyrazol-5-yl)-2-[4-(thiophen-2-yl)phenyl]acetamide
4EK8	1.7	Cyclin-dependent kinase 2	16K	4-(2,4-dimethyl-1,3-thiazol-5-yl)-N-(3-nitrophenyl)pyrimidin-2-amine
4EKL	2	RAC-alpha serine/threonine-protein kinase	0RF	(2S)-2-(4-chlorophenyl)-1-{4-[(5R,7R)-7-hydroxy-5-methyl-6,7-dihydro-5H-cyclopenta[d]pyrimidin-4-yl]piperazin-1-yl}-3-(propan-2-ylamino)propan-1-one
4EL9	1.55	Ribosomal protein S6 kinase alpha-3	AFE	5,7-dihydroxy-2-(4-hydroxyphenyl)-4-oxo-4H-chromen-3-yl 6-deoxy-alpha-L-mannopyranoside
4EOP	1.99	Cyclin-dependent kinase 2	1RO	(5E)-5-(quinolin-6-ylmethylidene)-2-[(thiophen-2-ylmethyl)amino]-1,3-thiazol-4(5H)-one
4EQC	2.01	Serine/threonine-protein kinase PAK 1	XR1	6-[2-chloro-4-(1,3-thiazol-5-yl)phenyl]-8-ethyl-2-{[4-(4-methylpiperazin-1-yl)phenyl]amino}pyrido[2,3-d]pyrimidin-7(8H)-one
4EQU	2	Serine/threonine-protein kinase 10	G6I	N-{3-[(3-{4-[(4-methoxyphenyl)amino]-1,3,5-triazin-2-yl}pyridin-2-yl)amino]-4-methylphenyl}-3-(trifluoromethyl)benzamide
4ERK	2.2	Extracellular regulated kinase 2	OLO	OLOMOUCINE
4EUU	1.8	Serine/threonine-protein kinase TBK1	BX7	N-(3-{[5-iodo-4-({3-[(thiophen-2-ylcarbonyl)amino]propyl}amino)pyrimidin-2-yl]amino}phenyl)pyrrolidine-1-carboxamide
4EWH	2.5	Activated CDC42 kinase 1	T77	6-{4-[2-(dimethylamino)ethoxy]phenyl}-N-(1,3-dithiolan-2-ylmethyl)-5-phenyl-7H-pyrrolo[2,3-d]pyrimidin-4-amine
4EWQ	2.1	MAP kinase 14	MWL	3-phenyl-4-(pyridin-4-yl)-6-[4-(pyrimidin-2-yl)piperazin-1-yl]pyridazine
4EYJ	2.102	MAP kinase 13	N61	1-(3-tert-butyl-1-methyl-1H-pyrazol-5-yl)-3-[4-(pyridin-4-yloxy)phenyl]urea
4EYM	2.353	MAP kinase 13	0RX	2-(morpholin-4-yl)-N-[4-(pyridin-4-yloxy)phenyl]pyridine-4-carboxamide
4EZ3	2	Cyclin-dependent kinase 2	0S0	4-[(E)-(6-hydroxy-2-oxo-1,2-dihydropyridin-3-yl)diazenyl]benzenesulfonamide
4F4P	2.37	Tyrosine-protein kinase SYK	0SB	N-{6-[3-(piperazin-1-yl)phenyl]pyridin-2-yl}-4-(trifluoromethyl)pyridin-2-amine

4F64	2.05	Fibroblast growth factor receptor 1	0S8	5-bromo-N~4~-[3-(3-methoxypropyl)-1H-pyrazol-5-yl]-N~2~-[(3-methyl-1,2-oxazol-5-yl)methyl]pyrimidine-2,4-diamine
4F65	2.26	Fibroblast growth factor receptor 1	0S9	5-bromo-N~2~-[(3-methyl-1,2-oxazol-5-yl)methyl]-N~4~-[3-(2-phenylethyl)-1H-pyrazol-5-yl]pyrimidine-2,4-diamine
4F6U	2.1	Cyclin-dependent kinase 8	0SR	1-[3-tert-butyl-1-(4-methylphenyl)-1H-pyrazol-5-yl]-3-[3-(morpholin-4-yl)propyl]urea
4F6W	2.39	Cyclin-dependent kinase 8	0SS	N-[3-tert-butyl-1-(4-methylphenyl)-1H-pyrazol-5-yl]-4-[2-({[3-tert-butyl-1-(4-methylphenyl)-1H-pyrazol-5-yl]carbamoyl}amino)ethyl]piperazine-1-carboxamide
4F7S	2.2	Cyclin-dependent kinase 8	0SW	N-(2-phenylethyl)quinazolin-4-amine
4F9B	2.5	Cell division cycle 7-related protein kinase	0SY	2-(pyridin-4-yl)-1,5,6,7-tetrahydro-4H-pyrrolo[3,2-c]pyridin-4-one
4F9C	2.08	Cell division cycle 7-related protein kinase	0SX	8-chloro-2-[(2S)-pyrrolidin-2-yl][1]benzofuro[3,2-d]pyrimidin-4(3H)-one
4F9W	2	MAP kinase 14	LM4	N,N-dimethyl-6-(naphthalen-2-yl)-5-(pyridin-4-yl)pyridazin-3-amine
4F9Y	1.85	MAP kinase 14	LM3	N,N-dimethyl-6-(naphthalen-1-yl)-5-(pyridin-4-yl)pyridazin-3-amine
4FA2	2	MAP kinase 14	SB0	trans-4-[4-(4-fluorophenyl)-5-(2-methoxypyrimidin-4-yl)-1H-imidazol-1-yl]cyclohexanol
4FEQ	2.2	Tyrosine-protein kinase receptor TYRO3	0T8	4-(cyclopentylamino)-N-[3-(2-oxopyrrolidin-1-yl)propyl]-2-{[2-(pyridin-4-yl)ethyl]amino}pyrimidine-5-carboxamide
4FF8	2.4	Tyrosine-protein kinase receptor TYRO3	14S	4-(cyclopentylamino)-2-[(2-methoxybenzyl)amino]-N-[3-(2-oxopyrrolidin-1-yl)propyl]pyrimidine-5-carboxamide
4FIC	2.5	Proto-oncogene tyrosine-protein kinase Src	0UL	6-phenyl[1,2,4]triazolo[1,5-a]pyrazin-2-amine
4FK6	2.2	Tyrosine-protein kinase JAK1	0UJ	N-({1-[(1R,2R,4S)-bicyclo[2.2.1]hept-2-yl]-1,6-dihydroimidazo[4,5-d]pyrrolo[2,3-b]pyridin-2-yl}methyl)methanesulfonamide
4FKG	1.51	Cyclin-dependent kinase 2	4CK	4-[(5-cyclopropyl-1H-pyrazol-3-yl)carbamoyl]benzoic acid
4FKI	1.6	Cyclin-dependent kinase 2	09K	N-(5-cyclopropyl-1H-pyrazol-3-yl)-2-[4-(trifluoromethoxy)phenyl]acetamide
4FKJ	1.63	Cyclin-dependent kinase 2	11K	N-(3-cyclopropyl-1H-pyrazol-5-yl)-2-[4-(2-pyrrolidin-1-ylethoxy)phenyl]acetamide
4FKL	1.26	Cyclin-dependent kinase 2	CK2	4-(2,4-DIMETHYL-1,3-THIAZOL-5-YL)PYRIMIDIN-2-AMINE
4FKO	1.55	Cyclin-dependent kinase 2	20K	2-chloro-N~4~-[4-(2,4-dimethyl-1,3-thiazol-5-yl)pyrimidin-2-yl]-N~1~,N~1~-dimethylbenzene-1,4-diamine
4FKP	1.6	Cyclin-dependent kinase 2	LS5	3-{[4-([AMINO(IMINO)METHYL]AMINOSULFONYL)ANILINO]METHYLENE}-2-OXO-2,3-DIHYDRO-1H-INDOLE
4FKQ	1.75	Cyclin-dependent kinase 2	42K	4-[(2Z)-2-(7-oxidanylidene-3,6-dihydropyrrolo[3,2-e]benzotriazol-8-ylidene)hydrazinyl]benzenesulfonamide
4FKR	1.9	Cyclin-dependent kinase 2	45K	(8Z)-8-{[(2,2-dioxido-1,3-dihydro-2-benzothiophen-5-yl)amino]methylidene}-6,8-dihydro-7H-[1,3]thiazolo[5,4-e]indol-7-one
4FKS	1.55	Cyclin-dependent kinase 2	46K	N-[(4-{[(Z)-(7-oxo-6,7-dihydro-8H-[1,3]thiazolo[5,4-e]indol-8-ylidene)methyl]amino}phenyl)sulfonyl]acetamide
4FKT	1.6	Cyclin-dependent kinase 2	48K	N-[2-(dimethylamino)ethyl]-4-{[(Z)-(2-oxo-1,2-dihydro-3H-indol-3-ylidene)methyl]amino}benzenesulfonamide
4FKU	1.47	Cyclin-dependent kinase 2	60K	(3Z)-2-oxo-3-[2-(4-sulfamoylphenyl)hydrazinylidene]-2,3-dihydro-1H-indole-5-carboxylic acid
4FKV	1.7	Cyclin-dependent kinase 2	61K	(3Z)-N-[2-(1H-imidazol-4-yl)ethyl]-2-oxo-3-[2-(4-sulfamoylphenyl)hydrazinylidene]-2,3-dihydro-1H-indole-5-carboxamide
4FKW	1.8	Cyclin-dependent kinase 2	62K	2-methylpropyl (3Z)-2-oxo-3-[2-(4-sulfamoylphenyl)hydrazinylidene]-2,3-dihydro-1H-indole-5-carboxylate
4FNY	2.45	ALK tyrosine kinase receptor	I3K	N-(4-chlorophenyl)-5-[(6,7-dimethoxyquinolin-4-yl)oxy]-1,3-benzoxazol-2-amine
4FOB	1.9	ALK tyrosine kinase receptor	0US	N-{1-[cis-4-(hydroxymethyl)cyclohexyl]-5-(piperidin-1-ylmethyl)-1H-benzimidazol-2-yl}-3-(prop-2-en-1-ylsulfamoyl)benzamide
4FOC	1.7	ALK tyrosine kinase receptor	0UU	methyl cis-4-[2-(benzoylamino)-6-(piperidin-1-ylmethyl)-1H-benzimidazol-1-yl]cyclohexanecarboxylate
4FOD	2	ALK tyrosine kinase receptor	0UV	4-fluoro-N-{(2E)-6-{[4-(2-hydroxypropan-2-yl)piperidin-1-yl]methyl}-1-[cis-4-(propan-2-ylcarbamoyl)cyclohexyl]-1,3-dihydro-2H-benzimidazol-2-ylidene}benzamide
4FSM	2.3	Serine/threonine-protein kinase Chk1	HK1	4-(6,7-dimethoxy-2,4-dihydroindeno[1,2-c]pyrazol-3-yl)phenol
4FSN	2.1	Serine/threonine-protein kinase Chk1	A58	4-(6-{[(4-METHYLCYCLOHEXYL)AMINO]METHYL}-1,4-DIHYDROINDENO[1,2-C]PYRAZOL-3-YL)BENZOIC ACID
4FSQ	2.4	Serine/threonine-protein kinase Chk1	HK3	4'-(6,7-dimethoxyindeno[1,2-c]pyrazol-3-yl)biphenyl-4-ol
4FSR	2.5	Serine/threonine-protein kinase Chk1	HKC	6,7-dimethoxy-3-[4-(1H-tetrazol-5-yl)phenyl]-1,4-dihydroindeno[1,2-c]pyrazole
4FST	1.9	Serine/threonine-protein kinase Chk1	HK4	4-[(6,7-dimethoxy-2,4-dihydroindeno[1,2-c]pyrazol-3-yl)ethynyl]-2-methoxyphenol
4FSU	2.1	Serine/threonine-protein kinase Chk1	HK5	4-[6-(1H-imidazol-1-ylmethyl)-7-methoxy-2,4-dihydroindeno[1,2-c]pyrazol-3-yl]benzonitrile
4FSW	2.3	Serine/threonine-protein kinase Chk1	HK6	8-chloro-5,10-dihydro-11H-dibenzo[b,e][1,4]diazepin-11-one
4FSY	2.3	Serine/threonine-protein kinase Chk1	HK7	2-(11-oxo-10,11-dihydro-5H-dibenzo[b,e][1,4]diazepin-3-yl)benzamide
4FSZ	2.3	Serine/threonine-protein kinase Chk1	HK8	(3-chloro-11-oxo-10,11-dihydro-5H-dibenzo[b,e][1,4]diazepin-8-yl)acetic acid
4FT0	2.3	Serine/threonine-protein kinase Chk1	HK9	2-methoxy-4-(11-oxo-10,11-dihydro-5H-dibenzo[b,e][1,4]diazepin-3-yl)benzoic acid
4FT3	2.5	Serine/threonine-protein kinase Chk1	H1K	1-(5-chloro-2,4-dimethoxyphenyl)-3-pyrazin-2-ylurea
4FT5	2.4	Serine/threonine-protein kinase Chk1	H2K	1-{5-chloro-2-[(3R)-pyrrolidin-3-yloxy]phenyl}-3-(5-cyanopyrazin-2-yl)urea
4FT7	2.2	Serine/threonine-protein kinase Chk1	H3K	1-{5-bromo-2-[(3R)-3-hydroxypiperidin-1-yl]phenyl}-3-(5-cyanopyrazin-2-yl)urea
4FT9	2.2	Serine/threonine-protein kinase Chk1	H4K	1-(5-cyanopyrazin-2-yl)-3-isoquinolin-3-ylurea
4FTA	2.4	Serine/threonine-protein kinase Chk1	H5K	6-{[(5-cyanopyrazin-2-yl)carbamoyl]amino}naphthalene-2-carboxylic acid
4FTC	2	Serine/threonine-protein kinase Chk1	H6K	1-(5-cyanopyrazin-2-yl)-3-(5-phenyl-1H-pyrazol-3-yl)urea
4FTI	2.2	Serine/threonine-protein kinase Chk1	H7K	methyl 5-(6-{[(cis-4-hydroxycyclohexyl)amino]methyl}-2,4-dihydroindeno[1,2-c]pyrazol-3-yl)furan-2-carboxylate
4FTJ	2.2	Serine/threonine-protein kinase Chk1	H8K	trans-4-({[3-(furan-3-yl)-2,4-dihydroindeno[1,2-c]pyrazol-6-yl]methyl}amino)cyclohexanol
4FTK	2.3	Serine/threonine-protein kinase Chk1	H9K	3-(4'-hydroxybiphenyl-4-yl)-2,4-dihydroindeno[1,2-c]pyrazol-6-ol
4FTL	2.5	Serine/threonine-protein kinase Chk1	H0K	4'-[7-(hydroxymethyl)-2,4-dihydroindeno[1,2-c]pyrazol-3-yl]biphenyl-4-ol
4FTM	1.9	Serine/threonine-protein kinase Chk1	1HK	4-[2-(2,4-dihydroindeno[1,2-c]pyrazol-3-yl)ethyl]-2-methoxyphenol
4FTN	2.02	Serine/threonine-protein kinase Chk1	2HK	4'-{6-methoxy-7-[2-(piperidin-1-yl)ethoxy]-1,2-dihydroindeno[1,2-c]pyrazol-3-yl}biphenyl-4-ol

4FTO	2.1	Serine/threonine-protein kinase Chk1	3HK	4-{7-methoxy-6-[3-(morpholin-4-yl)propoxy]-1,4-dihydroindeno[1,2-c]pyrazol-3-yl}benzonitrile
4FTQ	2	Serine/threonine-protein kinase Chk1	4HK	5-{7-ethyl-6-[(3S)-tetrahydrofuran-3-yloxy]-2,4-dihydroindeno[1,2-c]pyrazol-3-yl}pyridine-2-carbonitrile
4FTR	2.25	Serine/threonine-protein kinase Chk1	5HK	2-[3-(3-methoxy-4-nitrophenyl)-11-oxo-10,11-dihydro-5H-dibenzo[b,e][1,4]diazepin-8-yl]-N,N-dimethylacetamide
4FTT	2.3	Serine/threonine-protein kinase Chk1	6HK	methyl [3-(1-methyl-1H-imidazol-5-yl)-11-oxo-10,11-dihydro-5H-dibenzo[b,e][1,4]diazepin-8-yl]acetate
4FTU	2.1	Serine/threonine-protein kinase Chk1	7HK	methyl [11-oxo-3-(pyridin-4-ylamino)-10,11-dihydro-5H-dibenzo[b,e][1,4]diazepin-8-yl]acetate
4FUL	2.47	Phosphatidylinositol 4,5-bisphosphate 3-kinase	0VU	4-({4-[3-(piperidin-1-ylcarbonyl)phenyl]pyrimidin-2-yl}amino)benzenesulfonamide
4FUX	2.2	MAP kinase 1	E75	trans-4-{[4-(5-methyl-3-phenyl-1,2-oxazol-4-yl)pyrimidin-2-yl]amino}cyclohexanol
4FUY	2	MAP kinase 1	EK2	{4-[4-(3,5-dichlorophenyl)-1H-pyrazol-5-yl]-1H-pyrrol-2-yl}(morpholin-4-yl)methanone
4FV0	2.1	MAP kinase 1	EK3	N-cyclohexyl-4-[3-(4-fluorophenyl)-1H-pyrazol-4-yl]pyridin-2-amine
4FV1	1.99	MAP kinase 1	EK4	N-[(1S)-2-hydroxy-1-phenylethyl]-4-{4-[3-(trifluoromethyl)phenyl]-1H-pyrazol-5-yl}-1H-pyrrole-2-carboxamide
4FV2	2	MAP kinase 1	EK5	4-[4-(3-chlorophenyl)-1H-pyrazol-5-yl]-N-(2,3-dihydro-1-benzofuran-5-ylmethyl)-1H-pyrrole-2-carboxamide
4FV3	2.2	MAP kinase 1	EK6	ethyl N-{2-chloro-4-[5-(5-{[(1S)-1-(3-chloro-4-fluorophenyl)-2-hydroxyethyl]carbamoyl}-1H-pyrrol-3-yl)-1H-pyrazol-4-yl]benzyl}glycinate
4FV4	2.5	MAP kinase 1	EK7	9-(dimethylamino)-2-[(3-hydroxyphenyl)amino]-5,6-dihydrothieno[3,4-h]quinazoline-7-carbonitrile
4FV5	2.4	MAP kinase 1	EK9	N-[(1S)-2-hydroxy-1-phenylethyl]-4-(5-methyl-2-phenylpyrimidin-4-yl)-1H-pyrrole-2-carboxamide
4FV6	2.5	MAP kinase 1	E57	N-[(1S)-1-(3-chlorophenyl)-2-hydroxyethyl]-4-(2-{[(2S)-1-hydroxybutan-2-yl]amino}-5-methylpyrimidin-4-yl)-1H-pyrrole-2-carboxamide
4FV7	1.9	MAP kinase 1	E94	4-(3-chlorophenyl)-5-{2-[(3-hydroxyphenyl)amino]pyrimidin-4-yl}-2-{[2-(piperidin-1-yl)ethyl]amino}thiophene-3-carbonitrile
4FV8	2	MAP kinase 1	E63	6-({4-[(3-cyclopropyl-1H-pyrazol-5-yl)amino]-5-(phenylamino)pyrimidin-2-yl}amino)-1,2-dihydro-3H-indazol-3-one
4FV9	2.11	MAP kinase 1	E71	3-[4-(2,3-difluorophenyl)-1,2-oxazol-5-yl]-5-(pyridin-4-yl)-1H-pyrrolo[2,3-b]pyridine
4FYN	2.318	Tyrosine-protein kinase SYK	0VE	3-[8-({4-[ethyl(2-hydroxyethyl)amino]phenyl}amino)imidazo[1,2-a]pyrazin-5-yl]phenol
4FYO	1.4	Tyrosine-protein kinase SYK	0VF	4-{[(3S)-1-{7-[(3,4-dimethoxyphenyl)amino][1,3]thiazolo[5,4-d]pyrimidin-5-yl}pyrrolidin-3-yl]carbamoyl}benzoic acid
4FZ6	1.85	Tyrosine-protein kinase SYK	0VG	N-{6-[(2S)-2-methylpyrrolidin-1-yl]pyridin-2-yl}-6-phenylimidazo[1,2-b]pyridazin-8-amine
4FZ7	1.75	Tyrosine-protein kinase SYK	0VH	6-{[(1R,2S)-2-aminocyclohexyl]amino}-4-[(6-ethylpyridin-2-yl)amino]pyridazine-3-carboxamide
4G16	2.3	Casein kinase I	0VM	2-[(4-{[3-(trifluoromethyl)pyridin-2-yl]oxy}phenyl)amino]-1H-benzimidazole-6-carbonitrile
4G17	2.1	Casein kinase I	0VN	2-[(4-tert-butylphenyl)amino]-1H-benzimidazole-6-carbonitrile
4G1W	2.45	MAP kinase 8	G1W	methyl 7-fluoro-3-{4-[(2-hydroxyethyl)sulfonyl]benzyl}-4-oxo-1-phenyl-1,4-dihydroquinoline-2-carboxylate
4G2F	1.699	EPH receptor A3	C07	1-amino-5-(5-hydroxy-2-methylphenyl)-7,8,9,10-tetrahydropyrimido[4,5-c]isoquinolin-6(5H)-one
4G31	2.28	Eukaryotic translation initiation factor 2-alpha	0WH	1-[5-(4-amino-7-methyl-7H-pyrrolo[2,3-d]pyrimidin-5-yl)-2,3-dihydro-1H-indol-1-yl]-2-[3-(trifluoromethyl)phenyl]ethanone
4G3E	2.5	NF-kappa-beta-inducing kinase	0WC	(2R)-4-[1-(2-amino-5-chloropyrimidin-4-yl)-2,3-dihydro-1H-indol-6-yl]-2-(1,3-thiazol-2-yl)but-3-yn-2-ol
4G3F	1.642	NF-kappa-beta-inducing kinase	0WB	3-{2-[(5-fluoro-2-hydroxyphenyl)amino]-1,3-thiazol-4-yl}benzonitrile
4G3G	2.5	NF-kappa-beta-inducing kinase	0WA	4-fluoro-2-{[4-(pyridin-4-yl)-1,3-thiazol-2-yl]amino}phenol
4G6N	2	MAP kinase 1	EK0	3-(4-chlorophenyl)-4,5,6,7-tetrahydro-1H-indazole
4G6O	2.2	MAP kinase 1	E28	4-{4-[4-(aminomethyl)-3-(trifluoromethyl)phenyl]-1H-pyrazol-5-yl}-N-(2,3-dihydro-1-benzofuran-5-ylmethyl)-1H-pyrrole-2-carboxamide
4GB9	2.438	Phosphatidylinositol 4,5-bisphosphate 3-kinase	0WR	2-[1-({2-[2-(dimethylamino)-1H-benzimidazol-1-yl]-9-methyl-6-(morpholin-4-yl)-9H-purin-8-yl}methyl)piperidin-4-yl]propan-2-ol
4GCJ	1.42	Cyclin-dependent kinase 2	X64	4-{[4-amino-5-(2-nitrobenzoyl)-1,3-thiazol-2-yl]amino}benzenesulfonamide
4GFG	2.35	Tyrosine-protein kinase SYK	0XF	6-{[(1R,2S)-2-aminocyclohexyl]amino}-4-[(5,6-dimethylpyridin-2-yl)amino]pyridazine-3-carboxamide
4GFM	2.3	Tyrosine-protein kinase JAK2	0X2	2,6-dichloro-N-(2-oxo-2,5-dihydropyridin-4-yl)benzamide
4GG5	2.423	Hepatocyte growth factor receptor	0J3	3-(4-methylpiperazin-1-yl)-N-(3-nitrobenzyl)-7-(trifluoromethyl)quinolin-5-amine
4GG7	2.27	Hepatocyte growth factor receptor	0J8	N-(3-nitrobenzyl)-6-[1-(piperidin-4-yl)-1H-pyrazol-4-yl]-2-(trifluoromethyl)pyrido[2,3-d]pyrimidin-4-amine
4GH2	2.03	Serine/threonine-protein kinase Chk1	HK0	3-(3-methoxy-4-nitrophenyl)-6-[2-(morpholin-4-yl)ethoxy]-5,10-dihydro-11H-dibenzo[b,e][1,4]diazepin-11-one
4GIH	2	Non-receptor tyrosine-protein kinase TYK2	0X5	2,6-dichloro-N-{2-[(cyclopropylcarbonyl)amino]pyridin-4-yl}benzamide
4GII	2.31	Non-receptor tyrosine-protein kinase TYK2	0X6	2,6-dichloro-4-cyano-N-{2-[(cyclopropylcarbonyl)amino]pyridin-4-yl}benzamide
4GJ2	2.4	Non-receptor tyrosine-protein kinase TYK2	0XH	2,6-dichloro-N-[2-({[(1R,2R)-2-fluorocyclopropyl]carbonyl}amino)pyridin-4-yl]benzamide
4GJ3	2.5	Non-receptor tyrosine-protein kinase TYK2	0XP	2,6-dichloro-4-cyano-N-[2-({[(1R,2R)-2-fluorocyclopropyl]carbonyl}amino)pyridin-4-yl]benzamide
4GK2	2.195	EPH receptor A3	L66	7-(5-hydroxy-2-methylphenyl)-8-(2-methoxyphenyl)-1-methyl-1H-imidazo[2,1-f]purine-2,4(3H,8H)-dione
4GK3	1.898	EPH receptor A3	L87	8-butyl-1-methyl-7-(2-methylphenyl)-1H-imidazo[2,1-f]purine-2,4(3H,8H)-dione
4GK4	2.1	EPH receptor A3	L90	8-butyl-1-methyl-7-(5-methyl-1H-indazol-4-yl)-1H-imidazo[2,1-f]purine-2,4(3H,8H)-dione
4GRB	2.15	Casein kinase II	0XG	5-(2-{[4-(dimethylcarbamoyl)phenyl]amino}-4-methoxypyrimidin-5-yl)thiophene-3-carboxylic acid
4GS6	2.2	MAP kinase kinase kinase 7	1FM	(3S,5Z,8S,9S,11E)-8,9,16-trihydroxy-14-methoxy-3-methyl-3,4,9,10-tetrahydro-1H-2-benzoxacyclotetradecine-1,7(8H)-dione
4GU6	1.95	Focal adhesion kinase 1	10N	N-(3-{[(5-cyano-2-phenyl-1H-pyrrolo[2,3-b]pyridin-4-yl)amino]methyl}pyridin-2-yl)-N-methylmethanesulfonamide
4GU9	2.4	Focal adhesion kinase 1	4GU	N-(2-fluorophenyl)-1H-pyrazolo[3,4-d]pyrimidin-4-amine
4GUB	2.2	Casein kinase II	0Y4	N-[5-({3-cyano-7-[(1-methyl-1H-imidazol-4-yl)amino]pyrazolo[1,5-a]pyrimidin-5-yl}amino)-2-methylphenyl]acetamide
4GUE	1.8	Ribosomal protein S6 kinase alpha-3	QCT	2-(3,4-dihydroxyphenyl)-5,7-dihydroxy-4-oxo-4H-chromen-3-yl 6-deoxy-alpha-L-mannopyranoside
4GV1	1.49	RAC-alpha serine/threonine-protein kinase	0XZ	4-amino-N-[(1S)-1-(4-chlorophenyl)-3-hydroxypropyl]-1-(7H-pyrrolo[2,3-d]pyrimidin-4-yl)piperidine-4-carboxamide

4H1J	2	Protein-tyrosine kinase 2-beta	0YH	1-[3-tert-butyl-1-(4-methylphenyl)-1H-pyrazol-5-yl]-3-[3-(4-methoxy-2-methylphenyl)-1H-pyrazol-5-yl]urea
4H1M	1.99	Protein-tyrosine kinase 2-beta	0YJ	7-({[3-tert-butyl-1-(4-methylphenyl)-1H-pyrazol-5-yl]carbamoyl}amino)-N-(propan-2-yl)-1H-indole-2-carboxamide
4HCT	1.48	Tyrosine-protein kinase ITK/TSK	18R	3-{1-[(3R)-1-acryloylpiperidin-3-yl]-4-amino-1H-pyrazolo[3,4-d]pyrimidin-3-yl}-N-(3-tert-butylphenyl)benzamide
4HCU	1.43	Tyrosine-protein kinase ITK/TSK	13L	3-{4-amino-1-[(3R)-1-propanoylpiperidin-3-yl]-1H-pyrazolo[3,4-d]pyrimidin-3-yl}-N-[4-(propan-2-yl)phenyl]benzamide
4HCV	1.48	Tyrosine-protein kinase ITK/TSK	13J	3-{4-amino-1-[(3S)-1-propanoylpiperidin-3-yl]-1H-pyrazolo[3,4-d]pyrimidin-3-yl}-N-[4-(propan-2-yl)phenyl]benzamide
4HGE	2.3	Tyrosine-protein kinase JAK2	15V	N-[1-(3-chlorophenyl)-3-methyl-1H-pyrazol-5-yl]pyrazolo[1,5-a]pyrimidine-3-carboxamide
4HGL	2.4	Casein kinase I	0YO	2-[5-methoxy-2-(quinolin-3-yl)pyrimidin-4-yl]-1,5,6,7-tetrahydro-4H-pyrrolo[3,2-c]pyridin-4-one
4HGT	1.8	Casein kinase I	15G	2-{2-[(3,4-difluorophenoxy)methyl]-5-methoxypyridin-4-yl}-1,5,6,7-tetrahydro-4H-pyrrolo[3,2-c]pyridin-4-one
4HNF	2.07	Casein kinase I	16W	3-[(3-chlorophenoxy)methyl]-1-(tetrahydro-2H-pyran-4-yl)-1H-pyrazolo[3,4-d]pyrimidin-4-amine
4HVB	2.35	Phosphatidylinositol 4,5-bisphosphate 3-kinase	19P	1-{1-[(2S)-2-hydroxypropanoyl]piperidin-4-yl}-3-methyl-8-(6-methylpyridin-3-yl)-1,3-dihydro-2H-imidazo[4,5-c][1,5]naphthyridin-2-one
4HVD	1.85	Tyrosine-protein kinase JAK3	933	2-cyclopropyl-N-[(2S)-3,3-dimethylbutan-2-yl]-5H-pyrrolo[2,3-b]pyrazine-7-carboxamide
4HVH	2.3	Tyrosine-protein kinase JAK3	19R	2-cyclopropyl-N-[(2R)-3-hydroxy-3-methylbutan-2-yl]-5H-pyrrolo[2,3-b]pyrazine-7-carboxamide
4HVI	2.4	Tyrosine-protein kinase JAK3	19S	2-cyclopropyl-N-[(2R)-1-oxo-1-(piperidin-1-yl)propan-2-yl]-5H-pyrrolo[2,3-b]pyrazine-7-carboxamide
4HVS	1.9	Mast/stem cell growth factor receptor Kit	647	5-(1H-pyrrolo[2,3-b]pyridin-3-ylmethyl)-N-[4-(trifluoromethyl)benzyl]pyridin-2-amine
4HYH	1.7	Serine/threonine-protein kinase Chk1	1AM	2-(6-methoxy-1-oxo-1,3-dihydro-2H-isoindol-2-yl)-N-[4-(piperazin-1-yl)pyridin-3-yl]-1,3-thiazole-4-carboxamide
4HYI	1.399	Serine/threonine-protein kinase Chk1	1AO	2-(1H-indazol-1-yl)-N-[2-(piperazin-1-yl)phenyl]-1,3-thiazole-4-carboxamide
4HYS	2.415	MAP kinase 8	1BJ	trans-4-{[4-(1H-indazol-1-yl)pyrimidin-2-yl]amino}cyclohexanol
4HYU	2.152	MAP kinase 8	1BK	trans-4-[(4-{4-[3-(methylsulfonyl)propoxy]-1H-indazol-1-yl}pyrimidin-2-yl)amino]cyclohexanol
4I0R	2.1	Tyrosine-protein kinase SYK	1B4	N-(propan-2-yl)-2-(3,4,5-trimethoxyphenyl)-5H-pyrrolo[2,3-b]pyrazine-7-carboxamide
4I0S	1.98	Tyrosine-protein kinase SYK	1B5	2-(6-chloro-1-methyl-1H-indazol-3-yl)-N-(propan-2-yl)-5H-pyrrolo[2,3-b]pyrazine-7-carboxamide
4I0T	1.7	Tyrosine-protein kinase SYK	1B6	N-tert-butyl-2-(5,6,7,8-tetrahydroimidazo[1,5-a]pyridin-1-yl)-5H-pyrrolo[2,3-b]pyrazine-7-carboxamide
4I22	1.71	Epidermal growth factor receptor	IRE	Gefitinib
4I24	1.8	Epidermal growth factor receptor	1C9	(2E)-N-{4-[(3-chloro-4-fluorophenyl)amino]-7-methoxyquinazolin-6-yl}-4-(piperidin-1-yl)but-2-enamide
4I4E	1.55	Focal adhesion kinase 1	1BQ	[4-(2-hydroxyethyl)piperidin-1-yl][4-(5-methyl-4,4-dioxido-1,5-dihydropyrazolo[4,3-c][2,1]benzothiazin-8-yl)phenyl]methanone
4I5C	2.1	Tyrosine-protein kinase JAK1	C5I	3-oxo-3-[(3R)-3-(pyrrolo[2,3-b][1,2,3]triazolo[4,5-d]pyridin-1(6H)-yl)piperidin-1-yl]propanenitrile
4I5H	1.9	MAP kinase 1	G17	N-{3-[2-(cyclopropylamino)quinazolin-6-yl]-4-methylphenyl}-3-(trifluoromethyl)benzamide
4I5M	1.801	Serine/threonine-protein kinase PLK2	R78	4-{[(7R)-8-cyclopentyl-7-ethyl-5-methyl-6-oxo-5,6,7,8-tetrahydropteridin-2-yl]amino}-3-methoxy-N-(1-methylpiperidin-4-yl)benzamide
4I5P	1.738	Serine/threonine-protein kinase PLK2	1D1	(7R)-8-cyclopentyl-7-ethyl-5-methyl-2-(1H-pyrrol-2-yl)-7,8-dihydropteridin-6(5H)-one
4I6B	1.8	Serine/threonine-protein kinase PLK2	11G	(7R)-8-cyclopentyl-7-ethyl-5-methyl-7,8-dihydropteridin-6(5H)-one
4I6H	1.91	Serine/threonine-protein kinase PLK2	1C8	(7R)-8-cyclopentyl-7-ethyl-5-methyl-2-[2-(1,3-thiazol-4-yl)-1H-imidazol-1-yl]-7,8-dihydropteridin-6(5H)-one
4I6Q	1.85	Tyrosine-protein kinase JAK3	1DT	N-[(1S)-1-cyclopropylethyl]-2-phenoxy-5H-pyrrolo[2,3-b]pyrazine-7-carboxamide
4IBM	1.8	Insulin receptor	IR1	5-(2-phenylpyrazolo[1,5-a]pyridin-3-yl)-3H-pyrazolo[3,4-c]pyridazin-3-one
4IDT	2.4	MAP kinase kinase kinase 14	T28	11-bromo-5,6,7,8-tetrahydropyrimido[4',5':3,4]cyclohepta[1,2-b]indol-2-amine
4IE9	1.92	cAMP-dependent protein kinase	PZX	pyridin-3-ylmethyl 5-methyl-1H-pyrazole-3-carboxylate
4IHP	2.27	Calmodulin-domain protein kinase 1	1FB	1-tert-butyl-3-(3-chlorophenoxy)-1H-pyrazolo[3,4-d]pyrimidin-4-amine
4IJP	2.25	Serine/threonine-protein kinase PLK4	1EH	4-(5-{[(2-chloropyridin-4-yl)methyl]carbamoyl}thiophen-2-yl)-1-benzothiophene-2-carboxamide
4IM0	2.4001	Serine/threonine-protein kinase TBK1	1FV	N-{3-[(5-cyclopropyl-2-{[3-(morpholin-4-ylmethyl)phenyl]amino}pyrimidin-4-yl)amino]propyl}cyclobutanecarboxamide
4ITH	2.25	Receptor-interacting serine/threonine-protein	RCM	(5R)-5-[(7-chloro-1H-indol-3-yl)methyl]-3-methylimidazolidine-2,4-dione
4ITJ	1.8	Receptor-interacting serine/threonine-protein	1HX	N-[(1S)-1-(2-chloro-6-fluorophenyl)ethyl]-5-cyano-1-methyl-1H-pyrrole-2-carboxamide
4IVA	1.5	Tyrosine-protein kinase JAK2	1J5	trans-4-{2-[(1R)-1-hydroxyethyl]imidazo[4,5-d]pyrrolo[2,3-b]pyridin-1(6H)-yl}cyclohexanecarbonitrile
4IVC	2.35	Tyrosine-protein kinase JAK1	1J6	(trans-4-{2-[(1R)-1-hydroxyethyl]imidazo[4,5-d]pyrrolo[2,3-b]pyridin-1(6H)-yl}cyclohexyl)acetonitrile
4IVD	1.93	Tyrosine-protein kinase JAK1	15T	3-(trans-4-{2-[(1R)-1-hydroxyethyl]imidazo[4,5-d]pyrrolo[2,3-b]pyridin-1(6H)-yl}cyclohexyl)propanenitrile
4IWD	1.99	Hepatocyte growth factor receptor	1JC	1-{5-oxo-3-[1-(piperidin-4-yl)-1H-pyrazol-4-yl]-5H-benzo[4,5]cyclohepta[1,2-b]pyridin-7-yl}-N-(pyridin-2-ylmethyl)methanesulfonamide
4IZY	2.3	MAP kinase 8	1J2	trans-4-[(4-{4-[4-(methylsulfonyl)piperidin-1-yl]-1H-indol-1-yl}pyrimidin-2-yl)amino]cyclohexanol
4J52	2.3	Serine/threonine-protein kinase PLK1	1J3	4-{[(7R)-9-cyclopentyl-7-ethenyl-7-fluoro-5-methyl-6-oxo-6,7,8,9-tetrahydro-5H-pyrimido[4,5-b][1,4]diazepin-2-yl]amino}-3-methoxy-N-(4-methylpiperazin-1-yl)benzamide
4J53	2.5	Serine/threonine-protein kinase PLK1	1J4	4-[(9-cyclopentyl-7,7-difluoro-5-methyl-6-oxo-6,7,8,9-tetrahydro-5H-pyrimido[4,5-b][1,4]diazepin-2-yl)amino]-2-fluoro-5-methoxy-N-(1-methylpiperidin-4-yl)benzamide
4J71	2.31	Glycogen synthase kinase-3 beta	1JX	(2R)-2-methyl-1,4-dihydropyrido[2,3-b]pyrazin-3(2H)-one
4J8M	1.853	Aurora kinase A	CJ5	1-[4-[[4-[(5-cyclopentyl-1H-pyrazol-3-yl)amino]pyrimidin-2-yl]amino]phenyl]-3-[3-(trifluoromethyl)phenyl]urea
4JBO	2.493	Aurora kinase A	WPH	1-(4-{2-[(6-{4-[2-(dimethylamino)ethoxy]phenyl}furo[2,3-d]pyrimidin-4-yl)amino]ethyl}phenyl)-3-phenylurea
4JBP	2.45	Aurora Kinase A	YPH	1-(4-{2-[(6-{4-[2-(4-hydroxypiperidin-1-yl)ethoxy]phenyl}furo[2,3-d]pyrimidin-4-yl)amino]ethyl}phenyl)-3-phenylurea
4JBV	1.95	Calmodulin-domain protein kinase 1	V68	1-[(1-methylpiperidin-4-yl)methyl]-3-[6-(2-methylpropoxy)naphthalen-2-yl]pyrazolo[3,4-d]pyrimidin-4-amine
4JI9	2.4	Tyrosine-protein kinase JAK2	1M3	N-tert-butyl-3-[(5-methyl-2-{[4-(4-methylpiperazin-1-yl)phenyl]amino}pyrimidin-4-yl)amino]benzenesulfonamide
4JIA	1.85	Tyrosine-protein kinase JAK2	1K3	5-(4-methoxyphenyl)-N-[4-(4-methylpiperazin-1-yl)phenyl][1,2,4]triazolo[1,5-a]pyridin-2-amine

4JIK	1.9	Serine/threonine-protein kinase Chk1	1KO	2-(4-chlorophenyl)-8-[(3S)-piperidin-3-ylamino]imidazo[1,2-c]pyrimidine-5-carboxamide
4JPS	2.2	Phosphatidylinositol 4,5-bisphosphate 3-kinase	1LT	(2S)-N~1~-{4-methyl-5-[2-(1,1,1-trifluoro-2-methylpropan-2-yl)pyridin-4-yl]-1,3-thiazol-2-yl}pyrrolidine-1,2-dicarboxamide
4JS8	1.94	Dual specificity protein kinase TTK	1PF	4-(cyclohexylmethoxy)-3-{4-[(1-methylpiperidin-4-yl)oxy]phenyl}-2H-indazole
4JT3	2.2	Dual specificity protein kinase TTK	1PH	2-phenyl-N-[3-(3-sulfamoylphenyl)-2H-indazol-5-yl]acetamide
4JX7	2.4	Serine/threonine-protein kinase pim-1	1N6	2-[(trans-4-aminocyclohexyl)amino]-4-{[3-(trifluoromethyl)phenyl]amino}pyrido[4,3-d]pyrimidin-5(6H)-one
4JXF	2.4	Serine/threonine-protein kinase PLK4	631	(1R,2S)-2-{3-[(E)-2-{4-[(dimethylamino)methyl]phenyl}ethenyl]-2H-indazol-6-yl}-5'-methoxyspiro[cyclopropane-1,3'-indol]-2'(1'H)-one
4K0Y	1.954	Serine/threonine-protein kinase pim-1	1OA	N-(4-fluorophenyl)-7-hydroxy-5-(piperidin-4-yl)pyrazolo[1,5-a]pyrimidine-3-carboxamide
4K11	2.3	Proto-oncogene tyrosine-protein kinase Src	0J9	1-tert-butyl-3-(naphthalen-1-yl)-1H-pyrazolo[3,4-d]pyrimidin-4-amine
4K18	2.051	Serine/threonine-protein kinase pim-1	1OB	5-(4-cyanobenzyl)-N-(4-fluorophenyl)-7-hydroxypyrazolo[1,5-a]pyrimidine-3-carboxamide
4K1B	2.082	Serine/threonine-protein kinase pim-1	1OC	N-[5-(2-fluorophenyl)-1H-pyrrolo[2,3-b]pyridin-3-yl]-5-({[(3R,4R)-3-fluoropiperidin-4-yl]methyl}amino)pyrazolo[1,5-a]pyrimidine-3-carboxamide
4K77	2.4	Tyrosine-protein kinase JAK1	1Q4	4-(cyclohexylamino)pyrido[3,4-d]pyrimidin-8(7H)-one
4K9Y	2	Focal adhesion kinase 1	K9Y	1-[4-(6-amino-9H-purin-9-yl)phenyl]-3-[3-tert-butyl-1-(4-methylphenyl)-1H-pyrazol-5-yl]urea
4KAO	2.39	Focal adhesion kinase 1	KAO	1-[3-tert-butyl-1-(4-methylphenyl)-1H-pyrazol-5-yl]-3-[4-(pyridin-3-yl)phenyl]urea
4KB8	1.95	Casein kinase I	1QN	1-{4-[3-(4-fluorophenyl)-1-methyl-1H-pyrazol-4-yl]pyridin-2-yl}-N-methylmethanamine
4KBA	1.98	Casein kinase I	1QM	9-[3-(4-fluorophenyl)-1-methyl-1H-pyrazol-4-yl]-2,3,4,5-tetrahydropyrido[2,3-f][1,4]oxazepine
4KBC	1.98	Casein kinase I	1QJ	{4-[3-(4-fluorophenyl)-1H-pyrazol-4-yl]pyridin-2-yl}methanol
4KBK	2.1	Casein kinase I	1QG	(3S)-3-{4-[3-(4-fluorophenyl)-1-methyl-1H-pyrazol-4-yl]pyridin-2-yl}morpholine
4KD1	1.7	Cyclin-dependent kinase 2	1QK	3-[({3-ethyl-5-[(2S)-2-(2-hydroxyethyl)piperidin-1-yl]pyrazolo[1,5-a]pyrimidin-7-yl}amino)methyl]-1-hydroxypyridinium
4KIN	1.97	MAP kinase 14	1M8	5-(2-chlorophenyl)-N-[5-(cyclopropylcarbamoyl)-2-methylphenyl]thiophene-2-carboxamide
4KIO	2.18	Tyrosine-protein kinase ITK/TSK	G5K	1-[(3S)-3-{[4-(morpholin-4-ylmethyl)-6-([1,3]thiazolo[5,4-b]pyridin-2-ylamino)pyrimidin-2-yl]amino}pyrrolidin-1-yl]prop-2-en-1-one
4KIP	2.27	MAP kinase 14	1R9	2-(2-chlorophenyl)-N-[5-(cyclopropylcarbamoyl)-2-methylphenyl]-1,3-thiazole-5-carboxamide
4KIQ	2.5	MAP kinase 14	1RA	ethyl 6-{[5-(cyclopropylcarbamoyl)-2-methylphenyl]carbamoyl}-1H-indole-1-carboxylate
4KKH	2	MAP kinase 10	1RQ	cyclopropyl[(3R)-3-({4-[6-hydroxy-2-(naphthalen-2-yl)-1H-benzimidazol-1-yl]pyrimidin-2-yl}amino)piperidin-1-yl]methanone
4KNB	2.4	Hepatocyte growth factor receptor	1RU	7-[(1R)-1-(2,6-dichloro-3-fluorophenyl)ethoxy]-3-[1-(piperidin-4-yl)-1H-pyrazol-4-yl]furo[3,2-c]pyridin-6-amine
4KWP	1.25	Casein kinase II	EXX	4,5,6,7-tetrabromo-1-(2-deoxy-beta-D-erythro-pentofuranosyl)-1H-benzimidazole
4L23	2.501	Phosphatidylinositol 4,5-bisphosphate 3-kinase	X6K	3-(4-MORPHOLIN-4-YLPYRIDO[3',2':4,5]FURO[3,2-D]PYRIMIDIN-2-YL)PHENOL
4L7F	1.95	MAP kinase 8	1V5	N-[1-(4-fluorophenyl)cyclopropyl]-4-[(trans-4-hydroxycyclohexyl)amino]imidazo[1,2-a]quinoxaline-8-carboxamide
4L7S	2.03	Tyrosine-protein kinase ITK/TSK	G7K	trans-4-({4-[difluoro(4-fluorophenyl)methyl]-6-[(5-methoxy[1,3]thiazolo[5,4-b]pyridin-2-yl)amino]pyrimidin-2-yl}amino)cyclohexanol
4L8M	2.1	MAP kinase 14	F46	N-[2-fluoro-5-({9-[2-(morpholin-4-yl)ethoxy]-11-oxo-6,11-dihydrodibenzo[b,e]oxepin-3-yl}amino)phenyl]benzamide
4L9I	2.32	Rhodopsin kinase	8PR	Paroxetine
4LL5	2	Serine/threonine-protein kinase pim-1	SK8	6-(4-fluorophenyl)-5-(pyridin-4-yl)-2,3-dihydroimidazo[2,1-b][1,3]thiazole
4LM5	2.25	Serine/threonine-protein kinase pim-1	Q17	2-{4-[(3-aminopropyl)amino]quinazolin-2-yl}phenol
4LOP	2.049	MAP kinase 14	SB4	4-(4-FLUOROPHENYL)-1-(4-PIPERIDINYL)-5-(2-AMINO-4-PYRIMIDINYL)-IMIDAZOLE
4LQM	2.5	Epidermal growth factor receptor	DJK	N-[4-(3-BROMO-PHENYLAMINO)-QUINAZOLIN-6-YL]-ACRYLAMIDE
4LYN	2	Cyclin-dependent kinase 2	1YG	(2S)-N-(5-{[(5-tert-butyl-1,3-oxazol-2-yl)methyl]sulfanyl}-1,3-thiazol-2-yl)-2-phenylpropanamide
4M0Y	1.7	Tyrosine-protein kinase ITK/TSK	M0Y	4-(carbamoylamino)-1-(naphthalen-1-yl)-1H-pyrazole-3-carboxamide
4M0Z	2	Tyrosine-protein kinase ITK/TSK	M0Z	4-(carbamoylamino)-1-(7-methoxynaphthalen-1-yl)-1H-pyrazole-3-carboxamide
4M14	1.55	Tyrosine-protein kinase ITK/TSK	QWS	4-(carbamoylamino)-1-[7-(propan-2-yloxy)naphthalen-1-yl]-1H-pyrazole-3-carboxamide
4M7I	2.34	Eukaryotic translation initiation factor 2-alpha	27D	1-[5-(4-amino-7-methyl-7H-pyrrolo[2,3-d]pyrimidin-5-yl)-4-fluoro-1H-indol-1-yl]-2-(6-methylpyridin-2-yl)ethanone
4M84	1.998	Calmodulin-domain protein kinase 1	21E	5-amino-1-tert-butyl-3-(quinolin-2-yl)-1H-pyrazole-4-carboxamide
4MBI	2.3	Serine/threonine-protein kinase pim-1	26K	N,N-dimethyl-N'-[3-(1H-pyrazol-4-yl)pyrazolo[1,5-a]pyrimidin-5-yl]ethane-1,2-diamine
4MF1	2.113	Tyrosine-protein kinase ITK/TSK	29Y	(1S,2S)-2-{4-[(dimethylamino)methyl]phenyl}-N-[6-(1H-pyrazol-4-yl)-1,3-benzothiazol-2-yl]cyclopropanecarboxamide
4MK0	2.4	Beta-adrenergic receptor kinase 1	29X	5-{[(3S,4R)-4-(4-fluorophenyl)piperidin-3-yl]methoxy}-1H-isoindol-1-one
4MKC	2.01	ALK tyrosine kinase receptor	4MK	5-chloro-N~2~-[5-methyl-4-(piperidin-4-yl)-2-(propan-2-yloxy)phenyl]-N~4~-[2-(propan-2-ylsulfonyl)phenyl]pyrimidine-2,4-diamine
4MQ1	2.35	Dual specificity tyrosine-phosphorylation-	2C3	N-(5-{[(1R)-3-amino-1-(3-chlorophenyl)propyl]carbamoyl}-2-chlorophenyl)-2-methoxy-7-oxo-7,8-dihydropyrido[2,3-d]pyrimidine-6-carboxamide
4MTA	2.2	Serine/threonine-protein kinase pim-1	2D2	2-methyl-5-phenylfuran-3-carboxylic acid
4MXC	1.632	Hepatocyte growth factor receptor	DWF	N-(3-fluoro-4-{[2-({3-[(methylsulfonyl)methyl]phenyl}amino)pyrimidin-4-yl]oxy}phenyl)-N'-(4-fluorophenyl)cyclopropane-1,1-dicarboxamide
4N0S	1.7992	MAP kinase 1	DHC	CAFFEIC ACID
4N4S	2.2	MAP kinase 1	2H1	3-[2-(benzyloxy)-8-methylquinolin-6-yl]-1-(propan-2-yl)-1H-pyrazolo[3,4-d]pyrimidin-4-amine
4N6Z	2.2	Serine/threonine-protein kinase pim-1	2HW	3-amino-N-{4-[(3S)-3-aminopiperidin-1-yl]pyridin-3-yl}pyrazine-2-carboxamide
4N70	2.1	Serine/threonine-protein kinase pim-1	2HX	N-{4-[(3R,4R,5S)-3-amino-4-hydroxy-5-methylpiperidin-1-yl]pyridin-3-yl}-6-(2,6-difluorophenyl)-5-fluoropyridine-2-carboxamide
4NFN	1.42	Tau-tubulin kinase 1	2KC	3-({5-[(4-amino-4-methylpiperidin-1-yl)methyl]pyrrolo[2,1-f][1,2,4]triazin-4-yl}amino)-5-bromophenol
4NJ3	1.848	Cyclin-dependent kinase 2	2KD	6-(3-chlorophenyl)-2-{[(2S)-3-(4-hydroxyphenyl)-1-methoxy-1-oxopropan-2-yl]carbamoyl}quinoline-4-carboxylic acid

4NJD	2.5	Serine/threonine-protein kinase PAK 4	NJD	N-(1H-indazol-5-yl)-N'-[2-(1H-indol-3-yl)ethyl]-6-methoxy-1,3,5-triazine-2,4-diamine
4NKA	2.19	Fibroblast growth factor receptor 1	2K7	N~4~-{3-[2-(3,4-dimethoxyphenyl)ethyl]-1H-pyrazol-5-yl}-N~2~-[(3-methyl-1,2-oxazol-5-yl)methyl]pyrimidine-2,4-diamine
4NKS	2.5	Fibroblast growth factor receptor 1	2M2	N~2~-[(3-methyl-1,2-oxazol-5-yl)methyl]-N~4~-[5-(2-phenylethyl)-1H-pyrazol-3-yl]pyrimidine-2,4-diamine
4NUS	2.39	Ribosomal protein S6 kinase alpha-3	2NK	2,6-difluoro-4-{4-[4-(4-methylpiperazin-1-yl)phenyl]pyridin-3-yl}phenol
4NW5	1.94	Ribosomal protein S6 kinase alpha-3	2NR	7-(2-fluoro-6-methoxyphenyl)-N-(3,4,5-trimethoxyphenyl)-1,3-benzoxazol-2-amine
4NW6	1.74	Ribosomal protein S6 kinase alpha-3	2NS	7-(1H-benzimidazol-7-yl)-N-(3,4,5-trimethoxyphenyl)-1,3-benzoxazol-2-amine
4NWM	2.03	Tyrosine-protein kinase BTK	2P5	4-tert-butyl-N-[2-methyl-3-(6-{[4-(morpholin-4-ylcarbonyl)phenyl]amino}-7H-purin-2-yl)phenyl]benzamide
4O0R	2.4	Serine/threonine-protein kinase PAK 1	7KC	PF-3758309
4O0X	2.483	Serine/threonine-protein kinase PAK 4	2OQ	1-{[1-(4-amino-1,3,5-triazin-2-yl)-2-methyl-1H-benzimidazol-6-yl]ethynyl}cyclohexanol
4O0Y	2.2	Serine/threonine-protein kinase PAK 4	2OO	4-[1-(4-amino-1,3,5-triazin-2-yl)-2-(ethylamino)-1H-benzimidazol-6-yl]-2-methylbut-3-yn-2-ol
4O2P	2.1	Proto-oncogene tyrosine-protein kinase Src	11V	1-[(2R)-2-chloro-2-phenylethyl]-6-{[2-(morpholin-4-yl)ethyl]sulfanyl}-N-phenyl-1H-pyrazolo[3,4-d]pyrimidin-4-amine
4O6E	1.95	MAP kinase 1	2SH	N-[(1S)-1-(3-chloro-4-fluorophenyl)-2-hydroxyethyl]-2-(tetrahydro-2H-pyran-4-ylamino)-5,8-dihydropyrido[3,4-d]pyrimidine-7(6H)-carboxamide
4O6L	2.38	Dual specificity protein kinase TTK	2QK	N-[(1R)-1-(2-chlorophenyl)propyl]-3-{4-[(1-methylpiperidin-4-yl)oxy]phenyl}-1H-indazole-5-carboxamide
4O91	2.393	MAP kinase kinase kinase 7	NG2	N-{4-[(4-ethylpiperazin-1-yl)methyl]-3-(trifluoromethyl)phenyl}-4-methyl-3-(1H-pyrrolo[2,3-b]pyridin-4-yloxy)benzamide
4OBO	2.1	MAP kinase kinase kinase kinase 4	2QV	6-(3-chlorophenyl)quinazolin-4-amine
4OBP	2.27	MAP kinase kinase kinase kinase 4	2QU	6-(2-fluoropyridin-4-yl)pyrido[3,2-d]pyrimidin-4-amine
4OBQ	2.19	MAP kinase kinase kinase kinase 4	2QT	N-[3-(4-aminoquinazolin-6-yl)-5-fluorophenyl]-2-(pyrrolidin-1-yl)acetamide
4ONA	2.4	Calmodulin-domain protein kinase 1	UW1	5-amino-1-tert-butyl-3-(7-ethoxyquinolin-3-yl)-1H-pyrazole-4-carboxamide
4OT5	1.55	Tyrosine-protein kinase BTK	481	4-tert-butyl-N-{3-[8-({4-[(4-methylpiperazin-1-yl)carbonyl]phenyl}amino)imidazo[1,2-a]pyrazin-6-yl]phenyl}benzamide
4OT6	2.05	Tyrosine-protein kinase BTK	2V1	4-(methylsulfonyl)-N-[3-(8-{[4-(morpholin-4-ylcarbonyl)phenyl]amino}imidazo[1,2-a]pyrazin-6-yl)phenyl]benzamide
4OTH	1.8	Serine/threonine-protein kinase N1	DRN	BISINDOLYLMALEIMIDE IX
4OTQ	1.55	Tyrosine-protein kinase BTK	2V2	1-{5-[3-(7-tert-butyl-4-oxoquinazolin-3(4H)-yl)-2-methylphenyl]-1-methyl-2-oxo-1,2-dihydropyridin-3-yl}-3-methylurea
4OTR	1.95	Tyrosine-protein kinase BTK	2V3	6-cyclopropyl-2-[3-(5-{[5-(4-ethylpiperazin-1-yl)pyridin-2-yl]amino}-1-methyl-6-oxo-1,6-dihydropyridin-3-yl)-2-(hydroxymethyl)phenyl]-8-fluoroisoquinolin-1(2H)-one
4P4C	1.599	EPH receptor A3	25Q	2-amino-1-(3-methoxyphenyl)-1H-pyrrolo[2,3-b]quinoxaline-3-carboxamide
4P5Q	1.35	Ephrin type-A receptor 3	Q0B	2-amino-1-(2-chlorophenyl)-N-(3-ethoxypropyl)-1H-pyrrolo[2,3-b]quinoxaline-3-carboxamide
4P5Z	2.002	Ephrin type-A receptor 3	Q7M	2-amino-1-[4-({[3-(trifluoromethyl)phenyl]carbamoyl}amino)phenyl]-1H-pyrrolo[2,3-b]quinoxaline-3-carboxamide
4P90	2.49	Serine/threonine-protein kinase PAK 1	2K0	[2-chloro-5-(hydroxymethyl)phenyl]{5-[1-(piperidin-4-yl)-1H-pyrazol-4-yl]-1H-pyrrolo[2,3-b]pyridin-3-yl}methanone
4PMM	2	High affinity nerve growth factor receptor	31V	N-(3-cyclopropyl-1-phenyl-1H-pyrazol-5-yl)-2-{4-[3-methoxy-4-(4-methyl-1H-imidazol-1-yl)phenyl]-1H-1,2,3-triazol-1-yl}acetamide
4PMP	1.8	High affinity nerve growth factor receptor	31W	1-cyclopropyl-1-[3-(1,3-thiazol-2-yl)benzyl]-3-[4-(trifluoromethoxy)phenyl]urea
4PMT	2.1	High affinity nerve growth factor receptor	31Y	N~4~-[4-(morpholin-4-yl)phenyl]-N~6~-(pyridin-3-ylmethyl)pyrido[3,2-d]pyrimidine-4,6-diamine
4PNI	1.85	Rhodopsin kinase	KQQ	3-[(2-{[1-(N,N-dimethylglycyl)-6-methoxy-4,4-dimethyl-1,2,3,4-tetrahydroquinolin-7-yl]amino}-7H-pyrrolo[2,3-d]pyrimidin-4-yl)amino]thiophene-2-carboxamide
4PQN	1.71	Tyrosine-protein kinase ITK/TSK	2W6	N-{1-[(1S)-3-(dimethylamino)-1-phenylpropyl]-1H-pyrazol-4-yl}-6,6-dimethyl-4,5,6,7-tetrahydro-1H-indazole-3-carboxamide
4PTE	2.033	Glycogen synthase kinase-3 beta	2WF	N-[4-(isoquinolin-7-yl)pyridin-2-yl]cyclopropanecarboxamide
4PTG	2.361	Glycogen synthase kinase-3 beta	2WG	2-{2-[(cyclopropylcarbonyl)amino]pyridin-4-yl}-4-methoxypyrimidine-5-carboxamide
4PUZ	2.085	Tyrosine-protein kinase SYK	CG9	6-(1H-indazol-6-yl)-N-[4-(morpholin-4-yl)phenyl]imidazo[1,2-a]pyrazin-8-amine
4PV0	2	Tyrosine-protein kinase SYK	CG4	4-[(3-{8-[(3,4-dimethoxyphenyl)amino]imidazo[1,2-a]pyrazin-6-yl}benzoyl)amino]benzoic acid
4PX6	1.6	Tyrosine-protein kinase SYK	2X6	7-{[(1R,2S)-2-aminocyclohexyl]amino}-5-(1H-indol-7-ylamino)pyrido[4,3-d]pyrimidin-4(3H)-one
4PY1	2.16	Non-receptor tyrosine-protein kinase TYK2	2YK	6-[(2,5-dimethoxyphenyl)sulfanyl]-3-(1-methyl-1H-pyrazol-4-yl)[1,2,4]triazolo[4,3-b]pyridazine
4Q9S	2.07	Focal adhesion kinase 1	30G	3,5-dihydro[1,2,4]triazino[3,4-c][1,4]benzoxazin-2(1H)-one
4QD6	2.45	Tyrosine-protein kinase ITK/TSK	30T	trans-4-({6-[(5-phenyl-1H-pyrazol-3-yl)amino]-4-(phenylsulfonyl)pyridin-2-yl}amino)cyclohexanol
4QMM	1.852	Serine/threonine-protein kinase 24	35R	1-cyclopropyl-3-{3-[5-(morpholin-4-ylmethyl)-1H-benzimidazol-2-yl]-1H-pyrazol-4-yl}urea
4QMN	2.091	Serine/threonine-protein kinase 24	DB8	4-[(2,4-dichloro-5-methoxyphenyl)amino]-6-methoxy-7-[3-(4-methylpiperazin-1-yl)propoxy]quinoline-3-carbonitrile
4QMO	1.898	Serine/threonine-protein kinase 24	34L	(8Z)-8-(1H-imidazol-5-ylmethylidene)-6,8-dihydro-7H-[1,3]thiazolo[5,4-e]indol-7-one
4QMQ	1.769	Serine/threonine-protein kinase 24	34U	1-{2-[5-(2-methoxyethoxy)-1H-benzimidazol-1-yl]quinolin-8-yl}piperidin-4-amine
4QMT	1.5	Serine/threonine-protein kinase 24	H1N	N-[2-OXO-3-((E)-PHENYL{[4-(PIPERIDIN-1-YLMETHYL)PHENYL]IMINO}METHYL)-2,6-DIHYDRO-1H-INDOL-5-YL]ETHANESULFONAMIDE
4QMV	2.4	Serine/threonine-protein kinase 24	34W	N-{2-[(1S,4R)-6-{[4-(cyclobutylamino)-5-(trifluoromethyl)pyrimidin-2-yl]amino}-1,2,3,4-tetrahydro-1,4-epiminonaphthalen-9-yl]-2-oxoethyl}acetamide
4QMW	1.6	Serine/threonine-protein kinase 24	KS1	1-cyclopentyl-3-(1H-pyrrolo[2,3-b]pyridin-5-yl)-1H-pyrazolo[3,4-d]pyrimidin-4-amine
4QMX	1.882	Serine/threonine-protein kinase 24	H8H	N-(5-CHLORO-1,3-BENZODIOXOL-4-YL)-7-[2-(4-METHYLPIPERAZIN-1-YL)ETHOXY]-5-(TETRAHYDRO-2H-PYRAN-4-YLOXY)QUINAZOLIN-4-AMINE
4QO9	2.2	Serine/threonine-protein kinase 24	627	N-[(3E)-5-[(2R)-2-METHOXY-2-PHENYLACETYL]PYRROLO[3,4-C]PYRAZOL-3(5H)-YLIDENE]-4-(4-METHYLPIPERAZIN-1-YL)BENZAMIDE
4QP2	2.23	MAP kinase 1	36R	5-chloro-1,3-benzoxazol-2-amine
4QP4	2.2	MAP kinase 1	36O	N-cyclohexyl-9H-purin-6-amine
4QP7	2.249	MAP kinase 1	363	2-(1H-pyrazol-4-yl)-5H-pyrrolo[2,3-b]pyrazine
4QP8	2.446	MAP kinase 1	362	2-(1H-pyrazol-4-yl)-7-(pyridin-3-yl)-5H-pyrrolo[2,3-b]pyrazine

4QP9	2.001	MAP kinase 1	35X	7-(1-propyl-1H-pyrazol-4-yl)-2-(pyridin-4-yl)-5H-pyrrolo[2,3-b]pyrazine
4QPS	1.8	Tyrosine-protein kinase JAK3	37Q	N-{1-[6-(phenylamino)pyrazin-2-yl]-1H-benzimidazol-6-yl}prop-2-enamide
4QQ5	2.203	Fibroblast growth factor receptor 4	37O	N-(4-{[3-(3,5-dimethoxyphenyl)-7-{[4-(4-methylpiperazin-1-yl)phenyl]amino}-2-oxo-3,4-dihydropyrimido[4,5-d]pyrimidin-1(2H)-yl]methyl}phenyl)propanamide
4QT1	2.4	Tyrosine-protein kinase JAK3	3C9	1-{(3S)-1-[(2-methylpropyl)sulfonyl]piperidin-3-yl}-3-(5H-pyrrolo[2,3-b]pyrazin-2-yl)urea
4QTA	1.45	MAP kinase 1	38Z	(3R)-1-(2-oxo-2-{4-[4-(pyrimidin-2-yl)phenyl]piperazin-1-yl}ethyl)-N-[3-(pyridin-4-yl)-2H-indazol-5-yl]pyrrolidine-3-carboxamide
4QTE	1.5	MAP kinase 1	390	4-{2-[(2-chloro-4-fluorophenyl)amino]-5-methylpyrimidin-4-yl}-N-[(1S)-1-(3-chlorophenyl)-2-hydroxyethyl]-1H-pyrrole-2-carboxamide
4QYE	2.05	Serine/threonine-protein kinase Chk1	3DL	4-[6-(3-hydroxyphenyl)pyrazin-2-yl]benzoic acid
4QYF	2.15	Serine/threonine-protein kinase Chk1	3DV	4-[3-amino-6-(3-hydroxyphenyl)pyrazin-2-yl]benzoic acid
4QYG	1.75	Serine/threonine-protein kinase Chk1	3DW	3-[4-(4-methylpiperazin-1-yl)phenyl]-9H-pyrrolo[2,3-b:5,4-c']dipyridine-6-carboxylic acid
4QYH	1.9	Serine/threonine-protein kinase Chk1	3DX	3-[4-(4-methylpiperazin-1-yl)phenyl]-9H-pyrrolo[2,3-b:5,4-c']dipyridine-6-carbonitrile
4QYY	1.65	MAP kinase 1	3G7	(3R)-1-{2-[4-(4-acetylphenyl)piperazin-1-yl]-2-oxoethyl}-N-(3-chloro-4-hydroxyphenyl)pyrrolidine-3-carboxamide
4R1V	1.2	Hepatocyte growth factor receptor	3E8	3-[1-(3-{5-[(1-methylpiperidin-4-yl)methoxy]pyrimidin-2-yl}benzyl)-6-oxo-1,6-dihydropyridazin-3-yl]benzonitrile
4R1Y	2	Hepatocyte growth factor receptor	3EH	3-(diethylamino)propyl (3-{[5-(3,4-dimethoxyphenyl)-2-oxo-2H-1,3,4-thiadiazin-3(6H)-yl]methyl}phenyl)carbamate
4R3C	2.06	MAP kinase 14	3GF	6-(4-methylpiperazin-1-yl)-3-(naphthalen-2-yl)-4-(pyridin-4-yl)pyridazine
4R6V	2.353	Fibroblast growth factor receptor 4	FI3	N-[4-({[(2,6-dichloro-3,5-dimethoxyphenyl)carbamoyl](6-{[4-(4-methylpiperazin-1-yl)phenyl]amino}pyrimidin-4-yl)amino}methyl)phenyl]propanamide
4RC2	2.096	Serine/threonine-protein kinase pim-1	3O8	1-amino-5,8-dihydroxy-4-({2-[(2-hydroxyethyl)amino]ethyl}amino)anthracene-9,10-dione
4RC3	2.338	Serine/threonine-protein kinase pim-1	3OA	1,4-dihydroxy-5,8-bis{[2-(2-hydroxyethoxy)ethyl]amino}anthracene-9,10-dione
4RFM	2.1	Tyrosine-protein kinase ITK/TSK	3P6	(4aS,5aR)-N-{1-[(R)-[(2R)-1,1-dioxidotetrahydro-2H-thiopyran-2-yl](phenyl)methyl]-1H-pyrazol-4-yl}-5,5-difluoro-5a-methyl-1,4,4a,5,5a,6-hexahydrocyclopropa[f]indazole-3-carboxamide
4RFY	1.7	Tyrosine-protein kinase BTK	3OU	6-(dimethylamino)-2-[2-(hydroxymethyl)-3-(1-methyl-5-{[5-(morpholin-4-ylcarbonyl)pyridin-2-yl]amino}-6-oxo-1,6-dihydropyridin-3-yl)phenyl]-3,4-dihydroisoquinolin-1(2H)-one
4RFZ	1.17	Tyrosine-protein kinase BTK	3OV	6-(dimethylamino)-8-fluoro-2-[2-(hydroxymethyl)-3-(1-methyl-5-{[5-(morpholin-4-ylcarbonyl)pyridin-2-yl]amino}-6-oxo-1,6-dihydropyridin-3-yl)phenyl]isoquinolin-1(2H)-one
4RG0	2.5	Tyrosine-protein kinase BTK	3P0	2-{8-fluoro-2-[2-(hydroxymethyl)-3-(1-methyl-5-{[5-(4-methylpiperazin-1-yl)pyridin-2-yl]amino}-6-oxo-1,6-dihydropyridin-3-yl)phenyl]-1-oxo-1,2,3,4-tetrahydroisoquinolin-6-yl}-2-methylpropanenitrile
4RJ3	1.63	Cyclin-dependent kinase 2	3QS	1-cyclopentyl-N-[2-(4-methoxypiperidin-1-yl)pyrimidin-4-yl]-1H-pyrrolo[3,2-c]pyridin-6-amine
4RLL	1.85	Casein kinase II	E91	4-[(E)-(9H-fluoren-9-ylidenehydrazinylidene)methyl]benzoic acid
4RMZ	2.2	Interleukin-1 receptor-associated kinase 4	T20	3-nitro-N-[1-phenyl-5-(piperidin-1-ylmethyl)-1H-benzimidazol-2-yl]benzamide
4RSS	1.83	Tyrosine-protein kinase SYK	4MG	1-[(3-methyl-1-{2-[(1,2,3-trimethyl-1H-indol-5-yl)amino]pyrimidin-4-yl}-1H-pyrazol-4-yl)methyl]azetidin-3-ol
4RVK	1.85	Serine/threonine-protein kinase Chk1	3XK	N-[3-(6-cyano-9H-pyrrolo[2,3-b:5,4-c']dipyridin-3-yl)phenyl]acetamide
4RVL	1.85	Serine/threonine-protein kinase Chk1	3XL	3-(2-hydroxyphenyl)-9H-pyrrolo[2,3-b:5,4-c']dipyridine-6-carbonitrile
4RVM	1.86	Serine/threonine-protein kinase Chk1	3X7	3-[4-(piperidin-1-ylmethyl)phenyl]-9H-pyrrolo[2,3-b:5,4-c']dipyridine-6-carbonitrile
4RVT	2.4	MAP kinase kinase kinase kinase 4	3XM	3-hexanoyl-4-hydroxy-5-(4-hydroxyphenyl)pyridin-2(1H)-one
4RWL	2.193	Fibroblast growth factor receptor 1	3ZC	6-({7-[(1-aminocyclopropyl)methoxy]-6-methoxyquinolin-4-yl}oxy)-N-methylnaphthalene-1-carboxamide
4RX5	1.356	Tyrosine-protein kinase BTK	3YO	N-(6-fluoro-2-methyl-3-{5-[(5-methyl-4,5,6,7-tetrahydropyrazolo[1,5-a]pyrazin-2-yl)amino]-6-oxo-1,6-dihydropyridazin-3-yl}phenyl)-1-benzothiophene-2-carboxamide
4RX7	1.8	Tyrosine-protein kinase SYK	3YV	3-{[(1R,2S)-2-aminocyclohexyl]amino}-5-{[3-(2H-1,2,3-triazol-2-yl)phenyl]amino}-1,2,4-triazine-6-carboxamide
4RX8	1.59	Tyrosine-protein kinase SYK	3YX	3-{[(1R,2S)-2-aminocyclohexyl]amino}-5-(1H-indol-7-ylamino)-1,2,4-triazine-6-carboxamide
4RX9	1.75	Tyrosine-protein kinase SYK	3YT	2-{[(1R,2S)-2-aminocyclohexyl]amino}-4-{[3-(2H-1,2,3-triazol-2-yl)phenyl]amino}pyrimidine-5-carboxamide
4TN6	2.41	Casein kinase I	PFO	4-{4-(4-fluorophenyl)-1-[1-(1,2-oxazol-3-ylmethyl)piperidin-4-yl]-1H-imidazol-5-yl}pyrimidin-2-amine
4TNB	2.113	G protein-coupled receptor kinase 5	SGV	SANGIVAMYCIN
4TW9	2.4	Casein kinase I	386	N-(2,2-difluoro-5H-[1,3]dioxolo[4,5-f]benzimidazol-6-yl)-2-{[2-(trifluoromethoxy)benzoyl]amino}-1,3-thiazole-4-carboxamide
4TWC	1.7	Casein kinase I	37J	2-{[2-(trifluoromethoxy)benzoyl]amino}-N-[6-(trifluoromethyl)-1H-benzimidazol-2-yl]-1,3-thiazole-4-carboxamide
4TWO	2.047	Ephrin type-A receptor 3	37W	5-{[3-carbamoyl-4-(3,4-dimethylphenyl)-5-methylthiophen-2-yl]amino}-5-oxopentanoic acid
4TXC	1.951	Death-associated protein kinase 1	38G	4-(3-{3-[(R)-{[2-(dimethylamino)ethyl]amino}(hydroxy)methyl]phenyl}imidazo[1,2-b]pyridazin-6-yl)-2-methoxyphenol
4TZR	2	Calmodulin-domain protein kinase 1	UW2	1-{4-amino-3-[2-(cyclopropyloxy)quinolin-6-yl]-1H-pyrazolo[3,4-d]pyrimidin-1-yl}-2-methylpropan-2-ol
4U41	2.2	MAP kinase kinase kinase kinase 4	3C8	6-[(3S)-3-(4-methyl-1H-pyrazol-3-yl)piperidin-1-yl]pyrido[3,2-d]pyrimidin-4-amine
4U43	2.18	MAP kinase kinase kinase kinase 4	3D8	N-(pyridin-3-yl)pyrrolo[2,1-f][1,2,4]triazin-4-amine
4U44	2.43	MAP kinase kinase kinase kinase 4	3D9	6-phenyl-N-(pyridin-4-yl)pyrrolo[2,1-f][1,2,4]triazin-4-amine
4U5J	2.26	Proto-oncogene tyrosine-protein kinase Src	RXT	(3R)-3-cyclopentyl-3-[4-(7H-pyrrolo[2,3-d]pyrimidin-4-yl)-1H-pyrazol-1-yl]propanenitrile
4U6R	2.5	Serine/threonine-protein kinase/endoribonuclease	3E4	N-{4-[(3-{2-[(trans-4-aminocyclohexyl)amino]pyrimidin-4-yl}pyridin-2-yl)oxy]-3-methylnaphthalen-1-yl}-2-chlorobenzenesulfonamide
4U79	2.23	MAP kinase 10	3EL	N-{4-[(3-{2-[(trans-4-aminocyclohexyl)amino]pyrimidin-4-yl}pyridin-2-yl)oxy]naphthalen-1-yl}benzenesulfonamide
4UAL	1.71	Serine/threonine-protein kinase MRCK beta	3FV	4-chloro-1-(piperidin-4-yl)-N-[3-(pyridin-2-yl)-1H-pyrazol-4-yl]-1H-pyrazole-3-carboxamide
4UB7	2.1	Casein kinase II	3G5	4-(6,8-dibromo-3-hydroxy-4-oxo-4H-chromen-2-yl)benzoic acid
4UJ1	1.768	cAMP-dependent protein kinase	NVX	7-[(3S,4R)-4-(3-chlorophenyl)carbonylpyrrolidin-3-yl]-3H-quinazolin-4-one
4UJ2	2.019	cAMP-dependent protein kinase	NVV	7-[(3S,4R)-4-(3-iodanylphenyl)carbonylpyrrolidin-3-yl]-3H-quinazolin-4-one
4UJ9	1.87	cAMP-dependent protein kinase	S3N	7-[(3S,4R)-4-[4-(trifluoromethyl)phenyl]carbonylpyrrolidin-3-yl]-3H-quinazolin-4-one
4UJA	1.93	cAMP-dependent protein kinase	4L7	7-{(3S,4R)-4-[(5-bromothiophen-2-yl)carbonyl]pyrrolidin-3-yl}quinazolin-4(3H)-one

4UJB	1.949	cAMP-dependent protein kinase	8BQ	7-[(3S,4R)-4-(3-fluorophenyl)carbonylpyrrolidin-3-yl]-3H-quinazolin-4-one
4UMP	2.3	Maternal embryonic leucine zipper kinase	5QM	3-(isoquinolin-7-yl)prop-2-yn-1-ol
4UMT	1.98	Maternal embryonic leucine zipper kinase	47W	1-(4-{[3-(isoquinolin-7-yl)prop-2-yn-1-yl]oxy}-2-methoxybenzyl)piperazinediium
4UMU	2.02	Maternal embryonic leucine zipper kinase	0V0	(2-ethoxy-4-{[3-(isoquinolin-7-yl)prop-2-yn-1-yl]oxy}phenyl)methanaminium
4USF	1.75	Serine/threonine-protein kinase STE20-like	6UI	4-[4-(6-methoxynaphthalen-2-yl)-1H-imidazol-5-yl]pyridine
4UWB	2.31	Fibroblast growth factor receptor 1	JVT	N-[4-(4-methylpiperazin-1-yl)phenyl]-1H-indazole-3-carboxamide
4UWC	1.96	Fibroblast growth factor receptor 1	4Y0	3,4-dimethoxy-N-(5-phenyl-1H-pyrazol-3-yl)benzamide
4UYN	1.9	Aurora kinase A	Y3M	ethyl (9S)-9-[5-(1H-benzimidazol-2-ylsulfanyl)furan-2-yl]-8-hydroxy-5,6,7,9-tetrahydro-2H-pyrrolo[3,4-b]quinoline-3-carboxylate
4UZH	2	Aurora kinase A	JVE	(4S)-4-(2-fluorophenyl)-2,4,6,7,8,9-hexahydro-5H-pyrazolo[3,4-b][1,7]naphthyridin-5-one
4W4V	2.01	c-Jun N-terminal kinase 3	3H8	3-(4-{[(2-chlorophenyl)carbamoyl]amino}-1H-pyrazol-1-yl)-N-(2-methylpyridin-4-yl)benzamide
4W4W	1.9	c-Jun N-terminal kinase 3	3HJ	N-(2-methylpyridin-4-yl)-3-{4-[(phenylcarbamoyl)amino]-1H-pyrazol-1-yl}benzamide
4W4Y	2.3	c-Jun N-terminal kinase 3	3HQ	3-(4-{[(4-methylphenyl)carbamoyl]amino}-1H-pyrazol-1-yl)-N-(2-methylpyridin-4-yl)benzamide
4W8D	1.77	Serine/threonine-protein kinase 24	3JA	5-(1-methyl-1H-pyrazol-4-yl)-4-(morpholin-4-yl)-7H-pyrrolo[2,3-d]pyrimidine
4W8E	1.79	Serine/threonine-protein kinase 24	3JB	3-{4-[(2R)-2-(5-methyl-1,2,4-oxadiazol-3-yl)morpholin-4-yl]-7H-pyrrolo[2,3-d]pyrimidin-5-yl}benzonitrile
4W9W	1.72	BMP-2-inducible protein kinase	YDJ	5-(3-fluorophenyl)-N-[(3S)-3-piperidyl]-3-ureido-thiophene-2-carboxamide
4W9X	2.14	BMP-2-inducible protein kinase	3JW	Baricitinib
4WAF	2.39	Phosphatidylinositol 4,5-bisphosphate 3-kinase	3K6	N,N-dimethyl-4-[(6R)-6-methyl-5-(1H-pyrrolo[2,3-b]pyridin-4-yl)-4,5,6,7-tetrahydropyrazolo[1,5-a]pyrazin-3-yl]benzenesulfonamide
4WG3	2.2	Calmodulin-domain protein kinase 1	UWA	3-(7-ethoxynaphthalen-2-yl)-1-(2-methylpropyl)-1H-pyrazolo[3,4-d]pyrimidin-4-amine
4WG4	2.3	Calmodulin-domain protein kinase 1	UWB	3-(7-ethoxynaphthalen-2-yl)-1-(piperidin-4-ylmethyl)-1H-pyrazolo[3,4-d]pyrimidin-4-amine
4WG5	2.3	Calmodulin-domain protein kinase 1	UW3	5-amino-3-[7-(cyclobutyloxy)quinolin-3-yl]-1-cyclohexyl-1H-pyrazole-4-carboxamide
4WHZ	1.79	MAP kinase 10	3NL	3-(4-{[(2-chlorophenyl)carbamoyl]amino}-1H-pyrazol-1-yl)-N-{1-[(3S)-pyrrolidin-3-yl]-1H-pyrazol-4-yl}benzamide
4WNK	2.42	G protein-coupled receptor kinase 5	453	(4S)-4-{4-fluoro-3-[(pyridin-2-ylmethyl)carbamoyl]phenyl}-N-(1H-indazol-5-yl)-6-methyl-2-oxo-1,2,3,4-tetrahydropyrimidine-5-carboxamide
4WNM	2.5	Tyrosine-protein kinase SYK	3RT	N~3~-(tetrahydro-2H-pyran-4-yl)-N~6~-[5-(tetrahydro-2H-pyran-4-ylmethyl)[1,2,4]triazolo[1,5-a]pyridin-2-yl]-1H-indazole-3,6-diamine
4WNO	1.56	Serine/threonine-protein kinase ULK1	3RF	N~2~-(4-aminophenyl)-N~4~-(5-cyclopropyl-1H-pyrazol-3-yl)quinazoline-2,4-diamine
4WNP	1.88	Serine/threonine-protein kinase ULK1	3RJ	N~2~-(1H-benzimidazol-6-yl)-N~4~-(5-cyclobutyl-1H-pyrazol-3-yl)quinazoline-2,4-diamine
4WOV	1.8	Non-receptor tyrosine-protein kinase TYK2	3SM	2-methoxy-N-({6-[1-methyl-4-(methylamino)-1,6-dihydroimidazo[4,5-d]pyrrolo[2,3-b]pyridin-7-yl]pyridin-2-yl}methyl)acetamide
4WRS	2.2	Serine/threonine-protein kinase pim-1	3U1	3-{6-[(4R)-6-azaspiro[2.5]oct-4-yloxy]pyrazin-2-yl}-5-(2,6-difluorophenyl)-1H-indazole
4WSY	2.3	Serine/threonine-protein kinase pim-1	3U5	5-[3-(quinolin-3-yl)-2H-indazol-5-yl]-1,3-thiazol-2-amine
4WT6	2.3	Serine/threonine-protein kinase pim-1	3U6	6-[5-(5-amino-1,3,4-thiadiazol-2-yl)-1H-indol-3-yl]-N-cyclopentylpyridin-2-amine
4WUA	2	SRSF protein kinase 1	3UL	N-[2-(1-piperidinyl)-5-(trifluoromethyl)phenyl]-4-pyridinecarboxamide
4WUN	1.65	Fibroblast growth factor receptor 1	66T	N-{3-[2-(3,5-dimethoxyphenyl)ethyl]-1H-pyrazol-5-yl}-4-[(3R,5S)-3,5-dimethylpiperazin-1-yl]benzamide
4WWO	2.3	Phosphatidylinositol 4,5-bisphosphate 3-kinase	3VD	N-{(1S)-1-[8-chloro-2-(3-fluorophenyl)quinolin-3-yl]ethyl}-9H-purin-6-amine
4WWP	2.4	Phosphatidylinositol 4,5-bisphosphate 3-kinase	3VE	N-{(1S)-1-[8-chloro-2-(2-methylpyridin-3-yl)quinolin-3-yl]ethyl}-9H-purin-6-amine
4X0M	1.68	TGF-beta receptor type-1	3WA	4-aminopyrido[2,3-d]pyrimidin-5(8H)-one
4X21	1.95	MAP kinase 10	3WH	N-ethyl-4-{[4-(1H-indol-3-yl)-5-iodopyrimidin-2-yl]amino}piperidine-1-carboxamide
4X2F	1.49	TGF-beta receptor type-1	3WJ	4-amino-8-(4-aminophenyl)pyrido[2,3-d]pyrimidin-5(8H)-one
4X2G	1.51	TGF-beta receptor type-1	3WK	4-[(4-aminophenyl)amino]pyrido[2,3-d]pyrimidin-5(6H)-one
4X2J	1.69	TGF-beta receptor type-1	3WN	4-[(3-aminophenyl)amino]pyrido[2,3-d]pyrimidin-5(8H)-one
4X2K	1.69	TGF-beta receptor type-1	3WO	4-[(3-aminophenyl)amino]pyrido[2,3-d]pyrimidin-5(6H)-one
4X3J	2.5	Angiopoietin-1 receptor	3WR	1-[4-(4-amino-5-oxopyrido[2,3-d]pyrimidin-8(5H)-yl)phenyl]-3-[2-fluoro-5-(trifluoromethyl)phenyl]urea
4X7H	2	Eukaryotic translation initiation factor 2-alpha	3Z2	N-{5-[(6,7-dimethoxyquinolin-4-yl)oxy]pyridin-2-yl}-1-methyl-3-oxo-2-phenyl-5-(pyridin-4-yl)-2,3-dihydro-1H-pyrazole-4-carboxamide
4X7J	2.3	Eukaryotic translation initiation factor 2-alpha	3Z1	2-amino-N-[4-methoxy-3-(trifluoromethyl)phenyl]-4-methyl-3-[2-(methylamino)quinazolin-6-yl]benzamide
4X7K	1.8	Eukaryotic translation initiation factor 2-alpha	3Z3	4-{2-amino-3-[5-fluoro-2-(methylamino)quinazolin-6-yl]-4-methylbenzoyl}-1-methyl-2,5-diphenyl-1,2-dihydro-3H-pyrazol-3-one
4X7L	1.9	Eukaryotic translation initiation factor 2-alpha	3Z4	4-{2-amino-4-methyl-3-[2-(methylamino)-1,3-benzothiazol-6-yl]benzoyl}-1-methyl-2,5-diphenyl-1,2-dihydro-3H-pyrazol-3-one
4X7N	2.35	Eukaryotic translation initiation factor 2-alpha	3Z5	4-[2-amino-4-methyl-3-(2-methylquinolin-6-yl)benzoyl]-1-methyl-2,5-diphenyl-1,2-dihydro-3H-pyrazol-3-one
4X7Q	2.33	Serine/threonine-protein kinase pim-2	3YR	2-(2,6-difluorophenyl)-N-{4-[(3S)-pyrrolidin-3-yloxy]pyridin-3-yl}-1,3-thiazole-4-carboxamide
4XCU	1.71	Fibroblast growth factor receptor 4	40M	N-(2-{[6-(2,6-dichloro-3,5-dimethoxyphenyl)quinazolin-2-yl]amino}-3-methylphenyl)propanamide
4XE0	2.434	Phosphatidylinositol 4,5-bisphosphate 3-kinase	40L	5-fluoro-3-phenyl-2-[(1S)-1-(7H-purin-6-ylamino)propyl]quinazolin-4(3H)-one
4XG3	2.3	Tyrosine-protein kinase SYK	X3G	4-{[5-fluoro-4-(3-{[(3R)-3-hydroxypyrrolidin-1-yl]methyl}-4-methyl-1H-pyrrol-1-yl)pyrimidin-2-yl]amino}-2,6-dimethylphenyl methanesulfonate
4XG4	2.3	Tyrosine-protein kinase SYK	X4G	(3R)-1-{[1-(5-fluoro-2-{[4-(2-hydroxyethoxy)-3,5-dimethylphenyl]amino}pyrimidin-4-yl)-4-methyl-1H-pyrrol-3-yl]methyl}pyrrolidin-3-ol
4XG6	2.4	Tyrosine-protein kinase SYK	X6G	1-[(1-{2-[(3,5-dimethylphenyl)amino]pyrimidin-4-yl}-3-methyl-1H-pyrazol-4-yl)methyl]azetidin-3-ol
4XG7	1.76	Tyrosine-protein kinase SYK	X7G	1-[(3-methyl-1-{2-[(1-methyl-1H-indazol-5-yl)amino]pyrimidin-4-yl}-1H-pyrazol-4-yl)methyl]azetidin-3-ol
4XG8	2.4	Tyrosine-protein kinase SYK	X8G	1-[(1-{2-[(3-chloro-1-methyl-1H-indazol-5-yl)amino]pyrimidin-4-yl}-3-methyl-1H-pyrazol-4-yl)methyl]azetidin-3-ol

4XH6	2.04	Serine/threonine-protein kinase pim-1	HUL	5,7-dihydroxy-2-(4-hydroxyphenyl)-6-methoxy-4H-chromen-4-one
4XMO	1.75	Hepatocyte growth factor receptor	46G	6-{(1R)-1-fluoro-1-[8-fluoro-6-(3-methyl-1,2-oxazol-5-yl)[1,2,4]triazolo[4,3-a]pyridin-3-yl]ethyl}-3-methoxyquinoline
4XP0	1.46	MAP kinase 1	42A	1H-pyrrolo[2,3-b]pyridine-3-carbonitrile
4XP2	1.748	MAP kinase 1	TT4	1-phenyl-1H-1,2,4-triazole-3,5-diamine
4XP3	1.782	MAP kinase 1	DX4	2-amino-1,9-dihydro-6H-purine-6-thione
4XRJ	1.69	MAP kinase 1	620	N~1~-[3-(benzyloxy)benzyl]-1H-tetrazole-1,5-diamine
4XV1	2.47	Serine/threonine-protein kinase B-raf	904	N'-(3-{[5-(2-cyclopropylpyrimidin-5-yl)-1H-pyrrolo[2,3-b]pyridin-3-yl]carbonyl}-2,4-difluorophenyl)-N-ethyl-N-methylsulfuric diamide
4XV2	2.5	Serine/threonine-protein kinase B-raf	P06	Dabrafenib
4XV9	2	Serine/threonine-protein kinase B-raf	1OO	N-{3-[(5-chloro-1H-pyrrolo[2,3-b]pyridin-3-yl)carbonyl]-2,4-difluorophenyl}-4-(trifluoromethyl)benzenesulfonamide
4XYF	1.85	Hepatocyte growth factor receptor	44X	6-{(1S)-1-[8-fluoro-6-(3-methyl-1,2-oxazol-5-yl)[1,2,4]triazolo[4,3-a]pyridin-3-yl]ethyl}-3-(2-methoxyethoxy)quinoline
4Y46	2.04	MAP kinase 10	4F2	1-{trans-4-[(8-cyclopentyl-7-oxo-7,8-dihydropyrido[2,3-d]pyrimidin-2-yl)amino]cyclohexyl}-3-propan-2-ylurea
4Y5H	2.055	MAP kinase 10	519	1-(trans-4-{[7-oxo-8-(propan-2-yl)-7,8-dihydropyrido[2,3-d]pyrimidin-2-yl]amino}cyclohexyl)-3-propan-2-ylurea
4Y73	2.14	Interleukin-1 receptor-associated kinase 4	XPY	5-{[(1R,2S)-2-aminocyclohexyl]amino}-N-[1-methyl-3-(trifluoromethyl)-1H-pyrazol-4-yl]pyrazolo[1,5-a]pyrimidine-3-carboxamide
4Y85	2.33	MAP kinase kinase kinase 8	499	5-[5-(1H-indol-3-yl)-1H-pyrrolo[2,3-b]pyridin-3-yl]-1,3,4-oxadiazol-2-amine
4Y8D	2.1	Cyclin-G-associated kinase	49J	2-methoxy-4-[3-(morpholin-4-yl)[1,2]thiazolo[4,3-b]pyridin-6-yl]aniline
4YBK	2.5	Proto-oncogene tyrosine-protein kinase Src	4B7	2-({6-[4-(2-hydroxyethyl)piperazin-1-yl]-2-methylpyrimidin-4-yl}amino)-N-(4-phenoxyphenyl)-1,3-thiazole-5-carboxamide
4YHF	2.2	Tyrosine-protein kinase BTK	4C9	(2S)-2-({(3R)-3-[4-amino-3-(4-phenoxyphenyl)-1H-pyrazolo[3,4-d]pyrimidin-1-yl]piperidin-1-yl}carbonyl)-4,4-dimethylpentanenitrile
4YJO	1.6	Tyrosine-protein kinase SYK	4DF	5-chloro-N~2~-(1,1-dioxido-2,3-dihydro-1,2-benzothiazol-6-yl)-N~4~-ethyl-N~4~-(1H-indazol-4-yl)pyrimidine-2,4-diamine
4YJP	1.83	Tyrosine-protein kinase SYK	4DL	2-[{2-[(1,1-dioxido-2,3-dihydro-1,2-benzothiazol-6-yl)amino]pyrimidin-4-yl}(1H-indazol-4-yl)amino]ethanol
4YJQ	1.34	Tyrosine-protein kinase SYK	4DK	3-[1H-indazol-4-yl(2-{[3-(4-methyl-1,3-oxazol-5-yl)phenyl]amino}pyrimidin-4-yl)amino]propan-1-ol
4YJR	1.32	Tyrosine-protein kinase SYK	4DJ	3-(1H-indazol-4-yl{2-[(1-methyl-1H-indazol-5-yl)amino]pyrimidin-4-yl}amino)propan-1-ol
4YJS	2.22	Tyrosine-protein kinase SYK	4DN	3-[{2-[(1,1-dioxido-2,3-dihydro-1,2-benzothiazol-6-yl)amino]pyrimidin-4-yl}(1H-indazol-4-yl)amino]propan-1-ol
4YJT	1.52	Tyrosine-protein kinase SYK	4DQ	N~2~-(1,1-dioxido-2,3-dihydro-1,2-benzothiazol-6-yl)-N~4~-ethyl-5-fluoro-N~4~-(1H-indazol-4-yl)pyrimidine-2,4-diamine
4YJU	1.67	Tyrosine-protein kinase SYK	4DO	N~2~-(1,1-dioxido-2,3-dihydro-1,2-benzothiazol-6-yl)-5-fluoro-N~4~-(1H-indazol-4-yl)-N~4~-methylpyrimidine-2,4-diamine
4YJV	1.652	Tyrosine-protein kinase SYK	4DT	3-[6-({4-[ethyl(1H-indazol-4-yl)amino]-5-fluoropyrimidin-2-yl}amino)-2,4-dihydro-1H-indazol-1-yl]propan-1-ol
4YLL	1.4	Dual specificity tyrosine-phosphorylation-	4E3	10-bromo-2-iodo-11H-indolo[3,2-c]quinoline-6-carboxylic acid
4YMJ	2	NT-3 growth factor receptor	4EJ	4-[6-(benzylamino)imidazo[1,2-b]pyridazin-3-yl]benzonitrile
4YNE	2.0229	High affinity nerve growth factor receptor	4EK	6-[(2R)-2-(3-fluorophenyl)pyrrolidin-1-yl]-3-(pyridin-2-yl)imidazo[1,2-b]pyridazine
4YO4	1.6	Death-associated protein kinase 1	4FT	phthalazine
4YO6	2.32	Interleukin-1 receptor-associated kinase 4	4GD	N-(3-methyl-1-phenyl-1H-pyrazol-5-yl)pyrazolo[1,5-a]pyrimidine-3-carboxamide
4YPD	1.4	Death-associated protein kinase 1	DKG	4-methylpyridazine
4YPS	2.1012	High affinity nerve growth factor receptor	4F6	4-{6-[(3R)-3-(3-fluorophenyl)morpholin-4-yl]imidazo[1,2-b]pyridazin-3-yl}benzonitrile
4YTC	2.16	Tyrosine-protein kinase JAK2	4HW	N~3~-phenyl-1-[6-(phenylamino)pyrimidin-4-yl]-1H-1,2,4-triazole-3,5-diamine
4YTF	1.78	Tyrosine-protein kinase JAK2	4HZ	N~2~-[2-(5-chloro-1H-pyrrolo[2,3-b]pyridin-3-yl)-5-fluoropyrimidin-4-yl]-N-(2,2,2-trifluoroethyl)-L-alaninamide
4YTH	2.04	Tyrosine-protein kinase JAK2	467	N~2~-[2-(5-chloro-1H-pyrrolo[2,3-b]pyridin-3-yl)-5-fluoropyrimidin-4-yl]-2-methyl-N-(2,2,2-trifluoroethyl)-D-alaninamide
4YXR	2	cAMP-dependent protein kinase	0RW	3-methyl-2H-indazole
4YXS	2.11	cAMP-dependent protein kinase	EMU	N-BENZYL-9H-PURIN-6-AMINE
4YZ9	2.463	Serine/threonine-protein kinase/endoribonuclease	4K7	(5R)-2-(3,4-dichlorobenzyl)-N-(4-methylbenzyl)-2,7-diazaspiro[4.5]decane-7-carboxamide
4YZN	1.55	Probable serine/threonine-protein kinase roco4	4K5	(4-{[4-(cyclopropylamino)-5-(trifluoromethyl)pyrimidin-2-yl]amino}-2-fluoro-5-methoxyphenyl)(morpholin-4-yl)methanone
4Z3V	1.6	Tyrosine-protein kinase BTK	4L6	4-amino-8-(5-methyl-1H-indazol-6-yl)cinnoline-3-carboxamide
4Z55	1.55	ALK tyrosine kinase receptor	4LO	N~6~-[5-methyl-4-(1-methylpiperidin-4-yl)-2-(propan-2-yloxy)phenyl]-N~4~-[2-(propan-2-ylsulfonyl)phenyl]-2H-pyrazolo[3,4-d]pyrimidine-4,6-diamine
4Z9L	2.1	MAP kinase 10	880	CYCLOHEXYL-{4-[5-(3,4-DICHLOROPHENYL)-2-PIPERIDIN-4-YL-3-PROPYL-3H-IMIDAZOL-4-YL]-PYRIMIDIN-2-YL}AMINE
4ZEG	2.33	Dual specificity protein kinase TTK	052	N-cyclopropyl-2-methyl-4-(7-{[2-(morpholin-4-yl)ethyl]amino}-5-phenoxypyrazolo[1,5-a]pyrimidin-3-yl)benzamide
4ZJI	1.99	Serine/threonine-protein kinase PAK 1	4OQ	2-chloro-5-ethyl-8-fluoro-11-(4-methylpiperazin-1-yl)-dibenzodiazepine
4ZJJ	2.2	Serine/threonine-protein kinase PAK 1	4OR	(S)-N-(tert-butyl)-3-((2-chloro-5-ethyl-8-fluoro-dibenzodiazepin-11-yl)amino)pyrrolidine-1-carboxamide
4ZLO	2.5	Serine/threonine-protein kinase PAK 1	4PV	2,8-difluoro-11-(4-methylpiperazin-1-yl)-5H-dibenzo[b,e][1,4]diazepine
4ZLY	1.65	Tyrosine-protein kinase BTK	4RU	4-aminocinnoline-3-carboxamide
4ZLZ	2	Tyrosine-protein kinase BTK	4RV	4-amino-8-(4-methylpyridin-3-yl)cinnoline-3-carboxamide
4ZP5	2.29	MAP kinase kinase kinase kinase 4	4QG	4-[5-(4-chlorophenyl)-1,3-oxazol-2-yl]benzamide
4ZSA	2	Fibroblast growth factor receptor 1	4UT	4-(4-ethylpiperazin-1-yl)-N-[6-(3-methoxyphenyl)-2H-indazol-3-yl]benzamide
4ZSG	1.79	MAP kinase 7	4QX	3-amino-5-[(4-chlorophenyl)amino]-N-(propan-2-yl)-1H-1,2,4-triazole-1-carboxamide
4ZSJ	2.48	MAP kinase 7	4R0	3-amino-5-[(4-chloro-3-methylphenyl)amino]-N-(propan-2-yl)-1H-1,2,4-triazole-1-carboxamide
4ZSL	2.25	MAP kinase 7	4QZ	3-amino-5-[(4-chlorophenyl)amino]-N-[(1S)-1-phenylethyl]-1H-1,2,4-triazole-1-carboxamide

4ZTH	2.15	MAP kinase 14	VVT	6-chloro-3-phenyl-4-(pyridin-4-yl)pyridazine
4ZTL	2.39	Interleukin-1 receptor-associated kinase 4	4S1	(1R,2S,3R,5R)-3-{[5-(1,3-benzothiazol-2-yl)-2-(propylamino)pyrimidin-4-yl]amino}-5-(hydroxymethyl)cyclopentane-1,2-diol
4ZTN	2.23	Interleukin-1 receptor-associated kinase 4	4S3	5-(1,3-benzothiazol-2-yl)-2-(morpholin-4-yl)-6-[(3R)-piperidin-3-ylamino]pyrimidin-4(3H)-one
4ZXT	2	MAP kinase 1	CAQ	CATECHOL
4ZY5	2.35	Serine/threonine-protein kinase PAK 1	4T5	N~2~-[(trans-4-aminocyclohexyl)methyl]-N~4~-(3-cyclopropyl-1H-pyrazol-5-yl)pyrimidine-2,4-diamine
4ZY6	2.15	Serine/threonine-protein kinase PAK 1	4T6	N~2~-[(7-chloro-1H-benzimidazol-6-yl)methyl]-N~4~-(5-cyclopropyl-1H-pyrazol-3-yl)pyrimidine-2,4-diamine
4ZZM	1.89	MAP kinase 1	CQ6	7-ethylsulfonyl-N-(oxan-4-yl)-6,8-dihydro-5H-pyrido[3,4-d]pyrimidin-2-amine
4ZZN	1.33	MAP kinase 1	CQ8	2-[[5-chloranyl-2-(oxan-4-ylamino)pyridin-4-yl]amino]-N-methyl-benzamide
4ZZO	1.63	MAP kinase 1	CQ3	N-[2-[[5-chloranyl-2-(oxan-4-ylamino)pyrimidin-4-yl]amino]phenyl]propanamide
5A14	2	Cyclin-dependent kinase 2	LQ5	1-[4-(2-azanylpyrimidin-4-yl)oxyphenyl]-3-[4-[(4-methylpiperazin-1-yl)methyl]-3-(trifluoromethyl)phenyl]urea
5A3X	2.26	Dual specificity tyrosine-phosphorylation-	QIV	N-(5-oxidanyl-1,3-benzothiazol-2-yl)ethanamide
5A4C	2.09	Fibroblast growth factor receptor 1	XOJ	1-tert-butyl-3-[2-[3-(diethylamino)propylamino]-6-(3,5-dimethoxyphenyl)pyrido[2,3-d]pyrimidin-7-yl]urea
5A4Q	2.37	Dual specificity tyrosine-phosphorylation-	Y3L	N-(5-CHLORANYL-1,3-BENZOTHIAZOL-2-YL)ETHANAMIDE
5A4T	2.15	Dual specificity tyrosine-phosphorylation-	AJG	N-(6-CYANO-3H-1,3-BENZOTHIAZOL-2-YLIDENE)ETHANAMIDE
5A6N	1.7	Death-associated protein kinase 3	U7E	5-(3-SULFAMOYLPHENYL)-1H-1,2,3,4-TETRAZOL-1-IDE
5A9U	1.6	ALK tyrosine kinase receptor	5P8	(10R)-7-amino-12-fluoro-2,10,16-trimethyl-15-oxo-10,15,16,17-tetrahydro-2H-8,4-(metheno)pyrazolo[4,3-h][2,5,11]benzoxadiazacyclotetradecine-3-carbonitrile
5AE8	2.42	Phosphatidylinositol 4,5-bisphosphate 3-kinase	VVX	6-(1H-Indol-4-yl)-4-(5-{[4-(1-methylethyl)-1-piperazinyl]methyl}-1,3-oxazol-2-yl)-1H-indazole
5AE9	2.44	Phosphatidylinositol 4,5-bisphosphate 3-kinase	OKO	N-[5-[4-(5-{[(2R,6S)-2,6-DIMETHYL-4-MORPHOLINYL]METHYL}-1,3-OXAZOL-2-YL)-1H-INDAZOL-6-YL]-2-(METHYLOXY)-3-PYRIDINYL]METHANESULFONAMIDE
5AEP	1.95	Tyrosine-protein kinase JAK2	QUP	1-(5-chloro-2-methylphenyl)-4-(pyrrolo[2,1-f][1,2,4]triazin-4-yl)-1H-pyrrole-2-carboxamide
5AM7	1.957	Fibroblast growth factor receptor 1	38O	4-amino-5-fluoro-3-[5-(4-methylpiperazin-1-yl)-1H-benzimidazol-2-yl]quinolin-2(1H)-one
5AND	2.3	Cyclin-dependent kinase 2	5JE	2-IMIDAZOL-1-YL-1H-BENZIMIDAZOLE
5ANE	1.7	Cyclin-dependent kinase 2	SZL	6-METHOXY-9H-PURINE
5ANG	1.9	Cyclin-dependent kinase 2	WY3	7-HYDROXY-4-(MORPHOLINOMETHYL)CHROMEN-2-ONE
5ANI	1.9	Cyclin-dependent kinase 2	ES4	6-chloro-9H-purine
5ANJ	1.6	Cyclin-dependent kinase 2	ZXC	N-(9H-purin-6-yl)thiophene-2-carboxamide
5ANK	1.9	Cyclin-dependent kinase 2	RJI	2,4,6-TRIOXO-1-PHENYL-HEXAHYDROPYRIMIDINE-5-CARBOXAMIDE
5AP0	2.15	Dual specificity protein kinase TTK	AU5	9-CYCLOPENTYL-2-[[2-METHOXY-4-[(1-METHYLPIPERIDIN-4-YL)OXY]-PHENYL]AMINO]-7-METHYL-7,9-DIHYDRO-8H-PURIN-8-ONE
5AP1	2.05	Dual specificity protein kinase TTK	O38	6-{[3-(cyanomethoxy)-4-(1-methyl-1H-pyrazol-4-yl)phenyl]amino}-2-(cyclohexylamino)pyridine-3-carbonitrile
5AP6	2.1	Dual specificity protein kinase TTK	PWU	ISOPROPYL 6-((4-(1,2-DIMETHYL-1H-IMIDAZOL-5-YL)PHENYL)AMINO)-2-(1-METHYL-1H-PYRAZOL-4-YL)-1H-PYRROLO[3,2-C]PYRIDINE-1-CARBOXYLATE
5AP7	2.45	Monopolar Spindle Kinase 1	SVE	N-(2,6-DIETHYLPHENYL)-1-METHYL-8-({4-[(1-METHYLPIPERIDIN-4-YL)CARBAMOYL]-2-(TRIFLUOROMETHOXY)PHENYL}AMINO)-4,5-DIHYDRO-1H-PYRAZOLO[4,3-H]QUINAZOLINE-3-CARBOXAMIDE
5AUU	1.7	Death-associated protein kinase 1	LU2	2-(3,4-dihydroxyphenyl)-5,7-dihydroxy-4H-chromen-4-one
5AUX	1.5	Death-associated protein kinase 1	KMP	3,5,7-TRIHYDROXY-2-(4-HYDROXYPHENYL)-4H-CHROMEN-4-ONE
5AUY	2	Death-associated protein kinase 1	MRI	2-[2,4-bis(oxidanyl)phenyl]-3,5,7-tris(oxidanyl)chromen-4-one
5AV0	1.85	Death-associated protein kinase 1	47X	3-(3,4-dihydroxyphenyl)-7-hydroxy-4H-chromen-4-one
5AV4	1.4	Death-associated protein kinase 1	GEN	GENISTEIN
5AX9	2.4	TRAF2 and NCK-interacting protein kinase	4KT	4-methoxy-3-[2-[(3-methoxy-4-morpholin-4-yl-phenyl)amino]pyridin-4-yl]benzenecarbonitrile
5B0X	2.3	Casein kinase II	HCK	4-[2-[(4-methoxyphenyl)carbonylamino]-1,3-thiazol-5-yl]benzoic acid
5B7V	2.15	Fibroblast growth factor receptor 1	LWJ	[5-amino-1-(2-methyl-1H-benzimidazol-6-yl)-1H-pyrazol-4-yl](1H-indol-2-yl)methanone
5BPY	2.31	Tyrosine-protein kinase BTK	4UQ	6-{(3R)-3-[(4-tert-butylbenzoyl)amino]piperidin-1-yl}-2-{[4-(morpholin-4-ylcarbonyl)phenyl]amino}pyridine-3-carboxamide
5BQ0	1.57	Tyrosine-protein kinase BTK	4US	4-(2-chlorophenyl)-7-[(4-methylpiperazin-1-yl)carbonyl]-9H-carbazole-1-carboxamide
5BUE	2.4	MAP kinase 1	4V8	1-benzyl-4-[3-(pyridin-4-yl)-2,4,6,7-tetrahydro-5H-pyrazolo[4,3-c]pyridin-5-yl]pyridin-2(1H)-one
5BUI	2.12	MAP kinase 1	4V9	3-(4-fluorophenyl)-5-(pyridin-2-yl)-4,5,6,7-tetrahydro-2H-pyrazolo[4,3-c]pyridine
5BUJ	1.85	MAP kinase 1	4VB	4-[3-(pyridin-4-yl)-2,4,6,7-tetrahydro-5H-pyrazolo[4,3-c]pyridin-5-yl]pyridin-2(1H)-one
5BVD	1.9	MAP kinase 1	4VF	2-[(1S)-1-(3-chlorophenyl)-2-hydroxyethyl]-7-[2-(tetrahydro-2H-pyran-4-ylamino)pyrimidin-4-yl]-3,4-dihydropyrrolo[1,2-a]pyrazin-1(2H)-one
5BVE	2	MAP kinase 1	4VG	2-[(1S)-1-(3-chlorophenyl)-2-hydroxyethyl]-8-[2-(tetrahydro-2H-pyran-4-ylamino)pyrimidin-4-yl]-2,3,4,5-tetrahydro-1H-pyrrolo[1,2-a][1,4]diazepin-1-one
5BVF	1.9	MAP kinase 1	4VJ	2-[(1S)-1-(3-chlorophenyl)-2-hydroxyethyl]-8-(2-{[(1S,3R)-3-hydroxycyclopentyl]amino}pyrimidin-4-yl)-2,3,4,5-tetrahydro-1H-pyrrolo[1,2-a][1,4]diazepin-1-one
5BVK	2.29	Epithelial discoidin domain-containing receptor 1	4VC	1-(2-chlorophenyl)-3-(pyridin-3-ylmethyl)urea
5BVN	2.21	Epithelial discoidin domain-containing receptor 1	4VD	N-[5-({[(3-fluorophenyl)carbamoyl]amino}methyl)-2-methylphenyl]imidazo[1,2-a]pyridine-3-carboxamide
5BVO	1.98	Epithelial discoidin domain-containing receptor 1	4VE	N-(5-{(1S)-1-[(5-fluoro-1,3-benzoxazol-2-yl)amino]ethyl}-2-methylphenyl)imidazo[1,2-a]pyridine-3-carboxamide
5BX6	1.89	cAMP-dependent protein kinase	495	4-chlorophthalazin-1(2H)-one
5BX7	1.89	cAMP-dependent protein kinase	4W1	1-benzothiophen-3-ylmethanol
5BYZ	1.65	MAP kinase 7	4WE	4-({5-fluoro-4-[2-methyl-1-(propan-2-yl)-1H-imidazol-5-yl]pyrimidin-2-yl}amino)-N-[2-(piperidin-1-yl)ethyl]benzamide
5C26	1.95	Tyrosine-protein kinase SYK	50H	3-{8-[(3,4-dimethoxyphenyl)amino]imidazo[1,2-a]pyrazin-6-yl}benzamide

5C27	2.15	Tyrosine-protein kinase SYK	50J	3-{8-[(3,4-dimethoxyphenyl)amino]imidazo[1,2-a]pyrazin-6-yl}-N-[4-(methylcarbamoyl)phenyl]benzamide
5C8N	2.401	Epidermal growth factor receptor	4YX	N-{2-[4-(2-aminoethyl)-4-methoxypiperidin-1-yl]pyrimidin-4-yl}-2-methyl-1-(propan-2-yl)-1H-imidazo[4,5-c]pyridin-6-amine
5CAP	2.4	Epidermal growth factor receptor	4ZH	2-methyl-N-[2-(2-methyl-2-methylsulfonyl-propoxy)pyrimidin-4-yl]-1-propan-2-yl-imidazo[4,5-c]pyridin-6-amine
5CAQ	2.5	Epidermal growth factor receptor	4ZJ	N-[2-[(3R,4S)-3-fluoranyl-4-methoxy-piperidin-1-yl]pyrimidin-4-yl]-2-methyl-1-propan-2-yl-imidazo[4,5-c]pyridin-6-amine
5CAS	2.1	Epidermal growth factor receptor	4ZQ	(1R)-1-{6-({2-[(3R,4S)-3-fluoro-4-methoxypiperidin-1-yl]pyrimidin-4-yl}amino)-1-[(2S)-1,1,1-trifluoropropan-2-yl]-1H-imidazo[4,5-c]pyridin-2-yl}ethanol
5CAU	2.25	Epidermal growth factor receptor	4ZR	(1R)-1-{6-({2-[(3S,4R)-3-fluoro-4-methoxypiperidin-1-yl]pyrimidin-4-yl}amino)-1-[(2S)-1,1,1-trifluoropropan-2-yl]-1H-imidazo[4,5-c]pyridin-2-yl}ethanol
5CEI	2.24	Cyclin-dependent kinase 8	50R	4-(4-iodophenoxy)-N-methylthieno[2,3-c]pyridine-2-carboxamide
5CEO	2.28	MAP kinase kinase kinase 12	50D	2-[[6-[3,3-bis(fluoranyl)pyrrolidin-1-yl]-4-[1-(oxetan-3-yl)piperidin-4-yl]pyridin-2-yl]amino]pyridine-4-carbonitrile
5CEP	1.99	MAP kinase kinase kinase 12	50E	N-(5-piperidin-4-yl-1-propan-2-yl-pyrazol-3-yl)-4-(trifluoromethyl)pyridin-2-amine
5CEQ	1.911	MAP kinase kinase kinase 12	50F	2-[[1-cyclopentyl-5-[1-(oxetan-3-yl)piperidin-4-yl]pyrazol-3-yl]amino]pyridine-4-carbonitrile
5CF4	2.38	Tyrosine-protein kinase JAK2	50Y	N,N-dicyclopropyl-6-ethyl-4-[(3-methoxypropyl)amino]-1-methyl-1,6-dihydroimidazo[4,5-d]pyrrolo[2,3-b]pyridine-7-carboxamide
5CF5	2.45	Tyrosine-protein kinase JAK2	50W	N,N-dicyclopropyl-4-[(4,5-dimethyl-1,3-thiazol-2-yl)amino]-6-ethyl-1-methyl-1,6-dihydroimidazo[4,5-d]pyrrolo[2,3-b]pyridine-7-carboxamide
5CF6	2.5	Tyrosine-protein kinase JAK2	50O	N,N-dicyclopropyl-6-[(2S)-2,3-dihydroxypropyl]-1-methyl-4-(methylamino)-1,6-dihydroimidazo[4,5-d]pyrrolo[2,3-b]pyridine-7-carboxamide
5CF8	1.8	Tyrosine-protein kinase JAK2	50V	N,N-dicyclopropyl-4-[(1,5-dimethyl-1H-pyrazol-3-yl)amino]-6-ethyl-1-methyl-1,6-dihydroimidazo[4,5-d]pyrrolo[2,3-b]pyridine-7-carboxamide
5CI7	1.74	Serine/threonine-protein kinase ULK1	51W	N-[3-({4-[(3-aminopropyl)amino]-5-iodopyrimidin-2-yl}amino)phenyl]pyrrolidine-1-carboxamide
5CLP	1.684	Casein kinase II	42J	2-(3,4-dichlorophenyl)ethanamine
5CQU	2.35	Casein kinase II	JRJ	4-[4-[2-[4,5,6,7-tetrakis(bromanyl)benzotriazol-2-yl]ethyl]-1,2,3-triazol-1-yl]butan-1-amine
5CSP	1.5	Casein kinase II	54G	2-hydroxy-5-methylbenzoic acid
5CSV	1.375	Casein kinase II	GAB	3-AMINOBENZOIC ACID
5CT0	2.008	Casein kinase II	54P	3-{[(2-chlorobiphenyl-4-yl)methyl]amino}propan-1-ol
5CTP	2.033	Casein kinase II	54R	N-(3-{[(2-chlorobiphenyl-4-yl)methyl]amino}propyl)acetamide
5CU2	1.705	Casein kinase II	551	methyl 3-[(3-{[(2-chlorobiphenyl-4-yl)methyl]amino}propyl)amino]-3-oxopropanoate
5CU4	1.56	Casein kinase II	54S	N-[(2-chlorobiphenyl-4-yl)methyl]-beta-alanyl-N-(3-carboxyphenyl)-beta-alaninamide
5CXH	1.9	Tyrosine-protein kinase SYK	55M	(4R)-4-[(1R)-1-{[6-(3,4-dimethoxyphenyl)[1,3]thiazolo[5,4-c]pyridin-4-yl]oxy}ethyl]pyrrolidin-2-one
5CXZ	1.7	Tyrosine-protein kinase SYK	55U	N-{7-[4-(dimethylamino)phenyl]-1,6-naphthyridin-5-yl}propane-1,3-diamine
5CY3	1.76	Tyrosine-protein kinase SYK	55Y	(5R)-5-[(1R)-1-{[6-(1-methyl-1H-pyrazol-4-yl)-2,1-benzothiazol-4-yl]oxy}ethyl]-1,3-oxazolidin-2-one
5CYI	2	Cyclin-dependent kinase 2	55S	6-(cyclohexylmethoxy)-N-[4-(ethylsulfonyl)phenyl]-9H-purin-2-amine
5D11	2.3	Proto-oncogene tyrosine-protein kinase Src	56G	N-[3-({4-(4-methylpiperazin-1-yl)-6-[(5-methyl-1H-pyrazol-3-yl)amino]pyrimidin-2-yl}oxy)phenyl]prop-2-enamide
5D1J	1.8	Cyclin-dependent kinase 2	56H	N-(5-{[(5-tert-butyl-1,3-oxazol-2-yl)methyl]sulfanyl}-1,3-thiazol-2-yl)piperidine-4-carboxamide
5D9L	2.15	Ribosomal protein S6 kinase alpha-3	583	4,4'-(1H-pyrazole-3,4-diyl)diphenol
5DA3	1.7	Protein-tyrosine kinase 6	58V	(2-chloro-4-{[6-cyclopropyl-3-(1H-pyrazol-4-yl)imidazo[1,2-a]pyrazin-8-yl]amino}phenyl)(morpholin-4-yl)methanone
5DEW	1.9	Serine/threonine-protein kinase PAK 1	59N	8-[(trans-4-aminocyclohexyl)methyl]-6-[2-chloro-4-(6-methylpyrazin-2-yl)phenyl]-2-(ethylamino)pyrido[2,3-d]pyrimidin-7(8H)-one
5DEY	2.1	Serine/threonine-protein kinase PAK 1	59T	8-[(trans-5-amino-1,3-dioxan-2-yl)methyl]-6-[2-chloro-4-(6-methylpyridin-2-yl)phenyl]-2-(methylamino)pyrido[2,3-d]pyrimidin-7(8H)-one
5DFP	2.2	Serine/threonine-protein kinase PAK 1	59U	6-[2-chloro-4-(6-methylpyrazin-2-yl)phenyl]-8-ethyl-2-{[2-(1-methylpiperidin-4-yl)ethyl]amino}pyrido[2,3-d]pyrimidin-7(8H)-one
5DGZ	2.502	Serine/threonine-protein kinase pim-1	L20	(2S)-1-(1H-INDOL-3-YL)-3-{[5-(3-METHYL-1H-INDAZOL-5-YL)PYRIDIN-3-YL]OXY}PROPAN-2-AMINE
5DH3	2.468	Serine/threonine-protein kinase 3	5BS	4-[(5,10-dimethyl-6-oxo-6,10-dihydro-5H-pyrimido[5,4-b]thieno[3,2-e][1,4]diazepin-2-yl)amino]benzenesulfonamide
5DHJ	2.457	Serine/threonine-protein kinase pim-1	5E5	3-methyl-5-(pyridin-3-yl)-2H-pyrazolo[3,4-c]pyridine
5DIA	1.964	Serine/threonine-protein kinase pim-1	5E6	(1S,3S)-N-{6-[5-(pyridin-3-yl)-1H-pyrazolo[3,4-c]pyridin-3-yl]pyridin-2-yl}cyclohexane-1,3-diamine
5DLS	2.15	Serine/threonine-protein kinase Chk1	5CV	1-benzyl-N-(5-{5-[3-(dimethylamino)-2,2-dimethylpropoxy]-1H-indol-2-yl}-6-oxo-1,6-dihydropyridin-3-yl)-1H-pyrazole-4-carboxamide
5DRB	1.65	Serine/threonine-protein kinase WNK1	5FJ	N-tert-butyl-1-(1-{5-[5-(trifluoromethyl)-1,3,4-oxadiazol-2-yl]pyridin-2-yl}piperidin-4-yl)-1H-imidazole-5-carboxamide
5DWR	2	Serine/threonine-protein kinase pim-1	5H7	N-{4-[(1R,3S,5S)-3-amino-5-methylcyclohexyl]pyridin-3-yl}-6-(2,6-difluorophenyl)-5-fluoropyridine-2-carboxamide
5DXT	2.25	Phosphatidylinositol 4,5-bisphosphate 3-kinase	5H5	(2S)-2-({2-[1-(propan-2-yl)-1H-1,2,4-triazol-5-yl]-5,6-dihydroimidazo[1,2-d][1,4]benzoxazepin-9-yl}oxy)propanamide
5E1E	2.3	Tyrosine-protein kinase JAK1	5JG	6-chloro-2-(2-fluoro-4,5-dimethoxyphenyl)-N-(piperidin-4-ylmethyl)-3H-imidazo[4,5-b]pyridin-7-amine
5E1S	2.264	Insulin receptor	5JA	(5R)-N-(1-{2-[4-(2-methoxyethyl)piperazin-1-yl]ethyl}-1H-pyrazol-3-yl)-5,8-dimethyl-9-phenyl-6,8-dihydro-5H-pyrazolo[3,4-h]quinazolin-2-amine
5E7R	2.11	MAP kinase kinase kinase 7	5KW	2-chloro-N-{2-[(5-chloro-2-{[4-(4-methylpiperazin-1-yl)phenyl]amino}pyrimidin-4-yl)oxy]phenyl}acetamide
5E8Z	1.51	TGF-beta receptor type-1	5L4	3-amino-6-[4-(2-hydroxyethyl)phenyl]-N-[4-(morpholin-4-yl)pyridin-3-yl]pyrazine-2-carboxamide
5EH0	2.18	Dual specificity protein kinase TTK	5NW	N2-(2-Methoxy-4-(1-methyl-1H-pyrazol-4-yl)phenyl)-N8-neopentylpyrido[3,4-d]pyrimidine-2,8-diamine
5EHO	2.18	Dual specificity protein kinase TTK	5O1	~{N}8-cyclohexyl-~{N}2-[2-methoxy-4-(1-methylpyrazol-4-yl)phenyl]pyrido[3,4-d]pyrimidine-2,8-diamine
5EHY	2.26	Dual specificity protein kinase TTK	5O4	4-(furan-3-yl)-3-phenyl-2~{H}-pyrazolo[4,3-c]pyridine
5EI6	2.01	Dual specificity protein kinase TTK	5OQ	~{N}-(2,4-dimethoxyphenyl)-5-(1-methylpyrazol-4-yl)isoquinolin-3-amine
5EI8	2.17	Dual specificity protein kinase TTK	5OE	~{N}-[2-methoxy-4-(1-methylpyrazol-4-yl)phenyl]-8-(1-methylpyrazol-4-yl)pyrido[3,4-d]pyrimidin-2-amine
5EKO	2	MAP kinase 13	N17	3-(4-methyl-1H-imidazol-1-yl)-N-[4-(pyridin-4-yloxy)phenyl]benzamide
5EOB	1.75	Hepatocyte growth factor receptor	5QQ	6-[bis(fluoranyl)-[6-(4-fluorophenyl)-[1,2,4]triazolo[4,3-b][1,2,4]triazin-3-yl]methyl]quinoline

5EOL	2.2	Serine/threonine-protein kinase pim-1	5QO	macrocyclic quinoxaline-pyrrolodihydropiperidinone
5EW3	2.5	Vascular endothelial growth factor receptor 2	5T2	2-(pyridin-4-ylmethylamino)-~{N}-[3-(trifluoromethyl)phenyl]benzamide
5EW8	1.63	Fibroblast growth factor receptor 1	5SF	~{N}'-(3,5-dimethoxyphenyl)-~{N}'-[3-(1-methylpyrazol-4-yl)quinoxalin-6-yl]-~{N}-propan-2-yl-ethane-1,2-diamine
5EW9	2.181	Aurora kinase A	5VC	4-(3-chloranyl-2-fluoranyl-phenoxy)-1-[[6-(1,3-thiazol-2-ylamino)pyridin-2-yl]methyl]cyclohexane-1-carboxylic acid
5EYC	1.8	Hepatocyte growth factor receptor	5SZ	6-[(1~{R})-1-[8-fluoranyl-6-(3-methyl-1,2-oxazol-5-yl)-[1,2,4]triazolo[4,3-a]pyridin-3-yl]ethyl]-1,6-naphthyridin-5-one
5EYD	1.85	Hepatocyte growth factor receptor	5T1	6-[(1~{R})-1-[8-fluoranyl-6-(1-methylpyrazol-4-yl)-[1,2,4]triazolo[4,3-a]pyridin-3-yl]ethyl]-3-(2-methoxyethoxy)-1,6-naphthyridin-5-one
5EYK	1.93	Aurora kinase B-A	5U5	3-[(3~{Z})-3-[[[4-[(dimethylamino)methyl]phenyl]amino]-phenyl-methylidene]-2-oxidanylidene-1~{H}-indol-6-yl]-~{N}-ethyl-prop-2-ynamide
5EZR	2.5	cGMP-dependent protein kinase	4ZS	N-[5-(3-{2-[(cyclopropylmethyl)amino]pyrimidin-4-yl}-7-[(dimethylamino)methyl]-6-methylimidazo[1,2-a]pyridin-2-yl)-2-fluorophenyl]methanesulfonamide
5F4N	1.91	Serine/threonine-protein kinase Chk1	5UY	methyl 6-[(5-cyanopyrazin-2-yl)amino]-4-[[(2~{R})-morpholin-2-yl]methylamino]pyridine-3-carboxylate
5F9E	2	Protein kinase C	5VS	2,2-dimethyl-7-(2-oxidanylidene-3~{H}-imidazo[4,5-b]pyridin-1-yl)-1-(phenylmethyl)-3~{H}-quinazolin-4-one
5FBN	1.8	Tyrosine-protein kinase BTK	5WF	4-[8-azanyl-3-[(3~{R})-1-(3-methyloxetan-3-yl)carbonylpiperidin-3-yl]imidazo[1,5-a]pyrazin-1-yl]-~{N}-[4-(trifluoromethyl)pyridin-2-yl]benzamide
5FBO	1.894	Tyrosine-protein kinase BTK	5WH	4-[8-azanyl-3-[(3~{R},6~{S})-1-cyclopropylcarbonyl-6-methyl-piperidin-3-yl]imidazo[1,5-a]pyrazin-1-yl]-3-fluoranyl-~{N}-[4-(trifluoromethyl)pyridin-2-yl]benzamide
5FDP	2.25	Epithelial discoidin domain-containing receptor 1	5WR	(4~{S})-4-methyl-~{N}-[3-[(4-methylpiperazin-1-yl)methyl]-5-(trifluoromethyl)phenyl]-2-pyrimidin-5-yl-3,4-dihydro-1~{H}-isoquinoline-7-carboxamide
5FGK	2.36	Cyclin-dependent kinase 8	5XG	8-[3-(3-azanyl-2~{H}-indazol-6-yl)-5-chloranyl-pyridin-4-yl]-2,8-diazaspiro[4.5]decan-1-one
5FI4	2.5	Phosphatidylinositol 4,5-bisphosphate 3-kinase	5XV	~{N}-[6-[5-azanyl-6-[(2~{S})-1,1,1-tris(fluoranyl)propan-2-yl]oxy-pyrazin-2-yl]imidazo[1,2-a]pyridin-2-yl]ethanamide
5FP6	1.85	Cyclin-dependent kinase 2	MFZ	3-(4,7-dichloro-1H-indol-3-yl)prop-2-yn-1-ol
5FRI	2	TGF-beta receptor type-1	ZUQ	N-[4-[(6-chloro-[1,3]dioxolo[4,5-b]pyridin-7-yl)amino]-2-pyridyl]cyclopropanecarboxamide
5FTQ	1.7	ALK tyrosine kinase receptor	U4W	N-[5-(3,5-DIFLUOROBENZYL)-1H-INDAZOL-3-YL]-2-[(4-HYDROXYCYCLOHEXYL)AMINO]-4-(4-METHYLPIPERAZIN-1-YL) BENZAMIDE
5FXQ	2.3	Insulin-like growth factor 1 receptor	GD5	5-chloranyl-4-imidazo[1,2-a]pyridin-3-yl-N-(5-methyl-1-piperidin-4-yl-pyrazol-4-yl)pyrimidin-2-amine
5FXR	2.4	Insulin-like growth factor 1 receptor	8LN	5-chloranyl-4-imidazo[1,2-a]pyridin-3-yl-N-(3-methyl-1-piperidin-4-yl-pyrazol-4-yl)pyrimidin-2-amine
5FXS	1.9	Insulin-like growth factor 1 receptor	OZN	2-[4-[4-[[(6Z)-5-chloranyl-6-pyrazolo[1,5-a]pyridin-3-ylidene-1H-pyrimidin-2-yl]amino]-3,5-dimethyl-pyrazol-1-yl]piperidin-1-yl]-N,N-dimethyl-ethanamide
5G55	2.45	Phosphatidylinositol 4,5-bisphosphate 3-kinase	3QH	6-cyano-4-[[(1R)-1-(4-methylphenyl)ethyl]amino]quinoline-3-carboxamide
5GNK	1.796	Epidermal growth factor receptor	80U	1-[(3R)-3-[4-azanyl-3-[3-chloranyl-4-[(1-methylimidazol-2-yl)methoxy]phenyl]pyrazolo[3,4-d]pyrimidin-1-yl]piperidin-1-yl]prop-2-en-1-one
5GRN	1.77	Platelet-derived growth factor receptor alpha	748	N-[2-(dimethylamino)ethyl]-N-[[4-[[4-methyl-3-[(4-pyridin-3-ylpyrimidin-2-yl)amino]phenyl]carbamoyl]phenyl]methyl]pyridine-3-carboxamide
5GZA	2	Protein O-mannose kinase	ZZ1	4-METHYL-2H-CHROMEN-2-ONE
5H09	1.945	Tyrosine-protein kinase HCK	OOO	ethyl (2~{S})-2-[[4-[4-azanyl-5-(4-phenoxyphenyl)pyrrolo[2,3-d]pyrimidin-7-yl]cyclohexyl]amino]-4-methyl-pentanoate
5H0B	1.651	Tyrosine-protein kinase HCK	OOQ	(2~{S})-2-[[4-[4-azanyl-5-(4-phenoxyphenyl)pyrrolo[2,3-d]pyrimidin-7-yl]cyclohexyl]azaniumyl]-4-methyl-pentanoate
5H0E	2.1	Tyrosine-protein kinase HCK	OOS	(2~{S})-2-[[4-[4-azanyl-5-(4-phenoxyphenyl)pyrrolo[2,3-d]pyrimidin-7-yl]cyclohexyl]amino]-4-methyl-pentanamide
5H0G	1.8	Tyrosine-protein kinase HCK	OOU	(2~{S})-2-[[4-[4-azanyl-5-(4-phenoxyphenyl)pyrrolo[2,3-d]pyrimidin-7-yl]cyclohexyl]amino]-~{N},4-dimethyl-pentanamide
5H0H	1.72	Tyrosine-protein kinase HCK	OOV	(2~{S})-2-[[4-[4-azanyl-5-(4-phenoxyphenyl)pyrrolo[2,3-d]pyrimidin-7-yl]cyclohexyl]amino]-~{N},~{N},4-trimethyl-pentanamide
5H3Q	2.1	High affinity nerve growth factor receptor	7HF	1-[(3S,4R)-4-[3,4-bis(fluoranyl)phenyl]-1-(2-methoxyethyl)pyrrolidin-3-yl]-3-(5-ethoxy-4-methyl-2-phenyl-pyrazol-3-yl)urea
5H8E	2.15	Casein kinase II	5Y3	~{N}-[2-[2-azanylethyl(methyl)amino]-5-[[3-cyano-7-(cyclopropylamino)pyrazolo[1,5-a]pyrimidin-5-yl]amino]phenyl]ethanamide
5H8G	2	Casein kinase II	5Y4	~{N}-[5-[[3-cyano-7-(cyclopropylamino)pyrazolo[1,5-a]pyrimidin-5-yl]amino]-2-[2-(dimethylamino)ethyl-methyl-amino]phenyl]ethanamide
5HBE	2.38	Cyclin-dependent kinase 8	5Y6	8-[3-chloranyl-5-[1-methyl-2,2-bis(oxidanylidene)-3~{H}-2,1-benzothiazol-5-yl]pyridin-4-yl]-1-oxa-3,8-diazaspiro[4.5]decan-2-one
5HBH	2.5	Cyclin-dependent kinase 8	5Y7	5-[5-chloranyl-4-[1-(2-methoxyethyl)-1,8-diazaspiro[4.5]decan-8-yl]pyridin-3-yl]-1-methyl-3~{H}-2,1-benzothiazole 2,2-dioxide
5HCY	2.46	Epidermal growth factor receptor	60D	6-[[2-(1-cyclopropylsulfonylpyrazol-4-yl)pyrimidin-4-yl]amino]-~{N}-(oxan-4-yl)-1-propan-2-yl-pyrrolo[3,2-c]pyridine-3-carboxamide
5HG5	1.52	Epidermal growth factor receptor	633	N-{3-[(2-{[4-(4-methylpiperazin-1-yl)phenyl]amino}-7H-pyrrolo[2,3-d]pyrimidin-4-yl)oxy]phenyl}propanamide
5HG7	1.85	Epidermal growth factor receptor	630	1-{(3R,4R)-3-[({5-chloro-2-[(1-methyl-1H-pyrazol-4-yl)amino]-7H-pyrrolo[2,3-d]pyrimidin-4-yl}oxy)methyl]-4-methoxypyrrolidin-1-yl}propan-1-one
5HG8	1.42	Epidermal growth factor receptor	634	N-[3-({2-[(1-methyl-1H-pyrazol-4-yl)amino]-7H-pyrrolo[2,3-d]pyrimidin-4-yl}oxy)phenyl]propanamide
5HG9	2.15	Epidermal growth factor receptor	63A	1-[(3R,4R)-3-[({2-[(1-methyl-1H-pyrazol-4-yl)amino]-7H-pyrrolo[2,3-d]pyrimidin-4-yl}oxy)methyl]-4-(trifluoromethyl)pyrrolidin-1-yl]propan-1-one
5HHW	1.79	Insulin receptor	60O	7-[3-(azetidin-1-ylmethyl)cyclobutyl]-5-[3-[[(2~{R})-oxan-2-yl]methoxy]phenyl]pyrrolo[2,3-d]pyrimidin-4-amine
5HID	2.5	Serine/threonine-protein kinase B-raf	B1E	3-(2-cyanopropan-2-yl)-N-{4-methyl-3-[(3-methyl-4-oxo-3,4-dihydroquinazolin-6-yl)amino]phenyl}benzamide
5HKM	2.1	3-phosphoinositide-dependent protein kinase 1	61Y	4-ethyl-6-[5-(1H-pyrazol-4-yl)-1H-pyrrolo[2,3-b]pyridin-3-yl]pyrimidin-2-amine
5HLP	2.45	Glycogen synthase kinase-3 beta	65A	4-(2-methoxyphenyl)-3,7,7-trimethyl-1,6,7,8-tetrahydro-5H-pyrazolo[3,4-b]quinolin-5-one
5HLW	1.97	Hepatocyte growth factor receptor	62E	1-[2-(1-ethylpiperidin-4-yl)ethyl]-3-(6-{[6-(thiophen-2-yl)[1,2,4]triazolo[4,3-b]pyridazin-3-yl]sulfanyl}-1,3-benzothiazol-2-yl)urea
5HNB	2.35	Cyclin-dependent kinase 8	62M	[6-hydroxy-3-(3-methylbenzyl)-1H-indazol-5-yl][(3S)-3-hydroxypyrrolidin-1-yl]methanone
5HNI	1.71	Hepatocyte growth factor receptor	63B	methyl (6-{[6-(4-fluorophenyl)[1,2,4]triazolo[4,3-b]pyridazin-3-yl]sulfanyl}-1H-benzimidazol-2-yl)carbamate
5HO6	1.97	Hepatocyte growth factor receptor	63K	1-(6-{[6-(4-fluorophenyl)[1,2,4]triazolo[4,3-b]pyridazin-3-yl]sulfanyl}-1,3-benzothiazol-2-yl)-3-[2-(morpholin-4-yl)ethyl]urea
5HQ0	2.3	Cyclin-dependent kinase 1	LZ9	{[(2,6-difluorophenyl)carbonyl]amino}-N-(4-fluorophenyl)-1H-pyrazole-3-carboxamide
5HTI	1.66	Hepatocyte growth factor receptor	66L	N-[3-fluoro-4-({7-[2-(morpholin-4-yl)ethoxy]-1,6-naphthyridin-4-yl}oxy)phenyl]-N'-(4-fluorophenyl)cyclopropane-1,1-dicarboxamide
5HU9	1.529	Tyrosine-protein kinase ABL1	66K	4-[(4-methylpiperazin-1-yl)methyl]-N-(4-methyl-3-{[1-(pyridin-3-ylcarbonyl)piperidin-4-yl]oxy}phenyl)-3-(trifluoromethyl)benzamide
5HVY	2.39	Cyclin-dependent kinase 8	66X	N-{(3S)-1-[2-(methylamino)pyrimidin-4-yl]pyrrolidin-3-yl}-N'-{4-[(morpholin-4-yl)methyl]-3-(trifluoromethyl)phenyl}urea
5HX6	2.23	Receptor-interacting serine/threonine-protein	65U	5-benzyl-N-[(3S)-5-methyl-4-oxo-2,3,4,5-tetrahydro-1,5-benzoxazepin-3-yl]-1,2-oxazole-3-carboxamide

5HX8	2.2	Tyrosine-protein kinase JAK1	66P	4-[(4-aminocyclohexyl)amino]-3-(1H-benzimidazol-2-yl)-1H-pyridin-2-one
5HZE	2.4	Dual specificity MAP kinase kinase 1	E62	(3S,4R,8S,9S,11E)-8,9,16-trihydroxy-3,4-dimethyl-14-(methylamino)-3,4,5,6,9,10-hexahydro-1H-2-benzoxacyclotetradecine-1,7(8H)-dione
5HZN	2.2	Insulin-like growth factor 1 receptor	66A	7-[cis-3-(azetidin-1-ylmethyl)cyclobutyl]-5-[3-(benzyloxy)phenyl]-7H-pyrrolo[2,3-d]pyrimidin-4-amine
5I3O	2.4	BMP-2-inducible protein kinase	IDV	N-(6-{3-[(dimethylsulfamoyl)amino]phenyl}-1H-indazol-3-yl)cyclopropanecarboxamide
5I3R	2.4	BMP-2-inducible protein kinase	IDK	N-[6-(3-{[(cyclopropylmethyl)sulfonyl]amino}phenyl)-1H-indazol-3-yl]cyclopropanecarboxamide
5I4U	2.372	Phosphatidylinositol 4,5-bisphosphate 3-kinase	67T	2,4-diamino-6-{[(1S)-1-(5-chloro-4-oxo-3-phenyl-3,4-dihydroquinazolin-2-yl)ethyl]amino}pyrimidine-5-carbonitrile
5I8A	2.33	High affinity nerve growth factor receptor	69C	(6R)-3-(methylsulfanyl)-6-phenyl-1-(1H-pyrazol-3-yl)-6,7-dihydrothieno[3,4-c]pyridin-4(5H)-one
5IA0	1.948	Ephrin type-A receptor 2	A5B	alisertib
5IA1	2.036	Ephrin type-A receptor 2	ZZL	4-{[9-CHLORO-7-(2,6-DIFLUOROPHENYL)-5H-PYRIMIDO[5,4-D][2]BENZAZEPIN-2-YL]AMINO}BENZOIC ACID
5IA3	1.788	Ephrin type-A receptor 2	P17	6-(2,6-DICHLORO-PHENYL)-8-METHYL-2-(3-METHYLSULFANYL-PHENYLAMINO)-8H-PYRIDO[2,3-D]PYRIMIDIN-7-ONE
5IA5	1.776	Ephrin type-A receptor 2	GV0	golvatinib
5ICP	2.18	Cyclin-dependent kinase 8	69Z	[(2S)-2-(4-chlorophenyl)pyrrolidin-1-yl](5-methylimidazo[5,1-b][1,3,4]thiadiazol-2-yl)methanone
5IDN	2.26	Cyclin-dependent kinase 8	6A7	[(2S)-2-(4-chlorophenyl)pyrrolidin-1-yl](3-methyl-1H-pyrazolo[3,4-b]pyridin-5-yl)methanone
5IEV	2.03	Cyclin-dependent kinase 2	R0N	Roniciclib
5IEX	2.03	Cyclin-dependent kinase 2	6AF	(2R,3R)-3-[(5-bromo-2-{[4-(S-cyclopropylsulfonimidoyl)phenyl]amino}pyrimidin-4-yl)oxy]butan-2-ol
5IEY	1.66	Cyclin-dependent kinase 2	6AE	4-[(4-{[(2R,3R)-3-hydroxybutan-2-yl]amino}pyrimidin-2-yl)amino]benzene-1-sulfonamide
5IH5	2.25	Casein kinase I	AUE	6-(3-chlorophenyl)pteridine-2,4,7-triamine
5IH6	2.3	Casein kinase I	AUG	6-(3-bromophenyl)pteridine-2,4,7-triamine
5IH8	1.85	Maternal embryonic leucine zipper kinase	6BJ	N-[(pyridin-2-yl)methyl]-4-[4-(pyridin-4-yl)-1H-pyrazol-1-yl]benzamide
5IH9	1.79	Maternal embryonic leucine zipper kinase	6BF	1-methyl-4-[4-(4-{3-[(piperidin-4-yl)methoxy]pyridin-4-yl}-1H-pyrazol-1-yl)phenyl]piperazine
5IHA	1.96	Maternal embryonic leucine zipper kinase	6BE	1-methyl-4-(4-{4-[3-(2-methylpropoxy)pyridin-4-yl]-1H-pyrazol-1-yl}phenyl)piperazine
5IHC	2.14	Maternal embryonic leucine zipper kinase	6BB	4-[1-(2-fluorophenyl)-1H-pyrazol-4-yl]-3-[(piperidin-4-yl)methoxy]pyridine
5IIS	2.1	Serine/threonine-protein kinase pim-1	6CB	3-amino-N-(2'-amino-6'-methyl[4,4'-bipyridin]-3-yl)-6-(2-fluorophenyl)pyridine-2-carboxamide
5IKW	2.41	BMP-2-inducible protein kinase	6BU	N-(6-{3-[(cyclopropylsulfonyl)amino]phenyl}-1H-indazol-3-yl)cyclopropanecarboxamide
5IME	2.217	Serine/threonine-protein kinase PAK 1	6BZ	8-(3-aminopropyl)-6-[2-chloro-4-(3-methyl-2-oxopyrazin-1(2H)-yl)phenyl]-2-(methylamino)pyrido[2,3-d]pyrimidin-7(8H)-one
5IMX	2.12	ALK tyrosine kinase receptor	CZ4	5-chloro-N~2~-{5-methyl-4-(1-methylpiperidin-4-yl)-2-[(propan-2-yl)oxy]phenyl}-N~4~-{1-methyl-3-[(propan-2-yl)sulfonyl]-1H-pyrazol-4-yl}pyrimidine-2,4-diamine
5IPJ	2.1	Serine/threonine-protein kinase pim-1	6CD	2-(tert-butylamino)-3-methyl-8-[(6R)-6-methyl-4-oxo-1,4,5,6-tetrahydropyrrolo[3,4-b]pyrrol-2-yl]quinazolin-4(3H)-one
5ITA	1.95	Serine/threonine-protein kinase B-raf	6DC	N-{2-cyano-3-[(3-methyl-4-oxo-3,4-dihydroquinazolin-6-yl)amino]phenyl}propane-1-sulfonamide
5IUG	1.93	ALK tyrosine kinase receptor	729	N-[3-(4-{[(5-tert-butyl-1,2-oxazol-3-yl)carbamoyl]amino}-3-methylphenyl)-1H-pyrazol-5-yl]-4-[(4-methylpiperazin-1-yl)methyl]benzamide
5IUH	2.1	ALK tyrosine kinase receptor	34Y	4-[(4-methylpiperazin-1-yl)methyl]-N-{3-[3-methyl-4-({[5-(propan-2-yl)-1,2-oxazol-3-yl]carbamoyl}amino)phenyl]-1H-pyrazol-5-yl}benzamide
5IUI	1.88	ALK tyrosine kinase receptor	45Q	N-[3-(4-amino-3-methylphenyl)-1H-pyrazol-5-yl]-4-[(4-methylpiperazin-1-yl)methyl]benzamide
5J1W	2.42	Dual specificity protein kinase CLK1	6FB	pyrido[3,4-g]quinazolin-2-amine
5J5S	2.153	Proto-oncogene tyrosine-protein kinase Src	6G3	N-{4-[8-amino-3-(propan-2-yl)imidazo[1,5-a]pyrazin-1-yl]naphthalen-1-yl}-N'-[3-(trifluoromethyl)phenyl]urea
5J7S	2.368	MAP kinase kinase kinase 7	6H3	N-{2-[(5-chloro-2-{[4-(4-methylpiperazin-1-yl)phenyl]amino}pyrimidin-4-yl)amino]phenyl}propanamide
5J87	1.59	Tyrosine-protein kinase BTK	N42	N-[3-(5-{[3-(acryloylamino)-4-(morpholine-4-carbonyl)phenyl]amino}-1-methyl-6-oxo-1,6-dihydropyridin-3-yl)-2-methylphenyl]-4-tert-butylbenzamide
5J8I	2.404	MAP kinase kinase kinase 7	6H4	N-{2-[(5-chloro-2-{[4-(4-methylpiperazin-1-yl)phenyl]amino}pyrimidin-4-yl)oxy]phenyl}prop-2-enamide
5J95	2.5	MAP kinase kinase kinase kinase 4	6HH	1-{4-[6-amino-5-(4-chlorophenyl)pyridin-3-yl]phenyl}cyclopentane-1-carboxylic acid
5J9Z	2.5	Epidermal growth factor receptor	6HJ	(R)-1-(3-(4-amino-3-(1-methyl-1H-indol-3-yl)-1H-pyrazolo[3,4-d]pyrimidin-1-yl)piperidin-1-yl)prop-2-en-1-one
5JFS	2.07	High affinity nerve growth factor receptor	6K0	N-{4-[4-amino-7-(propan-2-yl)-7H-pyrrolo[2,3-d]pyrimidine-5-carbonyl]pyridin-2-yl}-N'-(2,4-difluorophenyl)urea
5JFV	1.59	High affinity nerve growth factor receptor	6K1	N-{5-[4-amino-7-(propan-2-yl)-7H-pyrrolo[2,3-d]pyrimidine-5-carbonyl]pyridin-3-yl}-2-(4-chlorophenyl)acetamide
5JFW	1.52	High affinity nerve growth factor receptor	6K2	2-(4-cyanophenyl)-N-{5-[7-(propan-2-yl)-7H-pyrrolo[2,3-d]pyrimidine-5-carbonyl]pyridin-3-yl}acetamide
5JFX	1.63	High affinity nerve growth factor receptor	6K4	N-{5-[2-amino-7-(1-hydroxy-2-methylpropan-2-yl)-7H-pyrrolo[2,3-d]pyrimidine-5-carbonyl]pyridin-3-yl}-2-(5-chloropyridin-2-yl)acetamide
5JGA	2	MAP kinase kinase kinase 7	6KC	N-[5-(4-methylpiperazine-1-carbonyl)[1,1'-biphenyl]-2-yl]-4-oxo-3,4-dihydrothieno[3,2-d]pyrimidine-7-carboxamide
5JH6	2.365	MAP kinase kinase kinase 7	T92	2-[(5-chloro-2-{[4-(4-methylpiperazin-1-yl)phenyl]amino}pyrimidin-4-yl)amino]phenyl prop-2-enoate
5JHB	2.48	Phosphatidylinositol 4,5-bisphosphate 3-kinase	6K5	[1-{4-[6-amino-4-(trifluoromethyl)pyridin-3-yl]-6-(morpholin-4-yl)-1,3,5-triazin-2-yl}-3-(chloromethyl)azetidin-3-yl]methanol
5JK3	2.371	MAP kinase kinase kinase 7	6L4	~{N}-[2-[5-chloranyl-2-[(1-methylpyrazol-4-yl)amino]pyrimidin-4-yl]oxyphenyl]prop-2-enamide
5JKG	2.352	Fibroblast growth factor receptor 4	6LF	2-[4-[E-2-[5-[(1R)-1-[3,5-bis(chloranyl)pyridin-4-yl]ethoxy]-1H-indazol-3-yl]ethenyl]pyrazol-1-yl]ethanol
5JMS	2.3	Calmodulin-domain protein kinase 1	6LP	N~1~-(4-{2-[(3-chlorophenyl)amino]pyrimidin-4-yl}pyridin-2-yl)ethane-1,2-diamine
5JN2	2.2	Calmodulin-domain protein kinase 1	6LO	N-[(3-{4-amino-5-[3-(benzyloxy)phenyl]-7H-pyrrolo[2,3-d]pyrimidin-7-yl}cyclobutyl)methyl]acetamide
5JQ5	1.94	Cyclin-dependent kinase 2	I74	(3R,4R)-4-[[[7-[(phenylmethyl)amino]-3-propan-2-yl-pyrazolo[1,5-a]pyrimidin-5-yl]amino]methyl]piperidin-3-ol
5JQ8	1.94	Cyclin-dependent kinase 2	I73	(3S,4S)-4-[[[7-[(phenylmethyl)amino]-3-propan-2-yl-pyrazolo[1,5-a]pyrimidin-5-yl]amino]methyl]piperidin-3-ol
5JRQ	2.287	Serine/threonine-protein kinase B-raf	6N9	(3-{[dihydroxy(propyl)-lambda~4~-sulfanyl]amino}-2,6-difluorophenyl)[5-(4-methoxyphenyl)-1H-pyrrolo[2,3-b]pyridin-3-yl]methanone
5JRS	1.97	Tyrosine-protein kinase BTK	6MV	4-[2-fluoro-3-(4-oxoquinazolin-3(4H)-yl)phenyl]-7-(2-hydroxypropan-2-yl)-9H-carbazole-1-carboxamide

5JSM	2.19	Serine/threonine-protein kinase B-raf	6NB	N,N'-{ethane-1,2-diylbis[oxyethane-2,1-diyloxy-4,1-phenylene-1H-pyrrolo[2,3-b]pyridine-5,3-diylcarbonyl(2,4-difluoro-3,1-phenylene)]}di(propane-1-sulfonamide)
5K00	1.77	Maternal embryonic leucine zipper kinase	6PV	4-{2-[(3-methoxyphenyl)amino]-4-[(piperidin-4-yl)methoxy]pyrimidin-5-yl}-N-[2-oxo-2-(phenylamino)ethyl]benzamide
5K0X	2.231	Tyrosine-protein kinase Mer	K0X	(7S)-7-amino-N-[(4-fluorophenyl)methyl]-8-oxo-2,9,16,18,21-pentaazabicyclo[15.3.1]henicosa-1(21),17,19-triene-20-carboxamide
5K3Y	1.6	Aurora kinase B-A	6Q4	N-methyl-N-(1-methylpiperidin-4-yl)-4-{[4-({(1R,2S)-2-[(propan-2-yl)carbamoyl]cyclopentyl}amino)-5-(trifluoromethyl)pyrimidin-2-yl]amino}benzamide
5K4J	1.6	Cyclin-dependent kinase 2	6QB	1-[(1~{S})-1-(4-chloranyl-3-fluoranyl-phenyl)-2-oxidanyl-ethyl]-4-[2-[(2-methylpyrazol-3-yl)amino]pyrimidin-4-yl]pyridin-2-one
5K5N	2.2	Glycogen synthase kinase-3 beta	6QH	5-(3-chloranyl-4-methoxy-phenyl)-~{N}-[3-(1,2,4-triazol-1-yl)propyl]-1,3-oxazole-4-carboxamide
5K72	2.22	Interleukin-1 receptor-associated kinase 4	6QY	~{N}4,~{N}4-dimethyl-~{N}1-[5-(oxan-4-yl)-7~{H}-pyrrolo[2,3-d]pyrimidin-4-yl]cyclohexane-1,4-diamine
5K75	2.03	Interleukin-1 receptor-associated kinase 4	6QX	~{N}1-(7,8-dihydro-6~{H}-cyclopenta[2,3]thieno[2,4-~{c}]pyrimidin-1-yl)-~{N}4,~{N}4-dimethyl-cyclohexane-1,4-diamine
5K7G	2.23	Interleukin-1 receptor-associated kinase 4	6R0	(3~{a}~{S},7~{a}~{R})-1-methyl-5-[4-[[5-(oxan-4-yl)-7~{H}-pyrrolo[2,3-d]pyrimidin-4-yl]amino]cyclohexyl]-3,3~{a},4,6,7,7~{a}-hexahydropyrrolo[3,2-c]pyridin-2-one
5K7I	2.31	Interleukin-1 receptor-associated kinase 4	6QZ	(3~{a}~{R},7~{a}~{S})-1-methyl-5-[4-[[5-(oxan-4-yl)-7~{H}-pyrrolo[2,3-d]pyrimidin-4-yl]amino]cyclohexyl]-3,3~{a},4,6,7,7~{a}-hexahydropyrrolo[3,2-c]pyridin-2-one
5K9I	2.5	Proto-oncogene tyrosine-protein kinase Src	O44	4-[(4-{4-[(3-cyclopropyl-1H-pyrazol-5-yl)amino]-6-[(prop-2-yn-1-yl)carbamoyl]pyrimidin-2-yl}piperazin-1-yl)methyl]benzene-1-sulfonyl fluoride
5KBR	2.36	Serine/threonine-protein kinase PAK 1	IPW	(4-chlorophenyl)-[5-(1-piperidin-4-ylpyrazol-4-yl)-1~{H}-pyrrolo[2,3-b]pyridin-3-yl]methanone
5KCX	2.2	Serine/threonine-protein kinase pim-1	6S3	4-chloranyl-1-methyl-2-[4-(4-methylpiperazin-1-yl)phenyl]pyrrolo[2,3-b]pyridine-6-carboxamide
5KE0	1.68	MAP kinase 1	6S9	1-[3-(2-methylpyridin-4-yl)-1~{H}-pyrazolo[4,3-c]pyridin-6-yl]-3-(phenylmethyl)urea
5KGD	1.98	Serine/threonine-protein kinase pim-1	6SL	2-pyridin-3-yl-1~{H}-benzimidazole
5KGE	2.23	Serine/threonine-protein kinase pim-1	6SN	5-(3,4-dichlorophenyl)-1~{H}-pyrazol-3-amine
5KGG	1.95	Serine/threonine-protein kinase pim-1	6SO	2-(5-chloranyl-1~{H}-indol-3-yl)ethanamine
5KGI	2.13	Serine/threonine-protein kinase pim-1	6SF	2-[3,4-bis(chloranyl)phenoxy]ethanamine
5KHX	2.4	Tyrosine-protein kinase JAK1	6TE	~{N}-[3-[methyl(7~{H}-pyrrolo[2,3-d]pyrimidin-4-yl)amino]cyclobutyl]methanesulfonamide
5KMI	1.87	High affinity nerve growth factor receptor	6UE	1-(9~{H}-fluoren-9-yl)-3-(2-methyl-4-phenyl-pyrimidin-5-yl)urea
5KMJ	2.04	High affinity nerve growth factor receptor	6UF	~{N}-(pyridin-2-ylmethyl)-2-(2-thiophen-2-ylindol-1-yl)ethanamide
5KMK	2.24	High affinity nerve growth factor receptor	6UG	2-fluoranyl-~{N}-[2-(4-fluorophenyl)-6-methyl-pyridin-3-yl]-4-(trifluoromethyl)benzamide
5KML	2.01	High affinity nerve growth factor receptor	6UH	1-(5-methyl-3-phenyl-1,2-oxazol-4-yl)-3-[[2-(trifluoromethyl)phenyl]methyl]urea
5KMM	2.12	High affinity nerve growth factor receptor	6UJ	1-(2-methyl-4-phenyl-pyrimidin-5-yl)-3-naphthalen-1-yl-urea
5KMN	2.14	High affinity nerve growth factor receptor	6UK	1-(2-methyl-4-phenyl-pyrimidin-5-yl)-3-[[2-(trifluoromethyl)phenyl]methyl]urea
5KO1	2.16	Mixed lineage kinase domain-like protein	6UY	[(1~{R})-2-[(4-fluorophenyl)amino]-2-oxidanylidene-1-phenyl-ethyl] 3-azanylpyrazine-2-carboxylate
5KPK	2.4	Glycogen synthase kinase-3 beta	6VK	(4~{S})-3-cyclopropyl-4,7,7-trimethyl-4-phenyl-2,6,8,9-tetrahydropyrazolo[3,4-b]quinolin-5-one
5KU8	2.22	Casein kinase II	6XK	~{N}-[2-[(1~{S},2~{S})-2-(aminomethyl)cyclopropyl]-5-[[3-cyano-7-(cyclopropylamino)pyrazolo[1,5-a]pyrimidin-5-yl]amino]phenyl]ethanamide
5KUP	1.389	Tyrosine-protein kinase BTK	6XL	6-~{tert}-butyl-8-fluoranyl-2-[3-(hydroxymethyl)-4-[1-methyl-6-oxidanylidene-5-(pyrimidin-4-ylamino)pyridin-3-yl]pyridin-2-yl]phthalazin-1-one
5KVT	2.45	High affinity nerve growth factor receptor	YMX	Entrectinib
5KWH	2.12	Casein kinase II	6XT	~{N}-[5-[[7-(cyclopropylamino)pyrazolo[1,5-a]pyrimidin-5-yl]amino]-2-methyl-phenyl]ethanamide
5KZ0	2.3	ALK tyrosine kinase receptor	6YL	2-[(1~{R})-1-[2-azanyl-5-(1,3-dimethylpyrazol-4-yl)pyridin-3-yl]oxyethyl]-4-fluoranyl-~{N},~{N}-dimethyl-benzamide
5KZI	2.1	Serine/threonine-protein kinase pim-1	6YN	~{N}-[4-[(3~{S})-3-azanylpiperidin-1-yl]pyridin-3-yl]-2-[2,6-bis(fluoranyl)phenyl]imidazo[1,5-b]pyridazin-7-amine
5L2S	2.27	Cyclin-dependent kinase 6	6ZV	N-{5-[(4-ethylpiperazin-1-yl)methyl]pyridin-2-yl}-5-fluoro-4-[4-fluoro-2-methyl-1-(propan-2-yl)-1H-benzimidazol-6-yl]pyrimidin-2-amine
5L2T	2.37	Cyclin-dependent kinase 6	6ZZ	7-cyclopentyl-N,N-dimethyl-2-{[5-(piperazin-1-yl)pyridin-2-yl]amino}-7H-pyrrolo[2,3-d]pyrimidine-6-carboxamide
5L3A	1.98	Tyrosine-protein kinase JAK2	6DP	~{N}-(1~{H}-indazol-4-yl)methanesulfonamide
5L4Q	1.97	AP2-associated protein kinase 1	LKB	~{N}-[5-(4-cyanophenyl)-1~{H}-pyrrolo[2,3-b]pyridin-3-yl]pyridine-3-carboxamide
5L6O	1.88	Ephrin type-B receptor 3	6P6	1-(4-phenylazanylquinazolin-7-yl)ethanone
5L6P	2.26	Ephrin type-B receptor 3	6P8	~{N}-(4-phenylazanylquinazolin-7-yl)ethanamide
5LAR	1.5	MAP kinase 14	6SH	5-azanyl-~{N}-[[4-[[(2~{S})-1-azanyl-4-cyclohexyl-1-oxidanylidene-butan-2-yl]carbamoyl]phenyl]methyl]-1-phenyl-pyrazole-4-carboxamide
5LCJ	1.78	MAP kinase 1	6TS	[(1~{R},4~{Z})-cyclooct-4-en-1-yl] ~{N}-[4-[4-[[5-chloranyl-4-[[2-(propanoylamino)phenyl]amino]pyrimidin-2-yl]amino]pyridin-2-yl]but-3-ynyl]carbamate
5LCK	1.89	MAP kinase 1	6TT	~{N}-[2-[[2-[(5-methoxypyridin-3-yl)amino]-5-(trifluoromethyl)pyrimidin-4-yl]amino]phenyl]propanamide
5LCP	1.433	cAMP-dependent protein kinase	M77	5-(1,4-DIAZEPAN-1-SULFONYL)ISOQUINOLINE
5LCQ	1.423	cAMP-dependent protein kinase	6TV	~{N}-[2-(propylamino)ethyl]isoquinoline-5-sulfonamide
5LCR	1.565	cAMP-dependent protein kinase	6TX	2-[isoquinolin-5-ylsulfonyl(propyl)amino]ethylazanium
5LCT	1.615	cAMP-dependent protein kinase	6TY	5-[(2~{R})-2-methylpiperazin-1-yl]sulfonylisoquinoline
5LCU	1.579	cAMP-dependent protein kinase	IQP	1-(5-ISOQUINOLINESULFONYL)-2-METHYLPIPERAZINE
5LMA	1.43	Tyrosine-protein kinase SYK	6ZG	~{N}'-[7-(4-methylphenyl)pyrido[3,4-b]pyrazin-5-yl]butane-1,4-diamine
5LMB	1.95	Tyrosine-protein kinase SYK	6ZF	7-[6-(dimethylamino)pyridin-3-yl]-~{N}-[[(3~{S})-piperidin-3-yl]methyl]pyrido[3,4-b]pyrazin-5-amine
5LMK	2.4	Cyclin-dependent kinase 2	6ZK	4-[4-[3-bromanyl-7-(pyridin-3-ylmethylamino)pyrazolo[1,5-a]pyrimidin-5-yl]phenyl]benzamide
5LQF	2.06	Cyclin-dependent kinase 1	4SP	O6-CYCLOHEXYLMETHOXY-2-(4'-SULPHAMOYLANILINO) PURINE
5LVL	1.4	3-phosphoinositide-dependent protein kinase 1	537	2,6-DIHYDROANTHRA/1,9-CD/PYRAZOL-6-ONE
5LWM	1.55	Tyrosine-protein kinase JAK3	79T	2-cyano-3-[5-(3-cyclohexyl-3,5,8,10-tetrazatricyclo[7.3.0.0^{2,6}]dodeca-1,4,6,8,11-pentaen-4-yl)furan-2-yl]-~{N},~{N}-dimethyl-prop-2-enamide

5LWN	1.6	Tyrosine-protein kinase JAK3	79R	(~{Z})-2-cyano-~{N},~{N}-dimethyl-3-[5-[3-[(1~{S},2~{R})-2-methylcyclohexyl]-3,5,8,10-tetrazatricyclo[7.3.0.0^{2,6}]dodeca-1,4,6,8,11-pentaen-4-yl]furan-2-yl]prop-2-enamide
5LXC	2.15	Dual specificity tyrosine-phosphorylation-	7AA	methyl 9-[(2,4-dichlorophenyl)amino]-[1,3]thiazolo[5,4-f]quinazoline-2-carboximidate
5M4C	1.935	Casein kinase II	7EY	3-[5-(4-methylphenyl)thieno[2,3-d]pyrimidin-4-yl]sulfanylpropanoic acid
5M4F	1.519	Casein kinase II	7FC	4-[6,8-bis(chloranyl)-3-oxidanyl-4-oxidanylidene-chromen-2-yl]benzoic acid
5M51	1.899	Serine/threonine-protein kinase NEK2	NU6	3-[[6-(cyclohexylmethoxy)-9~{H}-purin-2-yl]amino]benzamide
5M53	1.9	Serine/threonine-protein kinase NEK2	7GJ	3-[[6-(cyclohexylmethoxy)-7~{H}-purin-2-yl]amino]-~{N},~{N}-dimethyl-benzamide
5M55	2.4	Serine/threonine-protein kinase NEK2	7GG	6-[(~{Z})-2-(diethylamino)ethenyl]-~{N}-phenyl-7~{H}-purin-2-amine
5MAG	2.35	Maternal embryonic leucine zipper kinase	7KC	PF-3758309
5MAH	2	Maternal embryonic leucine zipper kinase	7KD	~{N}-methyl-4-[[4-[[3-[methyl(methylsulfonyl)amino]pyrazin-2-yl]methylamino]-5-(trifluoromethyl)pyrimidin-2-yl]amino]benzamide
5MHI	1.489	cAMP-dependent protein kinase	7N9	(5-chloranyl-2-methoxy-phenyl)methylazanium
5MHQ	1.3	Cyclin-dependent kinase 2	8QT	(2~{R},3~{S})-3-[[9-propan-2-yl-6-(pyridin-3-ylmethylamino)purin-2-yl]amino]pentan-2-ol
5MJA	2.14	Ephrin type-B receptor 1	7O3	2-chloranyl-~{N}-[4-[(2-chloranyl-5-oxidanyl-phenyl)amino]quinazolin-7-yl]ethanamide
5ML5	1.9	MAP kinase 14	1VI	3-(2,5-dimethoxyphenyl)-~{N}-[4-[4-(4-fluorophenyl)-2-methylsulfanyl-1~{H}-imidazol-5-yl]pyridin-2-yl]propanamide
5MMF	1.99	Casein kinase II	JMB	(3-chloranyl-4-phenyl-phenyl)methyl-propyl-azanium
5MMR	2	Casein kinase II	H83	~{N}'-[(3-chloranyl-4-phenyl-phenyl)methyl]butane-1,4-diamine
5MO5	2.04	Casein kinase II	4IH	~{N}-[3-[(3-chloranyl-4-phenyl-phenyl)methylamino]propyl]methanesulfonamide
5MO6	1.825	Casein kinase II	KXZ	3-[3-[(3-chloranyl-4-phenyl-phenyl)methylamino]propylamino]-3-oxidanylidene-propanoic acid
5MO7	2.15	Casein kinase II	YRA	3-[(3-chloranyl-4-phenyl-phenyl)methylamino]propanamide
5MO8	1.82	Casein kinase II	C98	3-[[3-[3-[(3-chloranyl-4-phenyl-phenyl)methylamino]propylamino]-3-oxidanylidene-propanoyl]amino]benzoic acid
5MOH	1.38	Casein kinase II	YTX	2-(3-methoxy-4-oxidanyl-phenyl)ethanoic acid
5MOT	2.09	Casein kinase II	HBD	4-HYDROXYBENZAMIDE
5MOV	2.2	Casein kinase II	HC4	4'-HYDROXYCINNAMIC ACID
5MOW	1.86	Casein kinase II	BR9	5-bromopyridine-2,3-diamine
5MQV	2.154	Casein kinase I	D5Q	4-(2,5-Dimethoxyphenyl)-N-(4-(5-(4-fluorphenyl)-2-(methylthio)-1H-imidazol-4-yl)-pyridin-2-yl)-1-methyl-1H-pyrrole-2-carboxamide
5MRB	2.2	Dual specificity protein kinase TTK	C5N	~{N}-(2,6-diethylphenyl)-8-[[2-methoxy-4-(4-methylpiperazin-1-yl)phenyl]amino]-1-methyl-4,5-dihydropyrazolo[4,3-h]quinazoline-3-carboxamide
5MTX	1.8	MAP kinase 14	FJI	3-[(3-benzamido-4-fluoranyl-phenyl)amino]-~{N}-(2-morpholin-4-ylethyl)-11-oxidanylidene-6~{H}-benzo[c][1]benzoxepine-9-carboxamide
5MTY	2.31	MAP kinase 14	HB9	~{N}-[2,4-bis(fluoranyl)-5-[[14-(2-hydroxyethylcarbamoyl)-2-oxidanylidene-6-tricyclo[9.4.0.0^{3,8}]pentadeca-1(15),3(8),4,6,11,13-hexaenyl]amino]phenyl]thiophene-2-carboxamide
5MXX	1.75	SRSF protein kinase 1	W4A	5-methyl-~{N}-[2-(4-methylpiperazin-1-yl)-5-(trifluoromethyl)phenyl]furan-2-carboxamide
5MY8	1.7	SRSF protein kinase 1	RXZ	SPHINX31
5MZ3	2.15	MAP kinase 14	8EN	~{N}-[3-(2-acetamidoimidazo[1,2-a]pyridin-6-yl)-4-methyl-phenyl]-3-(trifluoromethyl)benzamide
5MZL	1.955	Serine/threonine-protein kinase pim-1	8ET	~{N}-quinolin-5-ylpyridine-3-carboxamide
5N1D	1.609	cAMP-dependent protein kinase	8FZ	(5-pyridin-3-yloxyfuran-2-yl)methanamine
5N1E	1.529	cAMP-dependent protein kinase	9NT	~{N}-(1,3-benzodioxol-5-yl)-2-piperidin-1-ium-1-yl-ethanamide
5N1G	1.14	cAMP-dependent protein kinase	8G5	(4R)-4-(2-amino-1,3-thiazol-4-yl)-1-oxaspiro[4.5]decan-2-one
5N1H	1.18	cAMP-dependent protein kinase	8G2	(4-methoxycarbonylphenyl)methylazanium
5N1K	1.8	cAMP-dependent protein kinase	489	(1S)-2-amino-1-(4-fluorophenyl)ethanol
5N1L	1.489	cAMP-dependent protein kinase	47V	2,5-dimethyl-N-(pyridin-4-yl)furan-3-carboxamide
5N1M	1.436	cAMP-dependent protein kinase	BJK	(4-methoxy-3-oxidanyl-phenyl)methylazanium
5N1N	1.405	cAMP-dependent protein kinase	8G8	2-chloranyl-9-methyl-purine
5N1O	1.8	cAMP-dependent protein kinase	8GB	1-[5-chloranyl-3-(chloromethyl)-2-oxidanyl-phenyl]ethanone
5N32	1.833	cAMP-dependent protein kinase	8K5	[azanyl-[(4-chlorophenyl)methylsulfanyl]methylidene]azanium
5N36	1.58	cAMP-dependent protein kinase	F05	2H-isoindol-1-amine
5N37	1.589	cAMP-dependent protein kinase	8JT	1,3-benzodioxol-5-ylmethyl(cyclopentyl)azanium
5N3A	1.404	cAMP-dependent protein kinase	46P	4-methyl-5-(1-methyl-1H-imidazol-2-yl)-1,3-thiazol-2-amine
5N3B	1.64	cAMP-dependent protein kinase	BJN	2-pyridin-3-ylethylazanium
5N3C	1.772	cAMP-dependent protein kinase	8M2	[azanyl(thiophen-3-yl)methylidene]azanium
5N3D	1.77	cAMP-dependent protein kinase	8NE	[azanyl-[4-(trifluoromethyl)phenyl]methylidene]azanium
5N3E	1.529	cAMP-dependent protein kinase	46L	6-(dimethylamino)pyridine-3-carboxylic acid
5N3F	1.68	cAMP-dependent protein kinase	463	N-[3-(aminomethyl)phenyl]acetamide
5N3G	1.16	cAMP-dependent protein kinase	8KB	(6~{R})-1,4-oxazepan-6-ol
5N3H	1.36	cAMP-dependent protein kinase	NCA	NICOTINAMIDE
5N3I	1.14	cAMP-dependent protein kinase	BJQ	[(2~{S})-1-methoxy-1-oxidanylidene-3-phenyl-propan-2-yl]azanium
5N3J	1.12	cAMP-dependent protein kinase	4NB	4-NITROBENZOIC ACID

5N3L	1.38	cAMP-dependent protein kinase	E5E	Noradrenaline
5N3O	1.32	cAMP-dependent protein kinase	5AO	3-(1,3-oxazol-5-yl)aniline
5N3P	1.58	cAMP-dependent protein kinase	5H1	1H-indol-5-ol
5N3Q	1.31	cAMP-dependent protein kinase	3AB	3-aminobenzamide
5N3T	1.209	cAMP-dependent protein kinase	8K2	5-chloranylthiophene-2-sulfonamide
5N4N	2.09	Serine/threonine-protein kinase pim-1	8M8	3,4-dimethyl-5-(1~{H}-1,2,4-triazol-3-yl)thiophene-2-carbonitrile
5N4O	2.22	Serine/threonine-protein kinase pim-1	8MB	(~{E})-3-(4-methylphenyl)prop-2-enoic acid
5N4R	2.13	Serine/threonine-protein kinase pim-1	8MQ	2-(azepan-1-yl)-1-(1~{H}-indol-3-yl)propan-1-one
5N4U	2.202	Serine/threonine-protein kinase pim-1	8MZ	5-(2-azanyl-1,3-thiazol-4-yl)-1,3-dihydrobenzimidazol-2-one
5N4V	1.85	Serine/threonine-protein kinase pim-1	8MW	2-cyclopropyl-4,5-dimethyl-thieno[2,3-d]pyrimidine-6-carboxylic acid
5N4X	2.2	Serine/threonine-protein kinase pim-1	8MT	4,5-bis(bromanyl)thiophene-2-carbohydrazide
5N4Z	2.257	Serine/threonine-protein kinase pim-1	8MK	(~{E})-4-(4-hydroxyphenyl)but-3-en-2-one
5N50	1.92	Serine/threonine-protein kinase pim-1	8MN	2-(4-chlorophenyl)sulfanylethanehydrazide
5N51	2.118	Serine/threonine-protein kinase pim-1	8N8	3,4-bis(bromanyl)thiophene-2-carboxylic acid
5N52	2.252	Serine/threonine-protein kinase pim-1	8N2	(~{E})-3-(2,3-dimethoxyphenyl)prop-2-enoic acid
5N5L	1.97	Serine/threonine-protein kinase pim-1	8NZ	5-bromanyl-1~{H}-indole-3-carboxylic acid
5N5M	2.21	Serine/threonine-protein kinase pim-1	8O8	~{N}-(isoquinolin-5-ylmethyl)-~{N}-methyl-2-[(3~{R})-pyrrolidin-3-yl]benzamide
5N65	2	MAP kinase 14	8OT	2-phenyl-~{N}4-(2-thiophen-2-ylethyl)quinazoline-4,7-diamine
5N7P	1.501	cAMP-dependent protein kinase	E9Q	5-azanyl-3-pyrrolidin-1-yl-1~{H}-pyrazole-4-carbonitrile
5N84	2.3	Dual specificity protein kinase TTK	8Q5	~{N}-cyclopropyl-4-[8-(2-methylpropylamino)imidazo[1,2-a]pyrazin-3-yl]benzamide
5N87	2.29	Dual specificity protein kinase TTK	N66	~{N}-(2,6-diethylphenyl)-2-[[2-methoxy-4-(4-methylpiperazin-1-yl)phenyl]amino]-5,6-dihydropyrimido[4,5-e]indolizine-7-carboxamide
5N93	2.1	Dual specificity protein kinase TTK	8QE	4-[[4-azanyl-6-(~{tert}-butylamino)-5-cyano-pyridin-2-yl]amino]benzamide
5N9K	1.643	Casein kinase II	8QK	1,3-bis(chloranyl)-6-[(~{E})-(4-methoxyphenyl)iminomethyl]dibenzofuran-2,7-diol
5N9L	1.79	Casein kinase II	8QH	(4~{Z})-6,7-bis(chloranyl)-4-[[(4-methylphenyl)amino]methylidene]-8-oxidanyl-1,2-dihydrodibenzofuran-3-one
5N9S	2.3	Dual specificity protein kinase TTK	8QW	(2~{R})-2-(4-fluorophenyl)-~{N}-[4-[2-[(2-methoxy-4-methylsulfonyl-phenyl)amino]-[1,2,4]triazolo[1,5-a]pyridin-6-yl]phenyl]propanamide
5NCY	1.9	Phosphatidylinositol 4,5-bisphosphate 3-kinase	8TK	4-azanyl-6-[[(1~{S})-1-(3-methyl-5-oxidanylidene-6-phenyl-[1,3]thiazolo[3,2-a]pyridin-7-yl)ethyl]amino]pyrimidine-5-carbonitrile
5NCZ	1.94	Phosphatidylinositol 4,5-bisphosphate 3-kinase	8TN	4-azanyl-6-[[(1~{S})-1-[6-[3-[(dimethylamino)methyl]phenyl]-3-methyl-5-oxidanylidene-[1,3]thiazolo[3,2-a]pyridin-7-yl]ethyl]amino]pyrimidine-5-carbonitrile
5NHF	2.14	MAP kinase 1	8X5	2-[2-(oxan-4-ylamino)pyrimidin-4-yl]-5-(phenylmethyl)-6,7-dihydro-1~{H}-pyrrolo[3,2-c]pyridin-4-one
5NHH	1.94	MAP kinase 1	8XH	5-(2-methoxyethyl)-2-[2-(oxan-4-ylamino)pyrimidin-4-yl]-6,7-dihydro-1~{H}-pyrrolo[3,2-c]pyridin-4-one
5NHJ	2.12	MAP kinase 1	8XE	5-(2-methoxyethyl)-2-[2-[(2-methylpyrazol-3-yl)amino]pyrimidin-4-yl]-6,7-dihydro-1~{H}-pyrrolo[3,2-c]pyridin-4-one
5NHL	2.07	MAP kinase 1	8XB	(6~{R})-5-(2-methoxyethyl)-6-methyl-2-[5-methyl-2-[(2-methylpyrazol-3-yl)amino]pyrimidin-4-yl]-6,7-dihydro-1~{H}-pyrrolo[3,2-c]pyridin-4-one
5NHO	2.24	MAP kinase 1	8XN	(6~{S})-5-(2-methoxyethyl)-6-methyl-2-[5-methyl-2-[(2-methylpyrazol-3-yl)amino]pyrimidin-4-yl]-6,7-dihydro-1~{H}-pyrrolo[3,2-c]pyridin-4-one
5NHP	1.99	MAP kinase 1	8XK	5-(2-methoxyethyl)-1-methyl-2-[2-[(2-methylpyrazol-3-yl)amino]pyrimidin-4-yl]-6,7-dihydropyrrolo[3,2-c]pyridin-4-one
5NHV	2	MAP kinase 1	8QB	7-[2-(oxan-4-ylamino)pyrimidin-4-yl]-3,4-dihydro-2~{H}-pyrrolo[1,2-a]pyrazin-1-one
5NJZ	1.768	Ephrin type-A receptor 2	8ZH	~{N}-(2-chloranyl-6-methyl-phenyl)-2-[[3-(piperidin-4-ylcarbamoyl)phenyl]amino]-1,3-thiazole-5-carboxamide
5NK0	1.597	Ephrin type-A receptor 2	91E	2-[[3-[(3-azanyl-2,2-dimethyl-propyl)carbamoyl]phenyl]amino]-~{N}-(2-chloranyl-6-methyl-phenyl)-1,3-thiazole-5-carboxamide
5NK1	1.548	Ephrin type-A receptor 2	90N	2-[[3-[(4-azanyl-6-methyl-1,3,5-triazin-2-yl)carbamoyl]phenyl]amino]-~{N}-(2-chloranyl-6-methyl-phenyl)-1,3-thiazole-5-carboxamide
5NK2	1.649	Ephrin type-A receptor 2	8ZZ	~{N}-(2-chloranyl-6-methyl-phenyl)-2-[[3-[[(3~{R},4~{R})-3-fluoranylpiperidin-4-yl]carbamoyl]phenyl]amino]-1,3-thiazole-5-carboxamide
5NK3	1.586	Ephrin type-A receptor 2	92Q	~{N}-(2-chloranyl-6-methyl-phenyl)-2-[[3-[[(3~{S})-pyrrolidin-3-yl]carbamoyl]phenyl]amino]-1,3-thiazole-5-carboxamide
5NK4	1.45	Ephrin type-A receptor 2	90E	2-[[3-[[(4~{R})-3,3-bis(fluoranyl)piperidin-4-yl]carbamoyl]phenyl]amino]-~{N}-(2-chloranyl-6-methyl-phenyl)-1,3-thiazole-5-carboxamide
5NK5	1.329	Ephrin type-A receptor 2	90K	2-[[3-[(3-aminophenyl)carbamoyl]phenyl]amino]-~{N}-(2-chloranyl-6-methyl-phenyl)-1,3-thiazole-5-carboxamide
5NK6	1.267	Ephrin type-A receptor 2	90W	~{N}-(2-chloranyl-6-methyl-phenyl)-2-[[3-[(4-oxidanylcyclohexyl)carbamoyl]phenyl]amino]-1,3-thiazole-5-carboxamide
5NK8	1.761	Ephrin type-A receptor 2	90Z	~{N}-(2-chloranyl-6-methyl-phenyl)-2-[[3-[(2,2,6,6-tetramethyl-1-oxidanyl-piperidin-4-yl)carbamoyl]phenyl]amino]-1,3-thiazole-5-carboxamide
5NK9	1.588	Ephrin type-A receptor 2	912	(2~{Z})-~{N}-(2-chloranyl-6-methyl-phenyl)-2-[3-[(4-methyl-4-oxidanyl-cyclohexyl)carbamoyl]phenyl]imino-1,3-thiazolidine-5-carboxamide
5NKA	1.377	Ephrin type-A receptor 2	91H	4-[[3-[[5-[(2-chloranyl-6-methyl-phenyl)carbamoyl]-1,3-thiazol-2-yl]amino]phenyl]carbonylamino]cyclohexane-1-carboxylic acid
5NKB	1.5	Ephrin type-A receptor 2	8ZT	~{N}-(2-chloranyl-6-methyl-phenyl)-2-[(3,5-dimorpholin-4-ylphenyl)amino]-1,3-thiazole-5-carboxamide
5NKC	1.448	Ephrin type-A receptor 2	90T	(3~{S})-1-[3-[[5-[(2-chloranyl-6-methyl-phenyl)carbamoyl]-1,3-thiazol-2-yl]amino]phenyl]carbonylpyrrolidine-3-carboxylic acid
5NKD	1.408	Ephrin type-A receptor 2	91K	2-[(3~{R})-1-[3-[[5-[(2-chloranyl-6-methyl-phenyl)carbamoyl]-1,3-thiazol-2-yl]amino]phenyl]carbonylpyrrolidin-3-yl]ethanoic acid
5NKE	1.39	Ephrin type-A receptor 2	90B	2-[[3-bromanyl-5-(piperidin-4-ylcarbamoyl)phenyl]amino]-~{N}-(2-chloranyl-6-methyl-phenyl)-1,3-thiazole-5-carboxamide
5NKF	1.099	Ephrin type-A receptor 2	8ZN	~{N}-(2-chloranyl-6-methyl-phenyl)-2-[[3-(piperidin-4-ylcarbamoyl)-5-(trifluoromethyl)phenyl]amino]-1,3-thiazole-5-carboxamide
5NKG	1.1	Ephrin type-A receptor 2	8ZK	~{N}-(2-chloranyl-6-methyl-phenyl)-2-[[3-ethyl-5-(piperidin-4-ylcarbamoyl)phenyl]amino]-1,3-thiazole-5-carboxamide
5NKH	1.29	Ephrin type-A receptor 2	8ZQ	2-[[3-(2-aminophenyl)-5-(piperidin-4-ylcarbamoyl)phenyl]amino]-~{N}-(2-chloranyl-6-methyl-phenyl)-1,3-thiazole-5-carboxamide
5NKI	1.675	Ephrin type-A receptor 2	8ZW	~{N}-(2-chloranyl-6-methyl-phenyl)-2-[(3-methylsulfonyl-5-morpholin-4-yl-phenyl)amino]-1,3-thiazole-5-carboxamide

5NQC	2	Casein kinase II	1KP	(3E)-6,7-dichloro-3-(hydroxyimino)-1,3-dihydro-2H-indol-2-one
5NXC	2.25	LIM domain kinase 1	9DB	(2~{R})-2-azanyl-2-cyclohexyl-~{N}-[2-(1-methylpyrazol-4-yl)-9-oxidanylidene-3,10,11-triazatricyclo[6.4.1.0^{4,13}]trideca-1,4,6,8(13),11-pentaen-6-yl]ethanamide
5NXD	1.9	LIM domain kinase 2	9D8	4-[(3-chlorophenyl)sulfamoyl]-~{N}-(phenylmethyl)-~{N}-propyl-benzamide
5O13	2.44	Serine/threonine-protein kinase pim-1	9G5	(3~{E})-5-chloranyl-3-[[5-[3-[(4-methyl-1,4-diazepan-1-yl)carbonyl]phenyl]furan-2-yl]methylidene]-1~{H}-indol-2-one
5O49	1.91	Fibroblast growth factor receptor 1	9K5	[(2~{R},3~{S},4~{R},5~{R})-5-(6-aminopurin-9-yl)-3,4-bis(oxidanyl)oxolan-2-yl]methyl 3-fluorosulfonylbenzoate
5O4A	2.01	Fibroblast growth factor receptor 1	9K8	[(2~{R},3~{S},4~{R},5~{R})-5-(6-aminopurin-9-yl)-3,4-bis(oxidanyl)oxolan-2-yl]methyl 4-ethyl-3-fluorosulfonyl-benzoate
5OMY	1.95	Casein kinase II	9YE	4-(3-methylbut-2-enoxy)-5-propan-2-yl-7,8-dihydro-6~{H}-indeno[1,2-b]indole-9,10-dione
5OP2	1.9	Serine/threonine-protein kinase Chk1	A0Q	5-(4-methylpiperazin-1-yl)-2-phenylmethoxy-~{N}-pyridin-3-yl-benzamide
5OP4	2	Serine/threonine-protein kinase Chk1	A0T	[4-[[4-(ethylamino)-5-(trifluoromethyl)pyrimidin-2-yl]amino]-2-fluoranyl-5-methoxy-phenyl]-morpholin-4-yl-methanone
5OP5	1.9	Serine/threonine-protein kinase Chk1	3FE	3-[4-(morpholin-4-yl)-7H-pyrrolo[2,3-d]pyrimidin-5-yl]benzonitrile
5OP7	1.8	Serine/threonine-protein kinase Chk1	A1K	[4-[[5-chloranyl-4-(methylamino)-7~{H}-pyrrolo[2,3-d]pyrimidin-2-yl]amino]-3-methoxy-phenyl]-morpholin-4-yl-methanone
5OPB	1.55	Serine/threonine-protein kinase Chk1	A1N	(2~{R},6~{S})-2,6-dimethyl-4-[6-[5-(1-methylcyclopropyl)oxy-1~{H}-indazol-3-yl]pyrimidin-4-yl]morpholine
5OPR	1.95	Serine/threonine-protein kinase Chk1	A3E	5-[4-(morpholin-4-ylmethyl)phenyl]-3-(1-propan-2-yl-1,2,3-triazol-4-yl)pyridin-2-amine
5OPS	2	Serine/threonine-protein kinase Chk1	A3Q	4-(3-hydroxyphenyl)-1~{H}-pyrrolo[2,3-b]pyridine-3-carbonitrile
5OPU	1.55	Serine/threonine-protein kinase Chk1	A3K	6-azanyl-4-(3-methylphenyl)-1~{H}-pyrrolo[2,3-b]pyridine-3-carbonitrile
5OPV	1.9	Serine/threonine-protein kinase Chk1	A3H	4-(3-methylphenyl)-6-[(1-methylpyrazol-3-yl)amino]-1~{H}-pyrrolo[2,3-b]pyridine-3-carbonitrile
5OQ5	1.4	Serine/threonine-protein kinase Chk1	4K4	2-[(2-methoxy-4-{[4-(4-methylpiperazin-1-yl)piperidin-1-yl]carbonyl}phenyl)amino]-5,11-dimethyl-5,11-dihydro-6H-pyrimido[4,5-b][1,4]benzodiazepin-6-one
5OSM	1.77	Cyclin-dependent kinase 2	AEQ	methyl 1-propanoyl-3,4-dihydro-2~{H}-quinoline-6-carboxylate
5OSZ	2	Casein kinase II	AHK	2-(1~{H}-benzimidazol-2-yl)ethyl-[[3-chloranyl-4-(2-ethylphenyl)phenyl]methyl]azanium
5OT5	1.63	Casein kinase II	AWK	~{N}-[[3-chloranyl-4-(2-ethylphenyl)phenyl]methyl]-2-(7-methyl-1~{H}-benzimidazol-2-yl)ethanamine
5OT6	1.94	Casein kinase II	AJK	(3-chloranyl-4-phenyl-phenyl)methyl-[2-(1~{H}-pyrrol-2-yl)ethyl]azanium
5OTD	1.57	Casein kinase II	AOW	[2-[2-[[3-chloranyl-4-(2-ethylphenyl)phenyl]methylamino]ethyl]-3~{H}-benzimidazol-4-yl]-oxidanyl-oxidanylidene-azanium
5OTE	1.68	Serine/threonine-protein kinase MRCK beta	AQE	2-[4-[(6~{S})-1,8-diazaspiro[5.5]undecan-8-yl]-1~{H}-pyrrolo[2,3-b]pyridin-3-yl]-1,3-thiazole
5OTF	2	Serine/threonine-protein kinase MRCK beta	AQ5	(6~{S})-8-(3-pyrimidin-4-yl-1~{H}-pyrrolo[2,3-b]pyridin-4-yl)-1,8-diazaspiro[5.5]undecane
5OTH	1.69	Casein kinase II	AQ8	~{N}-[[3-chloranyl-4-(2-ethylphenyl)phenyl]methyl]-2-(4-methoxy-1~{H}-benzimidazol-2-yl)ethanamine
5OTI	1.59	Casein kinase II	AOK	~{N}-[[3-chloranyl-4-(2-ethylphenyl)phenyl]methyl]-2-(5-methyl-1~{H}-benzimidazol-2-yl)ethanamine
5OTL	1.57	Casein kinase II	AQT	~{N}-[[3-chloranyl-4-(2-ethylphenyl)phenyl]methyl]-2-(5-methoxy-1~{H}-benzimidazol-2-yl)ethanamine
5OTO	1.51	Casein kinase II	AQW	2-(5-chloranyl-1~{H}-benzimidazol-2-yl)-~{N}-[[3-chloranyl-4-(2-ethylphenyl)phenyl]methyl]ethanamine
5OTQ	1.38	Casein kinase II	AUH	2-(1~{H}-benzimidazol-2-yl)-~{N}-[[4-(2-ethylphenyl)-3-methoxy-phenyl]methyl]ethanamine
5OUE	2.01	Casein kinase II	AW5	(3-chloranyl-4-phenyl-phenyl)methyl-[2-(1~{H}-imidazol-4-yl)ethyl]azanium
5OUL	1.34	Casein kinase II	AWE	[3-chloranyl-4-(3-fluorophenyl)phenyl]methanamine
5OUM	2.05	Casein kinase II	AVK	~{N}-[(3-chloranyl-4-phenyl-phenyl)methyl]-2-(1~{H}-imidazol-2-yl)ethanamine
5OUU	1.81	Casein kinase II	AWN	2-(1~{H}-benzimidazol-2-yl)-~{N}-[(3-chloranyl-4-phenyl-phenyl)methyl]ethanamine
5OXG	2.13	Activin receptor type-1	B4B	5-[6-(4-piperazin-1-ylphenyl)pyrazolo[1,5-a]pyrimidin-3-yl]quinoline
5OYF	1.54	Casein kinase II	B4Q	2-(1~{H}-benzimidazol-2-yl)-~{N}-[[4-(2-ethylphenyl)-3-methyl-phenyl]methyl]ethanamine
5P9F	1.71	Tyrosine-protein kinase BTK	2VL	N-{3-[6-({4-[(2R)-1,4-dimethyl-3-oxopiperazin-2-yl]phenyl}amino)-4-methyl-5-oxo-4,5-dihydropyrazin-2-yl]-2-methylphenyl}-4,5,6,7-tetrahydro-1-benzothiophene-2-carboxamide
5P9G	1.75	Tyrosine-protein kinase BTK	7G6	6-cyclopropyl-8-fluoranyl-2-[2-(hydroxymethyl)-3-[1-methyl-5-[[5-(4-methylpiperazin-1-yl)pyridin-2-yl]amino]-6-oxidanylidene-pyridin-3-yl]phenyl]isoquinolin-1-one
5P9H	1.95	Tyrosine-protein kinase BTK	7G7	6-~{tert}-butyl-8-fluoranyl-2-[3-(hydroxymethyl)-4-[1-methyl-5-[[5-(1-methylpiperidin-4-yl)pyridin-2-yl]amino]-6-oxidanylidene-pyridazin-3-yl]pyridin-2-yl]phthalazin-1-one
5P9I	1.11	Tyrosine-protein kinase BTK	1E8	1-{(3R)-3-[4-amino-3-(4-phenoxyphenyl)-1H-pyrazolo[3,4-d]pyrimidin-1-yl]piperidin-1-yl}prop-2-en-1-one
5P9J	1.08	Tyrosine-protein kinase BTK	8E8	1-[(3~{R})-3-[4-azanyl-3-(4-phenoxyphenyl)pyrazolo[3,4-d]pyrimidin-1-yl]piperidin-1-yl]propan-1-one
5P9K	1.28	Tyrosine-protein kinase BTK	7G8	4-[4-[[5-fluoranyl-4-[[3-(propanoylamino)phenyl]amino]pyrimidin-2-yl]amino]phenoxy]-~{N}-methyl-pyridine-2-carboxamide
5P9L	1.25	Tyrosine-protein kinase BTK	7G9	~{N}-[3-[[5-fluoranyl-2-[[4-(2-methoxyethoxy)phenyl]amino]pyrimidin-4-yl]amino]phenyl]propanamide
5P9M	1.41	Tyrosine-protein kinase BTK	7GB	6-azanyl-9-[(3~{R})-1-[(~{E})-but-2-enoyl]pyrrolidin-3-yl]-7-(4-phenoxyphenyl)purin-8-one
5SWH	2.5	Proto-oncogene tyrosine-protein kinase Src	71D	(2R)-3-[4-amino-5-(4-chlorophenyl)-7-(2-methoxyethyl)-7H-pyrrolo[2,3-d]pyrimidin-6-yl]-2-cyano-N-(propan-2-yl)propanamide
5T0P	2.5	Proto-oncogene tyrosine-protein kinase Src	73A	(2E)-3-[4-amino-5-(4-chlorophenyl)-7-(2-methoxyethyl)-7H-pyrrolo[2,3-d]pyrimidin-6-yl]-2-cyano-N-(propan-2-yl)prop-2-enamide
5T18	1.5	Tyrosine-protein kinase BTK	73T	6-Fluoro-5-(R)-(3-(S)-(8-fluoro-1-methyl-2,4-dioxo-1,2-dihydroquinazolin-3(4H)-yl)-2-methylphenyl)-2-(S)-(2-hydroxypropan-2-yl)-2,3,4,9-tetrahydro-1H-carbazole-8-carboxamide
5T1H	2.11	Casein kinase II	75E	7-(cyclopropylamino)-5-[3-(6-oxo-1,6-dihydropyridin-3-yl)thiophen-2-yl]pyrazolo[1,5-a]pyrimidine-3-carbonitrile
5T1S	2.3	Interleukin-1 receptor-associated kinase 4	76Q	5-[3-(3,5-dimethylphenyl)-4-[4-(methylamino)butyl]quinolin-6-yl]pyridin-3-ol
5T1T	2.34	Interleukin-1 receptor-associated kinase 4	76P	~{N},~{N}-dimethyl-4-(6-nitroquinazolin-4-yl)oxy-cyclohexan-1-amine
5T2B	2.3	Phosphatidylinositol 4,5-bisphosphate 3-kinase	74O	5-[1-methyl-5-(1-phenylcyclopropyl)-1,2,4-triazol-3-yl]-3-(1~{H}-pyrazol-4-yl)pyrazin-2-amine
5T2I	2.3	Phosphatidylinositol 4,5-bisphosphate 3-kinase	74J	1-[3-azanyl-6-[1-methyl-5-(1-phenylcyclopropyl)-1,2,4-triazol-3-yl]pyrazin-2-yl]pyrazole-4-carboxamide
5T3Q	2	Hepatocyte growth factor receptor	75H	N-{3-fluoro-4-[(7-methoxyquinolin-4-yl)oxy]phenyl}-1-(2-hydroxy-2-methylpropyl)-5-methyl-3-oxo-2-phenyl-2,3-dihydro-1H-pyrazole-4-carboxamide
5T6A	2.05	Calmodulin-domain protein kinase 1	5GA	4-(3-chloro-4-fluorophenyl)-5-(1,5-naphthyridin-2-yl)-1,3-thiazol-2-amine

5T6I	2.05	Calmodulin-domain protein kinase 1	5GB	4-(3-methylphenyl)-5-(1,5-naphthyridin-2-yl)-1,3-thiazol-2-amine
5T6K	2.4	Calmodulin-domain protein kinase 1	5G9	4-(3-chlorophenyl)-5-(1,5-naphthyridin-2-yl)-1,3-thiazol-2-amine
5T8O	2.41	MAP kinase kinase kinase 14	76Z	10-(3-methyl-3-oxidanyl-but-1-ynyl)-5,6-dihydroimidazo[1,2-d][1,4]benzoxazepine-2-carboxamide
5T8P	2.32	MAP kinase kinase kinase 14	774	6,7-dihydrothieno[4,5]oxepino[1,2-~{c}]pyridine-2-carboxamide
5TA6	2.5	Serine/threonine-protein kinase PLK1	79D	4-{[(6R)-7-cyano-5-cyclopentyl-6-ethyl-5,6-dihydroimidazo[1,5-f]pteridin-3-yl]amino}-3-methoxy-N-(1-methylpiperidin-4-yl)benzamide
5TBE	2.44	MAP kinase 14	78L	~{N}-[2,4-bis(fluoranyl)-5-[[9-(2-morpholin-4-ylethylcarbamoyl)-11-oxidanylidene-5,6-dihydrodibenzo[1,2-~{d}:1',2'-~{f}][7]annulen-3-yl]amino]phenyl]thiophene-2-carboxamide
5TC0	2.24	Tyrosine-protein kinase Mer	79Y	N-(2-{4-[(2S)-4-(methylsulfonyl)morpholin-2-yl]-1,3-thiazol-2-yl}phenyl)-1H-imidazole-2-carboxamide
5TCO	2.1	MAP kinase 14	79Q	3-[(3-benzamido-4-fluoranyl-phenyl)amino]-~{N}-(2-morpholin-4-ylethyl)-11-oxidanylidene-5,6-dihydrodibenzo[1,2-~{d}:1',2'-~{f}][7]annulene-9-carboxamide
5TE0	1.9	AP2-associated protein kinase 1	XIN	methyl (3Z)-3-{[(4-{methyl[(4-methylpiperazin-1-yl)acetyl]amino}phenyl)amino](phenyl)methylidene}-2-oxo-2,3-dihydro-1H-indole-6-carboxylate
5TEL	2.214	Serine/threonine-protein kinase pim-1	7AJ	4-chloro-2-{5,6-dimethoxy-1-[2-(4-methylpiperazin-1-yl)ethyl]-1H-indol-3-yl}-1H-pyrrolo[2,3-b]pyridine
5TEX	2.149	Serine/threonine-protein kinase pim-1	7AU	(4-{4-chloro-1-methyl-2-[4-(piperazin-1-yl)phenyl]-1H-pyrrolo[2,3-b]pyridin-3-yl}phenyl)methanol
5TF9	2.5	Serine/threonine-protein kinase WNK1	7AV	{2-[(4-chlorophenyl)methoxy]phenyl}{5-[2-(methylamino)-1,3-thiazol-4-yl]-2,3-dihydro-1H-indol-1-yl}methanone
5TIU	1.49	Tyrosine-protein kinase SYK	7CU	5-{[(2S)-2-aminopropyl]amino}-3-(1H-indol-2-yl)pyrazine-2-carboxamide
5TKD	1.92	Non-receptor tyrosine-protein kinase TYK2	7GL	6-[(3,5-dimethylphenyl)amino]-8-(methylamino)imidazo[1,2-b]pyridazine-3-carboxamide
5TO8	1.9849	Protein-tyrosine kinase 2-beta	7FM	25-(methylsulfonyl)-8-(trifluoromethyl)-5,17,18,21,22,23,24,25-octahydro-12H-7,11-(azeno)-16,13-(metheno)pyrido[3,2-i]pyrrolo[1,2-q][1,3,7,11,17]pentaazacyclohenicosin-20(6H)-one
5TOE	2.301	Serine/threonine-protein kinase pim-1	7GK	2-[4-(piperazin-1-yl)phenyl]-1H-pyrrolo[2,3-b]pyridine-4-carbonitrile
5TOZ	1.98	Tyrosine-protein kinase JAK3	7H4	1-{(2S,5R)-2-methyl-5-[(7H-pyrrolo[2,3-d]pyrimidin-4-yl)amino]piperidin-1-yl}propan-1-one
5TQ4	2.3	Tyrosine-protein kinase JAK2	7GY	6-(2-ethyl-4-hydroxyphenyl)-1H-indazole-3-carboxamide
5TQ5	2.3	Tyrosine-protein kinase JAK2	7GX	6-(2-ethyl-4-hydroxyphenyl)-N-(6-methylpyridin-3-yl)-1H-indazole-3-carboxamide
5TQ6	2.06	Tyrosine-protein kinase JAK2	7GV	{(3R,4R)-4-methyl-3-[methyl(7H-pyrrolo[2,3-d]pyrimidin-4-yl)amino]piperidin-1-yl}(pyrrolidin-1-yl)methanone
5TQ7	2.1	Tyrosine-protein kinase JAK2	7GT	{(3R,4R)-4-methyl-3-[methyl(7H-pyrrolo[2,3-d]pyrimidin-4-yl)amino]piperidin-1-yl}[(3R)-3-(phenylsulfonyl)pyrrolidin-1-yl]methanone
5TQ8	1.59	Tyrosine-protein kinase JAK2	7GS	{2-[6-(2-ethyl-5-fluoro-4-hydroxyphenyl)-2H-indazol-3-yl]-3,4,6,7-tetrahydro-5H-imidazo[4,5-c]pyridin-5-yl}[5-(piperidin-1-yl)pyrazin-2-yl]methanone
5TR6	1.93	Tyrosine-protein kinase SYK	7KG	6-{[(1R,2S)-2-aminocyclohexyl]amino}-7-fluoro-4-(1-methyl-1H-pyrazol-4-yl)-1,2-dihydro-3H-pyrrolo[3,4-c]pyridin-3-one
5TT7	1.77	Tyrosine-protein kinase SYK	7KF	2-{[(1R,2S)-2-aminocyclohexyl]amino}-4-[(3-methylphenyl)amino]-6,7-dihydro-5H-pyrrolo[3,4-d]pyrimidin-5-one
5TTS	2.34	Tyrosine-protein kinase JAK3	7KU	1-{(3R)-3-[(7H-pyrrolo[2,3-d]pyrimidin-4-yl)amino]piperidin-1-yl}propan-1-one
5TTU	1.72	Tyrosine-protein kinase JAK3	7KV	1-[(3aR,7aR)-1-(7H-pyrrolo[2,3-d]pyrimidin-4-yl)octahydro-6H-pyrrolo[2,3-c]pyridin-6-yl]propan-1-one
5TTV	1.93	Tyrosine-protein kinase JAK3	7KX	N-[3-(7H-pyrrolo[2,3-d]pyrimidin-4-yl)phenyl]propanamide
5TVT	2.28	Maternal embryonic leucine zipper kinase	7LV	9-(3,5-dichloro-4-hydroxyphenyl)-1-{trans-4-[(dimethylamino)methyl]cyclohexyl}-3-methyl-3,4-dihydropyrimido[5,4-c][1,5]naphthyridin-2(1H)-one
5TWL	2.42	Maternal embryonic leucine zipper kinase	H91	9-(3,5-dichloro-4-hydroxyphenyl)-1-{trans-4-[(dimethylamino)methyl]cyclohexyl}-3,4-dihydropyrimido[5,4-c]quinolin-2(1H)-one
5U6C	2.1	Tyrosine-protein kinase Mer	7YS	(10R)-7-amino-11-chloro-12-fluoro-1-(2-hydroxyethyl)-3,10,16-trimethyl-16,17-dihydro-1H-8,4-(azeno)pyrazolo[4,3-h][2,5,11]benzoxadiazacyclotetradecin-15(10H)-one
5U6I	1.69	MAP kinase 1	81Y	3-[2-(morpholin-4-yl)pyridin-4-yl]-5-[(propan-2-yl)oxy]-1H-indazole
5U9D	1.33	Tyrosine-protein kinase BTK	83P	(R)-N-methyl-2-(3-((quinoxalin-6-ylamino)methyl)furan-2-carbonyl)-2,3,4,9-tetrahydro-1H-pyrido[3,4-b]indole-3-carboxamide
5UAB	1.9	Hepatocyte growth factor receptor	84M	N-{6-[([1,2,4]triazolo[4,3-a]pyridin-3-yl)sulfanyl]imidazo[1,2-b]pyridazin-2-yl}cyclopropanecarboxamide
5UAD	2.25	Hepatocyte growth factor receptor	84P	N-(6-{[6-(1-methyl-1H-pyrazol-4-yl)-1H-benzotriazol-1-yl]methyl}imidazo[1,2-b]pyridazin-2-yl)cyclopropanecarboxamide
5UBR	2.4	Phosphatidylinositol 4,5-bisphosphate 3-kinase	85S	1-[4-(3-{4-amino-5-[1-(oxan-4-yl)-1H-pyrazol-5-yl]pyrrolo[2,1-f][1,2,4]triazin-7-yl}phenyl)piperazin-1-yl]ethan-1-one
5UG8	1.46	Epidermal growth factor receptor	8BP	N-[(3R,4R)-4-fluoro-1-{6-[(1-methyl-1H-pyrazol-4-yl)amino]-9-(propan-2-yl)-9H-purin-2-yl}pyrrolidin-3-yl]propanamide
5UG9	1.33	Epidermal growth factor receptor	8AM	N-[(3R,4R)-4-fluoro-1-{6-[(3-methoxy-1-methyl-1H-pyrazol-4-yl)amino]-9-(propan-2-yl)-9H-purin-2-yl}pyrrolidin-3-yl]propanamide
5UGA	1.82	Epidermal growth factor receptor	8BM	4-(4-{[2-{[(3S)-1-acetylpyrrolidin-3-yl]amino}-9-(propan-2-yl)-9H-purin-6-yl]amino}phenyl)-1-methylpiperazin-1-ium
5UGC	1.58	Epidermal growth factor receptor	8BS	N-[(3R,4R)-4-fluoro-1-{6-[(3-methoxy-1-methyl-1H-pyrazol-4-yl)amino]-9-methyl-9H-purin-2-yl}pyrrolidin-3-yl]propanamide
5UIS	2.5	Interleukin-1 receptor-associated kinase 4	8C1	4-{[(3R)-piperidin-3-yl]oxy}-6-[(propan-2-yl)oxy]quinoline-7-carboxamide
5UIT	1.84	Interleukin-1 receptor-associated kinase 4	8CD	1-{[(2S)-5-oxopyrrolidin-2-yl]methoxy}-7-[(propan-2-yl)oxy]isoquinoline-6-carboxamide
5UIU	2.02	Interleukin-1 receptor-associated kinase 4	8CG	1-{[(2S,3S,4S)-3-ethyl-4-fluoro-5-oxopyrrolidin-2-yl]methoxy}-7-methoxyisoquinoline-6-carboxamide
5UK8	2.5	Phosphatidylinositol 4,5-bisphosphate 3-kinase	8DV	(R)-4-(6-(1-(cyclopropylsulfonyl)cyclopropyl)-2-(1H-indol-4-yl)pyrimidin-4-yl)-3-methylmorpholine
5UKF	2.4	Serine/threonine-protein kinase VRK1	080	4-{[(Z)-(7-oxo-6,7-dihydro-8H-[1,3]thiazolo[5,4-e]indol-8-ylidene)methyl]amino}benzene-1-sulfonamide
5UKL	2.15	Beta-adrenergic receptor kinase 1	SIX	2-{5-[(3S,4R)-3-{[(2H-1,3-benzodioxol-5-yl)oxy]methyl}piperidin-4-yl]-2-fluorophenyl}-N-[2-(1H-pyrazol-4-yl)ethyl]acetamide
5UOX	2.5	MAP kinase kinase kinase 5	8GY	2-(6-{4-[(2R)-1-hydroxypropan-2-yl]-4H-1,2,4-triazol-3-yl}pyridin-2-yl)-6-[(propan-2-yl)oxy]-2,3-dihydro-1H-isoindol-1-one
5UQ0	2.3	Fibroblast growth factor receptor 1	WP1	2,2-dimethyl-2,3-dihydro-1-benzofuran-7-carboxamide
5UR1	2.2	Fibroblast growth factor receptor 1	YY9	3-(2,6-dichloro-3,5-dimethoxyphenyl)-1-{1-[4-(dimethylamino)but-2-enoyl]piperidin-4-yl}-7-(phenylamino)-3,4-dihydropyrimido[4,5-d]pyrimidin-2(1H)-one
5UT3	1.501	Tyrosine-protein kinase JAK2	IK1	5-PHENYL-2-UREIDOTHIOPHENE-3-CARBOXAMIDE
5UT5	1.9	Tyrosine-protein kinase JAK2	2HB	N-(5-{4-[(1,1-dioxidothiomorpholin-4-yl)methyl]phenyl}[1,2,4]triazolo[1,5-a]pyridin-2-yl)cyclopropanecarboxamide
5UT6	1.645	Tyrosine-protein kinase JAK2	8MY	4-({4-amino-6-[3-(hydroxymethyl)-1H-pyrazol-1-yl]pyrimidin-2-yl}amino)benzonitrile
5UU1	2	Serine/threonine-protein kinase VRK2	7DZ	(7S)-2-[(3,5-difluoro-4-hydroxyphenyl)amino]-5,7-dimethyl-8-(3-methylbutyl)-7,8-dihydropteridin-6(5H)-one
5UY6	1.7	Calcium/calmodulin-dependent protein kinase	8R4	2-cyclopentyl-4-(5-phenylfuro[2,3-b]pyridin-3-yl)benzoic acid

5UYJ	1.6	Calcium/calmodulin-dependent protein kinase	8R7	2-cyclopentyl-4-(7-methoxyquinolin-4-yl)benzoic acid
5V24	2.5	MAP kinase kinase kinase 5	8V7	2-[4-(propan-2-yl)-4H-1,2,4-triazol-3-yl]-N-(pyridin-2-yl)-1,3-thiazole-4-carboxamide
5V5N	2.006	MAP kinase kinase kinase 7	EDH	N~1~-(1-propyl-1,3-dihydro-2H-benzimidazol-2-ylidene)benzene-1,3-dicarboxamide
5V62	1.9	MAP kinase 1	FRZ	5-(2-PHENYLPYRAZOLO[1,5-A]PYRIDIN-3-YL)-1H-PYRAZOLO[3,4-C]PYRIDAZIN-3-AMINE
5V82	1.888	Serine/threonine-protein kinase pim-1	96Y	1-{6-[(3R)-4,4-difluoropiperidin-3-yl]pyridin-2-yl}-6-(6-methylpyrazin-2-yl)-1H-pyrazolo[4,3-c]pyridine
5VAL	2.26	Serine/threonine-protein kinase B-raf	92D	N-(3-tert-butylphenyl)-4-methyl-3-[6-(morpholin-4-yl)pyrimidin-4-yl]benzamide
5VAM	2.1	Serine/threonine-protein kinase B-raf	92J	N-{2-methyl-5'-(morpholin-4-yl)-6'-[(oxan-4-yl)oxy][3,3'-bipyridin]-5-yl}-3-(trifluoromethyl)benzamide
5VC5	1.93	Wee1-like protein kinase	96M	6-(2,6-dichlorophenyl)-2-({4-[2-(diethylamino)ethoxy]phenyl}amino)-8-methylpyrido[2,3-d]pyrimidin-7(8H)-one
5VCW	2.25	Membrane-associated tyrosine- and threonine-	93J	(2E)-N-{4-[(3-chloro-4-fluorophenyl)amino]-3-cyano-7-ethoxyquinolin-6-yl}-4-(dimethylamino)but-2-enamide
5VCZ	1.5	Membrane-associated tyrosine- and threonine-	XZN	4-[(3,5-DICHLORO-4-METHOXYPHENYL)AMINO]-6-METHOXY-7-[3-(4-METHYLPIPERAZIN-1-YL)PROPOXY]QUINOLINE-3-CARBONITRILE
5VD0	2.13	Membrane-associated tyrosine- and threonine-	8X7	1-[6-(2-hydroxypropan-2-yl)pyridin-2-yl]-6-{[4-(4-methylpiperazin-1-yl)phenyl]amino}-2-(prop-2-en-1-yl)-1,2-dihydro-3H-pyrazolo[3,4-d]pyrimidin-3-one
5VD1	1.7	Membrane-associated tyrosine- and threonine-	P48	N,1,4,4-TETRAMETHYL-8-{[4-(4-METHYLPIPERAZIN-1-YL)PHENYL]AMINO}-4,5-DIHYDRO-1H-PYRAZOLO[4,3-H]QUINAZOLINE-3-CARBOXAMIDE
5VEE	2.5	Serine/threonine-protein kinase PAK 4	981	6-(2,4-dichlorophenyl)-8-ethyl-2-{[3-fluoro-4-(piperazin-1-yl)phenyl]amino}pyrido[2,3-d]pyrimidin-7(8H)-one
5VFI	1.59	Tyrosine-protein kinase BTK	9AJ	2-[3'-(hydroxymethyl)-1-methyl-5-({5-[(2S)-2-methyl-4-(oxetan-3-yl)piperazin-1-yl]pyridin-2-yl}amino)-6-oxo[1,6-dihydro[3,4'-bipyridine]]-2'-yl]-7,7-dimethyl-3,4,7,8-tetrahydro-2H-cyclopenta[4,5]pyrrolo[1,2-a]pyrazin-1(6H)-one
5VHB	1.61	cAMP-dependent protein kinase	9CY	N-[(2S)-1-hydroxy-3-phenylpropan-2-yl]-4-(pyridin-4-yl)benzamide
5VI9	1.95	cAMP-dependent protein kinase	9D4	N-[(3-fluorophenyl)methyl]-6-(pyridin-4-yl)-1,3-benzothiazol-2-amine
5VIB	2.37	cAMP-dependent protein kinase	9D1	3-({[6-(pyridin-4-yl)-1,3-benzothiazol-2-yl][2-(pyrrolidin-1-yl)ethyl]amino}methyl)phenol
5VLO	2.05	Calcium/calmodulin-dependent protein kinase	9EJ	N-[(2S)-2-(diethylamino)propyl]-2-[(2S)-2-(methylcarbamoyl)azetidin-1-yl]-6-[5-(thiophen-2-yl)pyrazolo[1,5-a]pyrimidin-3-yl]pyridine-4-carboxamide
5VND	2.2	Fibroblast growth factor receptor 1	9ES	N-{2-[(6-{[(2,6-dichloro-3,5-dimethoxyphenyl)carbamoyl](methyl)amino}pyrimidin-4-yl)amino]-5-(4-ethylpiperazin-1-yl)phenyl}propanamide
5VO1	2.45	MAP kinase kinase kinase 12	9FS	5-{5-[(1R,5S,6r)-3-(oxetan-3-yl)-3-azabicyclo[3.1.0]hexan-6-yl]-1-(propan-2-yl)-1H-pyrazol-3-yl}-3-(trifluoromethyl)pyridin-2-amine
5VT1	1.9	Calcium/calmodulin-dependent protein kinase	9JS	4-({5-[(3-hydroxy-4-methylphenyl)amino]-4-oxo-4H-1,2,6-thiadiazin-3-yl}amino)benzamide
5VUA	2.2	Serine/threonine-protein kinase pim-1	8GX	(2Z)-6-methoxy-7-(piperazin-1-ylmethyl)-2-(1H-pyrrolo[2,3-c]pyridin-3-ylmethylidene)-1-benzofuran-3-one
5VUB	2	Serine/threonine-protein kinase pim-1	8GU	(2Z)-6-methoxy-7-(piperazin-1-ylmethyl)-2-(1H-pyrrolo[3,2-b]pyridin-3-ylmethylidene)-1-benzofuran-3-one
5VUC	2	Serine/threonine-protein kinase pim-1	8GR	(2Z)-2-(1H-indol-3-ylmethylidene)-6-methoxy-7-(piperazin-1-ylmethyl)-1-benzofuran-3-one
5W4W	1.99	Casein kinase I	9WG	4-[3-(4-fluorophenyl)-1-methyl-1H-pyrazol-4-yl]-6-methyl-6,7-dihydro-5H-pyrrolo[3,4-b]pyridin-5-one
5W80	2	Calmodulin-domain protein kinase 1	9XJ	3-(3-chlorophenoxy)-1-[(piperidin-4-yl)methyl]-1H-pyrazolo[3,4-d]pyrimidin-4-amine
5W85	2.25	Interleukin-1 receptor-associated kinase 4	9YS	6-[(1,3-benzothiazol-6-yl)amino]-4-{[(2S)-1-hydroxy-3-phenylpropan-2-yl]amino}-N-methylpyridine-3-carboxamide
5W8R	2.2	Calmodulin-domain protein kinase 1	9XV	1-tert-butyl-3-[(3-chlorophenyl)methyl]-1H-pyrazolo[3,4-d]pyrimidin-4-amine
5W91	2.4	Calmodulin-domain protein kinase 1	9XP	1-tert-butyl-N~3~-(3-chlorophenyl)-1H-pyrazolo[3,4-d]pyrimidine-3,4-diamine
5W9E	2.44	Calmodulin-domain protein kinase 1	9YJ	1-tert-butyl-3-[(3-chlorophenyl)sulfanyl]-1H-pyrazolo[3,4-d]pyrimidin-4-amine
5WAL	2.45	Non-receptor tyrosine-protein kinase TYK2	9ZS	N-[2-(2,6-dichlorophenyl)-1H-imidazo[4,5-c]pyridin-4-yl]cyclopropanecarboxamide
5WFJ	2.48	Tyrosine-protein kinase JAK3	9Z4	4-({[3-(propanoylamino)phenyl]methyl}amino)pyrrolo[1,2-b]pyridazine-3-carboxamide
5WG4	2.31	Beta-adrenergic receptor kinase 1	AFV	2-fluoro-5-[(3S,4R)-3-{[(1H-indazol-5-yl)oxy]methyl}piperidin-4-yl]-N-[(pyridin-2-yl)methyl]benzamide
5WJJ	1.6	MAP kinase 14	AQY	N-{4-[2-(4-fluoro-3-methylphenyl)imidazo[1,2-b]pyridazin-3-yl]pyridin-2-yl}-2-methyl-1-oxo-1lambda~5~-pyridine-4-carboxamide
5WO4	1.84	Tyrosine-protein kinase JAK1	B7V	3-[(4-chloro-3-methoxyphenyl)amino]-1-[(3R,4S)-4-cyanooxan-3-yl]-1H-pyrazole-4-carboxamide
5X02	2.401	Receptor-type tyrosine-protein kinase FLT3	F6M	N-[(2S)-1-[5-[2-[(4-cyanophenyl)amino]-4-(propylamino)pyrimidin-5-yl]pent-4-ynylamino]-1-oxidanylidene-propan-2-yl]-4-(dimethylamino)-N-methyl-but-2-enamide
5X2A	1.85	Epidermal growth factor receptor	7XO	N2-[4-(4-methylpiperazin-1-yl)phenyl]-N8-phenyl-9-propan-2-yl-purine-2,8-diamine
5X2C	2.05	Epidermal growth factor receptor	7XR	9-cyclopentyl-N2-[4-(4-methylpiperazin-1-yl)phenyl]-N8-phenyl-purine-2,8-diamine
5X2F	2.2	Epidermal growth factor receptor	7XU	9-cyclohexyl-N2-[4-(4-methylpiperazin-1-yl)phenyl]-N8-phenyl-purine-2,8-diamine
5X5O	1.868	MAP kinase kinase kinase MLT	7Z0	N-[2,4-bis(fluoranyl)-3-[2-(3-methoxy-1H-pyrazolo[3,4-b]pyridin-5-yl)ethynyl]phenyl]-3-bromanyl-benzenesulfonamide
5X8I	1.902	Dual specificity protein kinase CLK1	SQZ	5-[1-[(1S)-1-(4-fluorophenyl)ethyl]-[1,2,3]triazolo[4,5-c]quinolin-8-yl]-1,3-benzoxazole
5XDK	2.346	Epidermal growth factor receptor	8JC	N-[3-[[2-[[4-(4-ethanoylpiperazin-1-yl)-2-methoxy-phenyl]amino]-5-(trifluoromethyl)pyrimidin-4-yl]amino]phenyl]prop-2-enamide
5XQX	2.3	Cyclin-dependent kinase 8	8CC	N-methyl-4-pyridin-4-yl-1H-pyrrole-2-carboxamide
5XS2	2.04	Cyclin-dependent kinase 8	8D6	3-chloranyl-4-pyridin-4-yl-1H-pyrrole-2-carboxamide
5XV7	2.32	serine-arginine (SR) protein kinase 1	EMH	9-ethyl-6,6-dimethyl-8-[4-(morpholin-4-yl)piperidin-1-yl]-11-oxo-6,11-dihydro-5H-benzo[b]carbazole-3-carbonitrile
5XVA	1.847	Serine/threonine-protein kinase PAK 4	8FU	[6-chloranyl-4-[(5-methyl-1H-pyrazol-3-yl)amino]quinazolin-2-yl]-[(3R)-3-methylpiperazin-1-yl]methanone
5XVG	2.1	Serine/threonine-protein kinase PAK 4	8FX	[6-chloranyl-4-[(5-cyclopropyl-1H-pyrazol-3-yl)amino]quinazolin-2-yl]-[(3R)-3-methylpiperazin-1-yl]methanone
5XYY	1.7	MAP kinase 14	P0F	3-(5-{[(2-chloro-6-fluorophenyl)methyl]amino}-4H-1,2,4-triazol-3-yl)phenol
6AUD	2.015	Phosphatidylinositol 4,5-bisphosphate 3-kinase	BWY	10-[(S)-(1-tert-butylpiperidin-4-yl)sulfinyl]-2-[1-(propan-2-yl)-1H-1,2,4-triazol-5-yl]-5,6-dihydroimidazo[1,2-d][1,4]benzoxazepine
6AYW	2.05	Calcium/calmodulin-dependent protein kinase	C2V	N-[2-(dimethylamino)ethyl]-3-[6-(thiophen-2-yl)imidazo[1,2-b]pyridazin-3-yl]benzamide
6B16	2.285	Serine/threonine-protein kinase PAK 1	C7Y	N~4~-(5-cyclopropyl-1H-pyrazol-3-yl)-N~2~-[(1S)-1-(1H-pyrrolo[3,2-b]pyridin-5-yl)ethyl]pyrimidine-2,4-diamine
6B8Y	1.65	TGF-beta receptor type-1	D0A	N-(3-fluoropyridin-4-yl)-2-[6-(trifluoromethyl)pyridin-2-yl]-7H-pyrrolo[2,3-d]pyrimidin-4-amine
6BAB	1.91	Calcium/calmodulin-dependent protein kinase	D0S	N-[(2S)-2-(diethylamino)propyl]-2-(3-hydroxyazetidin-1-yl)-6-[5-(thiophen-2-yl)pyrazolo[1,5-a]pyrimidin-3-yl]pyridine-4-carboxamide

6BBV	1.8	Tyrosine-protein kinase JAK2	D7D	N-{cis-3-[methyl(7H-pyrrolo[2,3-d]pyrimidin-4-yl)amino]cyclobutyl}propane-1-sulfonamide
6BFN	2.26	Interleukin-1 receptor-associated kinase 1	DL1	N-[2-methoxy-4-(morpholin-4-yl)phenyl]-6-(1H-pyrazol-5-yl)pyridine-2-carboxamide
6BKU	2	Calcium/calmodulin-dependent protein kinase	DXV	2-cyclopentyl-4-(5-phenyl-1H-pyrrolo[2,3-b]pyridin-3-yl)benzoic acid
6BP0	1.9	Serine/threonine-protein kinase VRK1	E1D	(7R)-2-[(3,5-difluoro-4-hydroxyphenyl)amino]-5,7,8-trimethyl-7,8-dihydropteridin-6(5H)-one
6BQL	2	Calcium/calmodulin-dependent protein kinase	BI9	2-({5-CHLORO-2-[(2-METHOXY-4-MORPHOLIN-4-YLPHENYL)AMINO]PYRIMIDIN-4-YL}AMINO)-N-METHYLBENZAMIDE
6BQP	1.95	Calcium/calmodulin-dependent protein kinase	6T2	1-(2-{5-[(3-Methyloxetan-3-yl)methoxy]-1H-benzimidazol-1-yl}quinolin-8-yl)piperidin-4-amine
6BRC	2.2	Calcium/calmodulin-dependent protein kinase	E5J	5-chloro-N~2~-{4-[4-(dimethylamino)piperidin-1-yl]-2-methoxyphenyl}-N~4~-[2-(dimethylphosphoryl)phenyl]pyrimidine-2,4-diamine
6BRU	1.8	Serine/threonine-protein kinase VRK1	E5M	(7S)-2-[(3,5-difluoro-4-hydroxyphenyl)amino]-5,7,8-trimethyl-7,8-dihydropteridin-6(5H)-one
6BTW	1.9	Serine/threonine-protein kinase VRK1	E8D	2-[(3,5-difluoro-4-hydroxyphenyl)amino]-8-phenyl-7,8-dihydropteridin-6(5H)-one
6BU6	1.8	Serine/threonine-protein kinase VRK1	E8V	4,4'-(2-aminopyridine-3,5-diyl)bis(2,6-difluorophenol)
6CFM	2.45	Serine/threonine-protein kinase VRK1	EA7	(7R)-2-[(3,5-difluoro-4-hydroxyphenyl)amino]-7,8-dimethyl-5-(prop-2-yn-1-yl)-7,8-dihydropteridin-6(5H)-one
6EHK	1.4	Casein kinase II	AUW	2-(1~{H}-benzimidazol-2-yl)-~{N}-[[3,5-bis(chloranyl)-4-(2-ethylphenyl)phenyl]methyl]ethanamine
6EHU	1.95	Casein kinase II	B5E	2-(1~{H}-benzimidazol-2-yl)-~{N}-[[4-(2-ethylphenyl)-3-(trifluoromethyl)phenyl]methyl]ethanamine
6EII	1.935	Casein kinase II	B5W	(3-chloranyl-4-phenyl-phenyl)methyl-(3-phenylpropyl)azanium
6EIM	1.43	Serine/threonine-protein kinase 10	B6E	~{N}-[5-[4-[[2-fluoranyl-5-(trifluoromethyl)phenyl]carbamoylamino]phenoxy]-1~{H}-benzimidazol-2-yl]furan-2-carboxamide
6ES0	2.38	Receptor-interacting serine/threonine-protein	BW8	2-[2-fluoranyl-4-[[2-fluoranyl-4-[2-(methylcarbamoyl)pyridin-4-yl]oxy-phenyl]carbamoylamino]phenyl]sulfanylethanoic acid
6EYZ	2.2	Phosphatidylinositol 4,5-bisphosphate 3-kinase	C5W	2-methoxy-5-[4-[5-[(4-propan-2-ylpiperazin-1-yl)methyl]-1,3-oxazol-2-yl]-2~{H}-indazol-6-yl]pyridine-3-carboxylic acid
6EZ6	2.04	Phosphatidylinositol 4,5-bisphosphate 3-kinase	C5Z	methyl 2-methoxy-5-[4-[5-[(4-propan-2-ylpiperazin-1-yl)methyl]-1,3-oxazol-2-yl]-2~{H}-indazol-6-yl]pyridine-3-carboxylate
6FC8	1.61	Serine/threonine-protein kinase Chk1	D4Q	2-(3-fluorophenyl)-4-[[(3~{S})-piperidin-3-yl]amino]thieno[3,2-c]pyridine-7-carboxamide
6FCF	1.85	Serine/threonine-protein kinase Chk1	D58	4-[[(2~{R},3~{S})-2-methylpiperidin-3-yl]amino]-2-phenyl-thieno[3,2-c]pyridine-7-carboxamide
6FCK	1.9	Serine/threonine-protein kinase Chk1	D4Z	2-phenyl-4-[[(3~{S})-piperidin-3-yl]amino]-1~{H}-indole-7-carboxamide
6MX8	1.96	ALK tyrosine kinase receptor	6GY	5-chloro-N~4~-[2-(dimethylphosphoryl)phenyl]-N~2~-{2-methoxy-4-[4-(4-methylpiperazin-1-yl)piperidin-1-yl]phenyl}pyrimidine-2,4-diamine
